# Alkynyl *Halo-*Prins Cyclizations for the Synthesis of Bicyclo[4.3.1] and [3.3.1] Oxygen-Bridged Heterocycles

**DOI:** 10.1021/acs.joc.5c01745

**Published:** 2025-09-09

**Authors:** Yusuf A. Ibrahim, Alison J. Frontier

**Affiliations:** Department of Chemistry, 6927 University of Rochester, 120 Trustee Road, Rochester, New York 14627, United States

## Abstract

This report presents the alkynyl *halo*-Prins cyclization of Achmatowicz adducts, enabling the synthesis of up to 24 (24) highly functionalized [4.3.1] and [3.3.1] heterocycles containing a bridging oxygen. The substrate scope demonstrates that this method is compatible with a range of substitution patterns and ring substituents and does not appear to depend upon the electronic nature of the ring system. Furthermore, the reaction can be carried out on a multigram scale without a significant loss in yield. To showcase the versatility of these oxacycles as synthetic tools, we explore their application in various chemical transformations, including cross-coupling reactions, [4 + 2] cycloaddition, cyclopropanation, addition, and reduction reactions, ultimately facilitating the preparation of a modest compound library.

## Graphical abstract

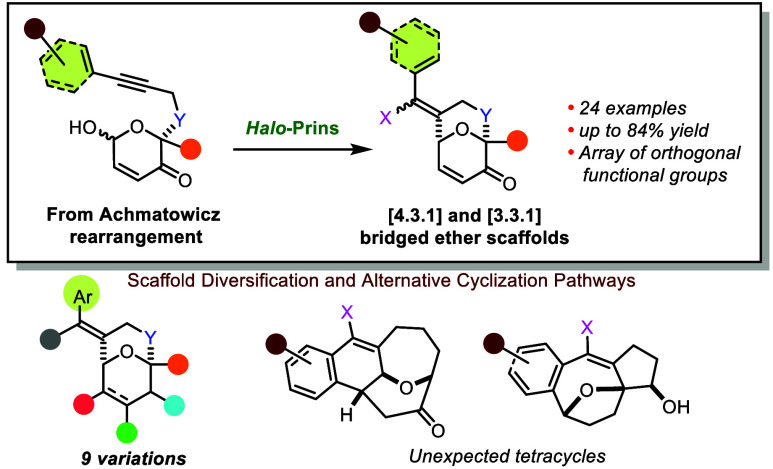

## Introduction

Oxygen heterocycles (O-heterocycles) are widely occurring structural units commonly found in various biologically active natural products and functional molecules.^1–5^ Bicyclic molecules with bridging oxygen adopt a rigid three-dimensional (3D) architecture, offering a chiral scaffold that may represent a novel shape and position within chemical space.^6,7^ If synthetic handles can be arrayed across this type of scaffold, they could be selectively modified to maximize appendage diversity.^8,9^

Over the years, various methods have been developed to synthesize bridged oxacycles. Generally speaking, these ring systems can be assembled using three different strategies: through inter- or intramolecular dipolar cycloaddition reactions of cyclic carbonyl ylide reactants (Scheme 1A),^10–17^ intramolecular cyclization onto a cyclic oxocarbenium ion intermediate (Scheme 1B),^18,19^ or Stevens rearrangement of fused bicyclic oxonium ylides (Scheme 1C).^20–23^ Most commonly, these bridged systems are constructed via the cycloaddition of a pyrylium ion intermediate with some type of dipolarophile (Scheme 1A). Variants include dipolar [3 + 3],^14,15^ [5 + 2],^10–13^ [5 + 3],^16,17^ or [6 + 4][Bibr ref24] cycloadditions. In some cases, the pyrylium ion intermediates are derived from Achmatowicz rearrangement products.^10,11,24^ In contrast to the large body of work exploring the cycloaddition mode of assembly, the oxocarbenium cyclization approach (Scheme 1B) is largely underdeveloped. Both intramolecular Friedel–Crafts^19^ and classical Prins cyclizations (alkene nucleophile) have been described.^25^ However, while it is well established that alkynyl Prins reactions can be employed to build fused bicyclic systems,^26^ construction of bridged bicyclic systems is less common. Of these methods, aza-Prins cyclizations that deliver bridged N-heterocycles dominate,^27–29^ with just one example producing a bicyclic scaffold with a bridging oxygen.^30^ Further exploration of this strategy could offer access to new classes of bridged oxygen heterocycles.

**Scheme 1:**
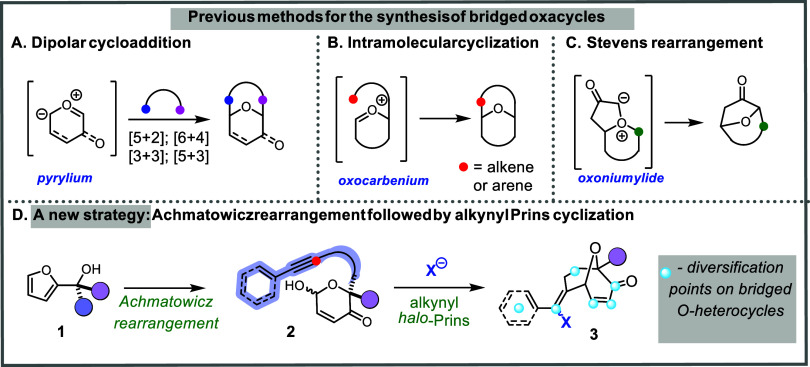
Cyclization Strategies Targeting Bridged Oxacycles^a^

a (A) Intermolecular dipolar cycloaddition (carbonyl ylide/oxidopyrylium partner), (B) intramolecular cyclization (oxycarbenium intermediate), (C) Stevens rearrangement (via oxonium ylide), and (D) Achmatowicz rearrangement followed by alkynyl Prins cyclization.

Our ongoing interest in *halo*-Prins chemistry^31–35^ led us to investigate the alkynyl Prins cyclization of Achmatowicz rearrangement adducts **2**, to enable the construction of highly functionalized bridged oxacycles **3** (Scheme 1D). In this work, we describe how this approach can deliver novel scaffolds that not only contain the bridged oxygen feature in the core but also carry multiple orthogonal functional groups for additional diversification. Synthetic handles are marked in pale blue in Scheme 1D. Such building blocks could serve as a versatile starting point for the preparation of novel small molecules for biological evaluation.

## Results and Discussion

### Alkynyl *Halo*-Prins Cyclization

To initiate the study, target reactants **2** were prepared using Achmatowicz rearrangement,^36,37^ wherein a furfuryl alcohol (**1**) is transformed into the desired dihydropyranone (**2**; cf. Scheme 1D. See Supporting Information for details). With **2a** in hand, we were ready to test the alkynyl *halo*-Prins cyclization as a means to construct **3a**, a [4.3.1]-bicyclic molecule containing a bridging oxygen. In earlier studies, *halo*-Prins cyclization was optimally achieved using triflic acid (TfOH) as the promoter and tetrabutylammonium iodide (TBAI) as the halide source,^38^ but these conditions proved ineffective for reactant **2a**.

Consequently, we turned our attention to exploring various Lewis acids as alternatives (Table 1). In early tests, we found that neither trimethylsilyl bromide (TMSBr) nor B­(C_6_F_5_)_3_ in the presence of tetrabutylammonium bromide (TBAB), is effective in promoting cyclization. However, a series of transition metal salts in chloroform (CHCl_3_) produces **3a** in yields ranging from 19 to 49% (entries 1–6). Indium trichloride (InCl_3_), indium iodide (InI_3_), and indium tribromide (InBr_3_) give comparatively higher yields (40–49%) relative to bismuth tribromide (BiBr_3_) and iron tribromide (FeBr_3_). Notably, a catalytic amount of BiBr_3_ furnishes the desired cyclization product **3a** in 19% yield, along with a 11% yield of the Friedel–Crafts tetracycle **4a** (entry 5). Also, when FeBr_3_ is employed as both the catalyst and halide source, the yield of **3a-(** ***Z*** **)** is found to be 27%, with a 9% yield of **4a** (entry 6). In addition, the use of sodium perchlorate (NaClO_4_) (1 equiv) as an additive improves reaction yields (See Supporting Information for details).

**Table 1:**
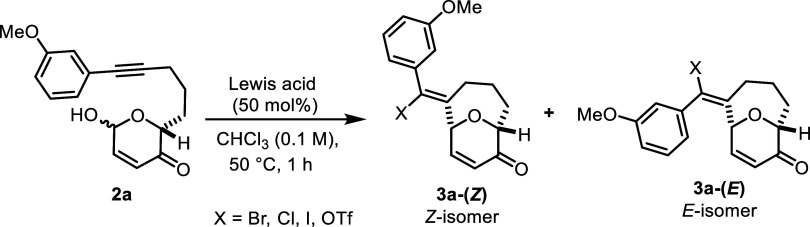
Alkynyl *Halo*-Prins Cyclizations: Lewis Acid Catalyst Screen^a^

entry	Lewis acid	**3a** (*Z*/*E*)
**1**	In(OTf)_3_	25% (2.3:1)
**2**	InCl_3_	49% (2.4:1)
**3**	InBr_3_	40% (4:1)^b^
**4**	InI_3_	42% (6.3:1)
**5**	BiBr_3_	19% (18:1)^c^
**6**	FeBr_3_	27%^c^

a 0.2 mmol of **2a**, isolated yield.

b less than 10% isolated yield of **4a**.

c 11% isolated yield of **4a** observed; The ratio in parentheses is the ratio of **3a-(** ***Z*** **)**:**3a-(** ***E*** **)** determined by ^1^H NMR analysis.
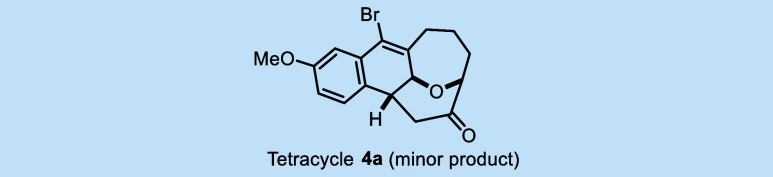

Ultimately, employing a catalytic amount of InBr_3_ along with NaClO_4_ in CHCl_3_ at 50 °C is optimal, affording **3a** in 59% yield as a mixture of nearly inseparable *Z*/*E*-isomers (Table 2). The structure of the major isomer is found to have Z-geometry, according to analysis using Nuclear Overhauser Effect Spectroscopy (NOESY) (see Supporting Information for details). Substituting InBr_3_ with InCl_3_ or InI_3_ under the same conditions gives lower yields of 36 and 37%, respectively (Table 2, entries 2 and 3). We also found that modifying the loading of InBr_3_ influenced the yield: both lower and higher catalyst loadings result in decreased yields of 33 and 34% (entries 4 and 5), compared to the optimal 59% yield (entry 1). Performing the reaction at room temperature furnished **3a** in 46% yield (entry 6). Results with DCE or DBE were also suboptimal (entries 7 and 8). Furthermore, the yields of **3a** decrease whenever we conduct the reaction in other solvents or in the presence of 5Å molecular sieves (See Supporting Information for details).

**Table 2:**
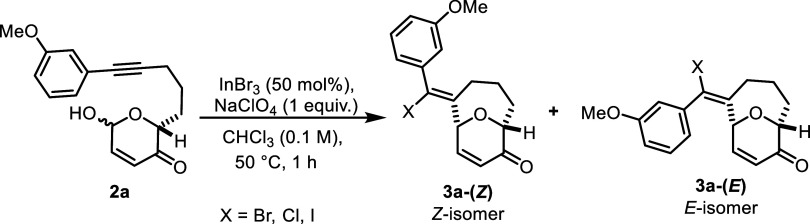
Reaction Optimization for Alkynyl *Halo*-Prins Cyclization^a^

entry	deviation	**3a** (*Z*/*E*)^b^
**1**	none	59% (2.5:1)
**2**	InCl_3_ instead of InBr_3_	36% (1.9:1)
**3**	InI_3_ instead of InBr_3_	37% (6:1)
**4**	InBr_3_ (30 mol % instead of 50 mol %)	33% (1.9:1)
**5**	InBr_3_ (1 equiv instead of 50 mol %)	34%
**6**	room temp. instead of 50 °C	46% (2.6:1)
**7**	DCE, rt instead of CHCl_3_, 50 °C	41% (1.5:1)
**8**	DBE instead of CHCl_3_	28%

a 0.2 mmol of **2a**, isolated yield.

b The ratio in parentheses is the ratio of **3a-(** ***Z*** **)**:**3a-(** ***E*** **)** determined by ^1^H NMR analysis.

With the optimized reaction conditions in place, we investigated the reaction using substrates with varying electronic properties, including electron-donating groups (EDGs) and electron-withdrawing groups (EWGs) (Scheme 2). For Achmatowicz adducts **2** bearing an EDG at C-2, the corresponding products **3b** and **3c** are obtained in moderate yields of 68 and 57% respectively, and the simple phenyl enyne delivers product **3d** in 67% yield. Substrates with EWGs at C-2 (**2e** and **2f**) afford the expected products **3e** and **3f** in moderate yields of 64 and 60%, respectively.

**Scheme 2:**
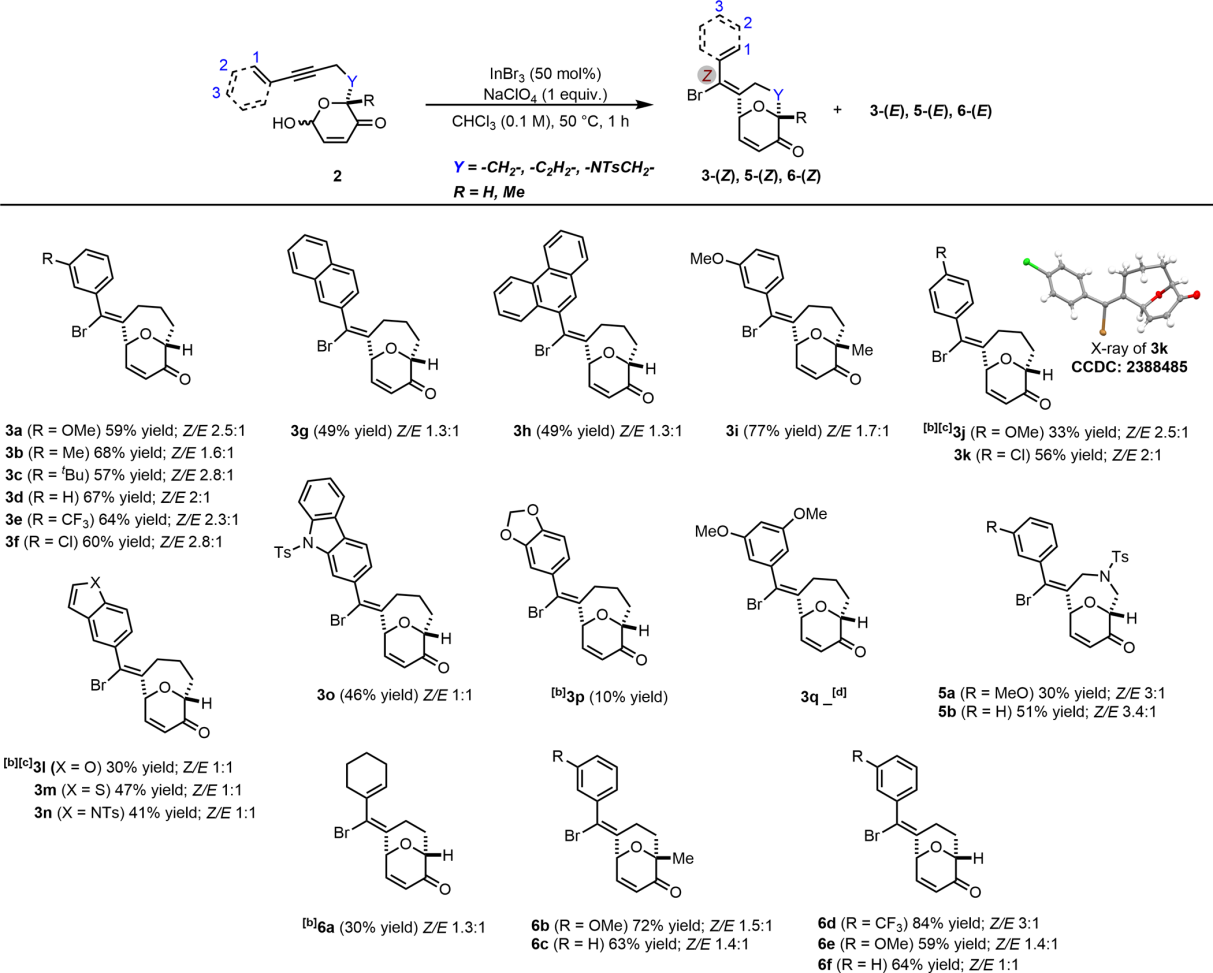
Synthesis of Bridged Oxacycles Using Alkynyl *Halo*-Prins Cyclization (Substrate Scope)^a^

a 0.2 mmol of **2**, anhydrous CHCl_3_.
b Reaction at room temperature.
c Yield based on recovered SM.
d Observed no product. The reactions were stopped immediately after complete consumption of **2**, as determined by thin-layer chromatography, to minimize decomposition of the *E*-isomer caused by prolonged exposure to the reaction conditions.

Naphthalene (**2g**) and phenanthrene (**2h**), undergo *halo*-Prins cyclization to afford **3g** and **3h** in 49% yield. Replacing the R-group with methyl group **2i** produces the anticipated product **3i** in 77% yield. Achmatowicz adducts **2** with an EDG and EWG at C-3 reacts smoothly to generate the desired products **3j** and **3k** (33 and 56% yield). The structure of the major isomer of **3k** (*Z*-isomer) is determined by X-ray crystallography (CCDC: 2388485). To further evaluate this method’s versatility, we examined heteroaromatic rings such as benzofuran, benzothiophene, indole, and carbazole, which furnish products **3l**–**3o** in 30–47% yield. The acetal adduct **2p** delivers **3p** in a disappointing 10% yield. In this case, substrate instability requires a lower reaction temperature. Unfortunately, the desired product **3q** is not observed when we examined the highly electron-rich Achmatowicz adduct **2q**.

Different scaffold classes can also be prepared using this strategy: with a nitrogen atom in the tether, **5a** and **5b** are produced in 30 and 51% yields, respectively. Additionally, substrates with a shorter tether (**2s**–**2x**) produce a [3.3.1] bridged bicyclic scaffold in moderate to high yields (**6a**–**6f**; 30–84% yield). Overall, the reaction rate and yield were generally unaffected by the tether length (Scheme 2).

To round out the study, we tested the process on a larger scale, leveraging the optimized reaction conditions to synthesize both [4.3.1] and [3.3.1] bridged oxygen heterocycles **3** (Figure 1). Achmatowicz adducts **2d**, **2v** and **2x** were subjected to the developed *halo*-Prins reaction on a gram scale, affording the corresponding products **3d**, **6d** and **6f** in high yields (65, 75, and 61% yield), comparable to those obtained on a 0.2 mmol scale (Scheme 2). These results demonstrate the scalability of the method.

**Figure 1:**
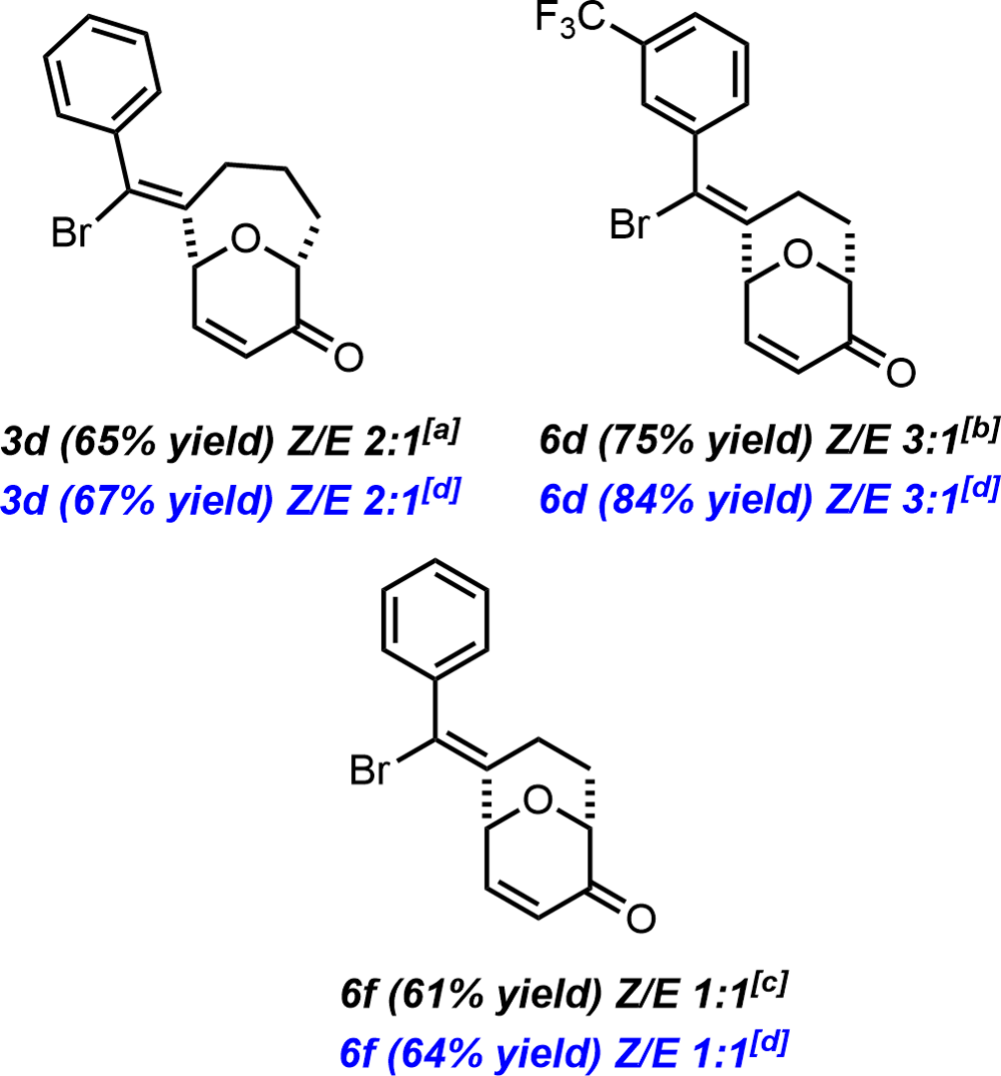
Gram-scale synthesis of vinyl bromides **3** and **6**. Reaction conditions: InBr_3_ (50 mol %), NaClO_4_ (1 equiv), CHCl_3_ (0.1 M), 50 °C, 1 h ^[a]^8.2 mmol of **2d**; ^[b]^3.23 mmol of **2v**; ^[c]^9.9 mmol of **2x**; ^[d]^0.2 mmol of **2**.

The mechanism for the Lewis acid-catalyzed *halo*-Prins cyclization is proposed as shown in Scheme 3. It is likely that the active species in this reaction is indium perchlorate (In­(ClO_4_)_3_), which forms in situ.^39,40^ The In­(ClO_4_)_3_ facilitates the elimination of the OH group from **2**, generating oxocarbenium intermediate **II**. The distal carbon of the arenyne cyclizes in a transannular fashion, and halide trapping delivers **3**, presumably via stabilized vinyl cation intermediate **III**. Two angles of attack are available to the terminal halide nucleophile, generating isomers **3-(** ***Z*** **)** and **3**-**(** ***E*** **)**. While the *E*/*Z* selectivity is modest at best, the **3-(** ***Z*** **)** isomer is dominant in many cases (Scheme 2).

**Scheme 3:**
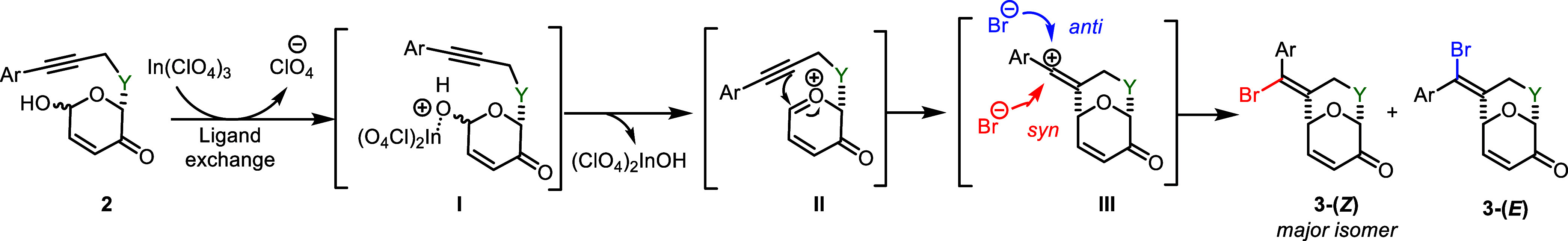
Proposed Mechanism for InBr_3_/NaClO_4_–Catalyzed *Halo*-Prins Cyclization

It is worth noting that in most published examples, alkynyl *halo*-Prins cyclizations exhibit very high *E* selectivity,^26^ in contrast to the Scheme 2 cases. We believe the primary factor affecting the *Z*/*E* ratios in these reactions is not the inherent selectivity of the cyclization step but rather the high reactivity of the *E*-isomer under the reaction conditions. Prolonged reaction time leads to the erosion of *Z*/*E* selectivity, driven by competing reactions involving the *E*-isomer. We imagine that the reaction of distal and proximal carbons of the alkyne may be largely synchronous in conventional alkynyl *halo*-Prins reactions, installing the halide nucleophile *trans* to the oxocarbenium electrophile (*E*-isomer). However, the transannular cyclizations reported here must occur stepwise since the vinyl halide product geometry reflects both *syn* and *anti* addition to the oxocarbenium electrophile (Scheme 3).

### Selective Functionalization of Bicyclo[3.3.1]­nonane Oxacyclic Scaffold **6**

The synthetic versatility of the type **6** scaffolds, which contain an electrophilic enone, a vinyl halide handle, and a ketone, offers the opportunity to achieve differential decoration of the building blocks using orthogonal chemical operations. Using **6f** as the model substrate, we identified several chemical transformations that can be used to diversify the scaffolds (Scheme 4). Heck cross-coupling reaction of **6f** with styrene in the presence of Pd­(OAc)_2_ yields **7a** (73% yield).^41^ Suzuki–Miyaura cross-coupling of the vinyl bromide with phenylboronic acid leads to **7b** in 89% yield.^38^ Conjugate reduction of the enone, catalyzed by Pd­(PPh_3_)_4_ and zinc chloride (ZnCl_2_) in the presence of diphenylsilane (Ph_2_SiH_2_) as the reducing agent, delivers **7c** (72% yield),^42^ which can be reduced diastereoselectively with sodium borohydride (NaBH_4_), to afford alcohol **7d** in 51% yield. Chemo-and diastereoselective reduction of the enone can be achieved using in situ prepared NaH*_n_* B­(OMe)_4–*n*_, to generate allylic alcohol **7e** (70% yield).^43,44^ Methylated adducts are accessible through diastereoselective 1,2-addition with the Grignard reagent (**7f**, 67% yield) and 1,4-addition with organocuprate (**7g**, 58% yield). Corey–Chaykovsky cyclopropanation^45^ of **6f** affords the corresponding product **7h** in 72% yield. [4 + 2] cycloaddition of **6f** with cyclopentadiene in the presence of ZnCl_2_ ^46^ yields **7i** in 86%. The stereochemistry of **7i** is assigned using 2D-NOESY experiments (see Supporting Information for details).^24,47^ All transformations on the bicyclic core occur with complete diastereoselectivity, influenced by the rigid cup-shaped conformation of the bridged [3.3.1] system.

**Scheme 4:**
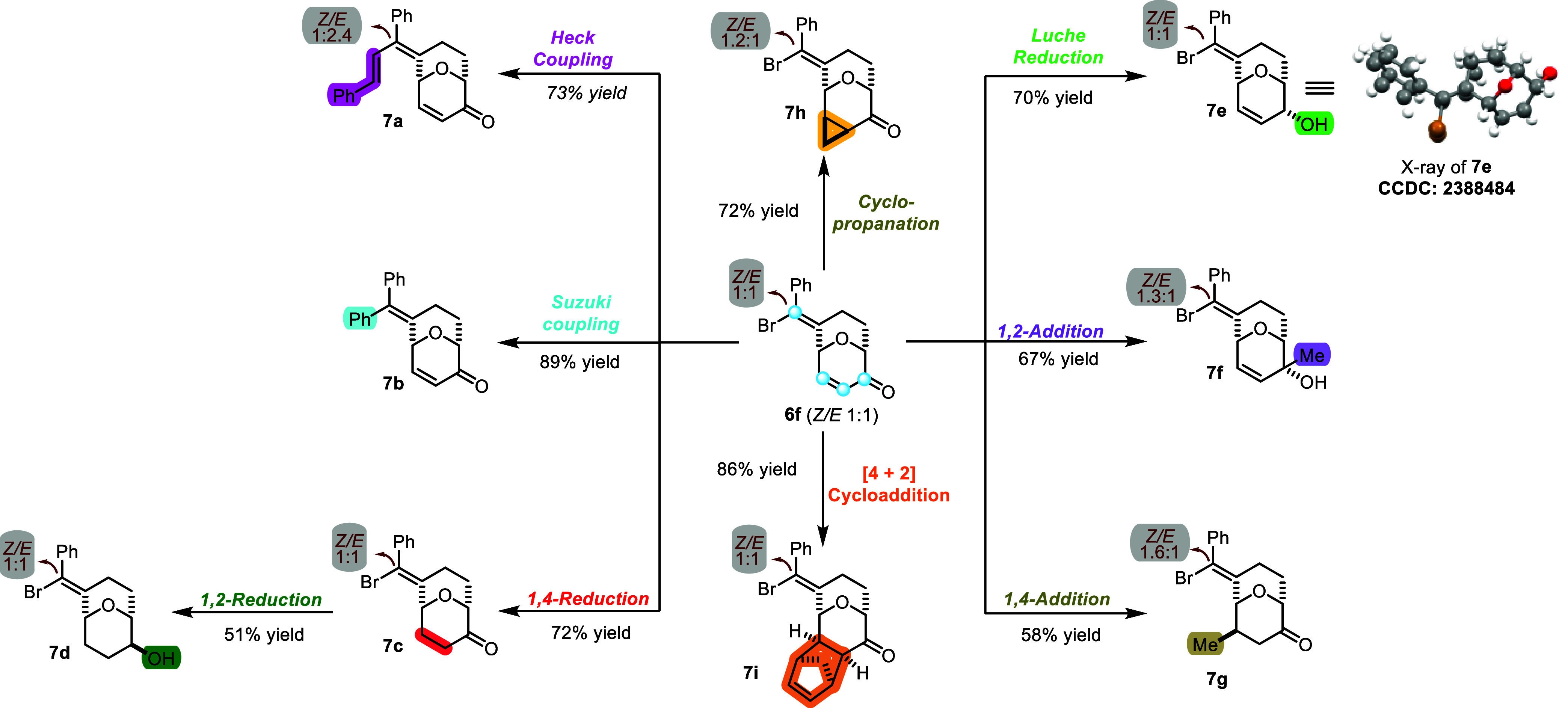
Transformation of the Dihydropyranone Scaffolds into Diverse Bridged Oxacycles and Other Derivatives^a^

a Reaction conditions: Heck coupling (**7a**): **6f**, styrene, Pd­(OAc)_2_, PPh_3_, DMF, 100 °C. Suzuki coupling (**7b**): **6f**, PhB­(OH)_2_, Pd­(PPh_3_)_4_, Na_2_CO_3_ (aq.), toluene, EtOH, 80 °C. 1,4-Reduction (**7c**): **6f**, Ph_2_SiH_2_, Pd­(PPh_3_)_4_, ZnCl_2_, CHCl_3_, rt. 1,2-Reduction (**7d**): **7c**, NaBH_4_, MeOH, THF, 0 °C. Luche reduction (**7e**): **6f**, CeCl_3_, MeOH, NaBH_4_, DCM, −78 °C. 1,2-Addition of Grignard (**7f**): **6f**, MeMgCl, THF, −78 °C. 1,4-Addition of Organocuprate (**7g**): **6f**, MeLi, CuI, Et_2_O, 0 °C. Cyclopropanation (**7h**): **6f**, Me_3_SO^+^I^–^, NaH, DMSO, rt. Cycloaddition (**7i**): **6f**, cyclopentadiene, ZnCl_2_, benzene, rt.

### Unexpected Cyclizations Generate Tetracyclic Scaffolds

Over the course of this alkynyl Prins cyclization study, we observed the formation of two types of unusual oxygen heterocycles (**4** and **10**), which arise from unanticipated reaction pathways. Both are tetracyclic structures and contain the bridging oxygen motif. These novel transformations appear to be without precedent.

***The bridging oxygen-containing tetracycle 4a*** was initially observed as a minor component in the alkynyl Prins cyclization of **2a**, promoted by BiBr_3_ in CHCl_3_ (Table 1, entry 5). When a *Z*/*E* mixture of **3a** (1.9:1) is treated with BiBr_3_, we isolate **4a** (13% yield) (Scheme 5A­(i)). Leftover reactant **3a** is also isolated from this experiment, but interestingly, the *Z*/*E* ratio had increased (3.8:1). When this *Z*-enriched batch of **3a** was resubjected to the reaction conditions, we again obtain **4a** (20% yield), along with **3a** (55% yield), this time as a single diastereoisomer (Scheme 5A­(ii)). And, when a sample of pure **3a-(** ***Z*** **)** is subjected to the same conditions, no reaction occurs (Scheme 5A­(iii)). These observations lead us to hypothesize that during the Prins cyclization, the catalyst can further activate the carbonyl enone of **3a-(** ***E*** **)**, promoting an intramolecular Friedel–Crafts reaction to form intermediate **IV**. Rearomatization of **IV** to form **V**, followed by tautomerization of intermediate **V**, produces bridged oxacycle **4a** (Scheme 5B). Indeed, when a pure sample of **3b-(** ***E*** **)** is treated with BiBr_3_ in DCM, **4b** is isolated in 80% yield (Scheme 5C), consistent with the proposed mechanism. It is noteworthy that no reaction occurs when oxacycle **6f** (shorter tether length) is subjected to BiBr_3_ conditions (Scheme 5D).

***The second unexpected tetracyclic structure (10)*** is observed when we investigated the reaction behavior of dihydopyran **8**, which is derived from γ-deoxygenation of the corresponding Achmatowicz adduct **2w**.^48^ We expected cyclization of **8** to afford **11**, following the typical reactivity pattern for alkynyl *halo*-Prins reactions.^38^ However, instead of **11**, cyclic acetal **9** is formed in 36% yield. Additional exposure of **9** to the reaction conditions produces tetracycle **10**, complete with bridging oxygen, in 46% yield (Scheme 6A). The structure of compound **10** is definitely confirmed by X-ray crystallography (CCDC: 2388459).

The mild conditions enable execution of the two steps in one pot, delivering tetracycle **10** in 29% yield (Scheme 6B). A plausible mechanism for the formation of compound **10** is proposed. The ketone, rather than the oxocarbenium derived from the enol ether, engages as the electrophile in the *halo*-alkynyl Prins cyclization, to generate putative intermediate **VII**. Then, transannular acetal formation affords **9**, which can open to provide oxocarbenium intermediate **IX**. Finally, Friedel–Crafts cyclization delivers the tetracycle with bridging oxygen motif **10** (Scheme 6B). This cyclization reaction, which occurs in one pot, generates two C/C bonds, two stereogenic sp^3^ centers, and one C–X bond.

**Scheme 5:**
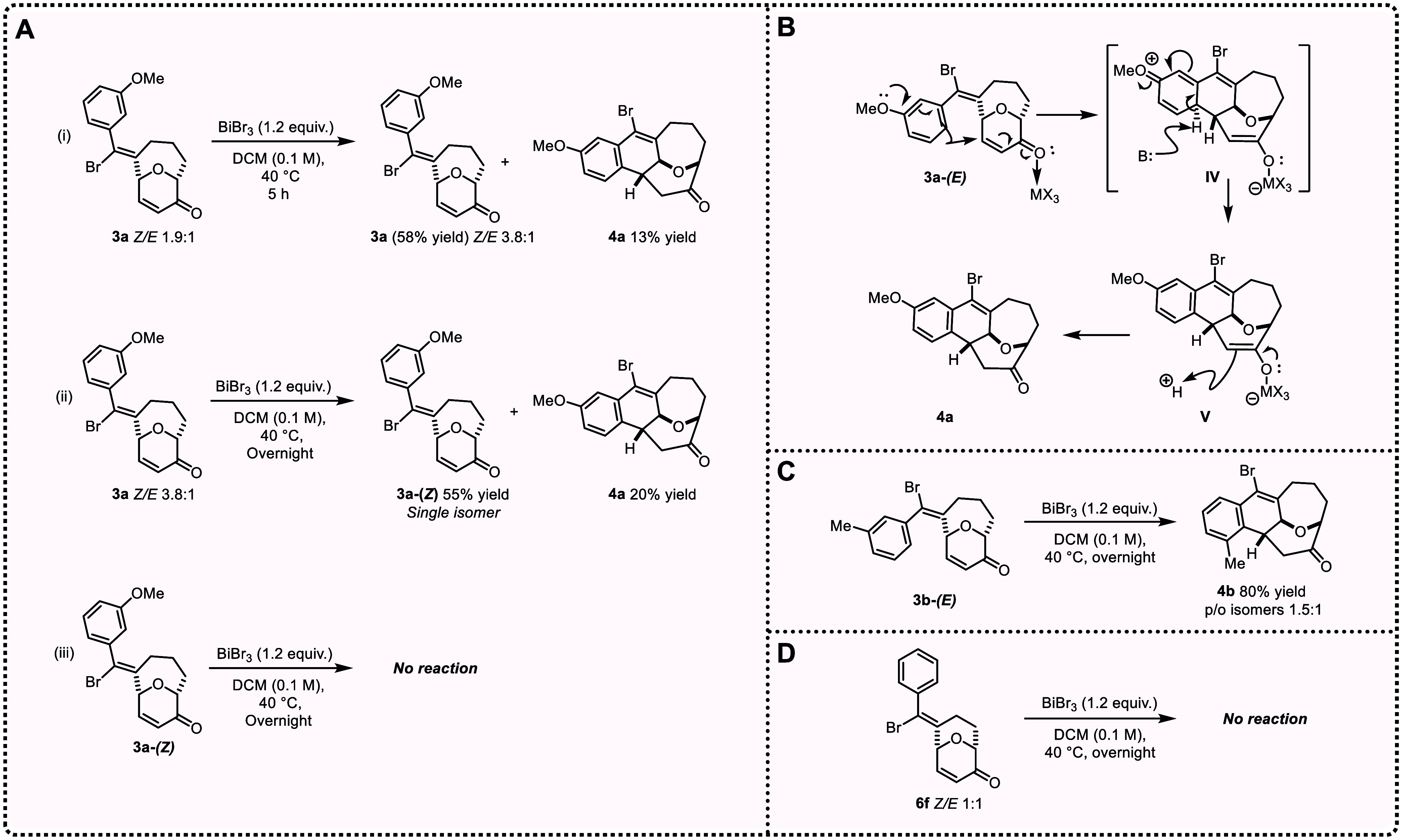
Insight into the Formation of **4a** ^a^

a [A] Understanding the formation of tetracycle **4a**. (i) 0.06 mmol of **3a** (*Z*/*E* 1.9:1); (ii) 0.03 mmol of **3a** (*Z*/*E* 3.8:1); (iii) 0.1 mmol of **3a-(** ***Z*** **)**. [B] Proposed mechanism for the formation of **4a** from **3a-(** ***E*** **)**. [C] 0.08 mmol of **3b-(** ***E*** **)**. [D] 0.2 mmol of **6f** (*Z*/*E* 1:1).

**Scheme 6:**
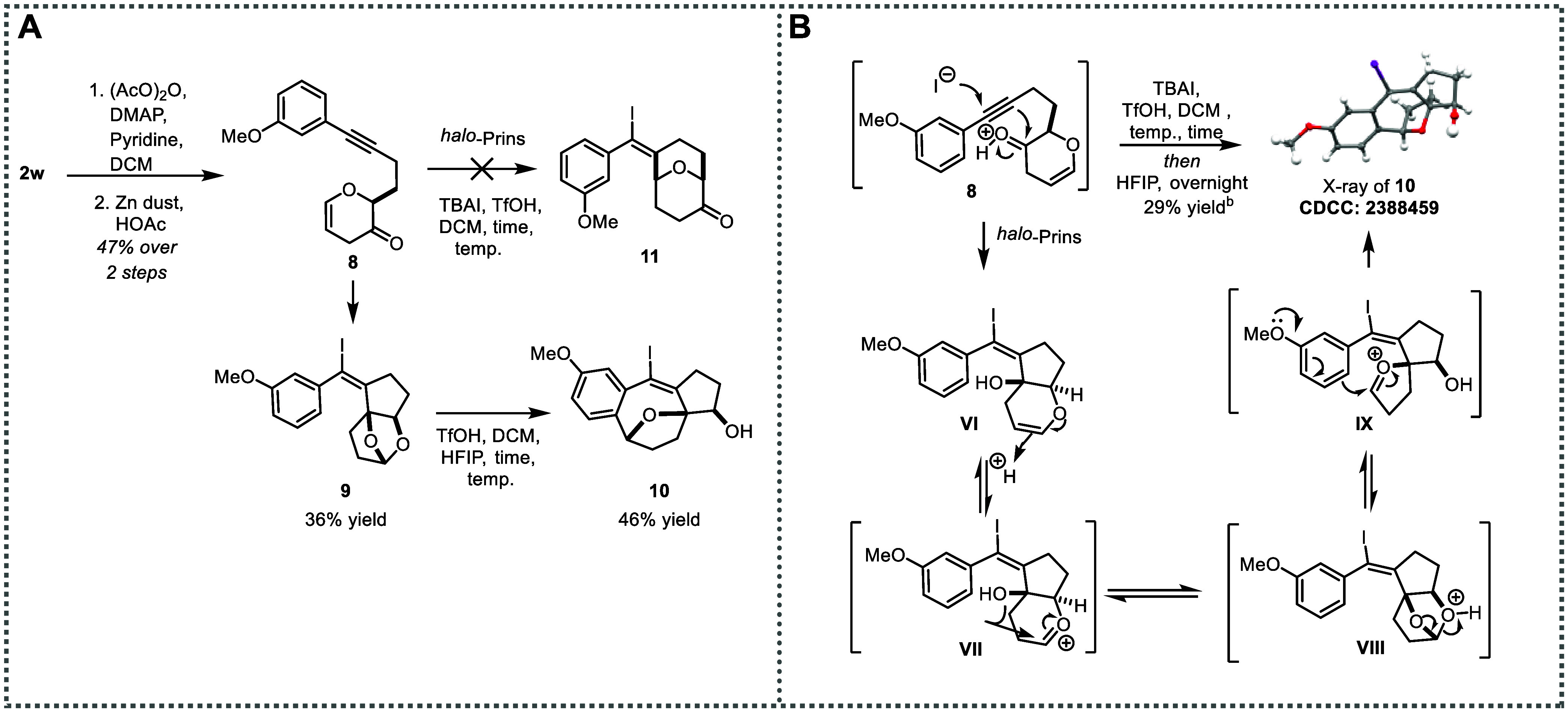
Unexpected Formation of Tetracyclic Structure **10** ^a^

a Anhydrous solvents, isolated yields.
b Recovered a small amount of **8**.
c [A] Unexpected behavior of dihydropyran **8**; Synthesis of **8**: **2w** (1 equiv), DCM (0.12 M), DMAP (41 mol %), pyridine (2.4 equiv) (AcO)_2_O (2.4 equiv), rt, 1 h; then zinc dust (5 equiv), HOAc (0.1 M), rt, 1 h. Synthesis of **10**: *halo*-Prins (step 1): **8** (0.2 mmol), TBAI (2 equiv), TfOH (1 equiv), DCM (0.1 M), 0 °C, 10 min. Ionization (step 2): **9** (0.1 mmol), TfOH (20 mol %), DCM: HFIP (5:1, 0.1 M), 0 °C, 10 min. [B] One-pot result and proposed mechanism: **8** (0.2 mmol), TBAI (1.2 equiv), TfOH (1.2 equiv), DCM (0.1 M), 0 °C, 10 min, then, HFIP (0.5 M), overnight.

## Conclusions

In conclusion, we have developed a novel method for synthesizing ring systems containing a bridging oxygen using alkynyl *halo*-Prins cyclization. Employing InBr_3_ as both a catalyst and halide source, we can build [4.3.1] and [3.3.1] oxygen heterocycles from Achmatowicz rearrangement products. This approach provides rapid access to a diverse collection of highly functionalized scaffolds featuring a bridging oxygen motif. To highlight the versatility of these rigid bicyclic scaffolds, we conducted a comprehensive product transformation study, demonstrating how each carbon atom can be selectively functionalized. We also report the formation of two novel tetracyclic heterocycles containing a bridging oxygen and offer a mechanistic rationale for the cascade processes responsible. Ongoing work in our laboratory is focused on the development of new cascade processes initiated by transannular alkynyl *halo*-Prins cyclizations.

## Experimental Section

### General Remarks

All vacuum/argon flushes and flame-drying methods were conducted using a Schlenk line, utilizing septa and needles (without Schlenk or multineck flasks unless explicitly mentioned). Reagents were used as provided by commercial suppliers without additional purification, unless otherwise specified. Tetrahydrofuran (THF, stabilized with BHT), methylene chloride (DCM), methanol, and dimethylformamide (DMF) were sourced from Fisher and dried by adding vacuum/flame-dried 4 Å molecular sieves (typically a ∼2 cm layer of spherical sieves per solvent bottle, for at least 3 days). The solvents were handled in an air atmosphere. Molecular sieves (3 and 5 Å) were purchased from Aldrich, stored in an oven at 120 °C before use, and further dried with a Tirrill burner under vacuum, followed by cooling under vacuum and exposure to argon. Screw-top tubes and flasks with thick glass walls were used to withstand higher pressures compared with regular round-bottom flasks (refer to CG-1880 from Chemglass). Celite 545 was obtained from the EMD. ACS-grade hexanes, toluene, ethyl acetate, and DCM were used for column chromatography. Thin-layer chromatography (TLC) was performed on precoated silica gel 60 F254 glass plates (from EMD), with visualization via UV light, followed by staining with *p*-anisaldehyde or Seebach’s solution and heating (using a hot plate until decoloration). Column chromatography was conducted by using EM Science silica gel with a 60 Å pore size and a 230–400 mesh range. Preparative thin-layer chromatography (prep-TLC) utilized glass-supported, precoated silica gel 60 F254 plates from EMD, which were also trimmed for use in regular TLC analysis. Deuterated solvents were obtained from Cambridge Isotope Laboratories. An oil bath was used as the heating source for reactions that require heating.

^1^H NMR spectra were measured at ambient temperature (unless otherwise noted) using either a Bruker Avance spectrometer (400 and 500 MHz) or a JEOL spectrometer (400 and 500 MHz), with data processed in MestReNova version 14.2.1–27684. Chemical shifts are expressed in parts per million (ppm), referenced to the residual proton signal of the solvent (δ = 7.26 for CHCl_3,_ or by using the built-in reference values in MestReNova for other solvents). NMR results are provided as follows: chemical shift, multiplicity (s = singlet, d = doublet, t = triplet, q = quartet, p = pentet/quintet, m = multiplet, and combinations like dt = doublet of triplets), coupling constants (*J*) in Hz, and integration. For samples containing two or more diastereomers or *Z*/*E*-isomers, chemical shifts of both isomers are presented, and the best-resolved peaks without overlap were used to calculate the diastereomeric ratio (dr) or *Z*/*E* ratio.

The ^13^C­{^1^H} NMR spectra were obtained at room temperature, unless otherwise specified, using a 126 or 101 MHz Bruker Avance and JEOL spectrometer with proton decoupling. Chemical shifts are reported in parts per million (ppm) and are referenced to the carbon resonance of the solvent (δ = 77.0 for CHCl_3_, or based on the built-in reference values for other solvents in MestReNova version 14.2.1–27684). When two or more mixtures of isomers (dr or *E*/*Z*) are present, the chemical shifts for all of the isomers are provided together.

High-resolution mass spectra (HRMS) were obtained at the University of Rochester Mass Spectrometry Resource Laboratory using a Thermo QExactive Plus hybrid quadrupole-Orbitrap mass spectrometer with scans acquired on the Orbitrap. X-ray crystallographic data were collected by Dr. William W. Brennessel at the University of Rochester’s X-ray Crystallographic Facility (Rochester, NY 14627, USA) using a Rigaku XtaLab Synergy-S Dualflex diffractometer equipped with a HyPix-6000HE HPC area detector at 100 K.

### General Procedure for Alkynyl *Halo-*Prins Cyclization

To a scintillation vial or round-bottom flask containing Achmatowicz adduct **2** (0.2 mmol, 1 equiv) under an argon atmosphere, was added InBr_3_ (50 mol %), NaClO_4_ (1 equiv), and anhydrous CHCl_3_ (0.1 M). The reaction mixture was stirred at 50 °C under an argon atmosphere for 1 h. Upon completion according to TLC, the reaction was cooled to room temperature; the precipitate was filtered off through a Celite pad, and the filtrate was concentrated. The crude was dissolved in a water-miscible organic solvent such as THF and added to a freshly prepared sat. NaHSO_3_, shake vigorously for a minute. Water was then added, and EA was used for extraction to obtain two separate layers (aqueous and organic layers). The organic layer was dried over MgSO_4_, filtered through a Celite pad, and concentrated. The crude residue was further purified by silica gel column chromatography using a mixture of hexanes (Hex) and ethyl acetate (EA) as the eluent to furnish **3, 5, or 6**, depending on the starting material **2**.

#### Purification by Silica Gel Column Chromatography (10% EA in Hex) Affords **3a** in 59% Yield (*Z*/*E* 2.5:1), 41 mg as a Yellow Oil

^1^H NMR (400 MHz, CDCl_3_) δ 7.52 (dd, *J* = 10.6, 4.3 Hz, 1H), 7.33–7.24 (m, 1H), 6.86–6.82 (m, 2H), 6.79–6.78 (m, 1H), 6.30–6.26 (m, 1H), 6.16 (dd, *J* = 10.4, 2.2 Hz, 1H, *minor isomer*), 5.75–5.74 (m, 1H), 5.24 (dd, *J* = 4.3, 2.2 Hz, 1H, *minor isomer*), 4.81 (d, *J* = 4 Hz, 1H, *minor isomer*), 4.61 (dd, *J* = 6.7, 2.0 Hz, 1H), 4.48 (d, *J* = 3.3 Hz, 1H, *minor isomer*), 3.81 (s, 3H), 2.72–2.65 (m, 1H), 2.45–2.37 (m, 1H), 2.16–2.01 (m, 3H), 1.62–1.55 (m, 1H). ^13^C­{^1^H} NMR (101 MHz, CDCl_3_) δ: 196.5, 159.3, 148.3, 141.2, 140.2, 129.4, 126.2, 121.3, 116.3, 114.6, 114.0, 79.2, 76.3, 55.3, 36.9, 34.3, 22.0. HRMS (ESI-Orbitrap) *m*/*z*: [M + H]^+^ Calcd for C_17_H_18_BrO_3_^+^ 349.0434; Found: 349.0428.

#### Purification by Silica Gel Column Chromatography (10% EA in Hex) Affords **3b-**(*Z*-Isomer) in 42% Yield, 28 mg as a Colorless Oil

^1^H NMR (400 MHz, CHLOROFORM-*D*) δ 7.52 (dd, *J* = 10.6, 4.3 Hz, 1H), 7.24 (d, *J* = 6.9 Hz, 1H), 7.11–7.03 (m, 3H), 6.27 (dd, *J* = 10.5, 2.2 Hz, 1H), 5.76–5.74 (m, 1H), 4.60 (dd, *J* = 6.7, 1.9 Hz, 1H), 2.88–2.59 (m, 1H), 2.60–2.38 (m, 1H), 2.34 (s, 3H), 2.19–1.97 (m, 2H), 1.63–1.55 (m, 2H). ^13^C­{^1^H} NMR (101 MHz, CHLOROFORM-*D*) δ: 196.7, 148.6, 140.1, 140.1, 138.3, 129.7, 129.4, 128.4, 126.3, 126.2, 117.0, 79.4, 76.5, 37.1, 34.4, 22.2, 21.5. HRMS (ESI-Orbitrap) *m*/*z*: [M + H]^+^ Calcd for C_17_H_18_BrO_2_^+^ 333.0485; Found: 333.0479.

#### Purification by Silica Gel Column Chromatography (10% EA in Hex) Affords **3b-**(*E*-Isomer) in 26% Yield, 17 mg as a Colorless Oil

^1^H NMR (400 MHz, CHLOROFORM-*D*) δ 7.28–7.24 (m, 1H), 7.14–7.08 (m, 3H), 6.78 (dd, *J* = 10.5, 4.3 Hz, 1H), 6.15 (dd, *J* = 10.5, 2.2 Hz, 1H), 5.21 (dd, *J* = 4.3, 2.2 Hz, 1H), 4.48 (dd, *J* = 5.7, 3.2 Hz, 1H), 3.03–2.96 (m, 1H), 2.76–2.68 (m, 1H), 2.35 (s, 3H), 2.13–2.07 (m, 2H), 1.97–1.70 (m, 2H). ^13^C­{^1^H} NMR (101 MHz, CHLOROFORM-*D*) δ: 196.7, 149.3, 140.3, 138.9, 138.5, 129.7, 129.1, 128.9, 126.7, 125.6, 122.0, 79.0, 72.9, 36.7, 35.6, 21.5, 21.4. HRMS (ESI-Orbitrap) *m*/*z*: [M + H]^+^ Calcd for C_17_H_18_BrO_2_^+^ 333.0485; Found: 333.0479.

#### Purification by Silica Gel Column Chromatography (10% EA in Hex) Affords **3c** in 57% Yield (*Z*/*E* 2.8:1), 43 mg as a Yellow Oil

^1^H NMR (400 MHz, CHLOROFORM-*D*) δ 7.54 (dd, *J* = 10.6, 4.3 Hz, 1H), 7.43–7.28 (m, 2H), 7.26–7.25 (m, 1H), 7.12–7.06 (m, 1H), 6.79 (dd, *J* = 10.5, 4.2 Hz, 1H, *minor isomer*), 6.22 (ddd, *J* = 49.1, 10.5, 2.2 Hz, 1H), 5.76 (dd, *J* = 4.4, 2.1 Hz, 1H), 4.54 (ddd, *J* = 49.2, 6.3, 2.7 Hz, 1H), 3.05–2.72 (m, 1H), 2.71–2.36 (m, 1H), 2.19–1.99 (m, 2H), 1.66–1.55 (m, 2H), 1.31 (s, 9H). ^13^C­{^1^H} NMR (101 MHz, CHLOROFORM-*D*) δ: 196.7, 152.1, 151.4, 149.3, 148.7, 139.9, 139.7, 138.4, 128.6, 128.1, 126.8, 126.3, 126.3, 126.0, 125.7, 125.6, 125.6, 122.6, 117.7, 79.4, 79.0, 76.6, 73.0, 37.1, 36.8, 35.6, 34.9, 34.9, 34.4, 31.4, 31.4, 22.2, 21.4. HRMS (ESI-Orbitrap) *m*/*z*: [M + H]^+^ Calcd for C_20_H_24_BrO_2_^+^ 375.0954; Found: 375.0945.

#### Purification by Silica Gel Column Chromatography (10% EA in Hex) Affords **3d** in 67% Yield (*Z*/*E* 2:1), 43 mg as a Yellow Oil

^1^H NMR (400 MHz, CHLOROFORM-*D*) δ 7.52 (dd, *J* = 10.6, 4.3 Hz, 1H), 7.41–7.27 (m, 4H), 7.26–7.23 (m, 1H), 6.78 (dd, *J* = 10.5, 4.2 Hz, 1H, *other isomer*), 6.27 (dd, *J* = 10.6, 2.1 Hz, 1H), 5.76 (dd, *J* = 4.5, 2.1 Hz, 1H), 4.60 (dd, *J* = 6.7, 1.9 Hz, 1H), 3.02 (dd, *J* = 6.1, 2.9 Hz, 1H), 2.47–2.33 (m, 1H), 2.25–1.96 (m, 2H), 1.93–1.72 (m, 1H), 1.65–1.53 (m, 1H). ^13^C­{^1^H} NMR (101 MHz, CHLOROFORM-*D*) δ: 196.6, 149.1, 148.5, 140.4, 140.3, 140.2, 138.8, 129.2, 129.0, 128.9, 128.6, 128.5, 126.8, 126.3, 121.8, 116.8, 79.4, 79.0, 76.5, 72.8, 37.1, 36.8, 35.6, 34.4, 22.2, 21.4. HRMS (ESI-Orbitrap) *m*/*z*: [M + H]^+^ Calcd for C_16_H_16_BrO_2_^+^ 319.0328; Found: 319.0322.

#### Purification by Silica Gel Column Chromatography (10% EA in Hex) Affords **3e** in 64% Yield (*Z*/*E* 2.3:1), 49 mg as a Colorless Oil

^1^H NMR (400 MHz, CDCl_3_) δ 7.83–7.39 (m, 5H), 6.74 (dd, *J* = 10.5, 4.2 Hz, 1H, *minor isomer*), 6.29 (dd, *J* = 10.6, 2.2 Hz, 1H), 6.19 (dd, *J* = 10.5, 2.2 Hz, 1H, *minor isomer*), 5.75 (dd, *J* = 4.4, 2.1 Hz, 1H), 5.11 (dd, *J* = 4.3, 2.2 Hz, 1H, *minor isomer*), 4.61 (dd, *J* = 6.8, 1.9 Hz, 1H), 4.49 (dd, *J* = 5.6, 3.3 Hz, 1H, *minor isomer*), 3.01–2.73 (m, 1H, *minor isomer*), 2.63–2.40 (m, 2H), 2.17–2.01 (m, 2H), 1.94–1.57 (m, 2H). ^13^C­{^1^H} NMR (101 MHz, CDCl_3_) δ: 196.2, 148.0, 147.7, 141.9, 140.7, 132.4, 131.9, 129.5, 129.0, 127.1, 126.4, 126.0, 125.2, 114.5, 79.2, 78.8, 76.2, 72.5, 36.8, 36.5, 35.3, 34.2, 22.0, 21.1. HRMS (ESI-Orbitrap) *m*/*z*: [M + H]^+^ Calcd for C_17_H_15_BrF_3_O_2_^+^ 387.0202; Found: 387.0194.

#### Purification by Silica Gel Column Chromatography (10% EA in Hex) Affords **3f** in 60% Yield (*Z*/*E* 2.8:1), 42 mg as a Colorless Oil

^1^H NMR (500 MHz, CHLOROFORM-*D*) δ 7.48 (dd, *J* = 10.6, 4.3 Hz, 1H), 7.37–7.23 (m, 3H), 7.23–7.08 (m, 1H), 6.76 (dd, *J* = 10.5, 4.2 Hz, 1H, *minor isomer*), 6.27 (dd, *J* = 10.6, 2.2 Hz, 1H), 6.17 (dd, *J* = 10.5, 2.2 Hz, 1H, *minor isomer*), 5.72 (dd, *J* = 4.5, 2.2 Hz, 1H), 5.16 (dd, *J* = 4.3, 2.2 Hz, 1H, *minor isomer*), 4.59 (dd, *J* = 6.9, 1.8 Hz, 1H), 4.48 (dd, *J* = 5.9, 3.0 Hz, 1H, *minor isomer*), 2.9d–2.94 (m, 1H, *minor isomer*), 2.75–2.69 (m, 1H, *minor isomer*), 2.65–2.60 (m, 1H), 2.44–2.38 (m, 1H), 2.21–1.98 (m, 2H), 1.91–1.74 (m, 2H, *minor isomer*), 1.69–1.49 (m, 2H). ^13^C­{^1^H} NMR (126 MHz, CHLOROFORM-*D*) δ: 196.4, 148.5, 148.0, 141.7, 141.5, 134.3, 130.3, 129.8, 129.3, 129.1, 128.8, 127.4, 127.1, 126.8, 126.5, 114.8, 79.3, 78.9, 76.3, 72.7, 37.0, 36.7, 35.5, 34.4, 22.1, 21.3. HRMS (ESI-Orbitrap) *m*/*z*: [M + H]^+^ Calcd for C_16_H_15_BrClO_2_^+^ 352.9939; Found: 352.9931.

#### Purification by Silica Gel Column Chromatography (10% EA in Hex) Affords **3g** in 49% Yield (*Z*/*E* 1.3:1), 36 mg as a Colorless Oil

^1^H NMR (400 MHz, CDCl_3_) δ 7.91–7.69 (m, 4H), 7.58 (dd, *J* = 10.6, 4.3 Hz, 1H, *minor isomer*), 7.54–7.50 (m, 2H), 7.42 (dd, *J* = 8.5, 1.8 Hz, 1H), 7.37 (dd, *J* = 8.5, 1.8 Hz, 1H, *minor isomer*), 6.81 (dd, *J* = 10.5, 4.2 Hz, 1H), 6.31 (dd, *J* = 10.6, 2.1 Hz, 1H, *minor isomer*), 6.15 (dd, *J* = 10.5, 2.2 Hz, 1H), 5.82 (dd, *J* = 4.4, 2.1 Hz, 1H, *minor isomer*), 5.29 (dd, *J* = 4.3, 2.2 Hz, 1H), 4.63 (dd, *J* = 6.6, 2.0 Hz, 1H, *minor isomer*), 4.50 (dd, *J* = 5.4, 3.4 Hz, 1H), 3.10–3.04 (m, 1H), 2.86–2.64 (m, 1H), 2.51–2.44 (m, 1H, *minor isomer*), 2.17–2.04 (m, 2H), 1.98–1.74 (m, 1H), 1.68–1.51 (m, 1H). ^13^C­{^1^H} NMR (101 MHz, CHLOROFORM-*D*) δ: 196.6, 149.1, 148.5, 140.7, 139.2, 137.6, 133.1, 133.0, 132.9, 129.0, 128.4, 128.4, 128.3, 128.3, 127.9, 127.8, 127.7, 127.2, 127.0, 126.9, 126.8, 126.8, 126.4, 126.2, 121.9, 79.4, 79.0, 76.5, 72.9, 37.1, 36.8, 35.6, 34.5, 22.2, 21.4. HRMS (ESI-Orbitrap) *m*/*z*: [M + H]^+^ Calcd for C_20_H_18_BrO_2_^+^ 369.0485; Found: 369.0478.

#### Purification by Silica Gel Column Chromatography (10% EA in Hex) Affords **3h** in 49% Yield (*Z*/*E* 1.3:1), 41 mg as a Colorless Oil

^1^H NMR (400 MHz, CHLOROFORM-*D*) δ 8.79–8.60 (m, 2H), 8.14–7.94 (m, 1H), 7.91–7.83 (m, 1H), 7.79–7.56 (m, 6H), 6.90 (dd, *J* = 10.5, 4.2 Hz, 1H, *minor isomer*), 6.63–6.29 (m, 1H), 6.14 (dd, *J* = 10.4, 2.2 Hz, 1H, *minor isomer*), 6.08–5.93 (m, 1H), 5.37 (dd, *J* = 4.3, 2.3 Hz, 1H, *minor isomer*), 4.94 (dd, *J* = 4.3, 2.2 Hz, 1H, *minor isomer*), 4.65 (dd, *J* = 6.8, 1.9 Hz, 1H), 4.51–4.45 (m, 1H, *minor isomer*), 3.32–3.22 (m, 1H, *minor isomer*), 2.98–2.83 (m, 1H, *minor isomer*), 2.72–2.39 (m, 1H), 2.23–2.07 (m, 2H), 2.05–1.83 (m, 1H), 1.68–1.40 (m, 2H). ^13^C­{^1^H} NMR (101 MHz, CHLOROFORM-*D*) δ: 196.6, 196.5, 149.4, 148.9, 148.5, 148.1, 142.6, 141.4, 135.7, 131.3, 131.2, 130.9, 130.6, 130.6, 129.5, 129.3, 129.1, 129.1, 128.5, 128.1, 127.9, 127.7, 127.4, 127.3, 127.3, 127.2, 127.2, 127.2, 126.7, 126.5, 125.9, 125.9, 125.7, 125.4, 123.5, 123.3, 122.7, 119.8, 114.4, 79.5, 79.1, 79.0, 76.3, 76.2, 73.6, 73.1, 37.1, 37.1, 36.8, 36.7, 36.1, 35.8, 34.8, 34.0, 22.1, 22.0, 21.6. HRMS (ESI-Orbitrap) *m*/*z*: [M + H]^+^ Calcd for C_24_H_20_BrO_2_^+^ 419.0641; Found: 419.0635.

#### Purification by Silica Gel Column Chromatography (10% EA in Hex) Affords **3i** in 77% Yield (*Z*/*E* 1.7:1), 56 mg as a Colorless Oil

^1^H NMR (400 MHz, CDCl_3_) δ 7.46 (dd, *J* = 10.4, 4.3 Hz, 1H), 7.34–7.19 (m, 1H), 6.94–6.70 (m, 3H), 6.22 (dd, *J* = 10.4, 2.2 Hz, 1H), 6.11 (dd, *J* = 10.4, 2.3 Hz, 1H, *minor isomer*), 5.84–5.72 (m, 1H), 5.22 (dd, *J* = 4.2, 2.3 Hz, 1H, *minor isomer*), 3.80 (s, 3H), 3.04–2.93 (m, 1H, *minor isomer*), 2.80–2.57 (m, 1H), 2.46–2.34 (m, 1H), 2.22–2.02 (m, 1H), 1.93–1.67 (m, 2H), 1.63–1.52 (m, 1H), 1.45 (s, 3H), 1.38 (s, 3H, *minor isomer*). ^13^C­{^1^H} NMR (101 MHz, CDCl_3_) δ: 198.6, 159.3, 148.9, 148.0, 141.2, 140.3, 129.9, 129.4, 126.3, 125.8, 121.4, 120.7, 115.9, 114.6, 114.3, 114.0, 82.5, 81.9, 72.8, 55.3, 44.4, 42.9, 36.7, 34.2, 26.6, 25.7, 22.2, 21.4. HRMS (ESI-Orbitrap) *m*/*z*: [M + H]^+^ Calcd for C_18_H_20_BrO_3_^+^ 363.0591; Found: 363.0582.

#### Purification by Silica Gel Column Chromatography (10% EA in Hex) with Little Modification (Reaction at Room Temperature) Affords **3j** in 33% Yield (*Z*/*E* 2.5:1), 23 mg as a Colorless Oil

^1^H NMR (400 MHz, CDCl_3_) δ 7.44–7.27 (m, 2H), 6.99–6.83 (m, 2H), 6.25–6.13 (m, 1H), 5.86–5.73 (m, 1H), 4.51 (dd, *J* = 7.5, 3.3 Hz, 1H), 4.23 (dd, *J* = 8.1, 3.9 Hz, 1H, *minor isomer*), 3.82 (s, 1H), 2.97–2.83 (m, 2H), 2.12–2.07 (m, 1H), 1.99–1.80 (m, 3H). ^13^C­{^1^H} NMR (101 MHz, CDCl_3_) δ: 195.3, 159.6, 142.5, 133.0, 130.6, 128.3, 122.1, 117.2, 113.5, 91.4, 89.5, 89.3, 78.8, 74.1, 55.2, 40.9, 28.4, 23.5, 23.0. HRMS (ESI-Orbitrap) *m*/*z*: [M + H]^+^ Calcd for C_17_H_18_BrO_3_^+^ 349.0434; Found: 349.0427.

#### Purification by Silica Gel Column Chromatography (10% EA in Hex) Affords **3k** in 56% Yield (*Z*/*E* 2:1), 39 mg as a White Solid

^1^H NMR (400 MHz, CHLOROFORM-*D*) δ 7.49 (dd, *J* = 10.6, 4.3 Hz, 1H), 7.34 (dd, *J* = 15.5, 8.2 Hz, 2H), 7.18 (d, *J* = 8.2 Hz, 2H), 6.74 (dd, *J* = 10.5, 4.3 Hz, 1H, *minor isomer*), 6.27 (dd, *J* = 10.5, 2.1 Hz, 1H), 6.16 (dd, *J* = 10.4, 2.2 Hz, 1H, *minor isomer*), 5.72 (d, *J* = 3.3 Hz, 1H), 5.35–5.09 (m, 1H, *minor isomer*), 4.59 (dd, *J* = 6.9, 1.9 Hz, 1H), 4.47 (dd, *J* = 5.7, 3.1 Hz, 1H, *minor isomer*), 3.08–2.79 (m, 1H, *minor isomer*), 2.79–2.68 (m, 1H, *minor isomer*), 2.67–2.54 (m, 1H), 2.43–2.36 (m, 1H), 2.28–1.96 (m, 2H), 1.93–1.69 (m, 1H), 1.62–1.49 (m, 1H). ^13^C­{^1^H} NMR (101 MHz, CHLOROFORM-*D*) δ: 196.5, 148.5, 148.1, 141.2, 139.7, 138.7, 138.5, 135.0, 134.6, 130.6, 130.1, 129.3, 128.8, 127.0, 126.4, 120.5, 115.3, 79.3, 79.0, 76.4, 72.7, 37.0, 36.7, 35.5, 34.4, 22.1, 21.3. m.p = 118–120 °C. HRMS (ESI-Orbitrap) *m*/*z*: [M + H]^+^ Calcd for C_16_H_15_BrClO_2_^+^ 352.9938; Found: 352.9935.

Reaction was performed at room temperature instead. Purification by silica gel column chromatography (10% EA in Hex) affords **3l**-(*Z*-*isomer*) in 15% yield, 11 mg as a colorless oil ^1^H NMR (500 MHz, CHLOROFORM-*D*) δ 7.60 (d, *J* = 2.1 Hz, 1H), 7.50 (dd, *J* = 10.6, 4.3 Hz, 1H), 7.45–7.39 (m, 2H), 7.14 (dd, *J* = 8.5, 1.8 Hz, 1H), 6.71 (s, 1H), 6.23 (dd, *J* = 10.6, 2.1 Hz, 1H), 5.73 (t, *J* = 3.1 Hz, 1H), 4.56 (d, *J* = 6.7 Hz, 1H), 2.64–2.59 (m, 1H), 2.41–2.34 (m, 1H), 2.13–1.90 (m, 3H), 1.52 (t, *J* = 4.8 Hz, 1H). ^13^C­{^1^H} NMR (126 MHz, CHLOROFORM-*D*) δ 196.7, 154.5, 148.6, 146.1, 140.3, 135.0, 127.5, 126.3, 125.6, 122.0, 117.2, 111.5, 106.8, 79.3, 76.5, 37.0, 34.5, 22.1. HRMS (ESI-Orbitrap) *m*/*z*: [M + H]^+^ Calcd for C_18_H_16_BrO_3_^+^ 359.0278; Found: 359.0270.

#### Purification by Silica Gel Column Chromatography (10% EA in Hex) Affords **3l**-(*E*-Isomer) in 15% Yield, 11 mg as a Colorless Oil

^1^H NMR (400 MHz, CHLOROFORM-*D*) δ 7.67 (d, *J* = 2.2 Hz, 1H), 7.59–7.47 (m, 2H), 7.29–7.20 (m, 1H), 6.84–6.74 (m, 2H), 6.14 (dd, *J* = 10.5, 2.2 Hz, 1H), 5.23 (dd, *J* = 4.2, 2.2 Hz, 1H), 4.48 (dd, *J* = 5.5, 3.3 Hz, 1H), 3.06–3.00 (m, 1H), 2.80–2.72 (m, 1H), 2.12–2.09 (m, 2H), 1.96–1.73 (m, 2H). ^13^C­{^1^H} NMR (101 MHz, CHLOROFORM-*D*) δ 196.6, 154.7, 149.2, 146.3, 139.1, 135.2, 127.9, 126.7, 125.0, 122.3, 121.5, 112.1, 106.8, 79.0, 72.9, 36.8, 35.6, 21.4. HRMS (ESI-Orbitrap) *m*/*z*: [M + H]^+^ Calcd for C_18_H_16_BrO_3_ 359.0278; Found: 359.0270.

#### Purification by Silica Gel Column Chromatography (10% EA in Hex) Affords **3m** in 47% Yield (*Z*/*E* 1:1), 35 mg as a Colorless Oil

^1^H NMR (400 MHz, CHLOROFORM-*D*) δ 7.89 (d, *J* = 8.3 Hz, 1H), 7.76 (d, *J* = 1.7 Hz, 1H), 7.57–7.49 (m, 1H), 7.34–7.22 (m, 2H), 6.79 (dd, *J* = 10.5, 4.2 Hz, 1H), 6.29 (dd, *J* = 10.5, 2.2 Hz, 1H, *other isomer*), 6.14 (dd, *J* = 10.5, 2.2 Hz, 1H), 5.79 (dd, *J* = 4.5, 2.1 Hz, 1H, *other isomer*), 5.25 (dd, *J* = 4.3, 2.2 Hz, 1H), 4.61 (dd, *J* = 6.7, 2.0 Hz, 1H, *other isomer*), 4.49 (dd, *J* = 5.5, 3.3 Hz, 1H), 3.07–3.01 (m, 1H), 2.83–2.64 (m, 1H), 2.50–2.38 (m, 1H, *other isomer*), 2.22–2.04 (m, 2H), 2.03–1.82 (m, 1H), 1.64–1.53 (m, 1H). ^13^C­{^1^H} NMR (101 MHz, CHLOROFORM-*D*) δ 196.6, 149.1, 148.5, 140.2, 139.8, 139.2, 136.4, 128.1, 127.8, 126.7, 126.3, 125.3, 124.6, 124.2, 124.0, 123.5, 123.2, 122.6, 122.0, 79.4, 79.0, 76.5, 72.9, 37.1, 36.8, 35.6, 34.5, 22.2, 21.4. HRMS (ESI-Orbitrap) *m*/*z*: [M + H]^+^ Calcd for C_18_H_16_BrO_2_S^+^ 375.0049; Found: 375.0043.

#### Purification by Silica Gel Column Chromatography (10% EA in Hex) Affords **3n** in 41% Yield (*Z*/*E* 1:1), 42 mg as a Colorless Oil

^1^H NMR (500 MHz, CHLOROFORM-*D*) δ 7.96 (dd, *J* = 17.8, 8.6 Hz, 1H), 7.78 (d, *J* = 8.4 Hz, 2H), 7.60 (dd, *J* = 9.5, 3.7 Hz, 1H), 7.52 (dd, *J* = 10.5, 4.3 Hz, 1H, *other isomer*), 7.44 (dd, *J* = 27.9, 1.7 Hz, 1H), 7.32–7.23 (m, 3H), 7.19 (dd, *J* = 8.6, 1.8 Hz, 1H), 6.75 (dd, *J* = 10.5, 4.2 Hz, 1H), 6.63 (dd, *J* = 5.3, 3.7 Hz, 1H), 6.27 (dd, *J* = 10.6, 2.2 Hz, 1H, *other isomer*), 6.13 (dd, *J* = 10.5, 2.2 Hz, 1H), 5.75 (dd, *J* = 4.4, 2.1 Hz, 1H, *other isomer*), 5.16 (dd, *J* = 4.2, 2.2 Hz, 1H), 4.60 (dd, *J* = 6.9, 1.8 Hz, 1H, *other isomer*), 4.46 (dd, *J* = 5.8, 3.1 Hz, 1H), 2.02–3.96 (m, 1H), 2.77–2.71 (m, 1H), 2.65–2.59 (m, 1H, *other isomer*), 2.35 (s, 3H), 2.14–2.00 (m, 2H), 1.88–1.75 (m, 1H), 1.57–1.53 (m, 1H). ^13^C­{^1^H} NMR (126 MHz, CHLOROFORM-*D*) δ 196.6, 196.6, 149.1, 148.5, 145.4, 145.4, 140.5, 139.1, 135.4, 135.3, 135.2, 134.4, 134.3, 130.9, 130.6, 130.2, 130.1, 127.5, 127.3, 127.0, 126.8, 126.3, 125.8, 125.1, 122.2, 122.1, 121.6, 116.9, 114.0, 113.5, 108.9, 108.8, 79.4, 78.9, 76.5, 72.8, 37.1, 36.8, 35.6, 34.5, 22.2, 21.7, 21.3. HRMS (ESI-Orbitrap) *m*/*z*: [M + H]^+^ Calcd for C_25_H_23_BrNO_4_S^+^ 512.0526; Found: 512.0518.

#### Purification by Silica Gel Column Chromatography (10% EA in Hex) Affords **3o** in 46% Yield (*Z*/*E* 1:1), 52 mg as a Colorless Oil

^1^H NMR (400 MHz, CHLOROFORM-*D*) δ 8.33 (dd, *J* = 8.4, 2.7 Hz, 1H), 8.28 (s, 1H), 8.22 (s, 1H, *other isomer*), 7.93–7.82 (m, 2H), 7.69 (d, *J* = 8.0 Hz, 2H), 7.58 (dd, *J* = 10.6, 4.3 Hz, 1H, *other isomer*), 7.54–7.48 (m, 1H), 7.40–7.34 (m, 1H), 7.35–7.25 (m, 1H), 7.10 (dd, *J* = 8.4, 3.2 Hz, 2H), 6.85 (dd, *J* = 10.5, 4.3 Hz, 1H), 6.33 (dd, *J* = 10.5, 2.1 Hz, 1H, *other isomer*), 6.18 (dd, *J* = 10.5, 2.2 Hz, 1H), 5.82 (dd, *J* = 4.5, 2.1 Hz, 1H, *other isomer*), 5.22 (dd, *J* = 4.3, 2.2 Hz, 1H), 4.64 (dd, *J* = 6.5, 2.0 Hz, 1H, *other isomer*), 4.52 (t, *J* = 4.4 Hz, 1H), 3.09–3.00 (m, 1H), 2.86–2.75 (m, 1H), 2.73–2.56 (m, 1H, *other isomer*), 2.53–2.44 (m, 1H, *other isomer*), 2.26 (s, 3H, *other isomer*), 2.25 (s, 3H), 2.21–2.02 (m, 2H), 2.01–1.76 (m, 1H), 1.73–1.54 (m, 1H). ^13^C­{^1^H} NMR (101 MHz, CHLOROFORM-*D*) δ 196.6, 196.6, 149.0, 148.4, 145.4, 145.3, 140.9, 139.5, 139.2, 139.1, 138.1, 138.0, 134.7, 129.9, 129.8, 128.2, 128.1, 126.9, 126.8, 126.6, 126.6, 126.5, 125.7, 125.1, 124.5, 124.3, 124.3, 121.9, 120.6, 120.4, 120.3, 120.0, 116.7, 116.1, 115.5, 115.4, 115.3, 79.4, 79.0, 76.5, 73.0, 37.1, 36.9, 35.6, 34.6, 22.2, 21.6, 21.4. HRMS (ESI-Orbitrap) *m*/*z*: [M + H]^+^ Calcd for C_29_H_25_BrNO_4_S^+^ 562.0682; Found: 562.0674.

Reaction was performed at room temperature instead. Purification by silica gel column chromatography (10% EA in Hex) affords **3p** in 10% yield (7 mg) as a colorless oil ^1^H NMR (400 MHz, CHLOROFORM-*D*) δ 7.49 (dd, *J* = 10.5, 4.3 Hz, 1H), 6.82–6.62 (m, 3H), 6.27 (dd, *J* = 10.5, 2.1 Hz, 1H), 5.98 (s, 2H), 5.72 (t, *J* = 3.2 Hz, 1H), 4.59 (d, *J* = 6.6 Hz, 1H), 2.75–2.64 (m, 1H), 2.45–2.35 (m, 1H), 2.17–1.98 (m, 2H), 1.62–1.57 (m, 2H). ^13^C­{^1^H} NMR (101 MHz, CHLOROFORM-*D*) δ: 196.6, 148.5, 147.8, 147.6, 140.4, 133.9, 126.3, 123.2, 116.7, 109.7, 108.2, 101.5, 79.3, 76.5, 37.1, 34.5, 22.2. HRMS (ESI-Orbitrap) *m*/*z*: [M + H]^+^ Calcd for C_17_H_16_BrO_4_^+^ 363.0227; Found: 363.0218.

#### Purification by Silica Gel Column Chromatography (10% EA in Hex) Affords **5a** in 30% Yield (*Z*/*E* 3:1), 30 mg as a Colorless Oil

^1^H NMR (500 MHz, CHLOROFORM-*D*) δ 7.66 (d, *J* = 8.3 Hz, 1H), 7.43 (dd, *J* = 10.6, 4.1 Hz, 1H), 7.38–7.25 (m, 3H), 7.17 (d, *J* = 8.0 Hz, 1H), 7.05–6.79 (m, 3H), 6.34 (dd, *J* = 10.6, 2.2 Hz, 1H), 6.28 (dd, *J* = 10.5, 2.3 Hz, 1H, *minor isomer*), 5.75 (dd, *J* = 4.2, 2.3 Hz, 1H), 5.20 (dd, *J* = 4.0, 2.3 Hz, 1H, *minor isomer*), 4.99 (dd, *J* = 17.4, 1.6 Hz, 1H, *minor isomer*), 4.46 (d, *J* = 4.5 Hz, 1H), 4.41 (dd, *J* = 16.3, 1.4 Hz, 1H), 4.34 (dd, *J* = 3.8, 1.5 Hz, 1H, *minor isomer*), 4.22 (t, *J* = 1.6 Hz, 1H, *minor isomer*), 4.20–4.15 (m, 2H), 3.85 (s, 3H), 3.81 (s, 3H, *minor isomer*), 3.71 (d, *J* = 17.4 Hz, 1H, *minor isomer*), 3.49 (d, *J* = 16.1 Hz, 1H), 3.12 (dd, *J* = 14.1, 4.4 Hz, 1H), 2.43 (s, 3H), 2.36 (s, 3H, *minor isomer*). ^13^C­{^1^H} NMR (126 MHz, CHLOROFORM-*D*) δ: 193.3, 193.2, 159.8, 159.6, 147.3, 147.2, 144.0, 143.8, 140.1, 140.0, 137.4, 135.9, 135.1, 130.2, 130.1, 129.9, 129.8, 127.8, 127.0, 126.8, 126.6, 122.0, 121.1, 120.7, 118.7, 115.4, 115.1, 114.1, 113.9, 78.8, 78.2, 77.4, 77.1, 76.9, 76.0, 72.7, 56.1, 55.7, 55.5, 55.5, 55.1, 52.2, 21.6. HRMS (ESI-Orbitrap) *m*/*z*: [M + H]^+^ Calcd for C_23_H_23_BrNO_5_S^+^ 504.0475; Found: 504.0471.

#### Purification by Silica Gel Column Chromatography (10% EA in Hex) Affords **5b** in 51% Yield (*Z*/*E* 3.4:1), 48 mg as a Colorless Oil

^1^H NMR (500 MHz, CDCl_3_) δ 7.72 (dd, *J* = 63.1, 8.0 Hz, 1H), 7.47–7.35 (m, 5H), 7.35–7.28 (m, 3H), 7.15 (d, *J* = 7.9 Hz, 1H), 6.82 (dd, *J* = 10.5, 4.0 Hz, 1H, *minor isomer*), 6.33 (dd, *J* = 10.6, 2.3 Hz, 1H), 6.28 (dd, *J* = 10.5, 2.3 Hz, 1H, *minor isomer*), 5.87–5.66 (m, 1H), 5.23–5.11 (m, 1H, *minor isomer*), 4.99 (dd, *J* = 17.3, 1.6 Hz, 1H, *minor isomer*), 4.45 (d, *J* = 4.6 Hz, 1H), 4.39–4.31 (m, 1H), 4.19 (dd, *J* = 14.4, 9.3 Hz, 1H), 3.72 (d, *J* = 17.4 Hz, 1H, *minor isomer*), 3.49 (d, *J* = 16.1 Hz, 1H), 3.36–2.98 (m, 1H), 2.43 (s, 3H, *minor isomer*), 2.35 (s, 3H). ^13^C­{^1^H} NMR (101 MHz, CHLOROFORM-*D*) δ: 193.3, 147.2, 143.7, 138.9, 137.4, 135.9, 130.1, 129.8, 129.5, 129.2, 129.1, 129.0, 128.7, 128.6, 127.9, 127.0, 126.8, 126.6, 119.0, 78.8, 78.2, 76.1, 72.7, 56.1, 55.1, 52.2, 30.4, 21.6. HRMS (ESI-Orbitrap) *m*/*z*: [M + H]^+^ Calcd for C_22_H_21_BrNO_4_S^+^ 474.0369; Found: 474.0363.

#### Purification by Silica Gel Column Chromatography (10% EA in Hex) with Little Modification (Reaction at Room Temperature) Affords **6a** in 30% Yield (*Z*/*E* 1.3:1), 18 mg as a Colorless Oil

^1^H NMR (400 MHz, CHLOROFORM-*D*) δ 7.07 (dd, *J* = 10.1, 4.8 Hz, 1H, *minor isomer*), 6.92 (dd, *J* = 10.2, 4.8 Hz, 1H), 6.29 (t, *J* = 10.2 Hz, 1H), 5.72–5.64 (m, 1H), 5.58–5.53 (m, 1H, *minor isomer*)­5.21 (d, *J* = 4.7 Hz, 1H), 4.34–4.21 (m, 1H), 3.06–2.83 (m, 1H), 2.71–2.65 (m, 1H, *minor isomer*), 2.40–2.20 (m, 2H), 2.16–2.03 (m, 4H), 1.96–1.84 (m, 1H), 1.82–1.55 (m, 4H). ^13^C­{^1^H} NMR (101 MHz, CHLOROFORM-*D*) δ: 197.2, 148.6, 147.7, 137.0, 136.2, 129.4, 129.3, 128.5, 128.4, 127.6, 127.1, 126.9, 124.4, 76.3, 76.3, 73.1, 70.5, 27.7, 27.7, 27.3, 25.3, 25.2, 23.9, 22.4, 22.2, 21.8, 21.7. HRMS (ESI-Orbitrap) *m*/*z*: [M + H]^+^ Calcd for C_15_H_17_BrO_2_^+^ 309.0485; Found: 309.0478.

#### Purification by Silica Gel Column Chromatography (10% EA in Hex) Affords **6b** in 72% Yield (*Z*/*E* 1.5:1), 50 mg as a Colorless Oil

^1^H NMR (400 MHz, CDCl_3_) δ 7.40–7.22 (m, 1H), 7.17–7.11 (m, 1H), 6.95–6.77 (m, 3H), 6.30 (d, *J* = 10.1 Hz, 1H), 6.23 (d, *J* = 10.0 Hz, 1H, *minor isomer*), 5.70 (d, *J* = 4.8 Hz, 1H), 4.93 (d, *J* = 4.8 Hz, 1H, *minor isomer*), 3.82 (s, 3H, *minor isomer*), 3.80 (s, 3H), 3.10–2.98 (m, 1H, *minor isomer*), 2.54–2.34 (m, 1H), 2.31–2.17 (m, 1H), 2.07–1.61 (m, 2H), 1.37 (s, 3H), 1.35 (s, 3H, *minor isomer*). ^13^C­{^1^H} NMR (101 MHz, CDCl_3_) δ: 198.8, 159.4, 147.7, 147.0, 140.3, 130.4, 130.0, 129.7, 129.4, 128.4, 128.2, 121.1, 120.8, 118.9, 114.6, 114.4, 114.3, 78.9, 73.8, 70.4, 55.3, 34.6, 34.2, 25.2, 23.6, 23.6, 23.2. HRMS (ESI-Orbitrap) *m*/*z*: [M + H]^+^ Calcd for C_17_H_18_BrO_3_^+^ 349.0434; Found: 349.0425.

#### Purification by Silica Gel Column Chromatography (10% EA in Hex) Affords **6c** in 63% Yield (*Z*/*E* 1.4:1), 40 mg as a Colorless Oil

^1^H NMR (400 MHz, CHLOROFORM-*D*) δ 7.60–7.20 (m, 5H), 7.14 (dd, *J* = 10.1, 4.8 Hz, 1H), 6.89 (dd, *J* = 10.0, 4.8 Hz, 1H, *minor isomer*), 6.29 (d, *J* = 10.1 Hz, 1H), 6.22 (d, *J* = 10.1 Hz, 1H, *minor isomer*), 5.71 (d, *J* = 4.8 Hz, 1H), 4.90 (d, *J* = 4.7 Hz, 1H, *minor isomer*), 3.12–2.99 (m, 1H, *minor isomer*), 2.51–2.32 (m, 1H), 2.31–2.13 (m, 1H), 2.06–1.55 (m, 2H), 1.37 (s, 3H), 1.34 (s, 3H, *minor isomer*). ^13^C­{^1^H} NMR­(101 MHz, CHLOROFORM-*D*) δ: 198.9, 198.9, 147.8, 147.2, 139.2, 139.2, 130.6, 130.0, 129.1, 129.0, 128.8, 128.8, 128.6, 128.5, 128.5, 128.4, 121.7, 119.4, 79.0, 78.9, 74.0, 70.5, 34.8, 34.3, 25.4, 23.8, 23.7, 23.3. HRMS (ESI-Orbitrap) *m*/*z*: [M + H]^+^ Calcd for C_16_H_16_BrO_2_^+^ 319.0328; Found: 319.0322.

#### Purification by Silica Gel Column Chromatography (10% EA in Hex) Affords **6d** in 84% Yield (*Z*/*E* 3:1), 62 mg as a Colorless Oil

^1^H NMR (500 MHz, CDCl_3_) δ 7.72–7.41 (m, 5H), 7.16 (dd, *J* = 10.2, 4.8 Hz, 1H), 6.89 (dd, *J* = 10.2, 4.8 Hz, 1H, *minor isomer*), 6.37 (d, *J* = 10.2 Hz, 1H), 6.31 (d, *J* = 10.2 Hz, 1H, *minor isomer*), 5.69 (d, *J* = 4.8 Hz, 1H), 4.80 (d, *J* = 4.8 Hz, 1H, *minor isomer*), 4.34 (d, *J* = 5.9 Hz, 1H), 4.30 (d, *J* = 6.0 Hz, 1H, *minor isomer*), 3.07 (dd, *J* = 15.6, 5.6 Hz, 1H, *minor isomer*), 2.52–2.35 (m, 1H), 2.31–2.22 (m, 1H), 2.02–1.91 (m, 1H), 1.74 (dd, *J* = 14.2, 5.3 Hz, 1H). ^13^C­{^1^H} NMR (101 MHz, CDCl_3_) δ: 196.4, 147.1, 146.7, 139.7, 139.6, 132.4, 132.2, 131.9, 131.8, 129.4, 129.1, 128.9, 128.9, 125.7, 125.5, 119.6, 117.2, 75.9, 75.9, 73.0, 69.7, 27.4, 26.9, 24.4, 22.4. HRMS (ESI-Orbitrap) *m*/*z*: [M + H]^+^ Calcd for C_16_H_13_BrF_3_O_2_^+^ 373.0046; Found: 373.0038.

#### Purification by Silica Gel Column Chromatography (10% EA in Hex) Affords **6e** in 59% Yield (*Z*/*E* 1.4:1), 39 mg as a Colorless Oil

^1^H NMR (400 MHz, CDCl_3_) δ 7.41–7.23 (m, 1H), 7.18–7.13 (m, 1H), 7.04–6.68 (m, 3H), 6.36 (d, *J* = 10.2 Hz, 1H), 6.29 (d, *J* = 10.2 Hz, 1H, *minor isomer*), 5.69 (d, *J* = 4.8 Hz, 1H), 4.91 (d, *J* = 4.8 Hz, 1H, *minor isomer*), 4.33 (d, *J* = 5.9 Hz, 1H), 4.29 (d, *J* = 6.0 Hz, 1H, *minor isomer*), 3.82 (d, *J* = 1.1 Hz, 3H, *minor isomer*), 3.80 (d, *J* = 1.1 Hz, 3H), 3.22–2.98 (m, 1H, minor isomer), 2.55–2.37 (m, 1H), 2.33–2.09 (m, 1H), 2.05–1.89 (m, 1H), 1.77–1.62 (m, 1H). ^13^C­{^1^H} NMR (101 MHz, CDCl_3_) δ: 196.8, 159.6, 159.4, 147.8, 147.1, 140.2, 140.1, 130.6, 130.1, 129.7, 129.5, 128.7, 128.5, 121.4, 121.0, 120.8, 119.1, 114.6, 114.4, 114.3, 114.2, 76.0, 75.9, 73.2, 69.9, 55.3, 55.3, 27.5, 27.1, 24.3, 22.3. HRMS (ESI-Orbitrap) *m*/*z*: [M + H]^+^ Calcd for C_16_H_16_BrO_3_^+^ 335.0278; Found: 335.0270.

#### Purification by Silica Gel Column Chromatography (10% EA in Hex) Affords **6f** in 64% Yield (*Z*/*E* 1:1), 39 mg as a Colorless Oil

^1^H NMR (400 MHz, CDCl_3_) δ 7.46–7.25 (m, 5H), 7.18 (dd, *J* = 10.2, 4.8 Hz, 1H), 6.93 (dd, *J* = 10.1, 4.8 Hz, 1H, *minor isomer*), 6.33 (dd, *J* = 26.6, 10.2 Hz, 1H), 5.72 (d, *J* = 4.8 Hz, 1H, *minor isomer*), 4.90 (d, *J* = 4.8 Hz, 1H), 4.35 (d, *J* = 6.0 Hz, 1H), 4.30 (d, *J* = 6.1 Hz, 1H, *minor isomer*), 3.07 (dd, *J* = 15.5, 5.7 Hz, 1H, *minor isomer*), 2.52–2.39 (m, 1H), 2.33–2.10 (m, 1H), 2.06–1.90 (m, 1H), 1.73 (dd, *J* = 13.5, 5.4 Hz, 1H). ^13^C­{^1^H} NMR (101 MHz, CDCl_3_) δ: 196.7, 147.7, 147.1, 139.0, 130.6, 129.0, 128.8, 128.7, 128.6, 128.5, 128.4, 121.8, 76.0, 73.3, 69.9, 27.5, 27.1, 24.4, 22.3. HRMS (ESI-Orbitrap) *m*/*z*: [M + H]^+^ Calcd for C_15_H_13_BrO_2_^+^ 305.0172; Found: 305.0164.

### Product Transformation

#### Heck Coupling

A scintillation vial, equipped with a stir bar, was flame-dried under a vacuum and refilled with argon once it had cooled to room temperature.

To this, **6f** (0.1 mmol), styrene (0.2 mmol, 2 equiv), Pd­(OAc)_2_, PPh_3_ (10 mol %), and anhydrous DMF (0.1 M) were added under an argon atmosphere. The flask was sealed, and the reaction was stirred at 100 °C. *Quenching:* Upon completion, the reaction was cooled to room temp. and quenched with H_2_O, extracted with EA. The organic layer was washed with brine, dried over MgSO_4_, filtered through a Celite pad, and concentrated using a rotary evaporator. The crude product was purified by column chromatography (Hex/EA; 4:1) to yield 24 mg (73%) of compound **7a** as a colorless oil.

^1^H NMR (500 MHz, CHLOROFORM-*D*) δ 7.47–7.37 (m, 3H), 7.36–7.26 (m, 6H), 7.26–7.19 (m, 1H), 7.16–7.08 (m, 2H), 6.87 (dd, *J* = 10.2, 4.8 Hz, 1H, *minor isomer*), 6.36 (d, *J* = 10.2 Hz, 1H), 6.27 (d, *J* = 10.1 Hz, 1H, *minor isomer*), 6.06 (d, *J* = 15.6 Hz, 1H), 6.01 (d, *J* = 15.8 Hz, 1H, *minor isomer*), 5.77 (d, *J* = 4.8 Hz, 1H), 4.70 (d, *J* = 4.8 Hz, 1H, *minor isomer*), 4.35 (d, *J* = 5.9 Hz, 1H), 4.32 (d, *J* = 5.8 Hz, 1H, *minor isomer*), 3.14–3.02 (m, 1H, *minor isomer*), 2.40–2.24 (m, 1H), 2.22–2.05 (m, 1H), 2.02–1.94 (m, 1H), 1.78–1.73 (m, 1H). ^13^C­{^1^H} NMR (126 MHz, CHLOROFORM-*D*) δ: 197.7, 197.5, 149.1, 148.6, 138.8, 138.6, 138.2, 137.2, 137.2, 134.9, 134.7, 129.6, 129.5, 128.8, 128.8, 128.7, 128.6, 128.6, 128.5, 128.1, 128.0, 128.0, 127.7, 127.4, 126.7, 126.7, 126.2, 125.3, 76.6, 76.6, 71.2, 69.1, 28.7, 28.2, 22.8, 22.2. HRMS (ESI-Orbitrap) *m*/*z*: [M + H]^+^ Calcd for C_23_H_21_O_2_^+^ 329.1536; Found: 329.1536.

#### Suzuki Coupling

A 10 mL high-pressure flask, equipped with a stir bar, was charged with **6f** (0.2 mmol, 1 equiv), PhB­(OH)_2_ (1.5 equiv), PhMe (1.2 mL), EtOH (0.4 mL), 2 M sat. Na_2_CO_3_ (0.4 mL), and Pd­(PPh_3_)_4_ (10 mol %). The flask was sealed, and the reaction was allowed to proceed overnight at 80 °C. *Quenching:* Upon completion, the flask was cooled to room temperature, and the reaction mixture was diluted with EA before being transferred to a separatory funnel. The organic layer was washed with water, sat. NaHCO_3_, and brine. It was then dried over MgSO_4_, filtered through a Celite pad, and the solvents were removed with a rotary evaporator. The crude product was purified by flash column chromatography (Hex/EA; 4:1, same Rf as **6f**), to afford 54 mg (89% yield) of **7b** as a pale-yellow oil.

^1^H NMR (400 MHz, CDCl_3_) δ 7.39–7.28 (m, 5H), 7.28–7.21 (m, 1H), 7.19–7.11 (m, 4H), 7.08 (dd, J = 10.2, 4.8 Hz, 1H), 6.35 (d, J = 10.2 Hz, 1H), 5.07 (d, J = 4.7 Hz, 1H), 4.35 (d, J = 5.9 Hz, 1H), 2.62–2.54 (m, 1H), 2.47–2.37 (m, 1H), 2.18–2.08 (m, 1H), 1.90–1.85 (m, 1H). ^13^C­{^1^H} NMR (101 MHz, CDCl_3_) δ: 197.5, 149.2, 141.3, 140.9, 140.8, 129.2, 129.0, 128.5, 128.4, 128.2, 127.9, 127.5, 127.2, 76.5, 70.9, 29.0, 21.4. HRMS (ESI-Orbitrap) *m*/*z*: [M + H]^+^ Calcd for C_21_H_19_O_2_^+^ 303.1380; Found: 303.1380.

#### 1,4-Reduction

The reaction was carried out according to the literature procedure with little modification.^11^ **6f** (0.2 mmol, 1 equiv) was dissolved in CHCl_3_ (0.2 M) along with Ph_2_SiH_2_ (1 equiv) and wet ZnCl_2_ (36 mol %). Pd­(PPh_3_)_4_ (2 mol %) was then added, and the mixture was stirred at room temperature overnight. Upon completion, the solution was filtered through a Celite pad, and the filtrate was concentrated by using a rotary evaporator. The crude product was further purified by column chromatography. Purification by silica gel column chromatography (Hex/EA; 4:1) affords **7c-** *(Z-isomer*) in 36% yield, 22 mg as a colorless oil; ^1^H NMR (500 MHz, CHLOROFORM-*D*) δ 7.38–7.33 (m, 2H), 7.32–7.29 (m, 1H), 7.28–7.25 (m, 2H), 5.35 (dd, *J* = 9.1, 3.2 Hz, 1H), 4.21 (dd, *J* = 6.0, 2.4 Hz, 1H), 2.94–2.80 (m, 1H), 2.79–2.69 (m, 1H), 2.53–2.36 (m, 2H), 2.23–2.07 (m, 1H), 1.99–1.74 (m, 3H). ^13^C­{^1^H} NMR (126 MHz, CHLOROFORM-*D*) δ: 214.0, 139.3, 136.6, 129.0, 128.6, 128.5, 116.9, 77.2, 71.4, 35.3, 27.1, 26.2, 24.1. HRMS (ESI-Orbitrap) *m*/*z*: [M + H]^+^ Calcd for C_16_H_15_BrO_2_^+^ 307.0328; Found: 307.0328.

Purification by silica gel column chromatography (Hex/EA; 4:1) affords **7c-**(*E-isomer*) in 36% yield, 22 mg as a colorless oil; ^1^H NMR (500 MHz, CHLOROFORM-*D*) δ 7.40–7.28 (m, 3H), 7.26 (d, *J* = 1.7 Hz, 2H), 4.68–4.61 (m, 1H), 4.1–4.15 (m, 1H), 3.08–3.03 (m, 1H), 2.81–2.68 (m, 1H), 2.53–2.36 (m, 2H), 2.34–2.26 (m, 1H), 2.18–1.97 (m, 2H), 1.87–1.77 (m, 1H). ^13^C­{^1^H} NMR (126 MHz, CHLOROFORM-*D*) δ: 214.5, 139.4, 137.3, 129.0, 128.7, 128.6, 119.7, 77.3, 68.1, 35.2, 27.5, 26.6, 25.8. HRMS (ESI-Orbitrap) *m*/*z*: [M + H]^+^ Calcd for C_15_H_16_BrO_2_^+^ 307.0328; Found: 307.0328.

#### 1,2-Reduction

To a 10 mL scintillation vial equipped with a stir bar was added **6f** (0.1 mmol, 1 equiv), MeOH: THF (1:1, 0.1 M), and cooled to 0 °C in an ice bath. NaBH_4_ (4 equiv) was added portionwise to the reaction mixture and allowed to stir for 1 h at 0 °C. *Quenching*: Upon completion, Et_2_O was added to the reaction mixture and then transferred to a separatory funnel containing water. The organic layer was washed with water and brine and dried over MgSO_4_ (filtered through a Celite pad). The crude filtrate was concentrated and purified by silica gel column chromatography (Hex/EA; 2:1) to afford 16 mg (51% yield, *Z*/*E* 1:1) of **7d** as a colorless oil.

^1^H NMR (500 MHz, CHLOROFORM-*D*) δ 7.36–7.31 (m, 2H), 7.30–7.22 (m, 3H), 4.82 (d, *J* = 5.4 Hz, 1H, *Z-isomer*), 4.37 (d, *J* = 4.8 Hz, 1H, *E-isomer*), 4.07–3.85 (m, 2H), 3.00–2.58 (m, 1H), 2.48–2.23 (m, 2H), 2.19–1.86 (m, 3H), 1.83–1.57 (m, 3H). ^13^C­{^1^H} NMR (126 MHz, CHLOROFORM-*D*) δ 140.2, 140.0, 138.5, 137.9, 129.2, 128.9, 128.7, 128.5, 128.4, 128.3, 118.8, 115.4, 71.8, 69.9, 69.6, 68.7, 68.6, 68.6, 29.6, 28.7, 27.8, 27.7, 26.3, 25.5, 21.8, 20.4. HRMS (ESI-Orbitrap) *m*/*z*: [M–OH]^+^ Calcd for C_15_H_16_BrO^+^ 291.0379; Found: 291.0379.

#### Luche Reduction

A solution of **6f** (0.2 mmol, 1 equiv) in anhydrous DCM (0.1 M) and CeCl_3_/MeOH (0.4 M) was cooled to −78 °C. NaBH_4_ (1.02 equiv) was then added, and the reaction mixture was stirred for 1 h at −78 °C. *Quenching:* Upon completion, the resulting solution was diluted with Et_2_O (5 mL) and quenched with saturated NaHCO_3_. The organic layer was dried over MgSO_4_, filtered through a Celite pad, and concentrated under reduced pressure. The crude product was purified by silica gel column chromatography (Hex/EA; 2:1), yielding 43 mg (70%, *Z*/*E* 1:1) of **7e** as a white solid.

^1^H NMR (400 MHz, CDCl_3_) δ 7.52–7.08 (m, 5H), 6.19–6.12 (m, 1H, *Z-isomer*), 5.94–5.86 (m, 1H, *Z-isomer*), 5.82 (dd, *J* = 10.3, 1.8 Hz, 1H, *E-isomer*), 5.71–5.67 (m, 1H, *E-isomer*), 5.17–5.15 (m, 1H, *Z-isomer*), 4.63–4.55 (m, 1H), 4.26 (q, *J* = 6.3 Hz, 1H, *Z-isomer*), 4.18–4.14 (m, 1H, *E-isomer*), 2.91–2.59 (m, 1H), 2.48–2.16 (m, 1H), 2.11–1.69 (m, 3H). ^13^C­{^1^H} NMR (101 MHz, CDCl_3_) δ: 139.4, 137.2, 136.4, 130.0, 129.2, 129.1, 128.9, 128.5, 128.5, 128.4, 128.3, 128.2, 118.1, 115.3, 72.0, 69.6, 69.1, 68.9, 65.2, 65.0, 25.5, 24.1, 22.0. m.p = 135–137 °C. HRMS (ESI-Orbitrap) *m*/*z*: [M–OH]^+^ Calcd for C_15_H_14_BrO^+^ 289.0223; Found: 289.0223.

#### 1,2-Addition

To a flame-dried scintillation vial was added **6f** (0.2 mmol) and THF (0.1 M), and the temperature was lowered to −78 °C. Then, 3 M MeMgCl (2 equiv) was added dropwise under argon and stirred until the reaction was complete according to TLC. *Quenching:* The reaction was quenched with sat. NH_4_Cl, extracted with EA, dried over MgSO_4_, filtered through a Celite pad, and concentrated under reduced pressure. The crude product was purified by silica gel column chromatography (Hex/EA; 2:1), yielding 43 mg (67%, *Z*/*E* 1.3:1) of **7f** as a colorless oil.

^1^H NMR (500 MHz, CHLOROFORM-*D*) δ 7.35–7.17 (m, 5H), 5.98 (dd, *J* = 10.2, 3.7 Hz, 1H), 5.73 (d, *J* = 10.2 Hz, 1H, *minor isomer*), 5.67 (d, *J* = 10.2 Hz, 1H), 5.50 (dd, *J* = 10.2, 3.9 Hz, 1H, *minor isomer*), 5.06 (d, *J* = 3.8 Hz, 1H), 4.52 (d, *J* = 4.0 Hz, 1H, *minor isomer*), 3.84 (t, *J* = 6.6 Hz, 1H), 3.76 (d, *J* = 6.6 Hz, 1H, *minor isomer*), 2.79–2.49 (m, 1H), 2.40–2.22 (m, 1H), 2.21–1.92 (m, 1H), 1.81–1.76 (m, 1H), 1.41 (s, 3H), 1.31 (s, 3H, *minor isomer)*. ^13^C­{^1^H} NMR (126 MHz, CHLOROFORM-*D*) δ: 139.5, 139.4, 137.4, 136.5, 133.7, 132.7, 129.3, 129.0, 128.6, 128.6, 128.3, 128.3, 127.2, 126.6, 118.1, 115.1, 75.5, 74.8, 72.3, 69.1, 69.0, 68.5, 29.6, 28.7, 25.5, 25.1, 24.5, 23.1. HRMS (ESI-Orbitrap) *m*/*z*: [M–OH]^+^ Calcd for C_16_H_16_BrO^+^ 303.0379; Found: 303.0379.

#### 1,4-Addition

1.4 M MeLi (1.4 M; 3.5 equiv), CuI (2 equiv), and Et_2_O (1 mL) were added to a flame-dried scintillation vial and stirred for 10 min at 0 °C under argon. Then, a solution of **6f** (0.2 M) in Et_2_O (1 mL) was added dropwise, and the mixture was stirred at the same temperature until the reaction was complete (∼1 h). *Quenching:* The reaction was quenched with sat. NH_4_Cl, extracted with Et_2_O, dried over MgSO_4_, filtered through a Celite pad, and concentrated under reduced pressure. The crude product was purified by silica gel column chromatography (Hex/EA; 4:1), yielding 37 mg (58%, *Z*/*E* 1.2:1) of **7g** as a colorless oil.

^1^H NMR (500 MHz, CHLOROFORM-*D*) δ 7.57–6.94 (m, 5H), 5.00 (d, *J* = 3.4 Hz, 1H), 4.22–4.14 (m, 1H, *minor isomer*), 4.07 (dd, *J* = 18.3, 5.6 Hz, 1H), 2.97 (dd, *J* = 15.5, 5.2 Hz, 1H, *minor isomer*), 2.56 (dd, *J* = 14.8, 10.3 Hz, 1H), 2.47–2.32 (m, 2H), 2.26–1.89 (m, 3H), 1.86–1.71 (m, 1H), 1.36 (d, *J* = 6.8 Hz, 3H), 0.88 (d, *J* = 6.8 Hz, 3H, *minor isomer*). ^13^C­{^1^H} NMR (126 MHz, CHLOROFORM-*D*) δ: 215.1, 215.1, 139.5, 139.4, 137.2, 136.4, 129.1, 129.0, 128.7, 128.6, 128.5, 128.5, 119.7, 116.9, 78.7, 76.5, 76.4, 75.9, 43.9, 43.7, 33.8, 33.5, 27.7, 27.3, 25.9, 23.7, 22.3, 21.6. HRMS (ESI-Orbitrap) *m*/*z*: [M + H]^+^ Calcd for C_16_H_18_BrO_2_^+^ 321.0485; Found: 321.0484.

#### Cyclopropanation

To a flame-dried flask was added a solution of Me_3_SO^+^I^–^ (1.2 equiv) in DMSO and cooled in an ice bath to solidify. NaH (1.1 equiv) was added to the flask and allowed to warm to room temperature and stirred for 10 min. Then a solution of **6f** (0.2 mmol) in DMSO was added and the mixture was stirred at room temperature until full consumption of **6f** according to TLC (∼1 h). *Quenching:* The reaction was quenched with sat. NH_4_Cl, extracted with Et_2_O, dried over MgSO_4_, filtered through a Celite pad, and concentrated under reduced pressure. The crude product was purified by silica gel column chromatography (Hex/EA; 4:1), yielding 46 mg (72%, *Z*/*E* 1.2:1) of **7h** as a colorless oil.

^1^H NMR (500 MHz, CHLOROFORM-*D*) δ 7.46–7.17 (m, 5H), 5.26 (s, 1H), 4.53 (s, 1H, *minor isomer*), 3.99 (dd, *J* = 24.2, 4.7 Hz, 1H), 3.04–2.97 (m, 1H), 2.50–2.38 (m, 1H), 2.28–2.21 (m, 1H, *minor isomer*), 2.02–1.94 (m, 2H), 1.80–1.64 (m, 2H), 1.55–1.43 (m, 1H), 1.28–1.04 (m, 1H). ^13^C­{^1^H} NMR (126 MHz, CHLOROFORM-*D*) δ: 208.7, 208.3, 139.4, 139.3, 136.2, 135.1, 129.0, 128.8, 128.8, 128.7, 128.6, 128.5, 120.3, 117.8, 75.2, 70.8, 67.5, 29.6, 29.1, 26.4, 26.3, 26.0, 23.8, 18.8, 18.4, 10.2, 10.1. HRMS (ESI-Orbitrap) *m*/*z*: [M + H]^+^ Calcd for C_16_H_16_BrO_2_^+^ 319.0328; Found: 319.0327.

#### Cycloaddition

To a solution of **6f** (0.2 mmol) in benzene (0.05 M) was added freshly distilled cyclopentadiene (6 equiv), and the mixture was stirred at room temperature under argon for 72 h. Upon completion, the precipitate was filtered through a Celite pad, and the filtrate was concentrated under reduced pressure. The crude product was purified by silica gel column chromatography. Purification by silica gel column chromatography (Hex/EA; 4:1) affords **7i-**(*E-isomer*) in 43% yield, 32 mg as a colorless oil; ^1^H NMR (400 MHz, CHLOROFORM-*D*) δ 7.45–7.26 (m, 5H), 6.06 (dd, *J* = 5.7, 2.9 Hz, 1H), 6.02 (dd, *J* = 5.7, 2.8 Hz, 1H), 4.40 (s, 1H), 3.72 (dd, *J* = 6.0, 1.5 Hz, 1H), 3.41–3.39 (m, 1H), 3.24–3.04 (m, 1H), 3.00–2.94 (m, 1H), 2.84 (s, 1H), 2.46 (dd, *J* = 9.1, 3.4 Hz, 1H), 2.25–2.17 (m, 1H), 2.08–1.94 (m, 1H), 1.91–1.82 (m, 1H), 1.39–1.36 (m, 2H). ^13^C­{^1^H} NMR (101 MHz, CHLOROFORM-*D*) δ: 212.7, 139.5, 138.0, 135.6, 134.6, 129.0, 128.8, 128.6, 119.1, 74.9, 71.6, 50.7, 49.7, 49.2, 48.5, 42.1, 29.3, 27.4. HRMS (ESI-Orbitrap) *m*/*z*: [M + H]^+^ Calcd for C_20_H_20_BrO_2_^+^ 371.0640; Found: 371.0639.

Purification by silica gel column chromatography (Hex/EA; 4:1) affords **7i-**(*Z-isomer*) in 43% yield, 32 mg as a colorless oil; ^1^H NMR (400 MHz, CHLOROFORM-*D*) δ 7.35 (t, *J* = 7.3 Hz, 2H), 7.31–7.25 (m, 3H), 6.36 (dd, *J* = 5.8, 3.0 Hz, 1H), 6.10 (dd, *J* = 5.7, 2.9 Hz, 1H), 5.15 (s, 1H), 3.76 (d, *J* = 5.7 Hz, 1H), 3.42 (s, 1H), 3.26 (s,1H), 2.96 (dd, *J* = 9.2, 4.5 Hz, 1H), 2.56–2.41 (m, 2H), 2.07–1.95 (m, 1H), 1.85–1.75 (m, 1H), 1.67–1.61 (m, 1H), 1.46 (d, *J* = 8.5 Hz, 1H), 1.41 (d, *J* = 6.1 Hz, 1H). ^13^C­{^1^H} NMR (101 MHz, CHLOROFORM-*D*) δ: 213.1, 139.4, 137.1, 136.0, 134.3, 129.0, 128.6, 128.5, 116.6, 74.9, 74.8, 50.5, 49.9, 49.3, 49.2, 41.6, 28.8, 25.1. HRMS (ESI-Orbitrap) *m*/*z*: [M + H]^+^ Calcd for C_20_H_20_BrO_2_^+^ 371.0640; Found: 371.0639.

### General Procedure for Bridged Oxacycles **4a** and **4b**

To a high-pressure flask containing a solution of **3-(** ***E*** **)** (1 equiv) in DCM (0.1 M) was added BiBr_3_ (1.2 equiv) under argon. The flask was sealed, and the reaction was carried out at 40 °C overnight. Upon completion of the reaction, the reaction was cooled to room temperature and filtered through a Celite pad. The filtrate was concentrated with a rotary evaporator and further purified by silica gel column chromatography (Hex/EA; 4:1).

**3a-(** ***E*** **)** affords **4a** as a colorless oil after purification by silica gel column chromatography (Hex/EA; 4:1). ^1^H NMR (400 MHz, CDCl_3_) δ 7.22 (d, *J* = 2.6 Hz, 1H), 7.02 (d, *J* = 8.2 Hz, 1H), 6.72 (dd, *J* = 8.2, 2.6 Hz, 1H), 4.89 (d, *J* = 6.1 Hz, 1H), 4.51–4.34 (m, 1H), 3.82 (s, 3H), 3.52–3.33 (m, 1H), 3.14 (dd, *J* = 12.7, 6.3 Hz, 1H), 2.71 (dd, *J* = 18.5, 7.9 Hz, 1H), 2.53 (t, *J* = 12.6 Hz, 1H), 2.41 (dd, *J* = 18.6, 9.9 Hz, 1H), 2.23–2.14 (m, 1H), 2.05–1.71 (m, 3H). ^13^C­{^1^H} NMR (101 MHz, CDCl_3_) δ: 208.6, 159.3, 141.9, 133.3, 128.6, 127.7, 118.3, 114.2, 113.3, 80.7, 75.8, 55.4, 41.2, 37.9, 33.4, 32.2, 23.8. HRMS (ESI-Orbitrap) *m*/*z*: [M + H]^+^ Calcd for C_17_H_18_BrO_3_^+^ 349.0434; Found: 349.0431.

0.08 mmol of **3b-(** ***E*** **)** affords **4b** in 80% yield as a mixture (p/o 1.5:1), 21 mg as a colorless oil after purification by silica gel chromatography (Hex/EA; 4:1). ^1^H NMR (400 MHz, CHLOROFORM-*D*) δ 7.52 (d, *J* = 7.9 Hz, 1H), 7.45 (s, 1H, *minor isomer*), 7.17 (t, *J* = 7.7 Hz, 1H, *minor isomer*), 7.06 (d, *J* = 7.6 Hz, 1H), 6.99 (d, *J* = 4.4 Hz, 1H), 4.89 (d, *J* = 6.1 Hz, 1H, *minor isomer*), 4.85 (d, *J* = 6.1 Hz, 1H), 4.50 (dd, *J* = 5.5, 2.6 Hz, 1H), 4.48–4.44 (m, 1H, *minor isomer*), 3.70–3.64 (m, 1H), 3.44–3.38 (m, 1H, *minor isomer*), 3.14 (dd, *J* = 12.8, 6.3 Hz, 1H), 2.78–2.68 (m, 1H), 2.63–2.39 (m, 2H), 2.34 (s, 3H, *minor isomer*), 2.32 (s, 3H), 2.21–2.15 (m, 1H), 2.04–1.93 (m, 1H), 1.90–1.81 (m, 1H), 1.48–1.32 (m, 1H). ^13^C­{^1^H} NMR (101 MHz, CHLOROFORM-*D*) δ: 208.8, 208.8, 141.4, 140.9, 137.8, 134.5, 134.1, 133.6, 132.3, 132.0, 130.6, 129.1, 128.8, 127.4, 126.7, 126.3, 119.0, 118.6, 81.0, 80.9, 75.8, 75.6, 41.2, 39.0, 38.4, 34.6, 33.5, 33.4, 32.5, 32.4, 24.0, 23.9, 21.4, 19.0. HRMS (ESI-Orbitrap) *m*/*z*: [M + H]^+^ Calcd for C_17_H_18_BrO_2_^+^ 333.0485; Found: 333.0481.

#### Synthesis of Cyclic Acetal **9**

To a flame-dried scintillation vial equipped with a stir bar under argon was added **8** (0.2 mmol), TBAI (2 equiv), and DCM (0.1 M). The reaction mixture was cooled to 0 °C, and TfOH (1 equiv) was added, and stirred for 10 min under argon. *Quenching:* Upon completion, the reaction was quenched with solid NaHCO_3_, filtered through a Celite pad, and concentrated. The crude was further purified by silica gel column chromatography (Hex/EA; 4:1) to afford **9** in a 36% yield (28 mg) as a pale-yellow oil. ^1^H NMR (500 MHz, CDCl_3_) δ 7.21 (t, *J* = 7.9 Hz, 1H), 6.74–6.69 (m, 2H), 6.66 (s, 1H), 5.47 (d, *J* = 4.3 Hz, 1H), 4.28 (dd, *J* = 12.2, 6.8 Hz, 1H), 3.77 (s, 3H), 2.69–2.52 (m, 2H), 2.28–2.18 (m, 2H), 2.16–2.07 (m, 1H), 1.87–1.71 (m, 2H), 1.33–1.21 (m, 1H). ^13^C­{^1^H} NMR (126 MHz, CDCl_3_) δ: 160.1, 148.0, 147.6, 130.2, 120.7, 114.0, 113.8, 104.7, 93.5, 87.8, 79.8, 56.0, 35.8, 29.5, 29.5, 25.5. HRMS (ESI-Orbitrap) *m*/*z*: [M + H]^+^ Calcd for C_16_H_18_IO_3_^+^ 385.0295; Found: 385.0295.

#### Synthesis of Bridged Oxacycle **10**

To a solution of **9** (0.1 mmol) in DCM: HFIP (1:1, 0.1 M) at 0 °C was added TfOH (20 mol %) under an argon atmosphere and stirred for 10 min. *Quenching:* Upon completion, the reaction was quenched with solid NaHCO_3_, filtered through a Celite pad, and concentrated. The crude was further purified by silica gel column chromatography (Hex/EA; 2:1) to afford **10** in 46% yield (18 mg) as a white solid. ^1^H NMR (500 MHz, CDCl_3_) δ 7.74 (t, *J* = 2.8 Hz, 1H), 6.91 (dd, *J* = 8.0, 4.2 Hz, 1H), 6.62–6.59 (m, 1H), 5.23–5.03 (m, 1H), 4.33–4.29 (m, 1H), 3.80 (s, 3H), 2.68–2.50 (m, 2H), 2.46–2.35 (m, 1H), 2.34–2.22 (m, 2H), 2.19–1.95 (m, 3H), 1.70–1.57 (m, 1H). ^13^C­{^1^H} NMR (126 MHz, CDCl_3_) δ: 159.8, 156.3, 135.0, 134.9, 129.3, 126.1, 112.5, 104.1, 94.6, 83.6, 75.0, 56.1, 40.3, 35.2, 34.5, 29.3. m.p = 128–130 °C HRMS (ESI-Orbitrap) *m*/*z*: [M + H]^+^ Calcd for C_16_H_17_IO_3_^+^ 385.0295; Found: 385.0292.

#### One-Pot Synthesis of Bridged Oxacycle **10**

A flame-dried scintillating flask containing a solution of **8** (0.2 mmol) in DCM (0.1 M), was added TBAI (1.2 equiv) and cooled to 0 °C. TfOH (1.2 equiv) was then added to the reaction and stirred for ∼10 min. HFIP (0.5 M) was then added to the reaction and stirred overnight. *Quenching:* The reaction was quenched with solid NaHCO_3_, filtered through a Celite pad, and concentrated. The crude was further purified by silica gel column chromatography (Hex/EA; 2:1) to afford **10** in a 29% yield (22 mg) as a white solid.

## Supplementary Material



## Data Availability

The data underlying this study are available in the published article and its Supporting Information.

## Notes

Additional details of general synthetic procedures, characterization data, a further optimization table, and figures, NMR spectra for all compounds, and crystallographic data (PDF)
