# Supplementary material for: Alkynyl Halo-Prins Cyclizations for the Synthesis of Bicyclo[4.3.1] and [3.3.1] Oxygen-Bridged Heterocycles
Source: J Org Chem. 2025 Sep 9;90(37):13131–44. doi: 10.1021/acs.joc.5c01745 (PMC12455657; doi:10.1021/acs.joc.5c01745)
Supplement: Supplementary file 1 [file jo5c01745_si_001.pdf]

Supporting Information

Alkynyl *Halo-Prins* Cyclizations for the Synthesis of Bicyclo[4.3.1] and [3.3.1] Oxygen-Bridged Heterocycles

Yusuf A. Ibrahim, Alison J. Frontier*

Department of Chemistry, University of Rochester, 120 Trustee Road, Rochester, New York 14611, USA. E-mail: alison.frontier@rochester.edu

Table of Contents

General Remarks 3

Experimental procedures 4

General method A for Achmatowicz product **2:** 4

General method B for Achmatowicz product **2:** 6

General method C for Achmatowicz products **2q and 2r:** 12

Supplementary tables for experiment 13

NOESY Experiment of Identifying the Major Isomer 14

Assigning stereochemistry for **7i** 15

Formation of bridged oxacycles **10** 15

Gram-scale reaction 16

References 16

X-ray data 17

NMR Data 22

General Remarks

All vacuum/argon flushes and flame-drying methods were conducted using a Schlenk line, utilizing septa and needles (without Schlenk or multi-neck flasks unless explicitly mentioned). Reagents were used as provided by commercial suppliers without additional purification unless otherwise specified. Tetrahydrofuran (THF, stabilized with BHT), methylene chloride (DCM), methanol, and dimethylformamide (DMF) were sourced from Fisher and dried by adding vacuum/flame-dried 4 Å molecular sieves (typically a ~2 cm layer of spherical sieves per solvent bottle, for at least three days). The solvents were handled in an air atmosphere. Molecular sieves (3 Å and 5 Å) were purchased from Aldrich, stored in an oven at 120 °C before use, and further dried with a Tirrill burner under vacuum, followed by cooling under vacuum and exposure to argon. Screw-top tubes and flasks with thick glass walls were used to withstand higher pressures compared to regular round-bottom flasks (refer to CG-1880 from Chemglass). Celite 545 was obtained from EMD. ACS-grade hexanes, toluene, ethyl acetate, and DCM were used for column chromatography. Thin-layer chromatography (TLC) was performed on pre-coated silica gel 60 F254 glass plates (from EMD), with visualization via UV light, followed by staining with *p*-anisaldehyde or Seebach's solution and heating (using a hot plate until decoloration). Column chromatography was conducted using EM Science silica gel with a 60 Å pore size and a 230-400 mesh range. Preparative thin-layer chromatography (prep-TLC) utilized glass-supported, pre-coated silica gel 60 F254 plates from EMD, which were also trimmed for use in regular TLC analysis. Deuterated solvents were obtained from Cambridge Isotope Laboratories.

^{1}H NMR spectra were measured at ambient temperature (unless otherwise noted) using either a Bruker Avance spectrometer (400 and 500 MHz) or a JOEL spectrometer (400 and 500 MHz), with data processed in MestReNova version 14.2.1-27684. Chemical shifts are expressed in parts per million (ppm), referenced to the residual proton signal of the solvent (δ = 7.26 for $CHCl_3$, or using the built-in reference values in MestReNova for other solvents). NMR results are provided as follows: chemical shift, multiplicity (s = singlet, d = doublet, t = triplet, q = quartet, p = pentet/quintet, m = multiplet, and combinations like dt = doublet of triplets), coupling constants (J) in Hz, and integration. For samples containing two or more diastereomers or *Z/E*-isomers, chemical shifts of both isomers are presented, and the best-resolved peaks without overlap were used to calculate the diastereomeric ratio (dr) or *Z/E* ratio.

The ^{13}C NMR spectra were obtained at room temperature, unless otherwise specified, using a 126 MHz or 101 MHz Bruker Avance and JEOL spectrometer with proton decoupling. Chemical shifts are reported in parts per million (ppm) and are referenced to the carbon resonance of the solvent (δ = 77.0 for $CHCl_3$, or based on the built-in reference values for other solvents in MestReNova version 14.2.1-27684). When two or more mixtures of isomers (dr or *E/Z*) are present, the chemical shifts for all isomers are provided together.

Experimental procedures

General method A for Achmatowicz product **2:**

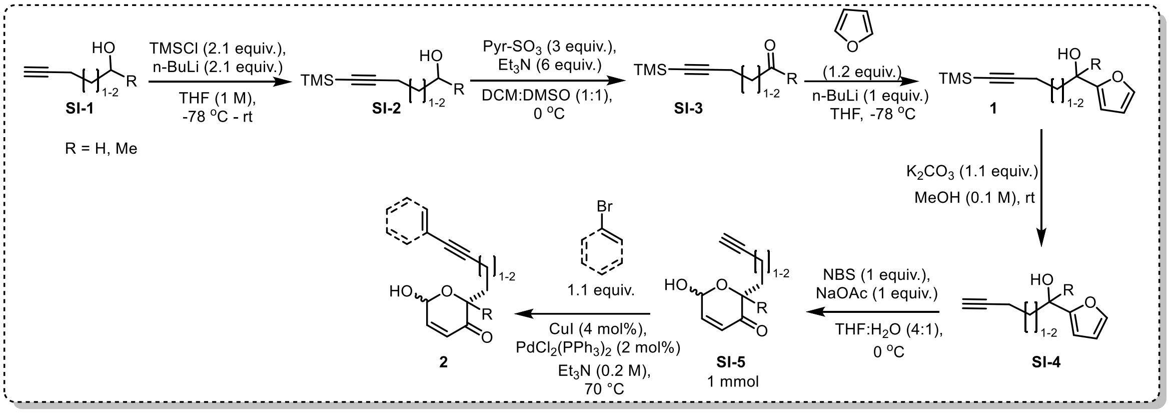

Scheme S1. Procedure A for synthesizing Achmatowicz adduct **2**.

To a solution of alkyne **SI-1** (1 equiv.) in THF (1 M), n-BuLi (2.5 M in hexanes, 2.1 equiv.) was added dropwise at –78 °C. The mixture was allowed to gradually warm to room temperature and stirred for 30 min, before being cooled back to –78 °C. TMSCl (2.2 equiv.) was then added dropwise, and the reaction was allowed to warm to room temperature. Stirring continued until completion as monitored by TLC (visualized using stain). The reaction mixture was quenched with 1 M HCl aqueous solution and vigorously stirred for 1 hour. The aqueous layer was extracted twice with Et_2O, and the combined organic layers were washed with brine, dried over anhydrous $MgSO_4$, filtered, and evaporated. The crude mixture was further purified by column chromatography (Hex: EA; 2:1), yielding **SI-2**. The NMR data is consistent with the literature.[1,2]

To a round-bottom flask equipped with a stir bar and exposed to air, **SI-2** (1 equiv.) was added, followed by reagent-grade DCM (0.5 M), and Et_3N (6 equiv.). In a separate Erlenmeyer flask, Pyr-SO_3 (3 equiv.) was added, followed by reagent-grade DMSO (0.5 M) to form a slurry. The round-bottom flask was cooled to 0 °C in an ice-water bath while stirring. After a few minutes, the Pyr-SO_3/DMSO slurry was added to the reaction mixture in one portion. Upon completion of the reaction, as confirmed by TLC analysis (visualized with stain), the reaction mixture was quenched with 1 M HCl. The mixtures were transferred to a separatory funnel, the layers separated, and the organic layer was washed with 1 M HCl until the aqueous layer reached a pH of about 1. The organic layer was then washed with water and brine, dried over anhydrous $MgSO_4$, filtered, and concentrated using rotary evaporation to afford **SI-3**. The crude aldehyde **SI-3** was used in the next step without further purification.[3] *Caution: The byproduct dimethylsulfide has a very strong odor.*

In a flame-dried round-bottom flask containing a solution of furan-2-yllithium, prepared from furan (1.2 equiv.) and n-BuLi (2.5 M in hexane, 1 equiv.) in THF (0.9 M), was added a solution of aldehyde **SI-3** (1 equiv.) in THF (0.9 M) at -78 °C under argon. Once the reaction was complete, as confirmed by TLC (visualized with stain), the reaction mixture was quenched with sat. NH_4Cl and allowed to warm to room temperature. The mixture was extracted with Et_2O, washed with water and brine, dried over $MgSO_4$, filtered, and concentrated by rotary evaporation. The crude product was then purified by column chromatography using hexanes (Hex) and ethyl acetate (EA) as the eluent to yield **1**.[4]

Compound **1a**[4]

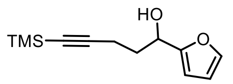

Purification by silica gel column chromatography (Hex: EA; 3:1), 37% yield as yellow oil. ^{1}H NMR (400 MHz, $CDCl_3$) δ 7.38 – 7.33 (m, 1H), 6.34 – 6.21 (m, 2H), 4.82 (q, *J* = 6.2 Hz, 1H), 2.45 – 2.25 (m, 2H), 2.13 (d, *J* = 5.1 Hz, 1H), 2.03 (q, *J* = 6.9 Hz, 2H), 0.13 (s, 9H). The NMR data is consistent with the literature.[4] HRMS (ESI-Orbitrap) *m/z*: $[M-OH]^+$ Calcd for $C_{12}H_{17}OSi^+$ 205.1043; Found: 205.1043.

Compound **1b**

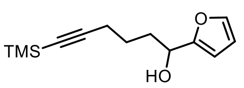

Purification by silica gel column chromatography (Hex: EA; 3:1), 59% yield as amber oil. ^{1}H NMR (400 MHz, CHLOROFORM-*D*) δ 7.35 (dd, *J* = 1.8, 0.8 Hz, 1H), 6.31 (dd, *J* = 3.3, 1.8 Hz, 1H), 6.22 (dt, *J* = 3.3, 0.7 Hz, 1H), 4.67 (t, *J* = 6.8 Hz, 1H), 2.24 (td, *J* = 7.0, 1.0 Hz, 2H), 2.00 – 1.87 (m, 2H), 1.70 – 1.59 (m, 1H), 1.53 (dtd, *J* = 13.4, 6.7, 2.3 Hz, 1H), 0.12 (s, 9H). $^{13}C\{^{1}H\}$ NMR (101 MHz, CHLOROFORM-*D*) δ 156.6, 142.1, 110.2, 107.1, 106.0, 85.0, 67.3, 34.6, 24.7, 19.7, 0.2. HRMS (ESI-Orbitrap) *m/z*: $[M-OH]^+$ Calcd for $C_{13}H_{19}OSi^+$ 219.1200; Found: 219.1200.

K_2CO_3 (1.1 equiv.) was added to a round bottom flask containing a solution of **1** (1 equiv.) in MeOH (0.1 M), and the reaction was stirred at room temperature until **1** was completely consumed according to the TLC (Visualized with stain). The reaction mixture was quenched water and Et_2O was used for extraction. The organic layer was further washed with brine, dried over $MgSO_4$, filtered, and concentrated using rotary evaporation. The crude was used in the next step without further purification.

To a stirred solution of furfuryl alcohol **SI-4** and anhydrous NaOAc (1 equiv.) in aqueous THF (0.2 M; THF: H_2O 4:1), NBS (1 equiv.) was added gradually at 0 °C, and the mixture was stirred at the same temperature (0 °C). Once the reaction was complete, it was quenched with sat. $Na_2S_2O_3$, and the mixture was extracted with Et_2O. The organic layer was then washed with brine, dried over $MgSO_4$, and the solvent was evaporated to yield a crude residue, which was purified by silica gel column chromatography (Hex: EA; 2:1) to afford **SI-5** as a dr mixture.

Compound **SI-5a**

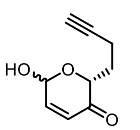

Purification by silica gel column chromatography (Hex: EA; 2:1) affords a 2:1 dr mixture was isolated as an amber oil in 66% yield. ^{1}H NMR (500 MHz, CHLOROFORM-*D*) δ 6.97 – 6.85 (m, 1H), 6.15 – 6.09 (m, 1H), 5.65 (d, *J* = 18.8 Hz, 1H), 4.75 – 4.18 (m, 1H), 3.74 – 3.35 (m, 1H), 2.44 – 2.29 (m, 2H), 2.23 – 2.15 (m, 1H), 2.00 – 1.92 (m, 1H), 1.89 – 1.79 (m, 1H). $^{13}C\{^{1}H\}$ NMR (126 MHz, CHLOROFORM-*D*) δ 196.3, 195.9, 148.0, 144.5, 128.8, 127.6, 91.0, 87.7, 83.3, 83.2, 72.3, 69.5, 69.4, 29.4, 28.5, 14.4, 14.3. HRMS (ESI-Orbitrap) *m/z*: $[M-OH]^+$ Calcd for $C_9H_9O_2^+$ 149.0597; Found: 149.0597.

Compound **SI-5b**

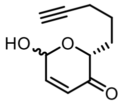

Purification by silica gel column chromatography (Hex: EA; 2:1) affords a 2:1 dr mixture was isolated as an amber oil in 49% yield. ^{1}H NMR (400 MHz, CHLOROFORM-*D*) δ 6.95 – 6.83 (m, 1H), 6.08 (ddt, *J* = 18.8, 10.2, 1.2 Hz, 1H), 5.60 (t, *J* = 5.7 Hz, 1H), 4.55 (ddd, *J* = 8.0, 3.9, 1.1 Hz, 1H), 4.12 – 3.98 (m, 1H), 2.19 (tdd, *J* = 7.1, 3.4, 2.4 Hz, 2H), 2.09 – 1.94 (m, 1H), 1.90 – 1.51 (m, 3H). $^{13}C\{^{1}H\}$ NMR (101 MHz, CHLOROFORM-*D*) δ 196.9, 196.4, 148.4, 145.0, 128.7, 127.5, 90.9, 87.6, 84.1, 84.1, 78.4, 73.7, 69.0, 68.9, 29.7, 28.7, 24.2, 24.0, 18.3, 18.3. HRMS (ESI-Orbitrap) *m/z*: $[M-OH]^+$ Calcd for $C_{10}H_{11}O_2^+$ 163.0753; Found: 163.0753.

A round-bottom flask equipped with a stir bar was purged with argon several times, aryl halide (1.1 equiv.) and Et_3N (0.2 M) was then added to the flask, which was degassed by sparging with argon through a long needle. After 10 minutes, the degassing was stopped, and **SI-5** (1 equiv., 1 mmol), $PdCl_2(PPh_3)_2$ (2 mol%) and CuI (4 mol%) were added. The reaction was carried out at 70 °C until the starting material was fully consumed, as monitored by TLC. The reaction was then cooled to room temperature and filtered through a Celite 545 pad, which was washed with excess Et_2O (also used for extraction). The filtrate was transferred to a separatory funnel, combined with ice, and washed with 1 M HCl to remove Et_3N. Once the aqueous layer reached a pH of around 1, the organic layer was washed with sat. $NaHCO_3$ solution, followed by water. The organic layer was dried over $MgSO_4$, filtered, concentrated, and purified by column chromatography (using a step gradient from toluene to 30% EA in Hex) to obtain pure product **2** as a dr-mixture (Scheme S1).

General method B for Achmatowicz product **2:**

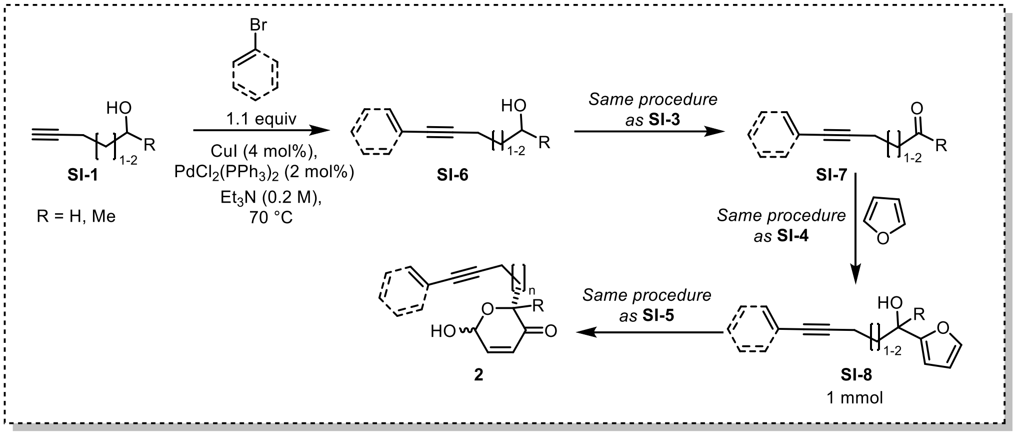

Scheme S2. Procedure B for synthesizing Achmatowicz adduct **2**.

Note: The reaction procedure for **SI-6** is the same as the procedure for **2** in Scheme S1.

Compound **2a**

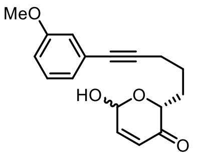

General procedure A or B affords **2a** in 98% yield (dr 2:1), 281 mg as a yellow oil after purification by silica gel column chromatography (Hex: EA; 2:1). ^{1}H NMR (500 MHz, $CDCl_3$) δ 7.15 (t, J = 8.0 Hz, 1H), 6.96 (dq, J = 7.5, 1.3 Hz, 1H), 6.92 – 6.84 (m, 2H), 6.80 (dd, J = 8.3, 2.7 Hz, 1H), 6.17 – 6.03 (m, 1H), 5.62 (q, J = 3.3 Hz, 1H), 4.60 – 4.09 (m, 1H), 3.76 (s, 3H), 2.42 (td, J = 7.1, 3.8 Hz, 2H), 2.16 – 2.02 (m, 1H), 1.98 – 1.65 (m, 4H). $^{13}C\{^1H\}$ NMR (126 MHz, $CDCl_3$) δ 197.2, 196.8, 160.0, 148.6, 145.2, 130.0, 129.4, 128.3, 125.6, 124.9, 117.2, 115.0, 114.9, 91.6, 90.3, 90.2, 88.4, 81.8, 81.7, 79.2, 74.4, 56.0, 30.6, 29.6, 25.1, 25.0, 20.0, 20.0. HRMS (ESI-Orbitrap) *m/z*: $[M-OH]^+$ Calcd for $C_{17}H_{17}O_2^+$ 269.1172; Found: 269.1170.

Compound **2b**

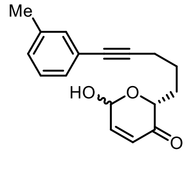

General procedure A or B affords **2b** in 85% yield (dr 2:1), 230 mg as a yellow solid after purification by silica gel column chromatography (Hex: EA; 2:1). ^{1}H NMR (400 MHz, CHLOROFORM-*D*) δ 7.21 – 7.10 (m, 3H), 7.06 (d, J = 7.3 Hz, 1H), 6.94 – 6.83 (m, 1H), 6.16 – 6.05 (m, 1H), 5.63 – 5.61 (m, 1H), 4.63 – 4.10 (m, 1H), 3.80 – 3.42 (m, 1H), 2.47 – 2.41 (m, 2H), 2.29 (s, 3H), 2.17 – 2.03 (m, 1H), 1.98 – 1.65 (m, 3H). $^{13}C\{^1H\}$ NMR (101 MHz, CHLOROFORM-*D*) δ 196.7, 196.2, 148.1, 144.7, 138.0, 132.3, 128.8,

128.7, 128.6, 128.6, 128.2, 127.6, 123.7, 91.0, 89.3, 89.3, 87.7, 81.3, 81.3, 78.6, 73.8, 29.9, 29.0, 24.5, 24.4, 21.3, 19.3, 19.3. HRMS (ESI-Orbitrap) *m/z*: $[M\text{-}OH]^+$ Calcd for $C_{17}H_{17}O_2^+$ 253.1223; Found: 253.1223.

Compound **2c**

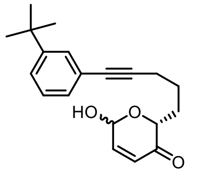

General procedure A or B affords **2c** in 87% yield (dr 2:1), 272 mg as a yellow oil after purification by silica gel column chromatography (Hex: EA; 2:1). ^{1}H NMR (400 MHz, CHLOROFORM-*D*) δ 7.41 – 7.39 (m, 1H), 7.30 – 7.25 (m, 1H), 7.25 – 7.14 (m, 2H), 6.93 – 6.86 (m, 1H), 6.18 – 6.05 (m, 1H), 5.64 (d, *J* = 3.5 Hz, 1H), 4.62 (dd, *J* = 7.9, 3.9 Hz, 1H), 4.18 – 4.06 (m, 1H), 2.47 – 2.43 (m, 2H), 2.15 – 2.09 (m, 1H), 1.98 – 1.63 (m, 3H), 1.29 (s, 9H). ^{13}C{^{1}H} NMR (101 MHz, CHLOROFORM-*D*) δ 196.4, 151.2, 147.8, 144.5, 128.8, 128.8, 128.7, 128.0, 127.7, 124.9, 123.4, 91.0, 89.0, 87.8, 81.6, 78.6, 73.8, 34.7, 31.3, 30.0, 29.0, 24.6, 24.5, 19.4, 19.3. HRMS (ESI-Orbitrap) *m/z*: $[M\text{-}OH]^+$ Calcd for $C_{20}H_{23}O_2^+$ 295.1692; Found: 295.1692.

Compound **2d**

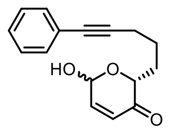

General procedure A or B affords **2d** in 83% yield (dr 2:1), 213 mg as a yellow oil after purification by silica gel column chromatography (Hex: EA; 2:1). ^{1}H NMR (400 MHz, CHLOROFORM-*D*) δ 7.39 – 7.37 (m, 2H), 7.27 – 7.25 (m, 3H), 6.92 – 6.85 (m, 1H), 6.15 – 6.08 (m, 1H), 5.64 – 5.62 (m, 1H), 4.63 – 4.11 (m, 1H), 3.67 – 3.38 (m, 1H), 2.47 – 2.40 (m, 2H), 2.17 – 2.05 (m, 1H), 1.98 – 1.65 (m, 3H). ^{13}C{^{1}H} NMR (101 MHz, CHLOROFORM-*D*) δ 196.6, 196.2, 148.0, 144.6, 131.7, 128.8, 128.3, 127.7, 127.7, 127.7, 123.9, 123.9, 91.0, 89.7, 89.7, 87.7, 81.1, 78.6, 73.8, 30.0, 29.0, 24.5, 24.4, 19.4, 19.3. HRMS (ESI-Orbitrap) *m/z*: $[M\text{-}OH]^+$ Calcd for $C_{16}H_{15}O_2^+$ 239.1066; Found: 239.1066.

Compound **2e**

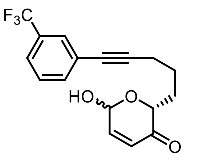

General procedure A or B affords **2e** in 99% yield (dr 1.5:1), 321 mg as an amber oil after purification by silica gel column chromatography (Hex: EA; 2:1). ^{1}H NMR (400 MHz, $CDCl_3$) δ 7.64 (s, 1H), 7.52 (dd, *J* = 17.4, 7.8 Hz, 2H), 7.39 (t, *J* = 7.8 Hz, 1H), 6.95 – 6.88 (m 1H), 6.17 – 6.10 (m, 1H), 5.67 – 5.65 (m, 1H), 4.63 (dd, *J* = 7.8, 3.9 Hz, 1H), 3.29 (d, *J* = 98.1 Hz, 1H), 2.48 – 2.44 (m, 2H), 2.15 – 2.07 (m, 1H), 2.00 – 1.70 (m, 3H). ^{13}C{^{1}H} NMR (101 MHz, $CDCl_3$) δ 196.1, 147.6, 144.2, 134.6, 128.6, 128.3, 127.6, 124.1, 91.4, 90.9, 87.6, 78.4, 73.6, 29.8, 28.8, 24.2, 24.1, 19.2. HRMS (ESI-Orbitrap) *m/z*: $[M\text{-}OH]^+$ Calcd for $C_{17}H_{14}F_3O_2^+$ 307.0940; Found: 307.0940.

Compound **2f**

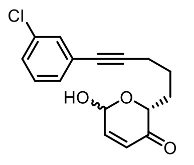

General procedure A or B affords **2f** in 85% yield (dr 3:1), 247 mg as a yellow oil after purification by silica gel column chromatography (Hex: EA; 2:1). ^{1}H NMR (500 MHz, CHLOROFORM-*D*) δ 7.37 – 7.35 (m, 1H), 7.27 – 7.15 (m, 3H), 6.94 – 6.87 (m, 1H), 6.16 – 6.10 (m, 1H), 5.66 – 5.65 (m, 1H), 4.63 – 4.09 (m, 1H), 2.44 – 2.42 (m, 2H), 2.17 – 2.05 (m, 1H), 2.00 – 1.62 (m, 4H). ^{13}C{^{1}H} NMR (126 MHz, CHLOROFORM-*D*) δ 196.3, 195.9, 147.8, 144.4, 134.1, 134.1, 131.6, 131.6, 129.8, 129.5, 129.5, 128.9, 128.0, 128.0, 127.7, 127.5, 125.7, 125.7, 91.1, 91.1, 91.0, 87.8, 87.8, 79.9, 79.9, 73.7, 30.0, 28.9, 24.4, 24.3, 19.3, 19.3. HRMS (ESI-Orbitrap) *m/z*: $[M\text{-}OH]^+$ Calcd for $C_{16}H_{14}ClO_2^+$ 273.0677; Found: 273.0677.

Compound **2g**

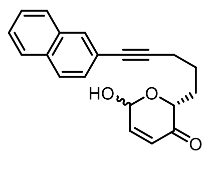

General procedure A or B affords **1g** in 91% yield (dr 3:1), 279 mg as a yellow oil after purification by silica gel column chromatography (Hex: EA; 2:1). ^{1}H NMR (500 MHz, CHLOROFORM-*D*) δ 7.90 (s, 1H), 7.78 – 7.72 (m, 3H), 7.46 – 7.43 (m, 3H), 6.92 – 6.86 (m, 1H), 6.16 – 6.09 (m, 1H), 5.64 (d, *J* = 3.3 Hz, 1H), 4.66 – 4.09 (m, 1H), 2.52 – 2.47 (m, 2H), 2.22 – 2.09 (m, 1H), 2.03 – 1.70 (m, 3H). ^{13}C{^{1}H} NMR (126 MHz, CHLOROFORM-*D*) δ 196.5, 196.1, 148.0, 144.6, 133.1, 132.6, 131.2, 128.8, 127.9, 127.9, 127.8, 127.7, 127.7, 126.5, 126.4, 121.3, 91.0, 90.2, 90.1, 87.8, 81.5, 78.6, 73.8, 60.6, 30.0, 29.0, 24.6, 24.5, 21.2, 19.5, 14.3. HRMS (ESI-Orbitrap) *m/z*: [M-OH]$^+$ Calcd for $C_{20}H_{17}O_2^+$ 289.1223; Found: 289.1223.

Compound **2h**

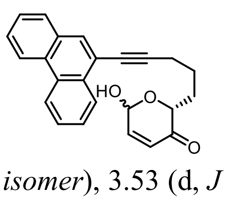

General procedure A or B affords **2h** in 49% yield (dr 1.2:1), 175 mg as a yellow oil after purification by silica gel column chromatography (Hex: EA; 2:1). ^{1}H NMR (400 MHz, CHLOROFORM-*D*) δ 8.75 – 8.57 (m, 2H), 8.51 – 8.39 (m, 1H), 7.93 (s, 1H), 7.81 (dd, *J* = 7.9, 1.5 Hz, 1H), 7.72 – 7.45 (m, 4H), 6.91 – 6.80 (m, 1H), 6.14 – 6.09 (m, 1H), 5.62 – 5.58 (m, 1H), 4.67 (dd, *J* = 7.8, 3.9 Hz, 1H), 4.20 – 4.06 (m, 1H, *minor isomer*), 3.53 (d, *J* = 7.1 Hz, 1H, *minor isomer*), 3.28 (d, *J* = 4.9 Hz, 1H), 2.64 (t, *J* = 7.0 Hz, 2H), 2.29 – 2.18 (m, 1H), 2.11 – 1.79 (m, 3H). ^{13}C{^{1}H} NMR (101 MHz, CHLOROFORM-*D*) δ 196.5, 196.1, 147.9, 144.5, 131.6, 131.5, 131.4, 130.2, 130.1, 128.8, 128.5, 127.7, 127.2, 127.1, 127.1, 127.1, 127.0, 127.0, 122.8, 122.7, 120.3, 120.3, 94.5, 94.5, 91.0, 87.8, 79.3, 78.5, 73.8, 30.1, 29.1, 24.7, 24.6, 19.8, 19.7. HRMS (ESI-Orbitrap) *m/z*: [M-OH]$^+$ Calcd for $C_{24}H_{19}O_2^+$ 339.1379; Found: 339.1379.

Compound **2i**

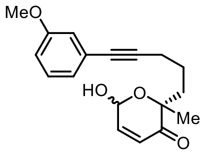

General procedure A or B affords **2i** in 83% yield (dr 1:1), 249 mg as a yellow oil after purification by silica gel column chromatography (Hex: EA; 2:1). ^{1}H NMR (500 MHz, CHLOROFORM-*D*) δ 7.17 (td, *J* = 8.0, 2.5 Hz, 1H), 6.98 – 6.95 (m, 1H), 6.92 – 6.89 (m, 1H), 6.87 – 6.84 (m, 1H), 6.83 – 6.79 (m, 1H), 6.09 – 6.06 (m, 1H), 5.73 – 5.69 (m, 1H), 3.77 (s, 3H), 3.18 – 3.14 (m, 1H), 2.41 – 2.37 (m, 2H), 2.12 – 1.98 (m, 1H), 1.92 – 1.69 (m, 2H), 1.48 (s, 3H). ^{13}C{^{1}H} NMR (126 MHz, CHLOROFORM-*D*) δ 199.0, 198.8, 159.3, 159.3, 146.1, 145.3, 145.3, 129.4, 129.3, 127.1, 126.8, 126.8, 125.0, 124.9, 124.2, 124.2, 116.5, 116.5, 114.3, 89.7, 89.5, 88.0, 87.8, 81.7, 81.7, 81.1, 81.0, 55.3, 38.1, 36.6, 25.4, 22.9, 22.8, 21.7, 19.7, 19.6. HRMS (ESI-Orbitrap) *m/z*: [M-OH]$^+$ Calcd for $C_{18}H_{19}O_3^+$ 283.1329; Found: 283.1329.

Compound **2j**

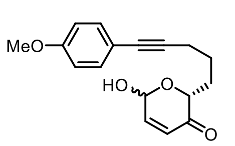

General procedure A or B affords **2j** in 90% yield (dr 1:1), 258 mg as an amber oil after purification by silica gel column chromatography (Hex: EA; 2:1). ^{1}H NMR (400 MHz, CHLOROFORM-*D*) δ 7.31 (d, *J* = 8.3 Hz, 2H), 6.95 – 6.84 (m, 1H), 6.79 (d, *J* = 9.1 Hz, 2H), 6.12 (dd, *J* = 16.8, 10.1 Hz, 1H), 5.64 (s, 1H), 4.63 – 4.10 (m, 1H), 3.78 (s, 3H), 3.34 (dd, *J* = 107.4, 6.0 Hz, 1H), 2.46 – 2.37 (m, 2H), 2.15 – 2.01 (m, 1H), 1.97 – 1.64 (m, 4H). ^{13}C{^{1}H} NMR (101 MHz, CHLOROFORM-*D*) δ 196.5, 159.1, 147.8, 144.4, 133.9, 133.0, 128.8, 127.7, 116.1, 113.9, 113.3, 91.0, 88.1, 87.8, 80.8, 78.6, 73.8, 55.4, 30.0, 29.0, 24.6, 24.5, 19.4, 19.3. HRMS (ESI-Orbitrap) *m/z*: [M-OH]$^+$ Calcd for $C_{17}H_{17}O_3^+$ 269.1172; Found: 269.1171.

Compound **2k**

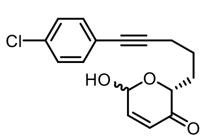

General procedure A or B affords **2k** in 59% yield (dr 6:1), 172 mg as a white solid after purification by silica gel column chromatography (Hex: EA; 2:1). ^{1}H NMR (500 MHz, CHLOROFORM-*D*) δ 7.30 (d, *J* = 8.5 Hz, 2H), 7.23 (d, *J* = 8.5 Hz, 2H), 6.93 – 6.86 (m, 1H), 6.16 – 5.82 (m, 1H), 5.65 – 5.64 (m, 1H), 4.63 – 4.61 (m, 1H), 2.75 (s, 1H), 2.45 – 2.42 (m, 2H), 2.15 – 2.05 (m, 1H), 1.89 – 1.80 (m, 1H), 1.78 – 1.68 (m, 2H). ^{13}C{^{1}H} NMR (126 MHz, CHLOROFORM-*D*) δ 196.3, 195.9, 147.7, 144.3, 133.6, 133.6, 132.9, 128.9, 128.6, 128.6, 127.8, 122.5, 91.0, 90.8, 87.8, 80.1, 78.6, 73.7, 30.0, 29.6, 28.9, 24.4, 24.3, 19.4, 19.3. m.p = 115 – 117 °C. HRMS (ESI-Orbitrap) *m/z*: [M-OH]$^+$ Calcd for $C_{16}H_{14}ClO_2^+$ 273.0677; Found: 273.0671.

Compound **2l**

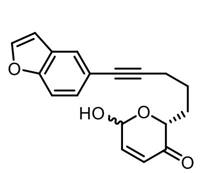

General procedure A or B affords **2l** in 82% yield (dr 2:1), 243 mg as yellow oil after purification by silica gel column chromatography (Hex: EA; 2:1). ^{1}H NMR (500 MHz, $CDCl_3$) δ 7.61 (dd, *J* = 19.3, 2.0 Hz, 2H), 7.39 – 7.30 (m, 2H), 6.93 – 6.85 (m, 1H), 6.70 (d, *J* = 1.3 Hz, 1H), 6.12 (dd, *J* = 21.0, 10.3 Hz, 1H), 5.65 (d, *J* = 3.2 Hz, 1H), 4.63 (dd, *J* = 8.1, 3.9 Hz, 1H), 2.83 (s, 1H), 2.49 – 2.40 (m, 2H), 2.17 – 2.07 (m, 1H), 1.90 – 1.81 (m, 1H), 1.77 – 1.71 (m, 1H). ^{13}C{^{1}H} NMR (126 MHz, $CDCl_3$) δ 197.1, 196.8, 154.9, 148.6, 146.4, 145.2, 129.4, 128.7, 128.3, 128.2, 125.3, 119.1, 112.1, 107.2, 91.6, 88.8, 88.7, 88.4, 81.9, 79.2, 74.5, 30.7, 30.4, 29.6, 25.2, 25.1, 20.0, 20.0, 14.9. HRMS (ESI-Orbitrap) *m/z*: [M-OH]$^+$ Calcd for $C_{18}H_{15}O_3^+$ 279.1016; Found: 279.1014.

Compound **2m**

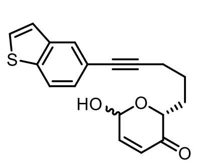

General procedure A or B affords **2m** in 90% yield (dr 2:1), 281 mg as yellow oil after purification by silica gel column chromatography (Hex: EA; 2:1). ^{1}H NMR (400 MHz, $CDCl_3$) δ 7.86 (s, 1H), 7.77 (d, *J* = 8.2 Hz, 1H), 7.44 (d, *J* = 5.4 Hz, 1H), 7.35 (d, *J* = 8.4 Hz, 1H), 7.28 (d, *J* = 5.3 Hz, 1H), 6.95 – 6.85 (m, 1H), 6.18 – 6.11 (m, 1H), 5.67 (s, 1H), 4.65 (dd, *J* = 7.9, 3.8 Hz, 1H), 3.02 – 2.91 (m, 1H), 2.50 – 2.47 (m, 2H), 2.17 – 2.14 (m, 1H), 1.94 – 2.14 (m, 3H). ^{13}C{^{1}H} NMR (101 MHz, $CDCl_3$) δ 196.3, 144.3, 139.5, 127.6, 127.4, 127.1, 126.7, 123.6, 122.2, 119.7, 90.8, 89.0, 87.6, 81.1, 78.5, 73.7, 29.9, 28.8, 24.3, 19.3. HRMS (ESI-Orbitrap) *m/z*: [M-OH]$^+$ Calcd for $C_{18}H_{15}O_2S^+$ 295.0787; Found: 295.0787.

Compound **2n**

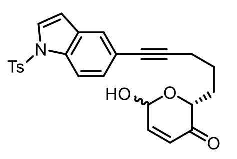

General procedure A or B affords **2n** in 72% yield (dr 1.2:1), 324 mg as a yellow oil after purification by silica gel column chromatography (Hex: EA; 2:1). ^{1}H NMR (400 MHz, $CDCl_3$) δ 7.87 (d, *J* = 8.6 Hz, 1H), 7.72 (d, *J* = 8.0 Hz, 2H), 7.57 – 7.48 (m, 2H), 7.32 (d, *J* = 8.6 Hz, 1H), 7.20 (d, *J* = 8.0 Hz, 2H), 7.00 – 6.81 (m, 1H), 6.58 (d, *J* = 3.7 Hz, 1H), 6.11 (dd, *J* = 16.2, 10.2 Hz, 1H), 5.64 (d, *J* = 3.4 Hz, 1H), 4.62 (dd, *J* = 8.0, 3.9 Hz, 1H), 4.13 (dd, *J* = 8.5, 3.9 Hz, 1H, *minor isomer*), 3.64 – 3.37 (m, 1H, *minor isomer*), 3.22 (s, 1H), 2.46 – 2.42 (m, 2H), 2.32 (s, 3H), 2.15 – 2.03 (m, 1H), 1.91 – 1.65 (m, 3H). ^{13}C{^{1}H} NMR (101 MHz, $CDCl_3$) δ 196.3, 147.7, 145.1, 144.3, 135.0, 133.9, 130.6, 129.9, 128.6, 128.1, 127.5, 127.0, 126.7, 124.6, 118.9, 113.4, 108.9, 90.8, 88.7, 87.6, 78.4, 73.6, 29.8, 28.8, 24.4, 24.3, 21.5, 19.2. HRMS (ESI-Orbitrap) *m/z*: [M-OH]$^+$ Calcd for $C_{25}H_{22}NO_4S^+$ 432.1264; Found: 432.1267.

Compound **2o**

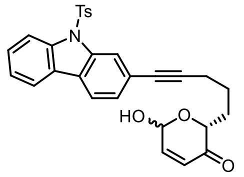

General procedure A or B affords **2o** in 77% yield (dr 3:1), 384 mg as an amber oil after purification by silica gel column chromatography (Hex: EA; 2:1). ^{1}H NMR (500 MHz, CHLOROFORM-*D*) δ 8.38 (s, 1H), 8.32 – 8.23 (m, 1H), 7.88 – 7.75 (m, 2H), 7.69 (dd, *J* = 8.5, 1.9 Hz, 2H), 7.54 – 7.30 (m, 3H), 7.10 (dd, *J* = 8.1, 1.8 Hz, 2H), 6.96 – 6.89 (m, 1H), 6.18 – 6.10 (m, 1H), 5.71 – 5.69 (m, 1H), 4.70 – 4.68 (m, 1H), 3.34 (dd, *J* = 136.0, 5.9 Hz, 1H), 2.54 – 2.50 (m, 2H), 2.25 (s, 3H), 2.04 – 1.75 (m, 3H). ^{13}C{^{1}H} NMR (126 MHz, CHLOROFORM-*D*) δ 196.5, 147.9, 145.1, 144.5, 138.8, 138.2, 134.9, 129.9, 128.9, 127.8, 127.7, 127.6, 126.6, 126.1, 125.8, 124.1, 123.0, 120.2, 119.9, 118.3, 115.2, 91.0, 90.6, 87.8, 81.6, 78.6, 73.7, 30.1, 29.0, 24.5, 24.4, 21.6, 19.5, 19.5. HRMS (ESI-Orbitrap) *m/z*: [M-OH]$^{+}$ Calcd for $C_{29}H_{24}NO_4S^{+}$ 482.1420; Found: 482.1421.

Compound **2p**

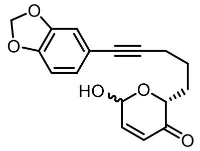

General procedure A or B affords **2p** in 83% yield (dr 2:1), 249 mg as a grey oil after purification by silica gel column chromatography (Hex: EA; 2:1). ^{1}H NMR (400 MHz, $CDCl_3$) δ 6.93 – 6.84 (m, 2H), 6.83 – 6.84 (m, 1H), 6.70 (dd, *J* = 8.0, 1.1 Hz, 1H), 6.19 – 6.03 (m, 1H), 5.93 (s, 2H), 5.64 (s, 1H), 4.63 – 4.60 (m, 1H), 3.57 – 3.25 (m, 1H), 2.44 – 2.39 (m, 2H), 2.14 – 2.03 (m, 1H), 1.95 – 1.62 (m, 3H). ^{13}C{^{1}H} NMR (101 MHz, $CDCl_3$) δ 196.3, 195.9, 147.7, 147.2, 144.4, 128.7, 127.5, 125.8, 117.1, 111.6, 108.3, 101.1, 90.8, 87.8, 87.6, 80.7, 78.4, 73.6, 29.8, 28.8, 24.4, 24.3, 19.2, 19.1. HRMS (ESI-Orbitrap) *m/z*: [M-OH]$^{+}$ Calcd for $C_{17}H_{15}O_4^{+}$ 283.0965; Found: 283.0963.

Compound **2s**

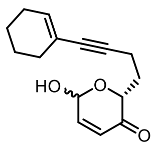

General procedure A or B affords **2s** in 85% yield (dr 2:1), 209 mg as a yellow oil after purification by silica gel column chromatography (Hex: EA; 2:1). ^{1}H NMR (400 MHz, CHLOROFORM-*D*) δ 6.94 – 6.86 (m, 1H), 6.12 – 6.08 (m, 1H), 6.00 – 5.97 (m, 1H), 5.67 – 5.63 (m, 1H), 4.75 – 4.71 (m, 1H), 3.58 – 3.26 (m, 1H), 2.51 – 2.46 (m, 2H), 2.21 – 2.12 (m, 1H), 2.16 – 2.03 (m, 4H), 1.99 – 1.75 (m, 1H), 1.62 – 1.50 (m, 4H). ^{13}C{^{1}H} NMR (101 MHz, CHLOROFORM-*D*) δ 196.4, 196.1, 147.8, 144.3, 133.8, 133.8, 128.9, 127.7, 120.9, 91.0, 87.8, 85.7, 85.7, 83.5, 72.5, 31.1, 29.9, 29.6, 29.6, 28.9, 25.6, 22.4, 21.6, 15.3, 15.1. HRMS (ESI-Orbitrap) *m/z*: [M-OH]$^{+}$ Calcd for $C_{15}H_{17}O_2^{+}$ 229.1223; Found: 229.1223.

Compound **2t**

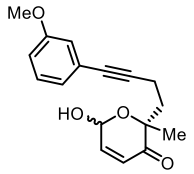

General procedure A or B affords **2t** in 85% yield (dr 1:1), 243 mg as a yellow oil after purification by silica gel column chromatography (Hex: EA; 2:1). ^{1}H NMR (400 MHz, $CDCl_3$) δ 7.19 – 7.15 (m, 1H), 6.96 – 6.94 (m, 1H), 6.90 – 6.80 (m, 3H), 6.11 – 6.07 (m, 1H), 5.75 – 5.70 (m, 1H), 3.77 (s, 3H), 3.42 (dd, *J* = 36.2, 6.7 Hz, 1H), 2.59 – 2.22 (m, 3H), 2.07 – 1.85 (m, 1H), 1.52 (s, 3H). ^{13}C{^{1}H} NMR (101 MHz, $CDCl_3$) δ 198.3, 198.1, 159.2, 146.1, 144.8, 129.2, 126.9, 126.5, 124.7, 124.0, 116.3, 114.2, 89.6, 89.3, 87.8, 87.7, 80.9, 80.8, 55.2, 37.5, 36.7, 25.8, 21.7, 14.0, 13.8. HRMS (ESI-Orbitrap) *m/z*: [M-OH]$^{+}$ Calcd for $C_{17}H_{17}O_3^{+}$ 269.1172; Found: 269.1172.

Compound **2u**

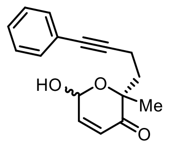

General procedure A or B affords **2u** in 89% yield (dr 1:1), 228 mg as an amber oil after purification by silica gel column chromatography (Hex: EA; 2:1). ^{1}H NMR (500 MHz, CHLOROFORM-*D*) δ 7.36 – 7.34 (m, 2H), 7.27 – 7.22 (m, 3H), 6.88 – 6.83 (m, 1H), 6.11 – 6.06 (m, 1H), 5.74 – 5.67 (m, 1H), 2.58 – 2.38 (m, 2H), 2.40 – 2.20 (m, 1H), 2.01 – 1.86 (m, 1H), 1.51 (s, 3H). ^{13}C{^{1}H} NMR (126 MHz, CHLOROFORM-*D*) δ 198.5, 198.3, 146.3, 145.0, 131.6, 128.3, 128.3, 127.8, 127.7, 127.1, 126.7, 123.8, 123.8, 89.8, 89.5, 87.9, 87.8, 81.2, 81.2, 81.1, 81.0, 60.6, 37.6, 36.9, 26.0, 21.9, 21.2, 14.3, 14.1, 13.9. HRMS (ESI-Orbitrap) *m/z*: $[M\text{-}OH]^+$ Calcd for $C_{16}H_{15}O_2^+$ 239.1066; Found: 239.1066.

Compound **2v**

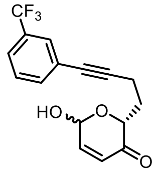

General procedure A or B affords **2v** in 97% yield (dr 1.5:1), 301 mg as an amber oil after purification by silica gel column chromatography (Hex: EA; 2:1). ^{1}H NMR (400 MHz, $CDCl_3$) δ 7.63 (s, 1H), 7.52 (t, *J* = 9.6 Hz, 2H), 7.41 – 7.37 (m, 1H), 6.96 – 6.89 (m, 1H), 6.19 – 6.11 (m, 1H), 5.71 – 5.67 (m, 1H), 4.80 – 4.77 (dd, *J* = 8.5, 3.9 Hz, 1H), 3.17 (s, 1H), 2.65 – 2.56 (m, 2H), 2.33 – 2.24 (m, 1H), 2.10 – 1.94 (m, 1H). ^{13}C{^{1}H} NMR (101 MHz, $CDCl_3$) δ 195.9, 147.6, 144.2, 134.6, 128.7, 128.3, 127.5, 124.6, 124.2, 90.9, 90.6, 87.7, 72.3, 29.5, 28.5, 15.2, 15.0. HRMS (ESI-Orbitrap) *m/z*: $[M\text{-}OH]^+$ Calcd for $C_{16}H_{12}F_3O_2^+$ 293.0784; Found: 293.0776.

Compound **2w**

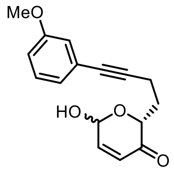

General procedure A or B affords **2w** in 92% yield (dr 2:1), 251 mg as an amber oil after purification by silica gel column chromatography (Hex: EA; 2:1). ^{1}H NMR (400 MHz, $CDCl_3$) δ 7.22 – 7.14 (m, 1H), 6.99 – 6.82 (m, 4H), 6.15 (dd, *J* = 19.9, 10.3 Hz, 1H), 5.72 – 5.67 (m, 1H), 4.81 (dd, *J* = 8.7, 3.8 Hz, 1H), 3.78 (s, 1H), 2.85 (d, *J* = 4.9 Hz, 1H), 2.65 – 2.56 (m, 2H), 2.34 – 2.25 (m, 1H), 1.99 – 1.92 (m, 1H). ^{13}C{^{1}H} NMR (126 MHz, $CDCl_3$) δ 196.9, 196.6, 160.0, 148.5, 145.0, 133.6, 130.0, 129.5, 128.3, 125.4, 125.4, 124.9, 117.3, 117.2, 115.1, 115.0, 91.7, 89.4, 89.3, 88.5, 82.3, 82.2, 73.2, 56.0, 30.4, 29.4, 16.0, 15.9. HRMS (ESI-Orbitrap) *m/z*: $[M\text{-}OH]^+$ Calcd for $C_{16}H_{15}O_3^+$ 255.1016; Found: 255.1016.

Compound **2x**

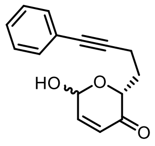

General procedure A or B affords **2x** in 99% yield (dr 2:1), 240 mg as an amber oil after purification by silica gel column chromatography (Hex: EA; 2:1). ^{1}H NMR (400 MHz, $CDCl_3$) δ 7.39 – 7.36 (m, 2H), 7.28 – 7.24 (m, 3H), 6.94 – 6.86 (m, 1H), 6.18 – 6.10 (m, 1H), 5.69 – 5.66 (m, 1H), 4.81 (dd, *J* = 8.6, 3.8 Hz, 1H), 3.17 (s, 1H), 2.64 – 2.56 (m, 2H), 2.33 – 2.24 (m, 1H), 2.08 – 1.92 (m, 1H). ^{13}C{^{1}H} NMR (101 MHz, $CDCl_3$) δ 147.6, 144.1, 131.5, 128.7, 128.2, 127.7, 127.6, 90.9, 88.7, 87.7, 81.6, 72.4, 29.6, 28.6, 15.3, 15.1. HRMS (ESI-Orbitrap) *m/z*: $[M\text{-}OH]^+$ Calcd for $C_{15}H_{13}O_2^+$ 225.0910; Found: 225.0910.

General method C for Achmatowicz products **2q and 2r:**

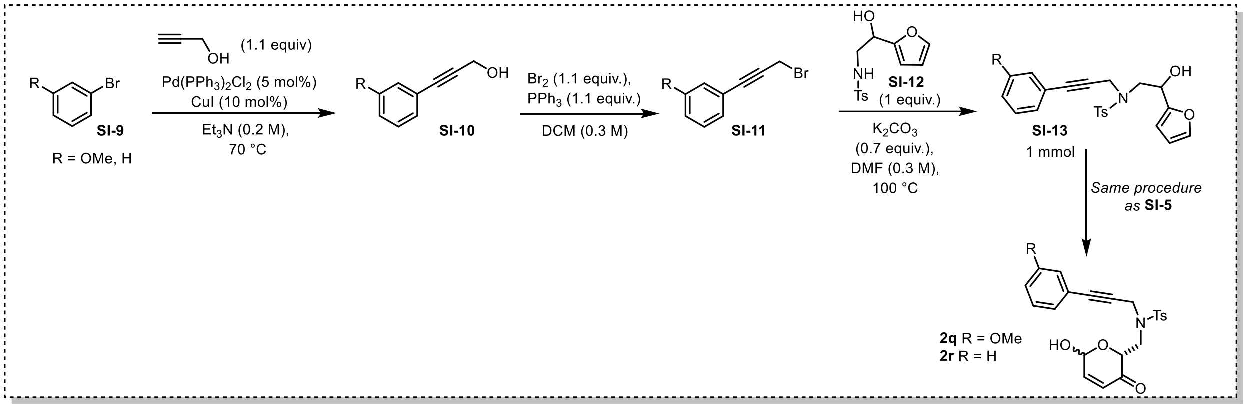

Scheme S3. Procedure C for synthesizing Achmatowicz adduct **2q** and **2v**.

$Pd(PPh_3)_2Cl_2$ (5 mol%) and CuI (10 mol%) were added to a round-bottom flask. The flask was evacuated using a vacuum pump and flushed with argon three times. Et_3N was then added under an argon atmosphere, followed by the addition of the aryl halide (1 equiv.) and propargyl alcohol (1.1 equiv.). The reaction mixture was stirred at 70°C, and the progress was monitored by TLC. After completion, the crude mixture was filtered through Celite, the solid residue was washed with Et_2O, and the combined organic phases were concentrated under reduced pressure. Purification by flash column chromatography (Hex: EA; 2:1) provided the desired aryl propargyl alcohols **SI-10**. The NMR data is consistent with the literature.[5,6]

To a solution of PPh_3 (1.1 equiv.) in DCM (0.3 M) at 0°C under air, Br_2 (1.1 equiv.) was added dropwise. The reaction mixture was stirred at 0°C for 30 minutes, gradually forming a yellow slurry. A solution of alcohol **SI-10** (1 equiv.) in DCM (0.5 M) was then added dropwise. The clear yellow solution formed was stirred at 0°C for 1 hour. After confirming completion by TLC, hexane (0.3 M) was added, causing a precipitate (triphenylphosphine oxide) to form immediately. The mixture was stirred for an additional 30 minutes at room temperature. The solids were filtered and thoroughly washed with hexane (0.4 M). The filtrate was concentrated, and the residue was purified by silica gel column chromatography (Hex : EA; 4:1) to yield **SI-11**. The NMR data is consistent with the literature.[7,8]

A mixture of **SI-12** (1 equiv.), prepared following the literature procedure,[9] **SI-11** (1 equiv.), and K_2CO_3 (0.7 equiv.) in DMF (0.3 M) were stirred at 100°C. Once the reaction was complete according to TLC, it was quenched with water and extracted with Et_2O. The organic layer was washed with additional water and brine, dried over $MgSO_4$, and concentrated. The resulting residue was purified by silica gel chromatography (Hex: EA; 2:1).

Compound **SI-13a**

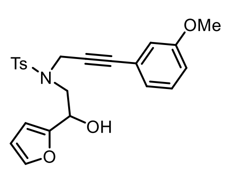

Purification by silica gel column chromatography (Hex: EA; 2:1) affords the title compound as a yellow oil. ^1H NMR (500 MHz, $CDCl_3$) δ 7.78 (d, J = 8.3 Hz, 2H), 7.37 – 7.36 (m, 1H), 7.26 – 7.24 (m, 2H), 7.13 (t, J = 8.0 Hz, 1H), 6.83 – 6.80 (m, 1H), 6.67 – 6.65 (m, 1H), 6.60 – 6.59 (m, 1H), 6.37 – 6.30 (m, 2H), 5.02 (dd, J = 8.3, 4.0 Hz, 1H), 4.45 – 4.21 (m, 2H), 3.75 (s, 3H), 3.63 – 3.50 (m, 2H), 2.15 (s, 3H). $^{13}C\{^1H\}$ NMR (126 MHz, CHLOROFORM-*D*) δ 159.3, 153.7, 144.1, 142.5, 135.6, 129.8, 129.3, 128.0, 124.1, 123.1, 117.0, 114.7, 110.6, 107.5, 85.7, 81.9, 66.8, 55.4, 51.5, 39.5, 21.5. HRMS (ESI-Orbitrap) *m/z*: $[M-OH]^+$ Calcd for $C_{23}H_{22}NO_4S^+$ 408.1264; Found: 408.1263.

Compound **SI-13b**

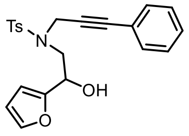

Purification by silica gel column chromatography (Hex: EA; 2:1) affords the title compound as a yellow oil. ^{1}H NMR (500 MHz, CHLOROFORM-*D*) δ 7.79 (d, *J* = 8.3 Hz, 1H), 7.38 – 7.25 (m, 9H), 6.35 – 6.34 (m, 1H), 5.18 – 5.06 (m, 1H), 4.63 – 4.54 (m, 2H), 3.67 (dd, *J* = 15.2, 9.3 Hz, 1H), 3.53 – 3.46 (m, 1H), 3.29 (d, *J* = 3.7 Hz, 1H), 2.42 (s, 3H). $^{13}C\{^1H\}$ NMR (126 MHz, CHLOROFORM-*D*) δ 153.7, 144.2, 142.5, 140.1, 135.9, 130.0, 129.3, 128.7, 128.5, 127.8, 121.3, 118.4, 110.5, 107.3, 67.0, 56.9, 54.2, 21.7. HRMS (ESI-Orbitrap) *m/z*: $[M-OH]^+$ Calcd for $C_{22}H_{20}NO_3S^+$ 378.1158; Found: 378.1157.

Compound **2q**

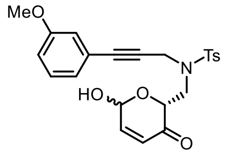

General procedure C affords **1q** in 76% yield (dr 2:1), 336 mg as yellow oil after purification by silica gel column chromatography (Hex: EA; 2:1). ^{1}H NMR (400 MHz, CHLOROFORM-*D*) δ 7.79 – 7.77 (m, 2H), 7.25 – 7.23 (m, 2H), 7.13 – 7.12 (m, 1H), 7.01 – 6.91 (m, 1H), 6.82 – 6.79 (m, 1H), 6.66 – 6.58 (m, 2H), 6.16 – 6.07 (m, 1H), 5.75 – 5.69 (m, 1H), 4.98 – 4.95 (m 1H), 4.56 – 4.42 (m, 3H), 3.98 – 3.82 (m, 1H), 3.74 (s, 2H), 3.63 – 3.45 (m, 1H), 2.32 (s, 3H). $^{13}C\{^1H\}$ NMR (101 MHz, CHLOROFORM-*D*) δ 194.4, 159.2, 145.3, 144.0, 143.9, 135.8, 129.8, 129.7, 129.3, 128.3, 128.0, 127.2, 124.1, 123.2, 116.9, 114.7, 114.7, 90.6, 87.7, 85.7, 82.1, 78.3, 73.6, 55.4, 47.9, 46.5, 39.5, 39.4, 21.5. HRMS (ESI-Orbitrap) *m/z*: $[M-OH]^+$ Calcd for $C_{23}H_{22}NO_5S^+$ 424.1213; Found: 424.1213.

Compound **2r**

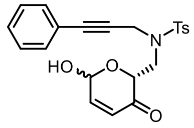

General procedure C affords **1r** in 77% yield (dr 2:1), 317 mg as yellow oil after purification by silica gel column chromatography (Hex: EA; 2:1). ^{1}H NMR (400 MHz, CHLOROFORM-*D*) δ 7.79 (dd, *J* = 8.3, 3.2 Hz, 2H), 7.36 – 7.18 (m, 7H), 7.08 – 7.03 (m, 1H), 7.01 – 6.88 (m, 1H), 6.18 – 6.09 (m, 1H), 5.75 – 5.69 (m, 1H), 4.97 (dd, *J* = 9.2, 3.0 Hz, 1H), 4.55 – 4.41 (m, 1H), 3.98 – 3.77 (m, 1H), 3.67 – 3.46 (m, 1H), 2.32 (s, 3H). $^{13}C\{^1H\}$ NMR (101 MHz, CHLOROFORM-*D*) δ 194.2, 193.9, 147.9, 144.9, 143.9, 143.8, 135.9, 135.7, 131.6, 131.6, 129.7, 129.7, 128.6, 128.2, 128.0, 127.9, 127.4, 122.2, 90.4, 87.7, 85.8, 82.3, 82.1, 78.2, 73.6, 48.1, 46.6, 39.5, 21.5. HRMS (ESI-Orbitrap) *m/z*: $[M-OH]^+$ Calcd for $C_{23}H_{20}NO_4S^+$ 394.1107; Found: 394.1108.

Supplementary tables for experiment

Table S1. Reaction optimization for the formation of vinyl halide.

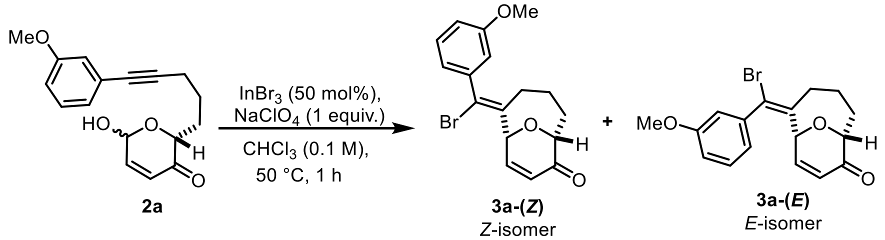

Entry	Deviation	3a (*Z*:*E*)
**1**	none	59% (2.5:1)
**2**	room temp. instead of 50 °C	46% (2.6:1)
**3**	DBE + without $NaClO_4$	30% (11:1)[b]

**4**	DCE + without $NaClO_4$ + rt	30% (1.4:1)[b]
**5**	DCE instead of DCM + $LiClO_4$	32% (1.2:1)
**6**	NBu_4PF_6 instead of $NaClO_4$	19% (1.2:1)
**7**	$B(C_6F_5)_3$ + TBAB + without $NaClO_4$ + room temp.	-[c]
**8**	TMSBr (3 equiv.), without $NaClO_4$ + room temp.	-[c]
**9**	5Å MS + without $NaClO_4$	17% (4:1)

[a]0.2 mmol of **2a**, isolated yield; [b]less than 10% isolated yield of **4a**; [c]product not observed; The ratio in parentheses is the ratio of **3a-(*Z*):3a-(*E*)** determined by ^{1}H NMR analysis.

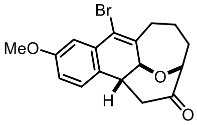

Tetracycle **4a** (minor product)

NOESY Experiment of Identifying the Major Isomer

According to the NOESY data, no cross peak was observed between any of the aromatic protons with the enone protons (*f*, *g*) and allylic proton (*e*). However, we did see cross-peaks between the aromatic protons (*a* and *d*) with the alkyl protons (*n* and *m*). This data shows that the major isomer is **3a-(*Z*)**, which is unexpected because it is uncommon for there to be syn-addition to the alkyne; typically, there is high selectivity for anti-addition.[10]

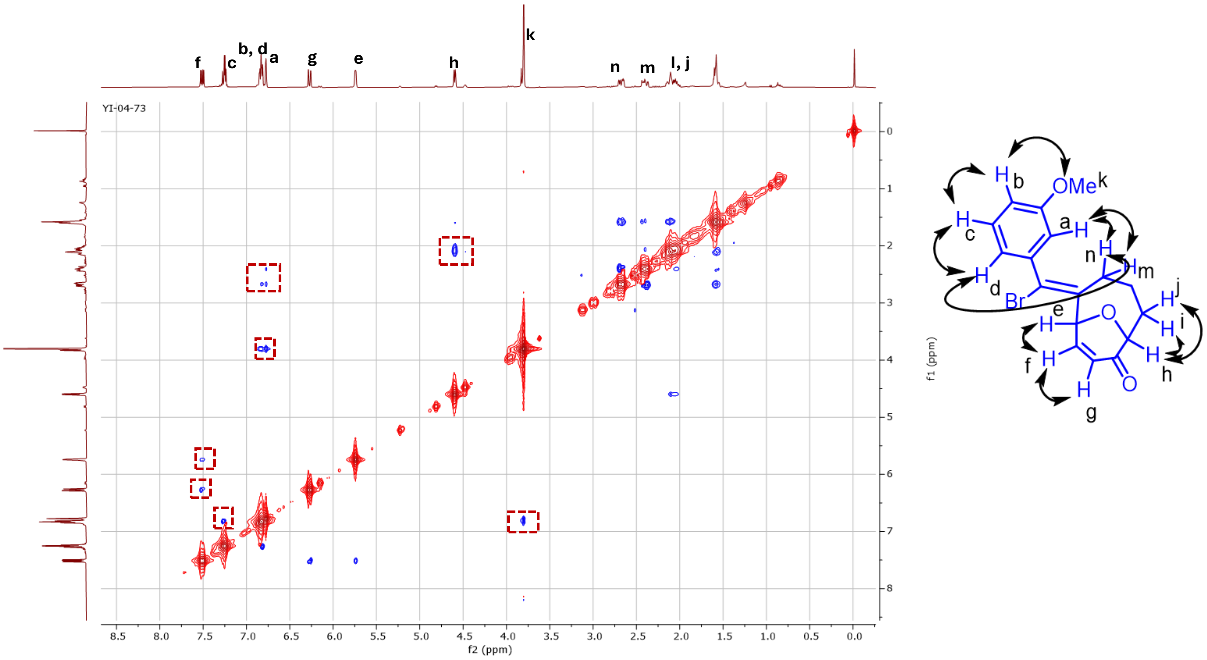

Figure 1. NOESY experiment of the major isomer.

Assigning stereochemistry for **7i**

Stereochemistry was assigned using 2D NOESY experiments and a comparison to the literature[11,12]

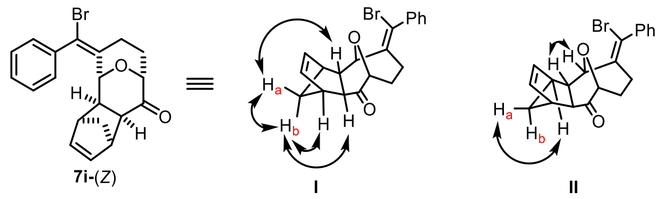

Formation of bridged oxacycles **10**

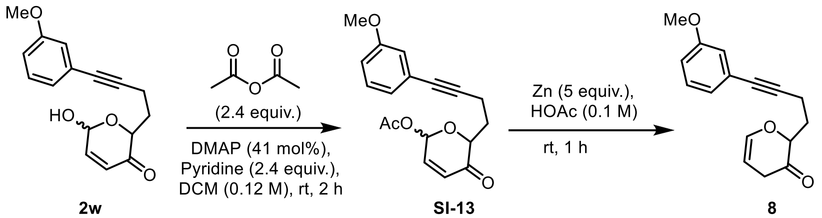

Scheme S4. Synthesis of precursor **8**

To a solution of **2w** (1 equiv.) in DCM (0.12 M) was added 4-dimethylaminopyridine (41 mol%), pyridine (2.4 equiv.) and acetic anhydride (2.4 equiv.). The reaction was stirred at room temperature until full consumption of **2w** according to TLC. *Quenching:* The reaction mixture was quenched with sat. NH_4Cl, transferred to a separatory funnel, washed with water, and brine, and dried over $MgSO_4$ (filtered through a celite pad). The filtrate was concentrated using a rotary evaporator and further purified by silica gel column chromatography (Hex: EA; 4:1) to afford **SI-13** as a yellow oil.

The procedure in the literature was used to prepare **8** with little modification.[13] To a stirred solution of **SI-13** (3 mmol) in acetic acid (0.1 M) was added freshly prepared activated Zinc dust (5 equiv.) at room temperature. The reaction mixture was stirred until completion according to TLC (~1 h). The reaction was then cooled to 0 °C and quenched by the addition of sat. aqueous K_2CO_3 and extracted with DCM. The combined organic layers were washed with brine, dried over $MgSO_4$, and concentrated by rotary evaporator. The resulting residue was purified by silica gel column chromatography (Hex: EA; 4:1) to afford 361 mg (47% yield) of **8** as a colorless oil.

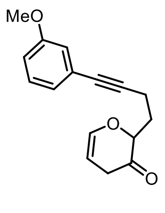

^{1}H NMR (500 MHz, $CDCl_3$) δ 7.16 (t, J = 7.9 Hz, 1H), 7.03 – 6.94 (m, 1H), 6.90 (dd, J = 2.7, 1.5 Hz, 1H), 6.83 – 6.80 (m, 1H), 6.53 – 6.51 (m, 1H), 4.96 – 4.93 (m, 1H), 4.34 – 4.31 (m, 1H), 3.77 (s, 3H), 2.99 – 2.80 (m, 2H), 2.59 – 2.56 (m, 2H), 2.21 – 2.10 (m, 1H), 2.08 – 1.97 (m, 1H). $^{13}C\{^1H\}$ NMR (126 MHz, $CDCl_3$) δ 207.1, 160.0, 144.2, 130.0, 125.4, 124.9, 117.2, 115.1, 99.6, 89.1, 82.2, 80.4, 56.0, 35.4, 29.8, 16.0. HRMS (ESI-Orbitrap) *m/z*: $[M+H]^+$ Calcd for $C_{16}H_{17}O_3^+$ 257.1172; Found: 257.1172.

Gram-scale reaction

The procedure for the multigram reaction is the same as that of the 0.2 mmol scale for the alkynyl *halo*-Prins cyclization.

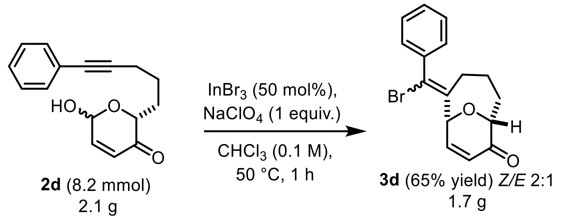

2d (8.2 mmol) 2.1 g → **3d** (65% yield) *Z/E* 2:1, 1.7 g

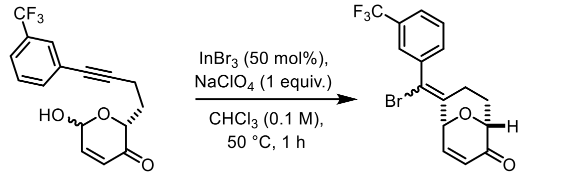

2v (3.23 mmol) 1 g → **6d** (75% yield) *Z/E* 3:1, 0.9 g

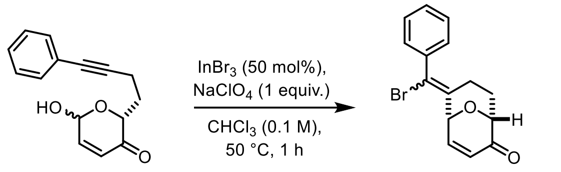

2x (9.9 mmol) 2.4 g → **6f** (61% yield) *Z/E* 1:1, 1.84 g

References

(1) Berkessel, A.; Krämer, J.; Mummy, F.; Neudörfl, J.-M.; Haag, R. Dendritic Fluoroalcohols as Catalysts for Alkene Epoxidation with Hydrogen Peroxide. *Angew. Chem. Int. Ed.* **2013**, *52, 739*–743.

(2) Bodinier, F.; Lannou, M.-I.; Sorin, G. Low-Valent Titanium (Ii) Mediated Intramolecular and Regioselective Alkyne/Alkoxyallene Reductive Coupling Reactions. *Chem. Commun.,* **2024**, *60*, 1786–1789.

(3) Alachouzos, G.; Holt, C.; Frontier, A. J. Stereochemical Relay through a Cationic Intermediate: Helical Preorganization Dictates Direction of Conrotation in the *Halo* -Nazarov Cyclization. *Org. Lett.* **2020**, *22*, 4010–4015.

(4) Honda, T.; Hoshi, M.; Kanai, K.; Tsubuki, M. Enantioselective Synthesis of Indolizidine Alkaloids: Formal Synthesis of (–)-Swainsonine and of (+)-Pumiliotoxin 251D. *J. Chem. Soc. Perkin Trans. 1* **1994**, 2091–2101.

(5) Panteleev, J.; Huang, R. Y.; Lui, E. K.; Lautens, M. Addition of Arylboronic Acids to Arylpropargyl Alcohols En Route to Indenes and Quinolines. *Org. Lett.* **2011**, *13*, 5314–5317.

(6) Fish, I.; Stößel, A.; Eitel, K.; Valant, C.; Albold, S.; Huebner, H.; Möller, D.; Clark, M. J.; Sunahara, R. K.; Christopoulos, A. Structure-Based Design and Discovery of New M2 Receptor Agonists. *J. Med. Chem.* **2017**, *60*, 9239–9250.

(7) Kleinbeck, F.; Toste, F. D. Gold (I)-Catalyzed Enantioselective Ring Expansion of Allenylcyclopropanols. *J. Am. Chem. Soc.* **2009**, *131*, 9178–9179.

(8) Domingo-Legarda, P.; Soler-Yanes, R.; Quirós-López, M. T.; Buñuel, E.; Cárdenas, D. J. Iron-catalyzed Coupling of Propargyl Bromides and Alkyl Grignard Reagents. *Eur. J. Org. Chem.* **2018**, *2018*, 4900–4904.
(9) van der Pijl, F.; van Delft, F. L.; Rutjes, F. P. The Aza-Achmatowicz Reaction: Facile Entry into Functionalized Piperidinones. *Eur. J. Org. Chem.* **2015**, *22*, 4811–4829.
(10) Holt, C.; Alachouzos, G.; Frontier, A. J. Leveraging the Halo-Nazarov Cyclization for the Chemodivergent Assembly of Functionalized Haloindenes and Indanones. *J. Am. Chem. Soc.* **2019**, *141*, 5461–5469.
(11) McLeod, D.; Cherubini-Celli, A.; Sivasothirajah, N.; McCulley, C. H.; Christensen, M. L.; Jørgensen, K. A. Enantioselective 1,3-Dipolar [6+4] Cycloaddition of Pyrylium Ions and Fulvenes towards Cyclooctanoids. *Chem. Eur. J.* **2020**, *26*, 11417–11422.
(12) Nair, V.; Anilkumar, G.; Sujatha, T. S.; SomarajanNair, J. $ZnCl_2$ Catalysed Diels-Alder Reactions of Oxa and Aza Bicyclo[3.2.1]Systems with Dienes: Formation of Some Novel Heterocyclic Systems. *Synth. Commun.*, **1998**, *28*, 2549–2557.
(13) Li, Z.; Ip, F. C.; Ip, N. Y.; Tong, R. Highly trans-Selective Arylation of Achmatowicz Rearrangement Products by Reductive γ-Deoxygenation and Heck–Matsuda Reaction: Asymmetric Total Synthesis of (−)-Musellarins A–C and Their Analogues. *Chem. Eur. J.* **2015**, *21*, 11152–11157.

X-ray data

Sample preparation for compounds **3k**, **7e**, **10**: crystals were obtained by dissolving the appropriate compound in a 10:1 mixture of hexane and DCM, heating the solution to a boil, and then allowing it to slowly cool to room temperature overnight to yield crystals suitable for X-ray analysis.

<u>Compound **3k** (CCDC: 2388485):</u>

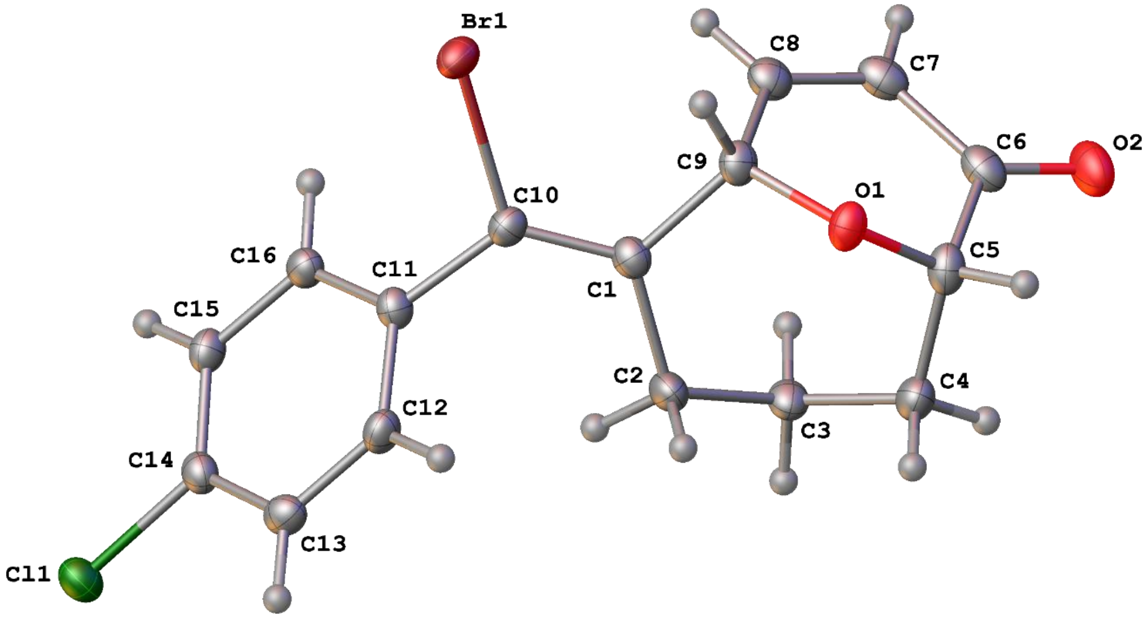

Crystal data and structure refinement for **3k** (major isomer). Ellipsoids drawn at the 50% probability level.

Identification code	**3k-(*Z*)**	
Empirical formula	C16 H14 Br Cl O2	
Formula weight	353.63	
Temperature	100.00(10) K	
Wavelength	1.54184 Å	
Crystal system	orthorhombic	
Space group	$Pna2_1$	
Unit cell dimensions	*a* = 11.31820(10) Å	α = 90°
	b = 13.97210(10) Å	β = 90°
	c = 8.87750(10) Å	γ = 90°
Volume	1403.88(2) $Å^3$	
Z	4	
Density (calculated)	1.673 Mg/m^3	
Absorption coefficient	5.726 mm^{-1}	
F(000)	712	
Crystal color, morphology	colourless, needle	
Crystal size	0.295 x 0.056 x 0.049 mm^3	
Theta range for data collection	5.029 to 80.174°	
Index ranges	$-14 \leq h \leq 14, -17 \leq k \leq 17, -10 \leq l \leq 11$	
Reflections collected	28700	
Independent reflections	2985 [*R*(int) = 0.0490]	
Observed reflections	2962	
Completeness to theta = 74.504°	100.0%	
Absorption correction	Multi-scan	
Max. and min. transmission	1.00000 and 0.60028	
Refinement method	Full-matrix least-squares on F^2	
Data / restraints / parameters	2985 / 1 / 181	
Goodness-of-fit on F^2	1.142	
Final *R* indices [*I*>2sigma(*I*)]	*R*1 = 0.0305, *wR*2 = 0.0804	
R indices (all data)	*R*1 = 0.0308, *wR*2 = 0.0807	
Absolute structure parameter	0.003(17)	
Largest diff. peak and hole	0.439 and -0.649 e.$Å^{-3}$	

Compound **7e** (CCDC: 2388484):

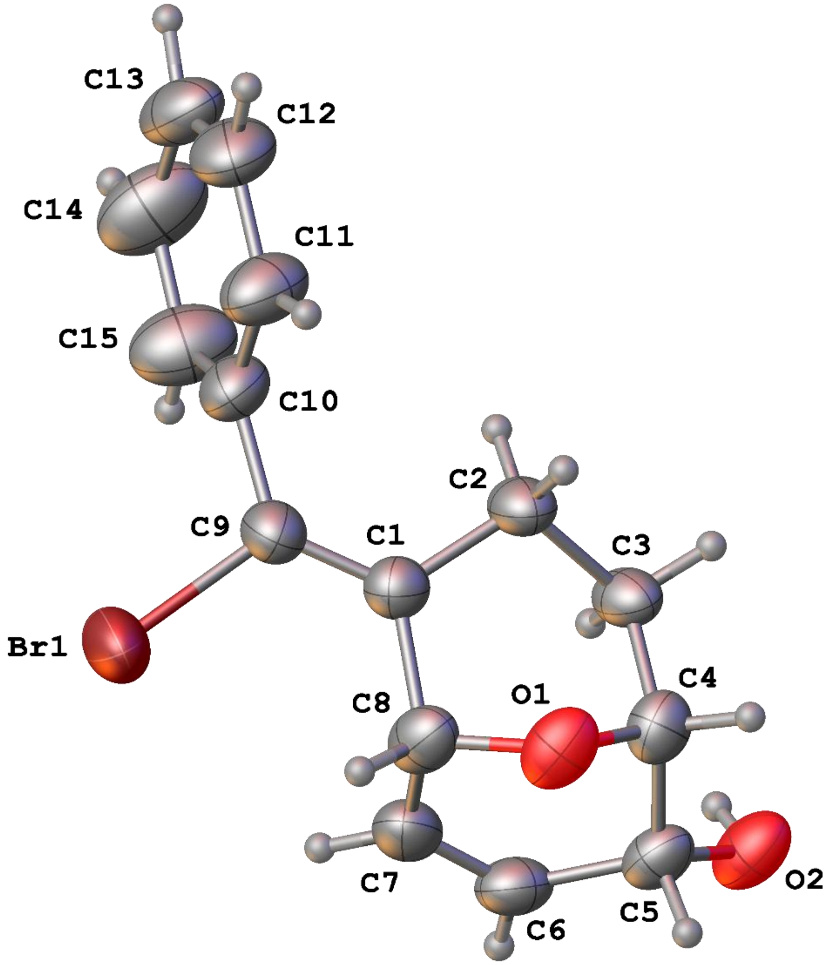

Crystal data and structure refinement for **7e** (*Z*-isomer). Ellipsoids drawn at the 50% probability level.

Identification code	**7e-(*Z*)**	
Empirical formula	C15 H15 Br O2	
Formula weight	307.18	
Temperature	293 K	
Wavelength	1.54184 Å	
Crystal system	monoclinic	
Space group	$P2_1/c$	
Unit cell dimensions	*a* = 5.24160(10) Å	α = 90°

	b = 22.6729(3) Å	β = 98.833(2)°
	c = 11.4289(2) Å	γ = 90°
Volume	1342.13(4) Å^3	
Z	4	
Density (calculated)	1.520 Mg/m^3	
Absorption coefficient	4.103 mm^{-1}	
F(000)	624	
Crystal color, morphology	yellow, plate	
Crystal size	0.111 x 0.049 x 0.026 mm^3	
Theta range for data collection	3.899 to 80.118°	
Index ranges	$-6 \le h \le 5$, $-26 \le k \le 28$, $-14 \le l \le 14$	
Reflections collected	22988	
Independent reflections	2889 [R(int) = 0.0354]	
Observed reflections	2371	
Completeness to theta = 67.684°	100.0%	
Absorption correction	Multi-scan	
Max. and min. transmission	1.00000 and 0.78794	
Refinement method	Full-matrix least-squares on F^2	
Data / restraints / parameters	2889 / 79 / 259	
Goodness-of-fit on F^2	1.049	
Final R indices [I>2sigma(I)]	R1 = 0.0330, wR2 = 0.0858	
R indices (all data)	R1 = 0.0407, wR2 = 0.0905	
Largest diff. peak and hole	0.300 and -0.400 e.Å^{-3}	

Compound **10** (CCDC: 2388459):

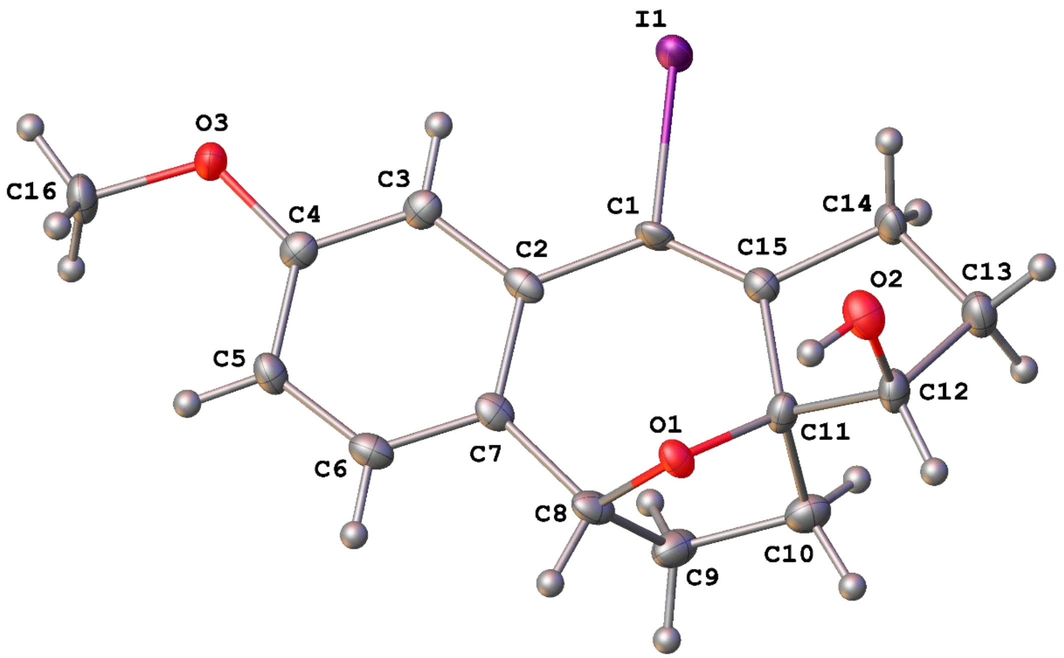

Crystal data and structure refinement for **8**. Ellipsoids drawn at the 50% probability level.

Identification code	**10**	
Empirical formula	C16 H17 I O3	
Formula weight	384.19	
Temperature	100.01(10) K	
Wavelength	1.54184 Å	
Crystal system	orthorhombic	
Space group	$Pna2_1$	
Unit cell dimensions	a = 10.87930(10) Å	α = 90°
	b = 12.57020(10) Å	β = 90°
	c = 10.37280(10) Å	γ = 90°
Volume	1418.53(2) Å^3	
Z	4	
Density (calculated)	1.799 Mg/m^3	
Absorption coefficient	17.782 mm^{-1}	
F(000)	760	
Crystal color, morphology	colourless, block	
Crystal size	0.177 x 0.078 x 0.06 mm^3	

Theta range for data collection	5.377 to 80.100°
Index ranges	$-13 \leq h \leq 13$, $-15 \leq k \leq 16$, $-12 \leq l \leq 12$
Reflections collected	23238
Independent reflections	2902 [R(int) = 0.0594]
Observed reflections	2857
Completeness to theta = 74.504°	100.0%
Absorption correction	Multi-scan
Max. and min. transmission	1.00000 and 0.34445
Refinement method	Full-matrix least-squares on F^2
Data / restraints / parameters	2902 / 1 / 186
Goodness-of-fit on F^2	1.144
Final R indices [I>2sigma(I)]	$R1$ = 0.0294, $wR2$ = 0.0816
R indices (all data)	$R1$ = 0.0299, $wR2$ = 0.0820
Absolute structure parameter	-0.011(6)
Largest diff. peak and hole	0.564 and -0.985 e.Å^{-3}

NMR Data

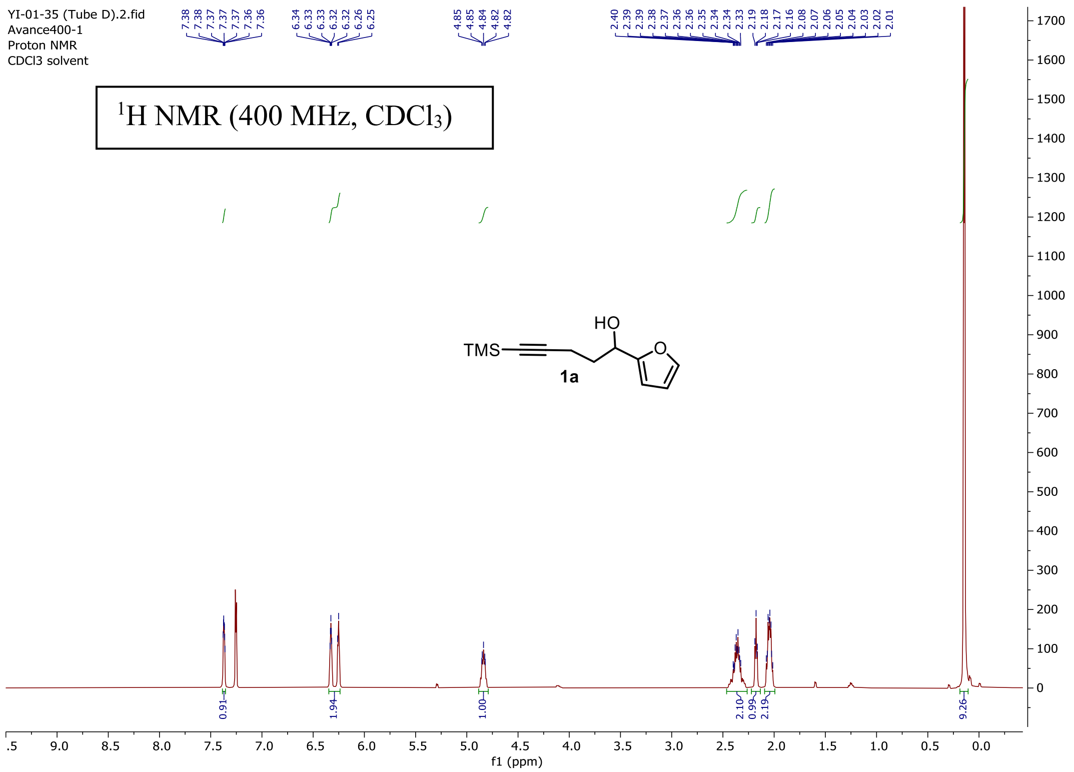

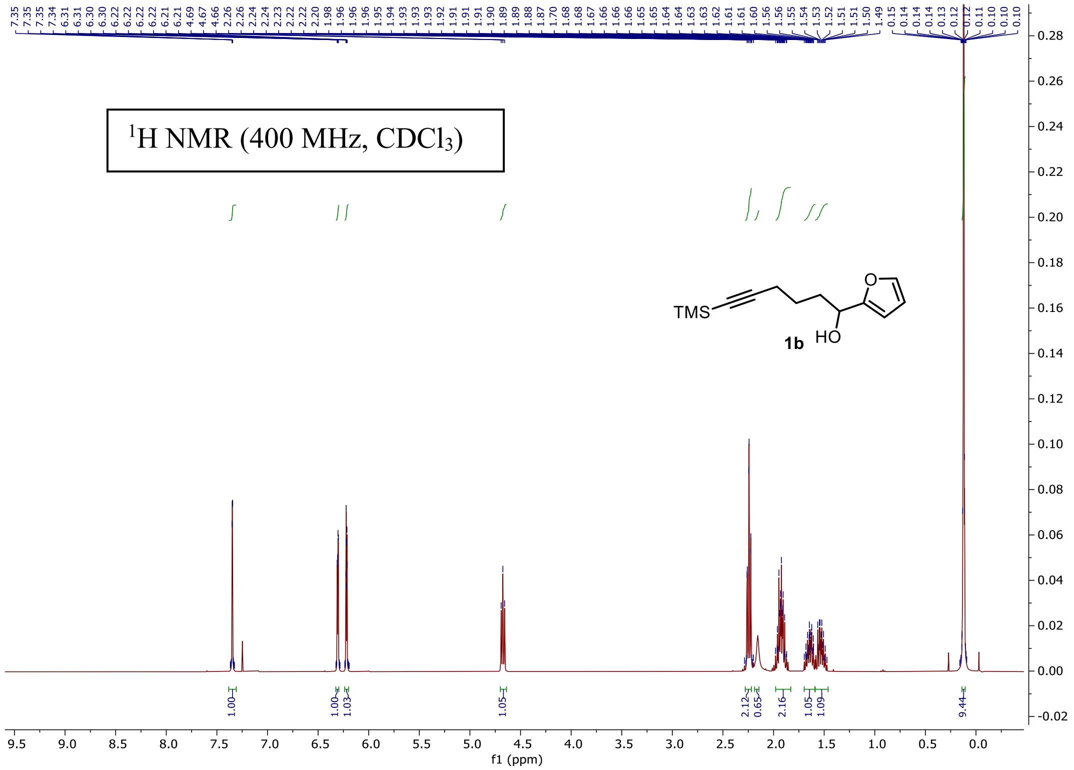

1H NMR (400 MHz, CDCl3)
TMS
1b
HO
O
f1 (ppm)

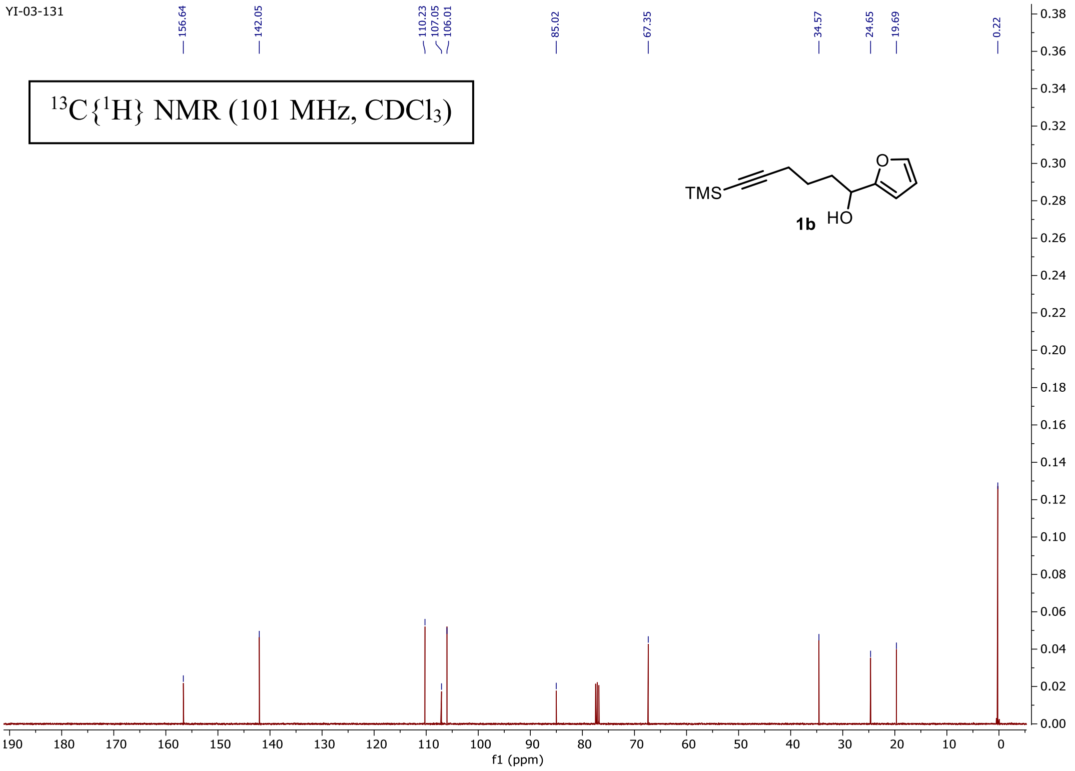

YI-03-131
13C{1H} NMR (101 MHz, CDCl3)
156.64
142.05
110.23
107.05
106.01
85.02
67.35
34.57
24.65
19.69
0.22
TMS
1b
HO
O
f1 (ppm)

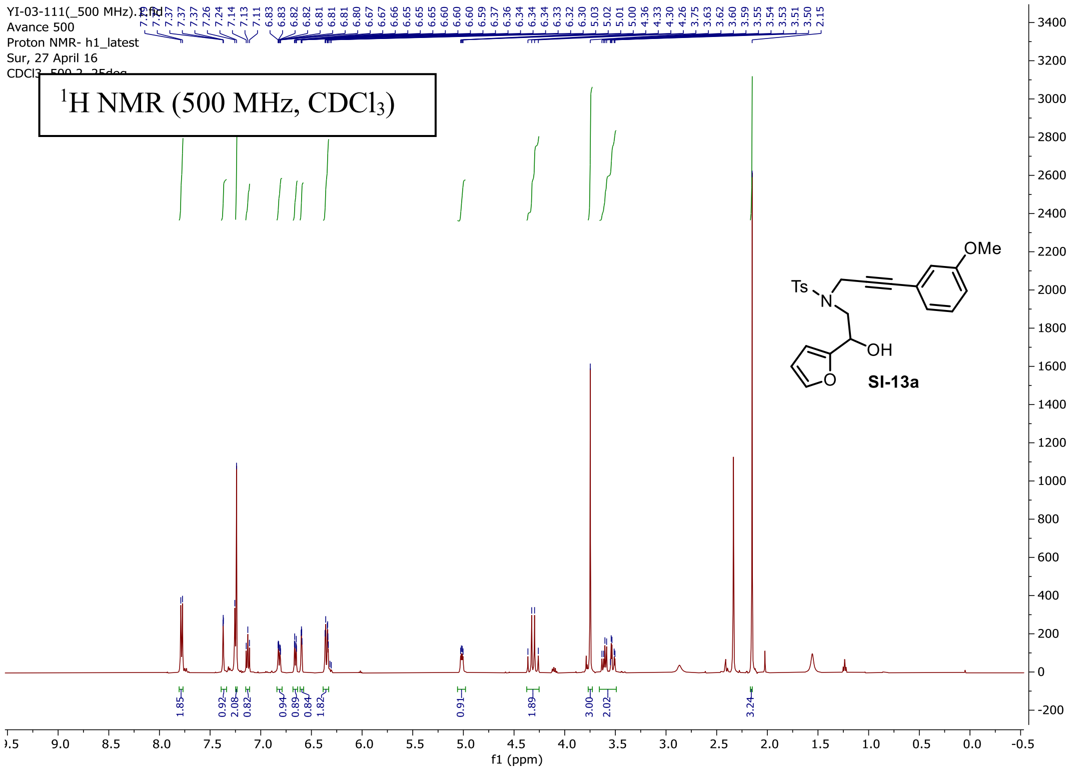
YI-03-111(_500 MHz).2.fid
Avance 500
Proton NMR- h1_latest
Sur, 27 April 16
1H NMR (500 MHz, CDCl3)
OMe
Ts
N
OH
O
SI-13a
f1 (ppm)

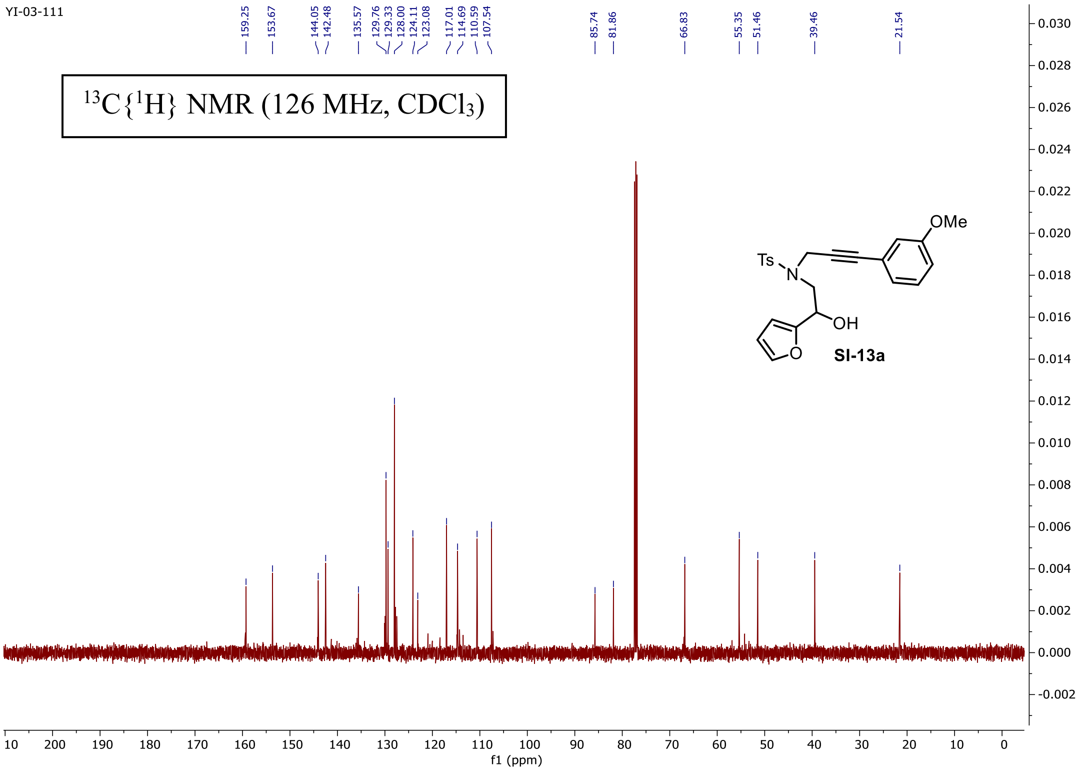
YI-03-111
159.25
153.67
144.05
142.48
135.57
129.76
129.33
128.00
124.11
123.08
117.01
114.69
110.59
107.54
85.74
81.86
66.83
55.35
51.46
39.46
21.54
13C{1H} NMR (126 MHz, CDCl3)
OMe
Ts
N
OH
O
SI-13a
f1 (ppm)

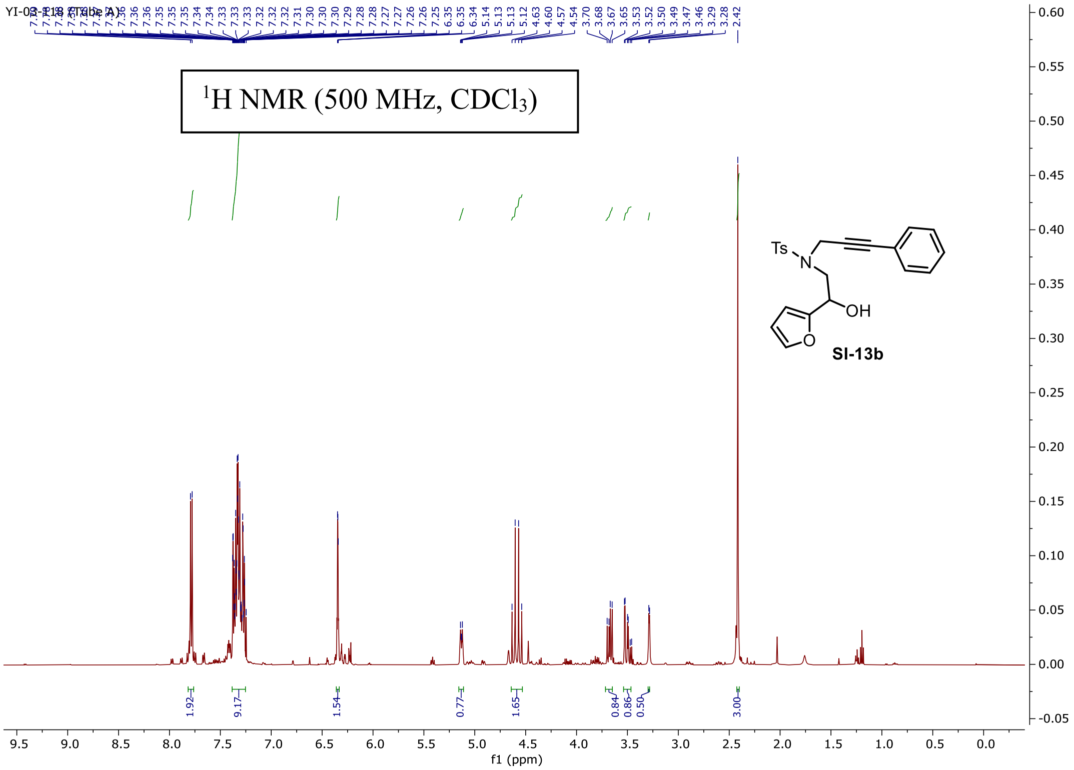
YI-03-118 (Tube A)
^{1}H NMR (500 MHz, CDCl$_3$)
Ts
N
OH
O
SI-13b
1.92
9.17
1.54
0.77
1.65
0.84
0.86
0.50
3.00
f1 (ppm)

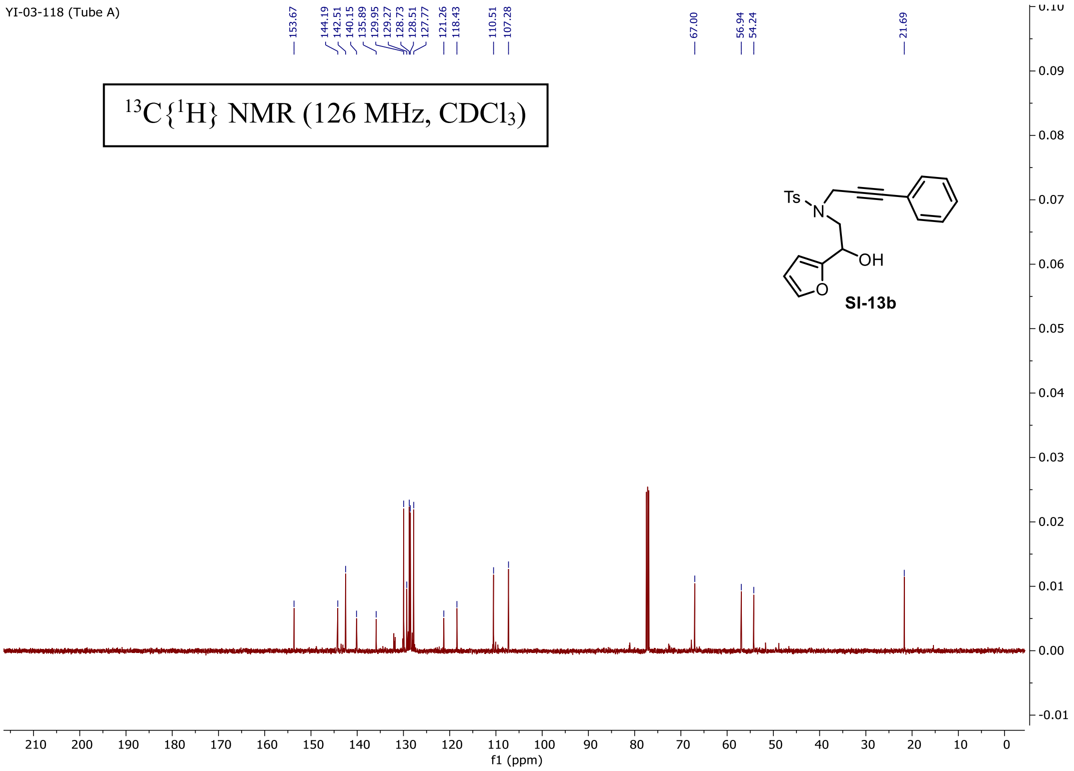
YI-03-118 (Tube A)
153.67
144.19
142.51
140.15
135.89
129.95
129.27
128.73
128.51
127.77
121.26
118.43
110.51
107.28
67.00
56.94
54.24
21.69
^{13}C{^{1}H} NMR (126 MHz, CDCl$_3$)
Ts
N
OH
O
SI-13b
f1 (ppm)

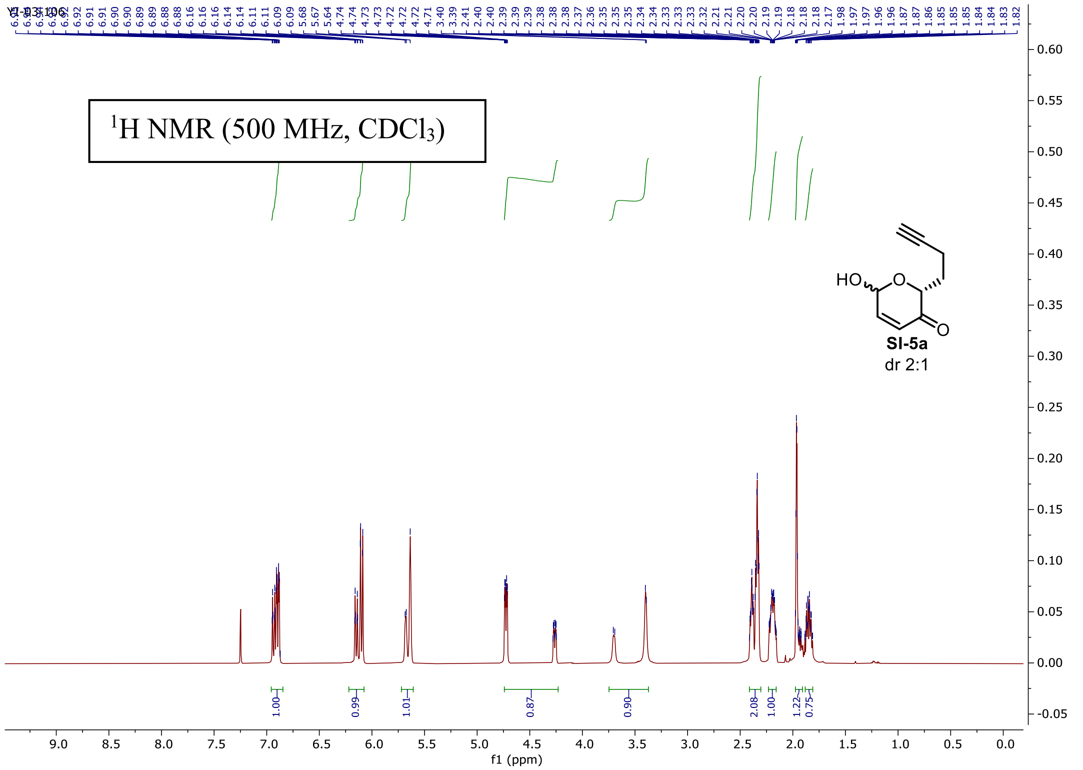

YI-03-106
^{1}H NMR (500 MHz, CDCl$_3$)
HO
O
O
SI-5a
dr 2:1
f1 (ppm)

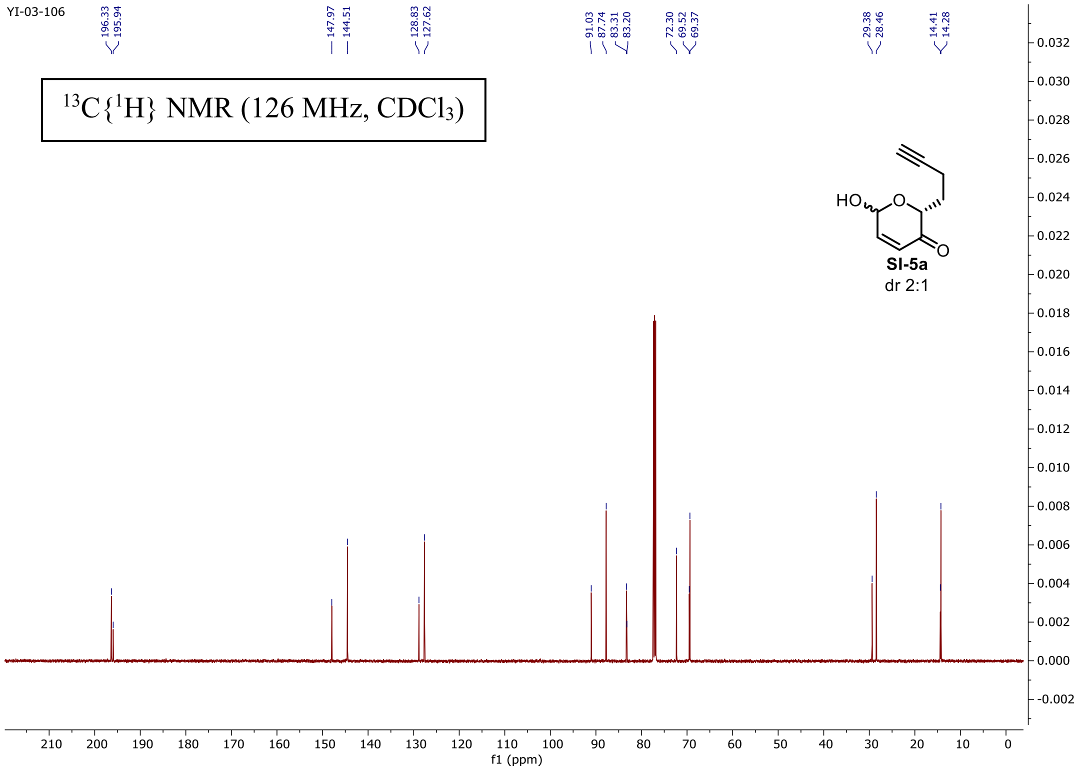

YI-03-106
196.33
195.94
147.97
144.51
128.83
127.62
91.03
87.74
83.31
83.20
72.30
69.52
69.37
29.38
28.46
14.41
14.28
^{13}C{^{1}H} NMR (126 MHz, CDCl$_3$)
HO
O
O
SI-5a
dr 2:1
f1 (ppm)

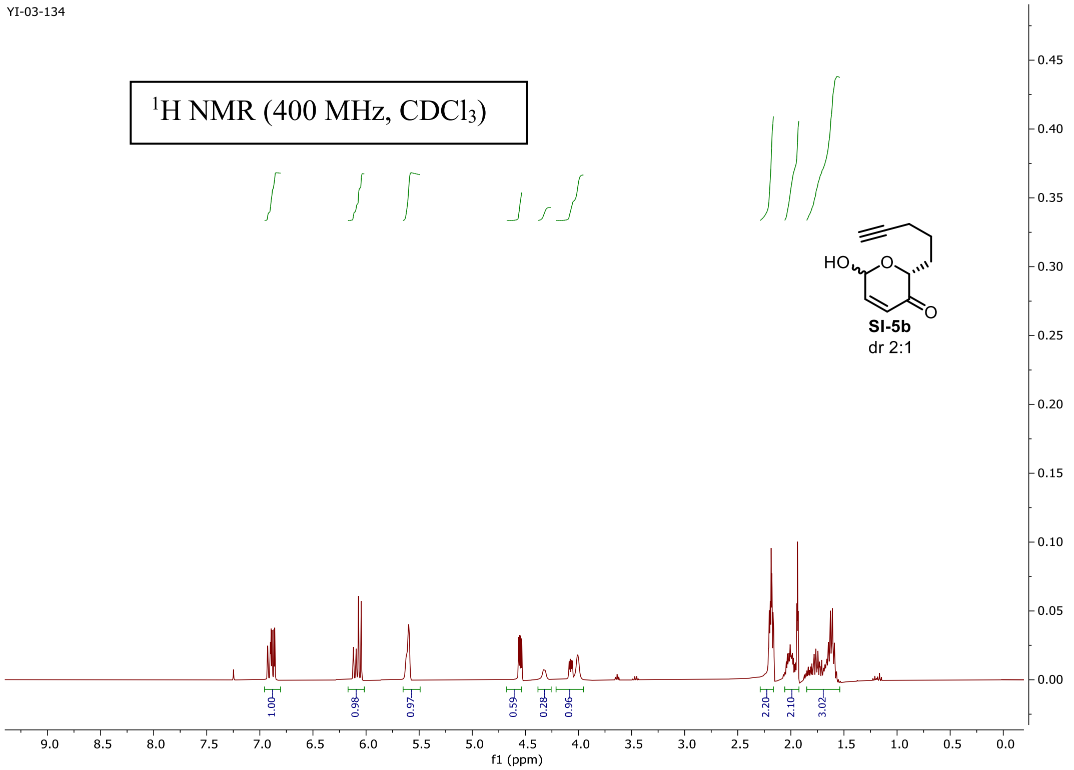
YI-03-134
^1H NMR (400 MHz, $CDCl_3$)
HO
O
O
SI-5b
dr 2:1
f1 (ppm)

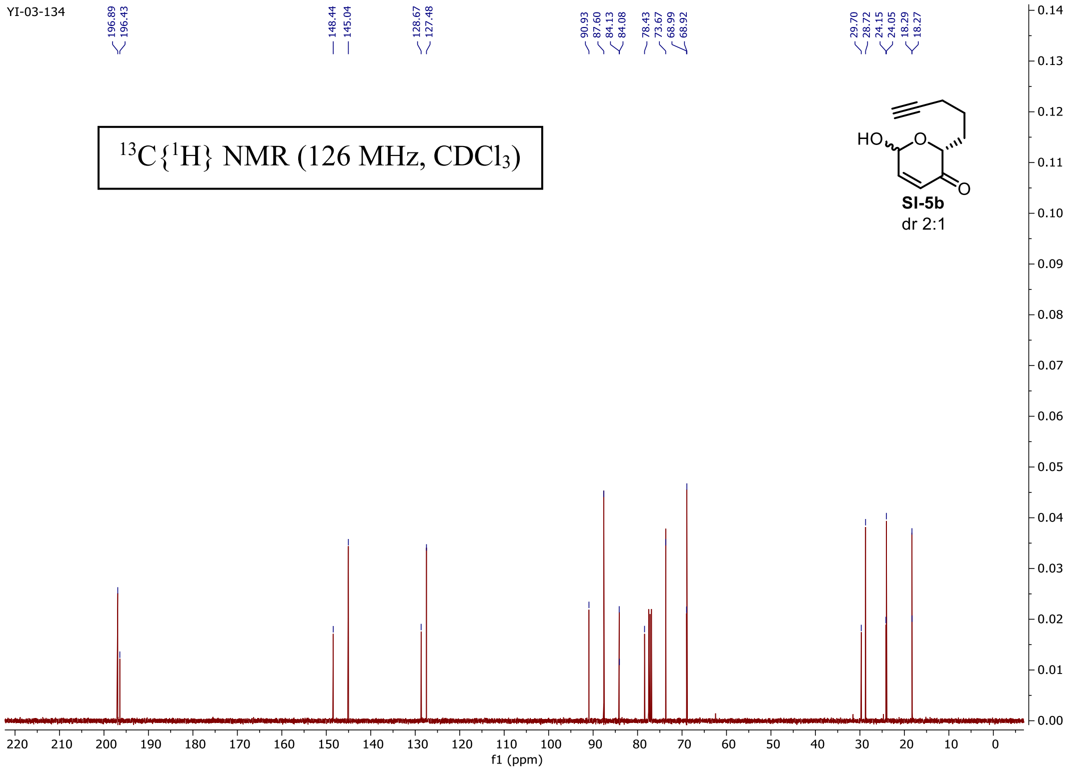
YI-03-134
196.89
196.43
148.44
145.04
128.67
127.48
90.93
87.60
84.13
84.08
78.43
73.67
68.99
68.92
29.70
28.72
24.15
24.05
18.29
18.27
$^{13}C\{^1H\}$ NMR (126 MHz, $CDCl_3$)
HO
O
O
SI-5b
dr 2:1
f1 (ppm)

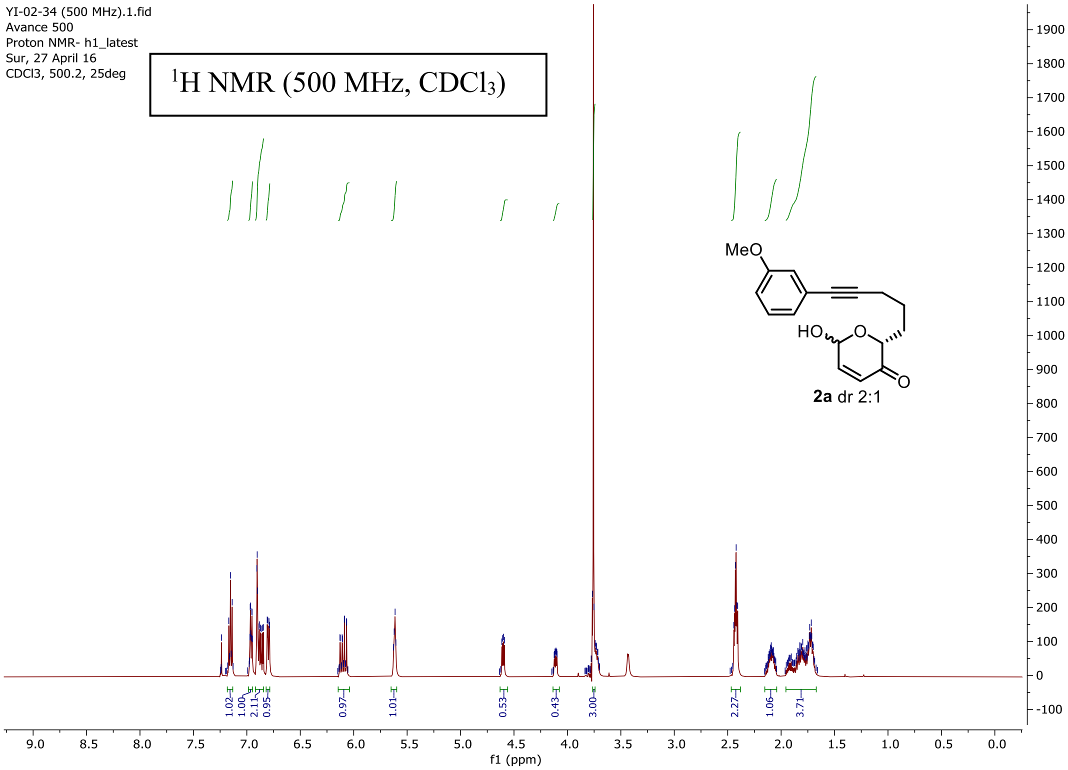
YI-02-34 (500 MHz).1.fid
Avance 500
Proton NMR- h1_latest
Sur, 27 April 16
CDCl3, 500.2, 25deg
1H NMR (500 MHz, CDCl3)
MeO
HO
O
O
2a dr 2:1
1.02
1.00
2.11
0.95
0.97
1.01
0.53
0.43
3.00
2.27
1.06
3.71
f1 (ppm)

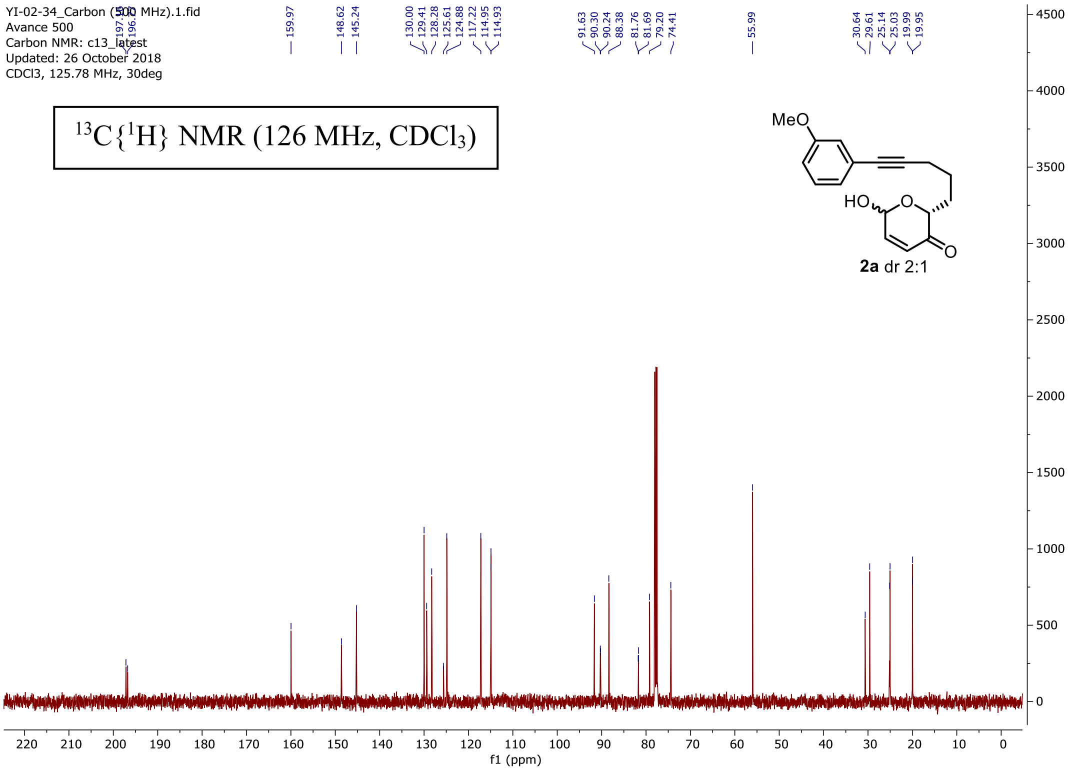
YI-02-34_Carbon (500 MHz).1.fid
Avance 500
Carbon NMR: c13_latest
Updated: 26 October 2018
CDCl3, 125.78 MHz, 30deg
13C{1H} NMR (126 MHz, CDCl3)
159.97
148.62
145.24
130.00
129.41
128.28
125.61
124.88
117.22
114.95
114.93
91.63
90.30
90.24
88.38
81.76
81.69
79.20
74.41
55.99
30.64
29.61
25.14
25.03
19.99
19.95
MeO
HO
O
O
2a dr 2:1
f1 (ppm)

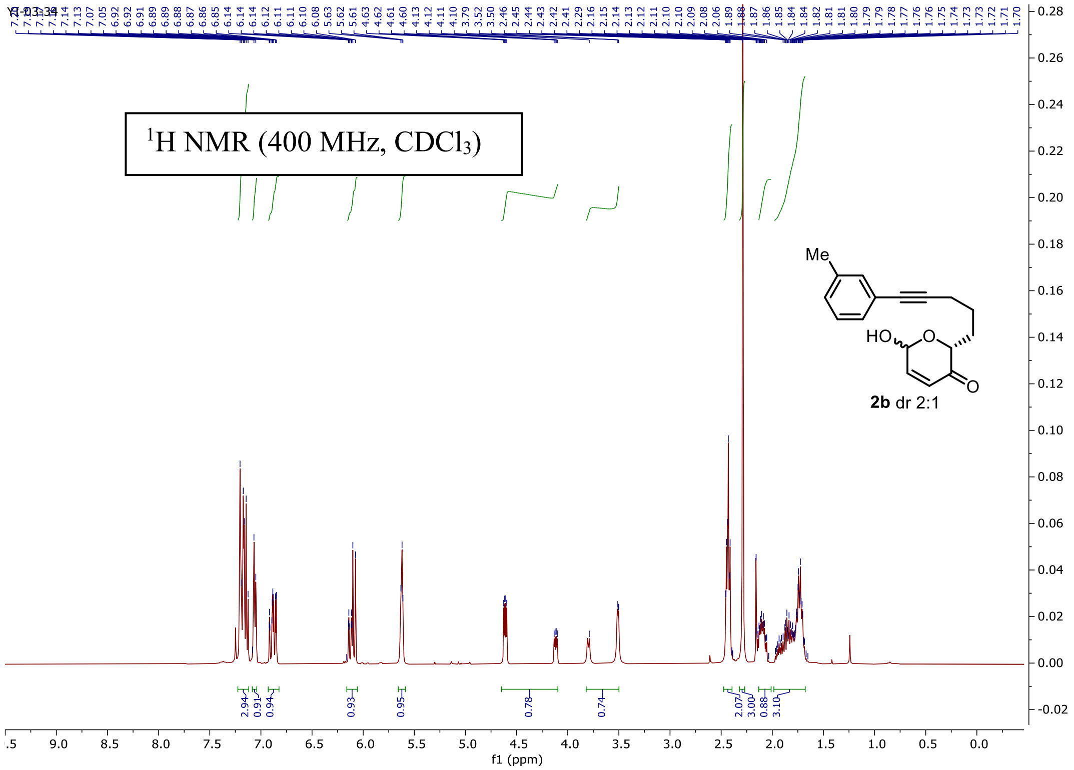

YI-03-34
1H NMR (400 MHz, CDCl3)
Me
HO
O
O
2b dr 2:1
f1 (ppm)

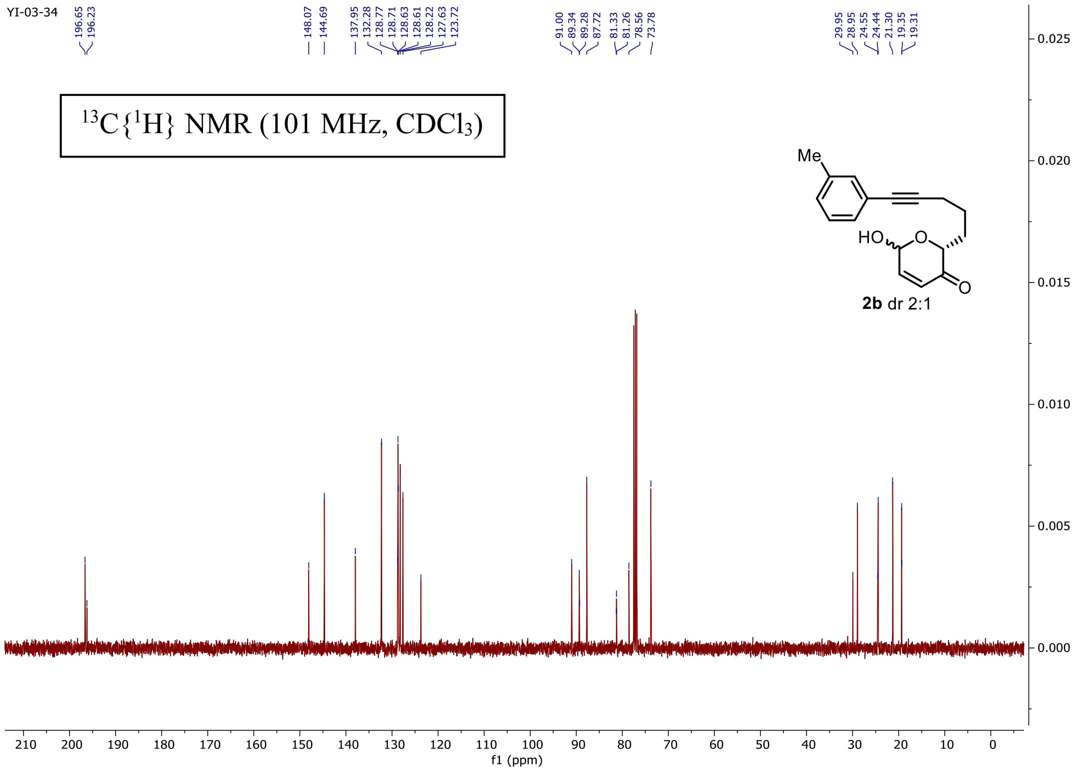

YI-03-34
196.65
196.23
148.07
144.69
137.95
132.28
128.77
128.71
128.63
128.61
128.22
127.63
123.72
91.00
89.34
89.28
87.72
81.33
81.26
78.56
73.78
29.95
28.95
24.55
24.44
21.30
19.35
19.31
13C{1H} NMR (101 MHz, CDCl3)
Me
HO
O
O
2b dr 2:1
f1 (ppm)

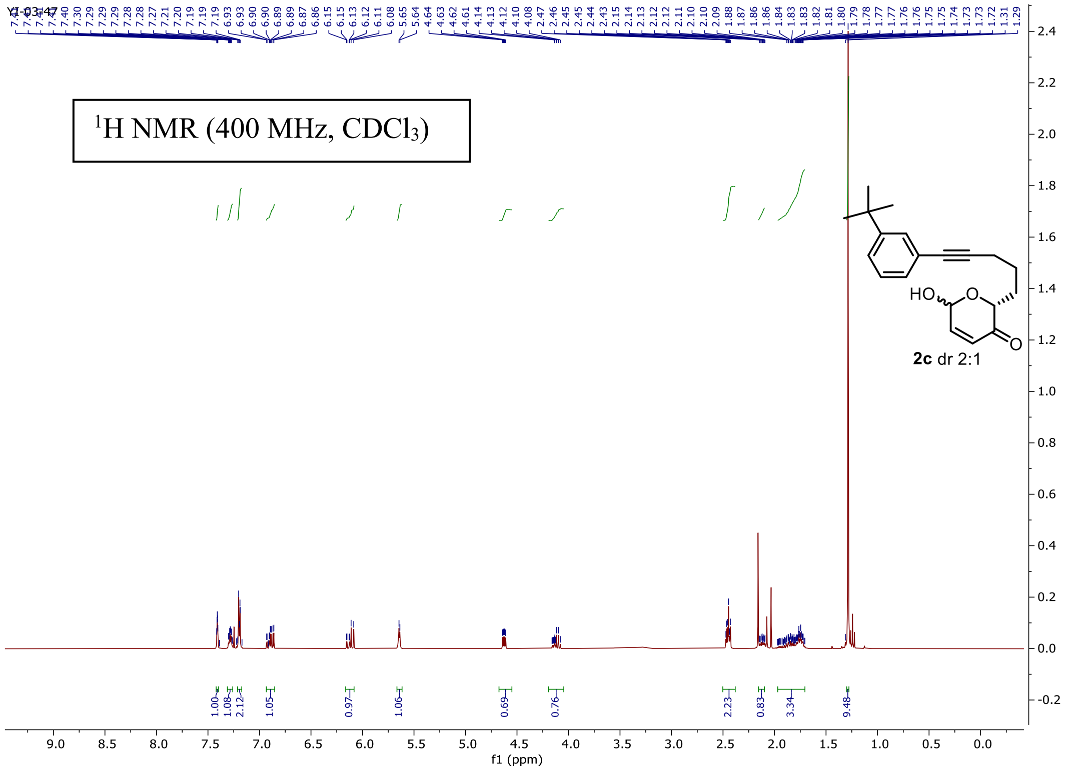

YI-03-47
^{1}H NMR (400 MHz, CDCl$_3$)
HO
O
O
2c dr 2:1
f1 (ppm)

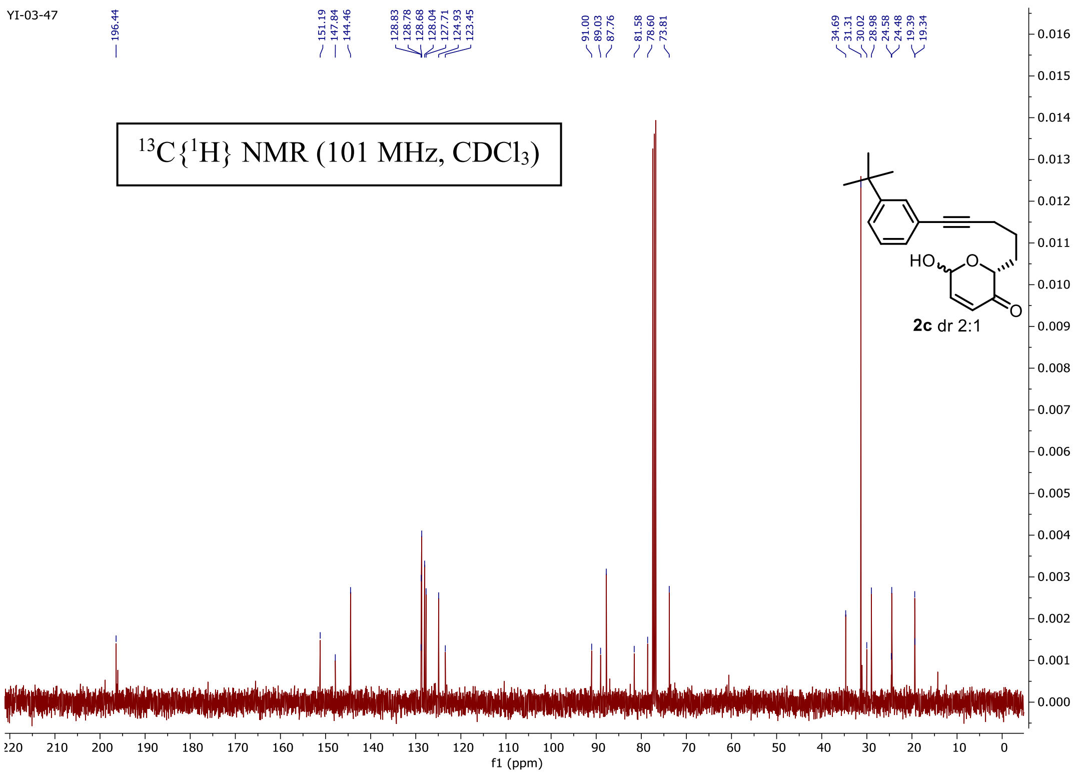

YI-03-47
196.44
151.19
147.84
144.46
128.83
128.78
128.68
128.04
127.71
124.93
123.45
91.00
89.03
87.76
81.58
78.60
73.81
34.69
31.31
30.02
28.98
24.58
24.48
19.39
19.34
^{13}C{^{1}H} NMR (101 MHz, CDCl$_3$)
HO
O
O
2c dr 2:1
f1 (ppm)

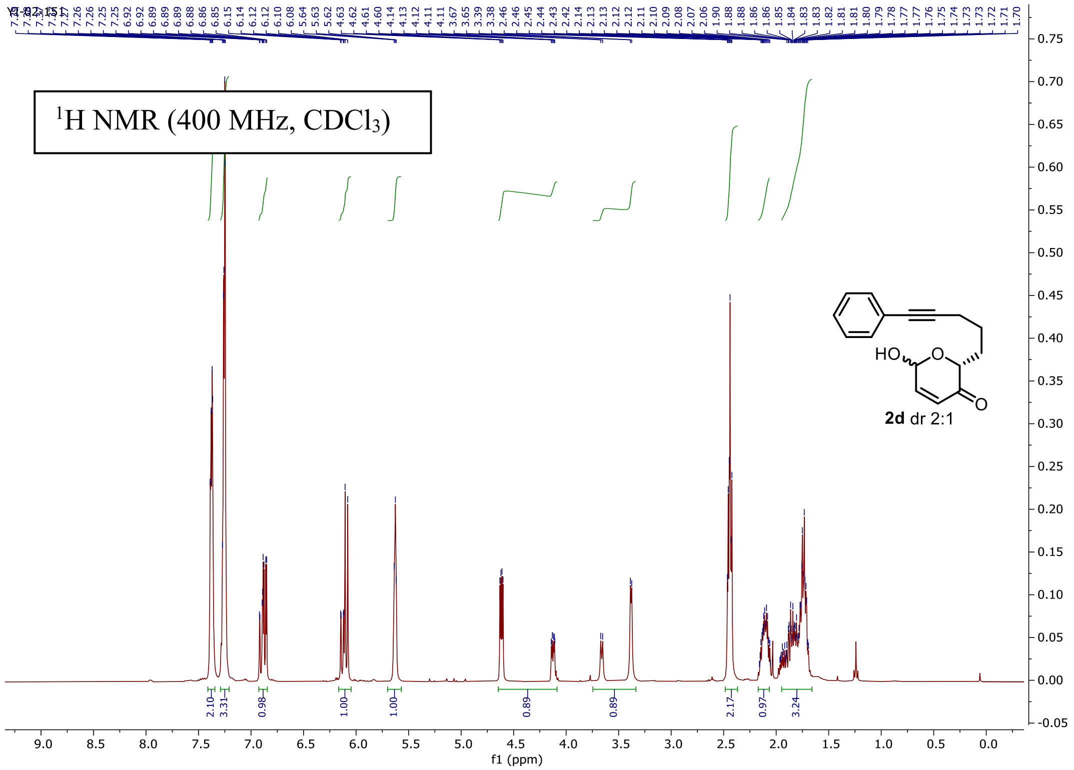

YI-02-151
¹H NMR (400 MHz, CDCl₃)
HO
O
O
2d dr 2:1
f1 (ppm)

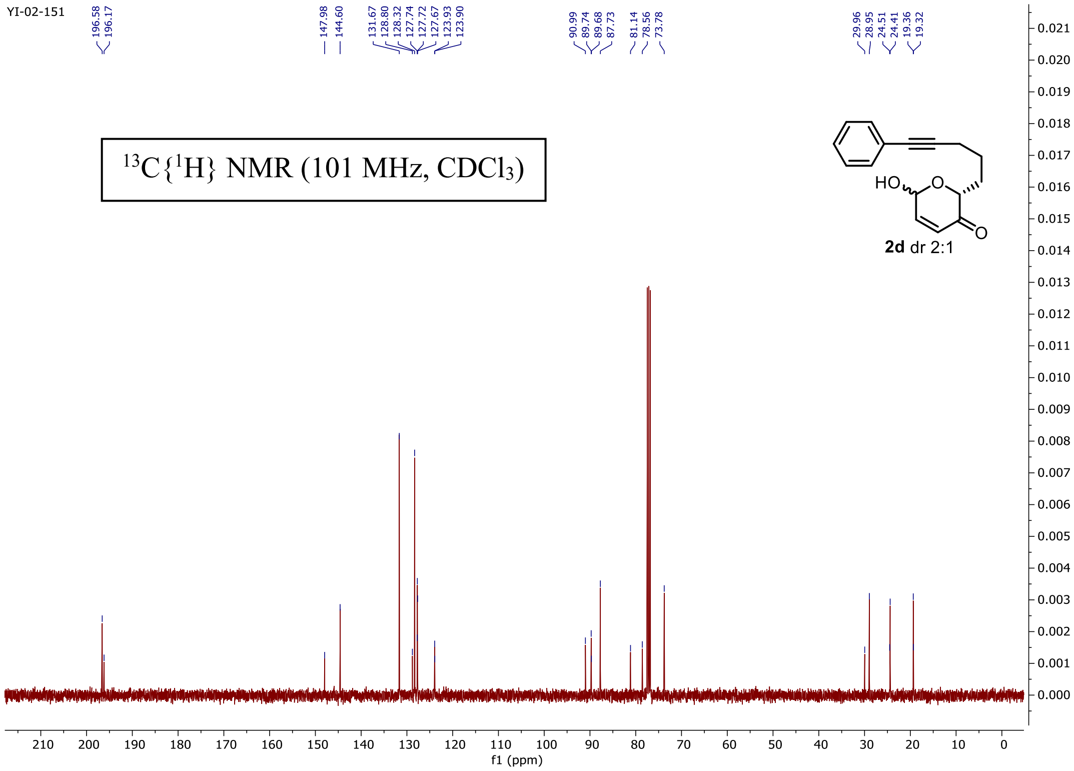

YI-02-151
¹³C{¹H} NMR (101 MHz, CDCl₃)
196.58
196.17
147.98
144.60
131.67
128.80
128.32
127.74
127.72
127.67
123.93
123.90
90.99
89.74
89.68
87.73
81.14
78.56
73.78
29.96
28.95
24.51
24.41
19.36
19.32
HO
O
O
2d dr 2:1
f1 (ppm)

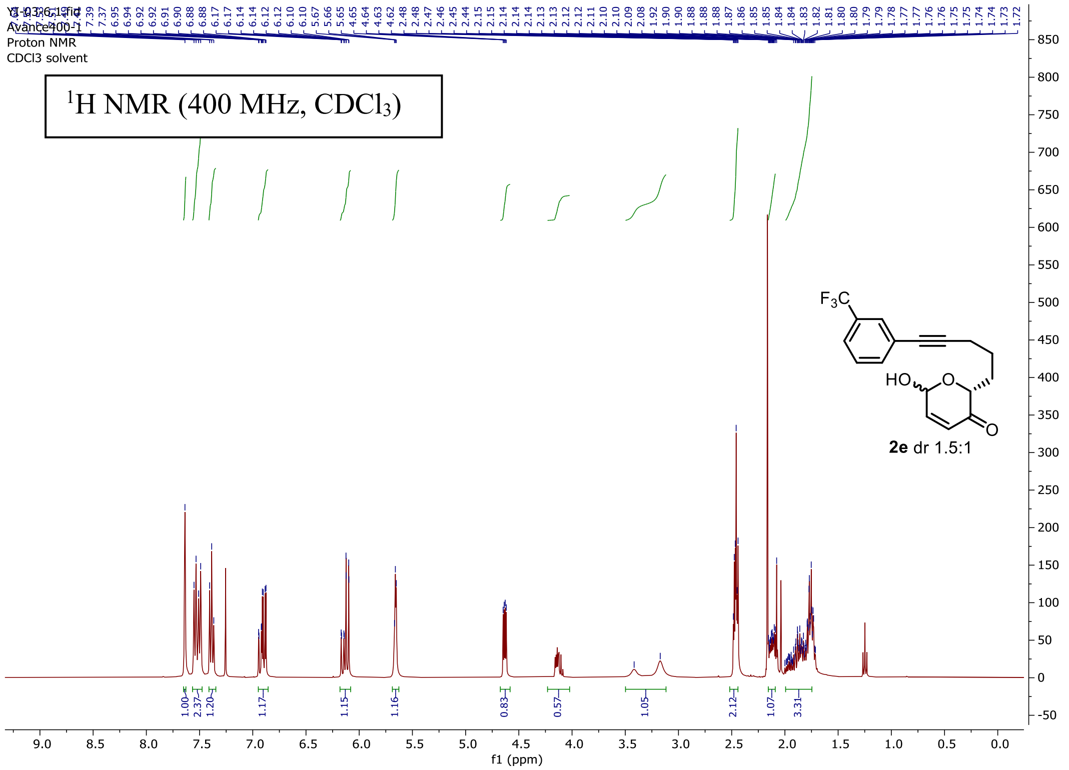

^{1}H NMR (400 MHz, $CDCl_3$)
2e dr 1.5:1
f1 (ppm)

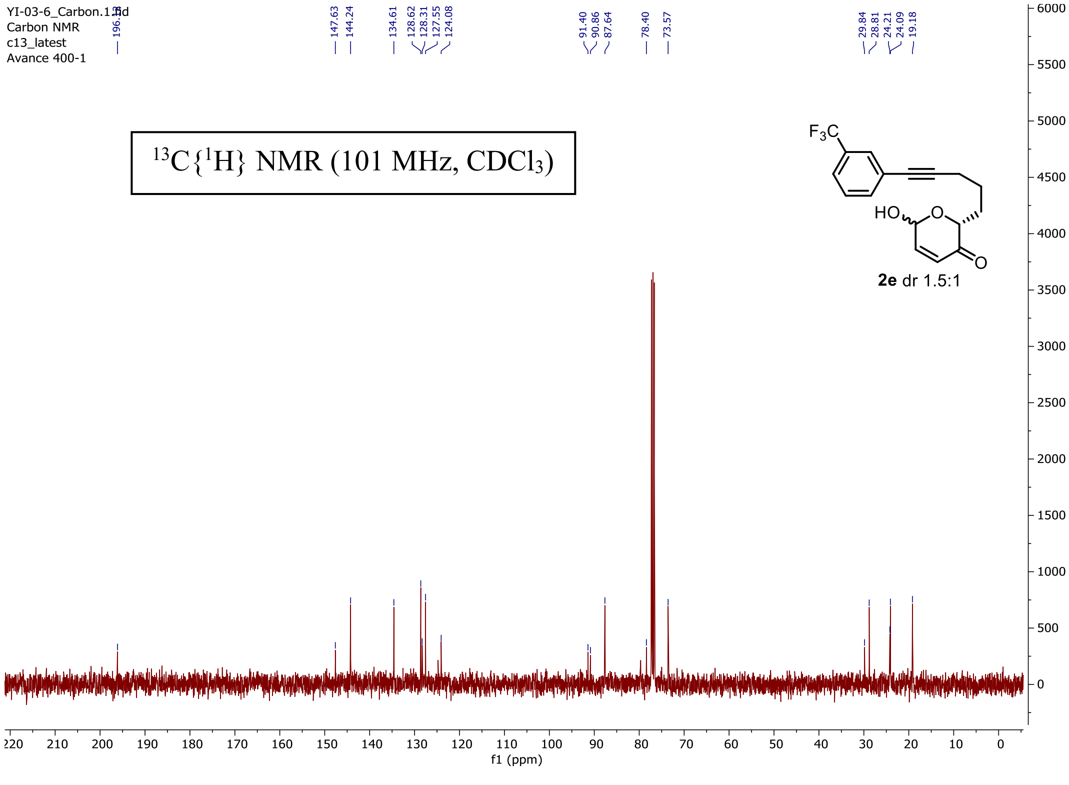

$^{13}C\{^1H\}$ NMR (101 MHz, $CDCl_3$)
2e dr 1.5:1
f1 (ppm)

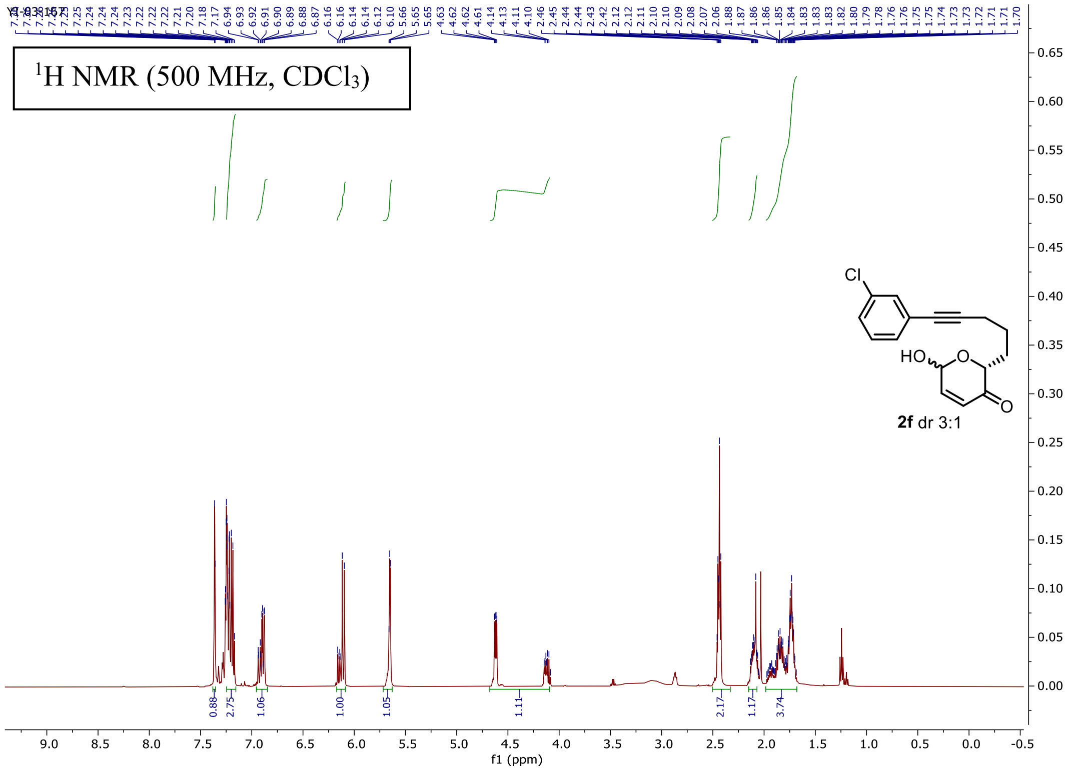
YI-03-167
1H NMR (500 MHz, CDCl3)
Cl
HO
O
O
2f dr 3:1
f1 (ppm)

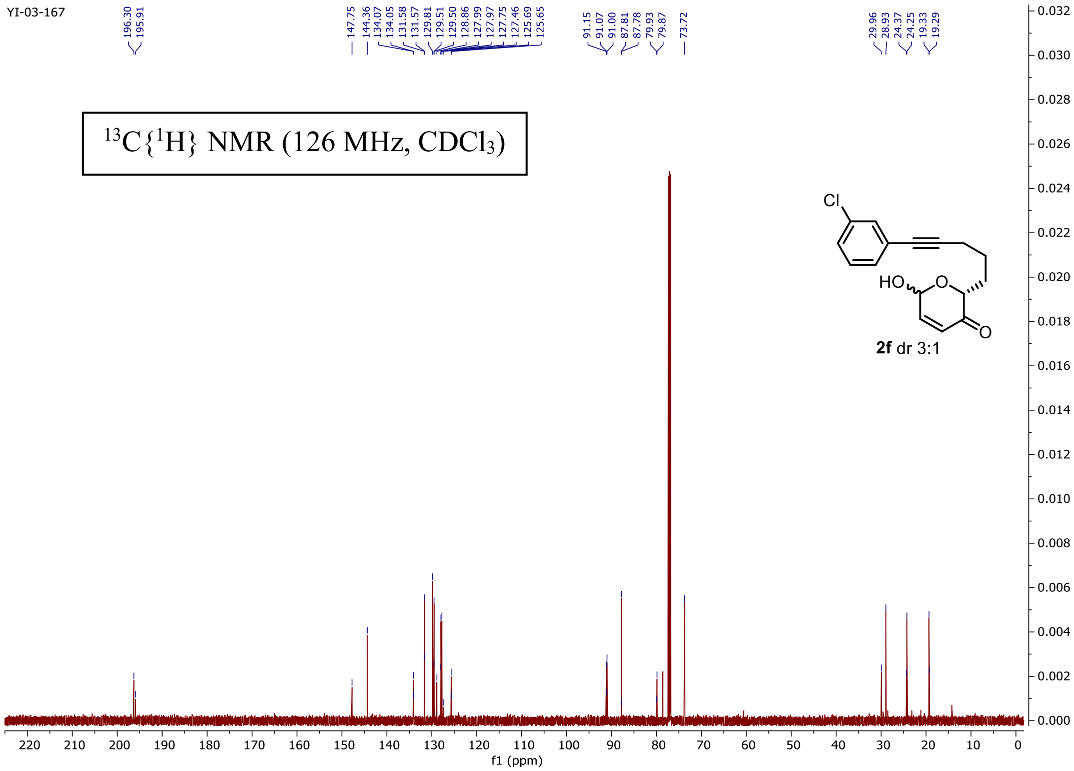
YI-03-167
13C{1H} NMR (126 MHz, CDCl3)
Cl
HO
O
O
2f dr 3:1
f1 (ppm)

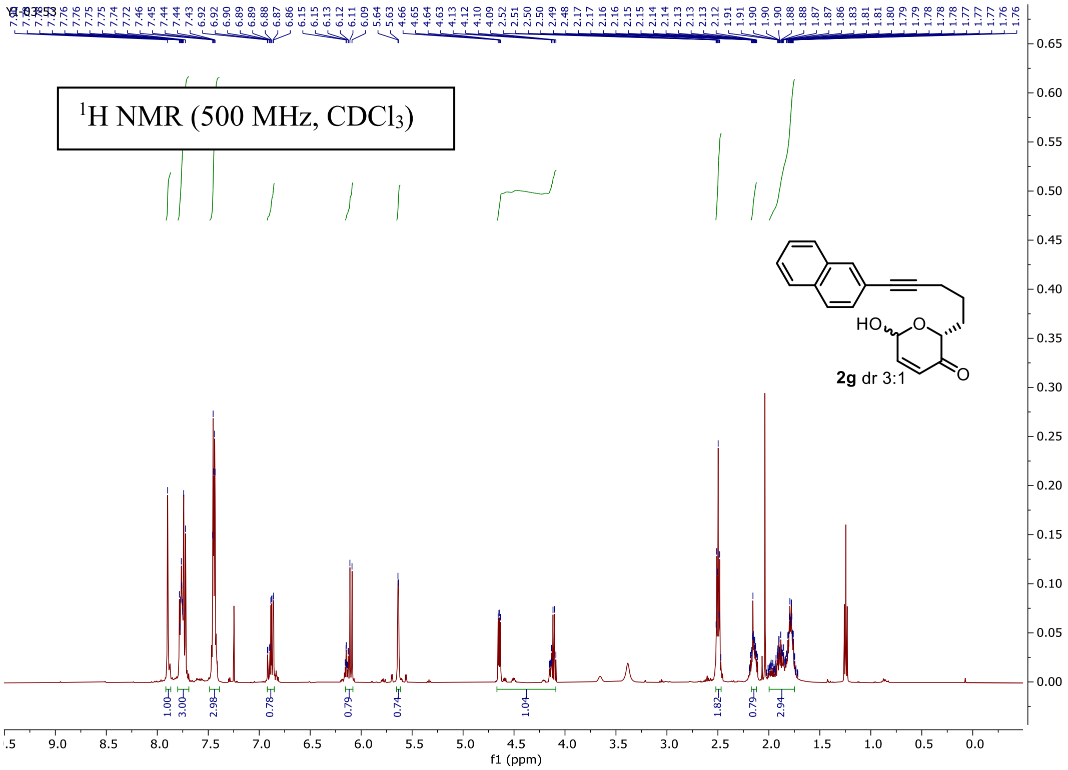

YI-03-53
¹H NMR (500 MHz, CDCl₃)
HO
O
O
2g dr 3:1
f1 (ppm)

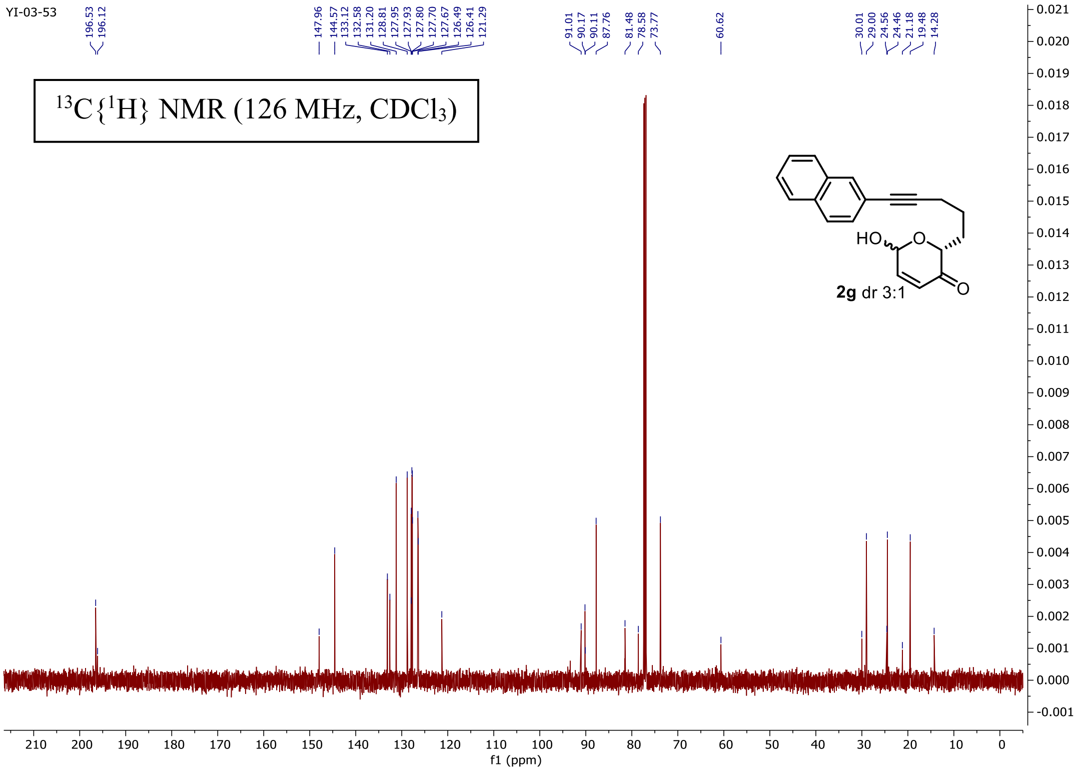

YI-03-53
¹³C{¹H} NMR (126 MHz, CDCl₃)
HO
O
O
2g dr 3:1
f1 (ppm)

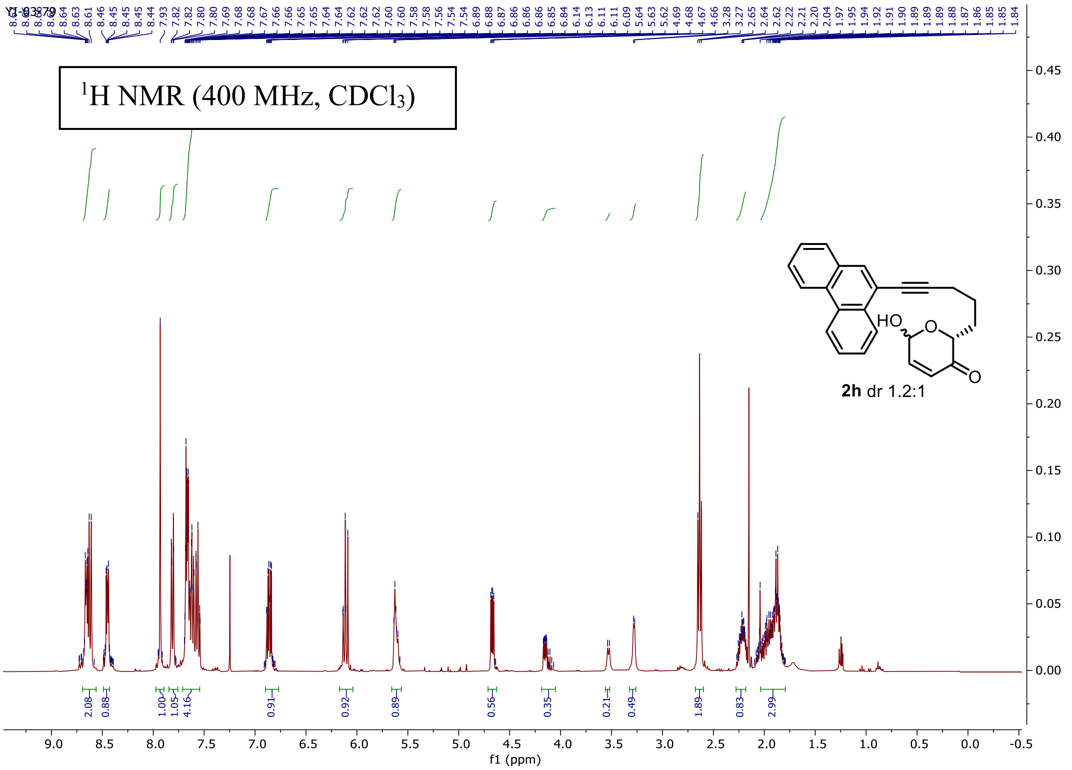
YI-03-79
¹H NMR (400 MHz, CDCl₃)
HO
O
O
2h dr 1.2:1
f1 (ppm)

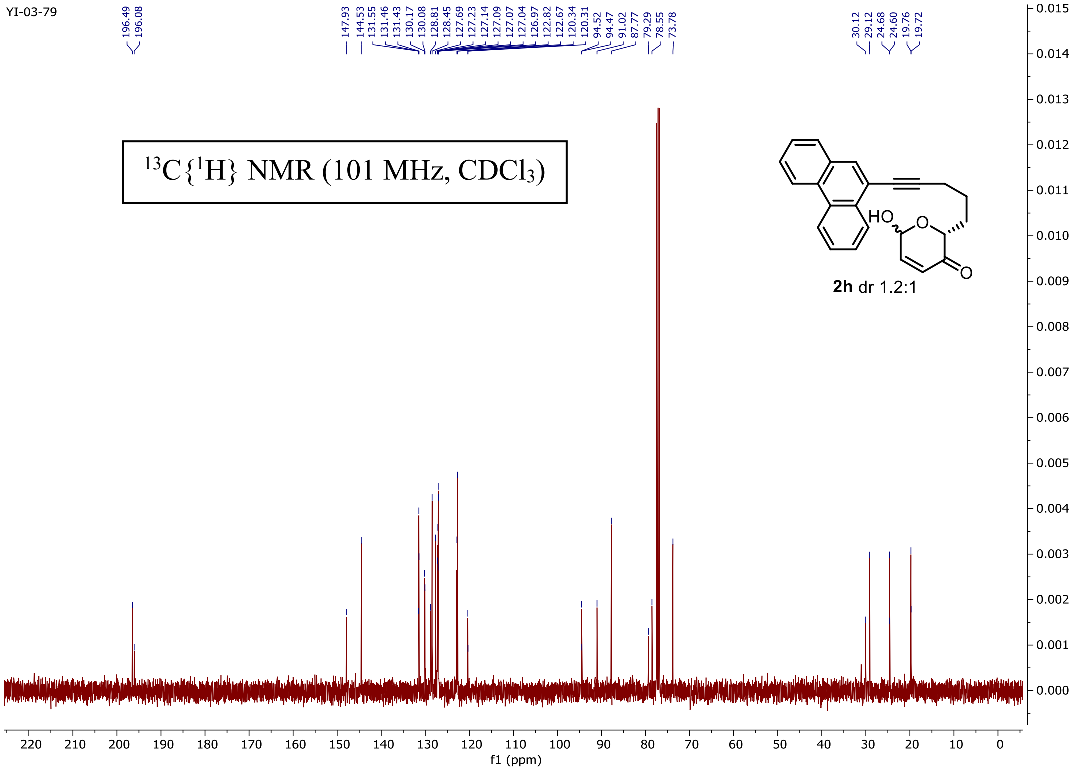
YI-03-79
¹³C{¹H} NMR (101 MHz, CDCl₃)
HO
O
O
2h dr 1.2:1
f1 (ppm)

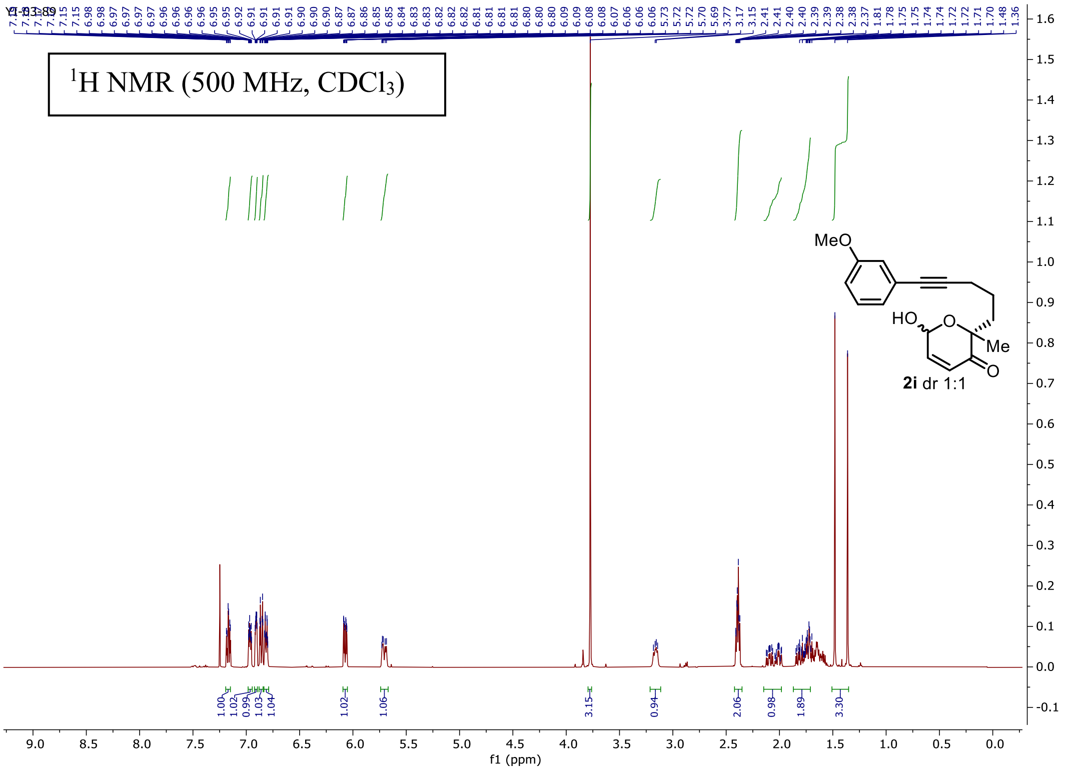
YI-03-89
1H NMR (500 MHz, CDCl3)
MeO
HO
O
Me
O
2i dr 1:1
f1 (ppm)

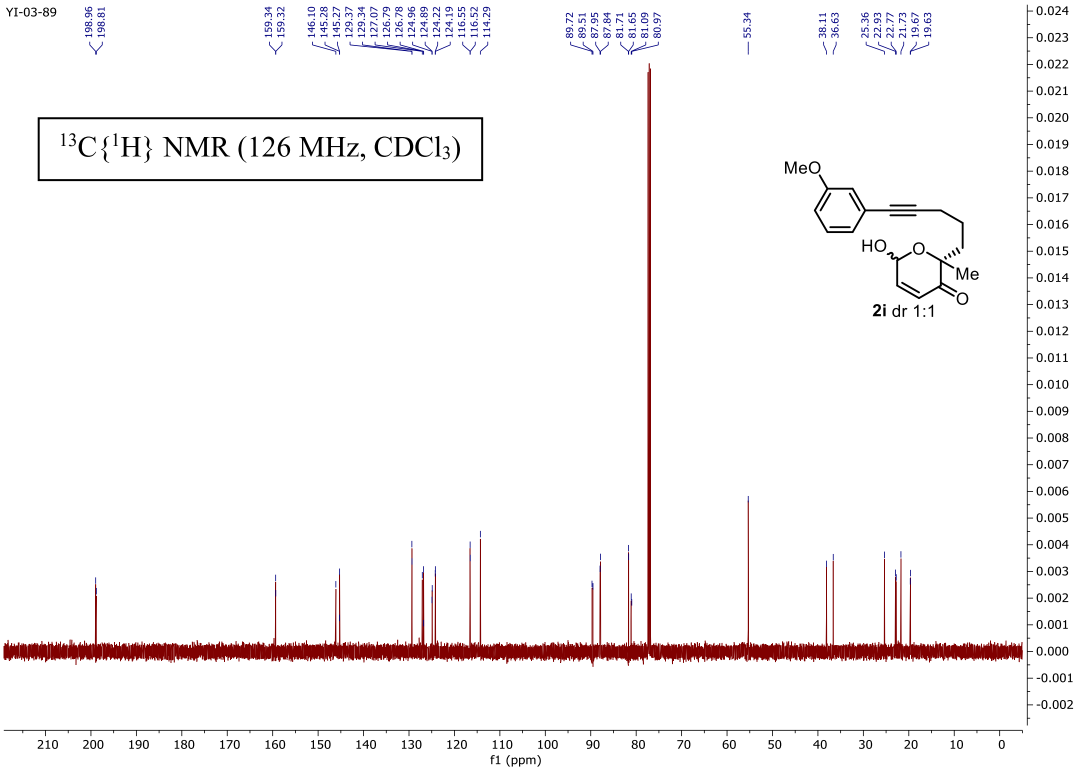
YI-03-89
13C{1H} NMR (126 MHz, CDCl3)
MeO
HO
O
Me
O
2i dr 1:1
f1 (ppm)

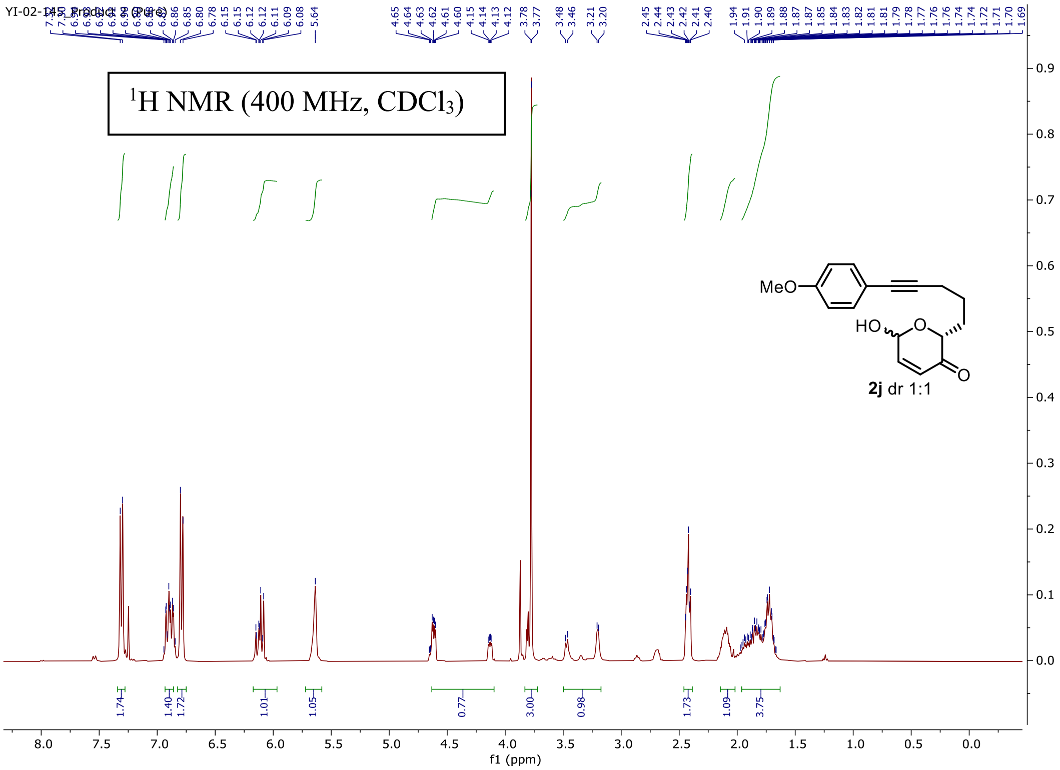

YI-02-145_Product 2 (Pure)
^{1}H NMR (400 MHz, CDCl$_3$)
MeO
HO
O
O
2j dr 1:1
f1 (ppm)

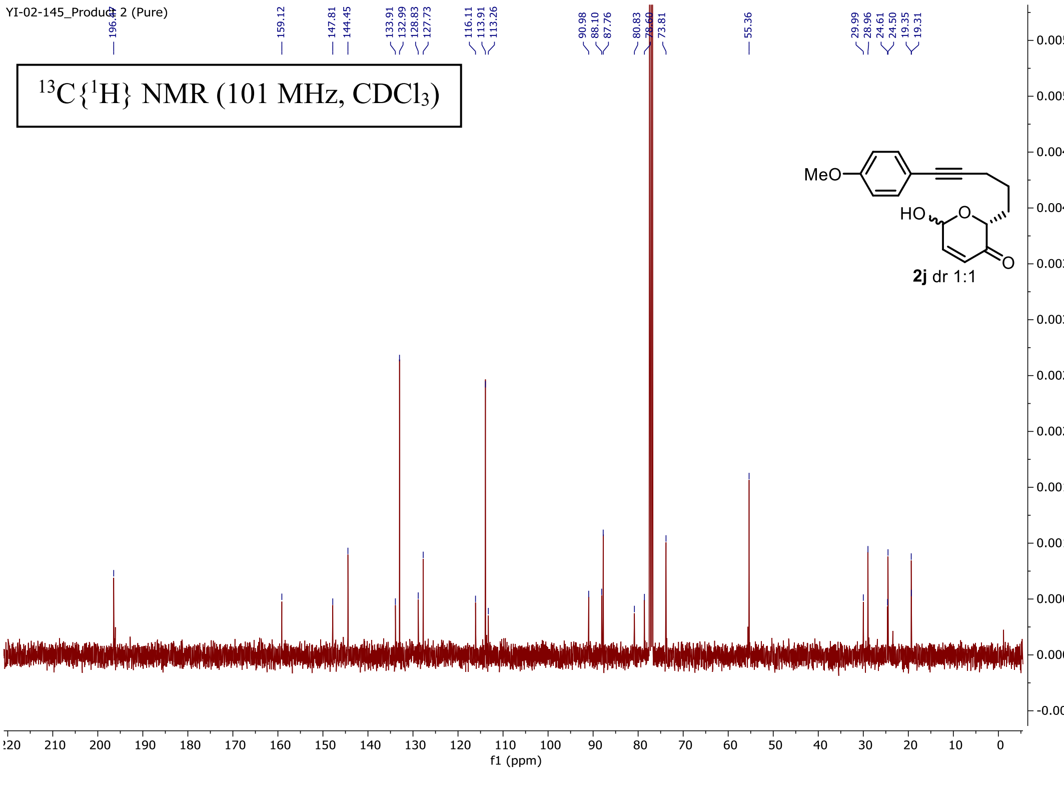

YI-02-145_Product 2 (Pure)
^{13}C{^{1}H} NMR (101 MHz, CDCl$_3$)
MeO
HO
O
O
2j dr 1:1
f1 (ppm)

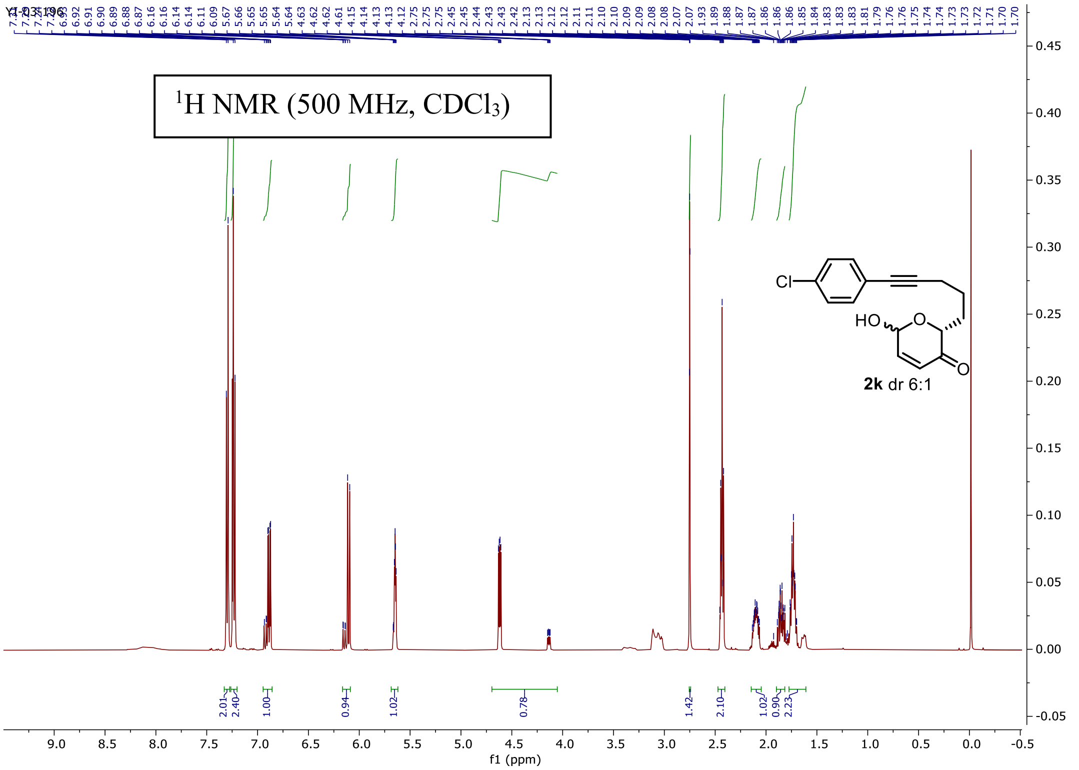
1H NMR (500 MHz, CDCl3)
2k dr 6:1
f1 (ppm)

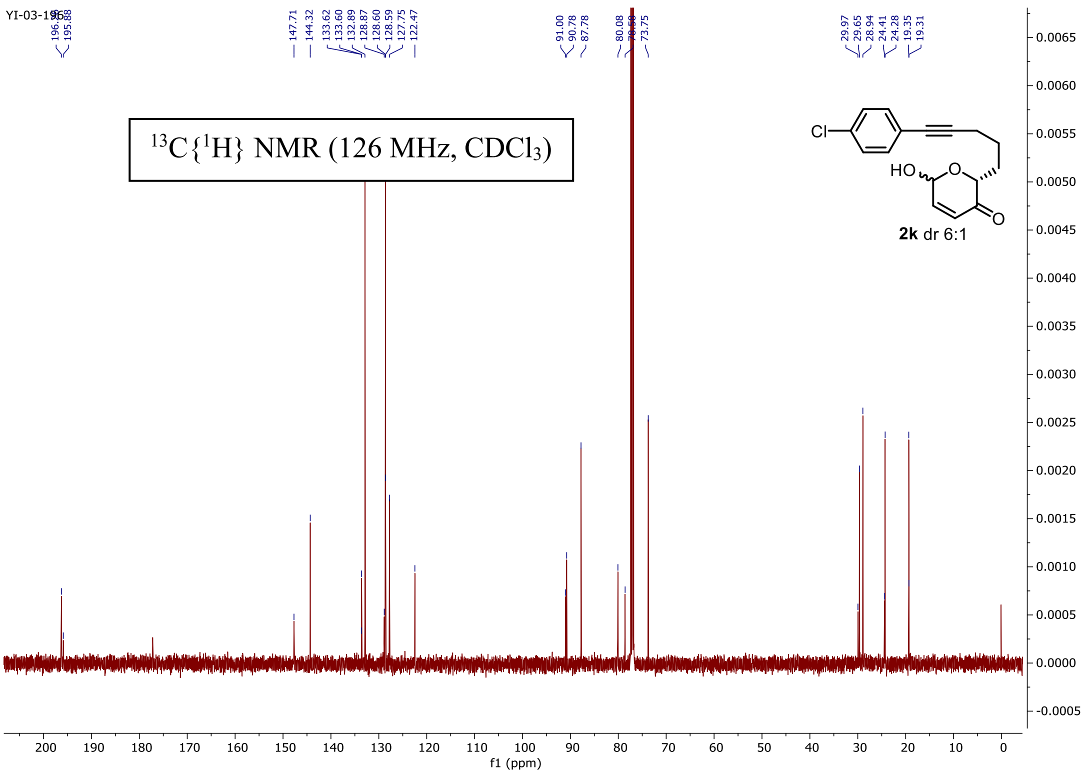
13C{1H} NMR (126 MHz, CDCl3)
2k dr 6:1
f1 (ppm)

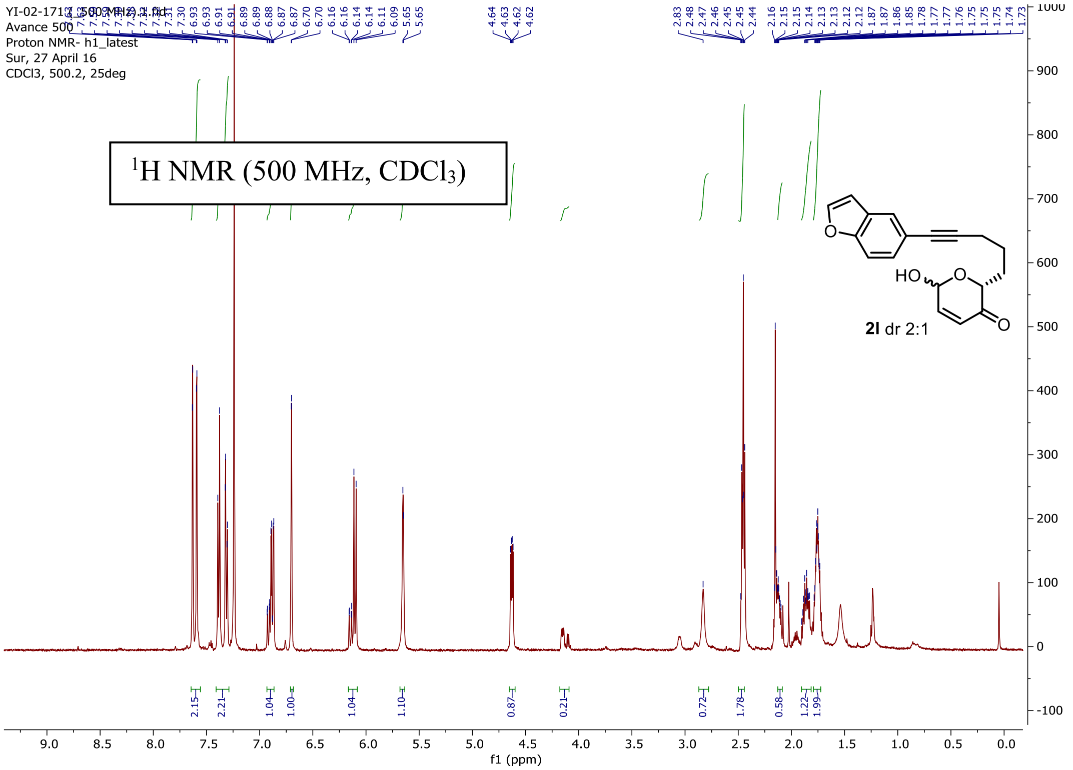
Avance 500
Proton NMR- h1_latest
Sur, 27 April 16
CDCl3, 500.2, 25deg
1H NMR (500 MHz, CDCl3)
HO
O
2l dr 2:1
f1 (ppm)

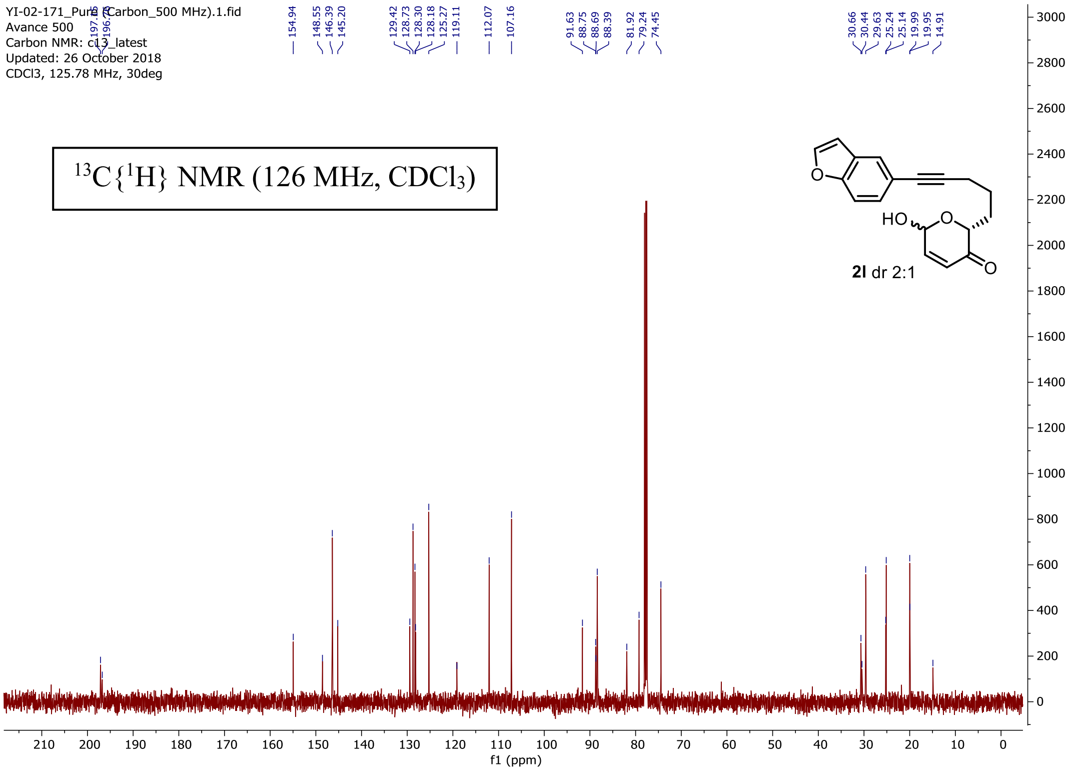
YI-02-171_Pure (Carbon_500 MHz).1.fid
Avance 500
Carbon NMR: c13_latest
Updated: 26 October 2018
CDCl3, 125.78 MHz, 30deg
13C{1H} NMR (126 MHz, CDCl3)
HO
O
2l dr 2:1
f1 (ppm)

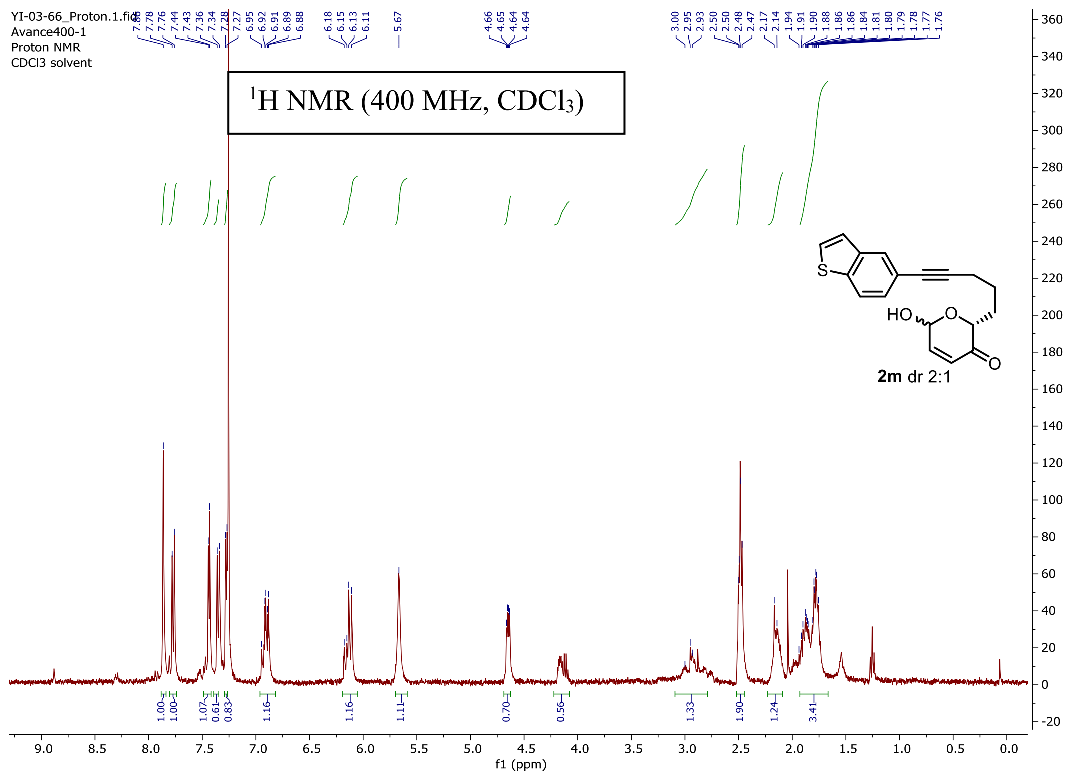
YI-03-66_Proton.1.fid
Avance400-1
Proton NMR
CDCl3 solvent
1H NMR (400 MHz, CDCl3)
2m dr 2:1
f1 (ppm)

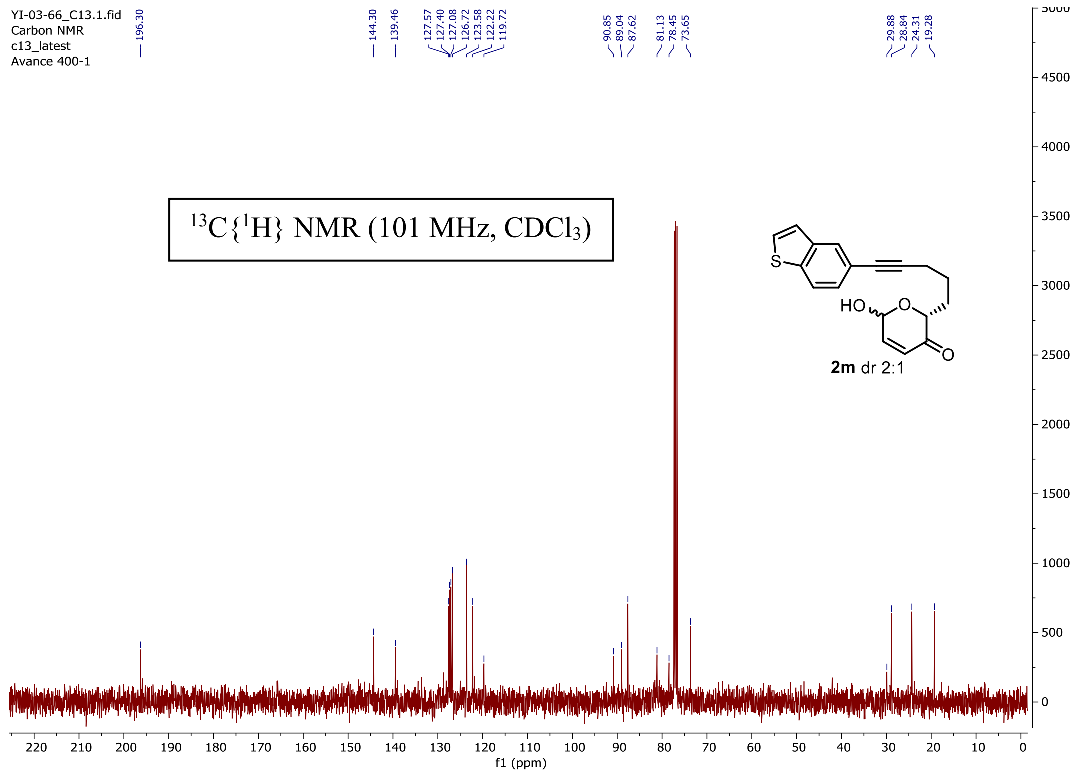
YI-03-66_C13.1.fid
Carbon NMR
c13_latest
Avance 400-1
13C{1H} NMR (101 MHz, CDCl3)
2m dr 2:1
f1 (ppm)

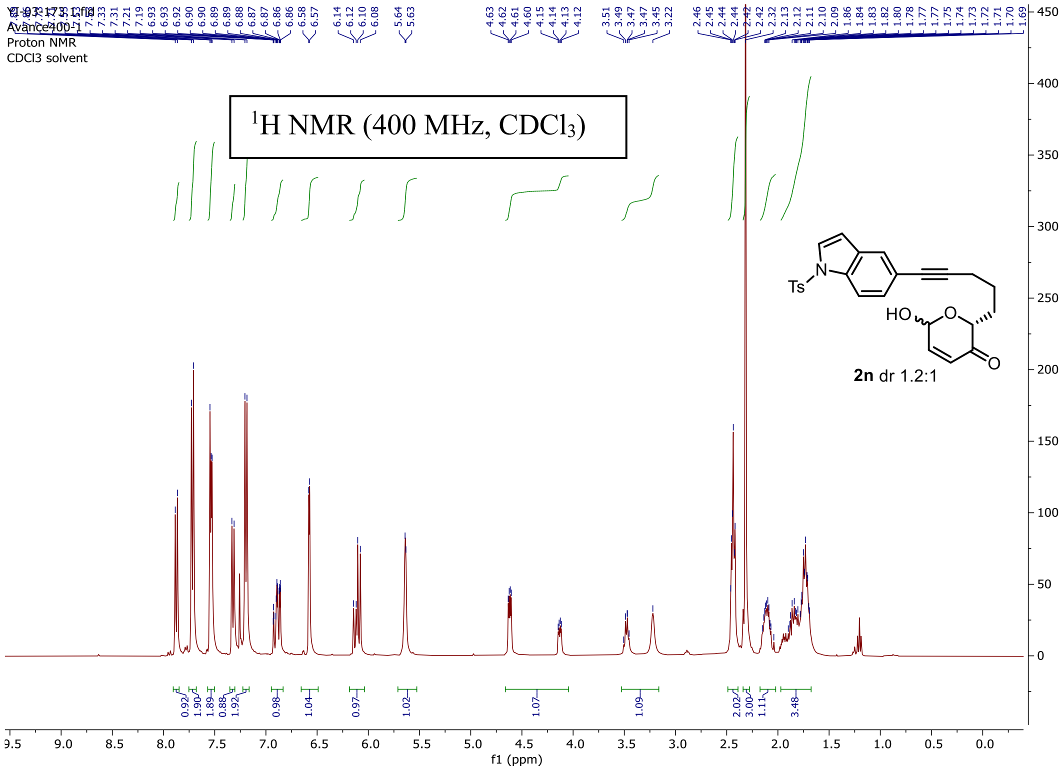
YI-03-173_1.fid
Avance 400-1
Proton NMR
CDCl3 solvent
^{1}H NMR (400 MHz, CDCl$_3$)
Ts
N
HO
O
O
2n dr 1.2:1
f1 (ppm)

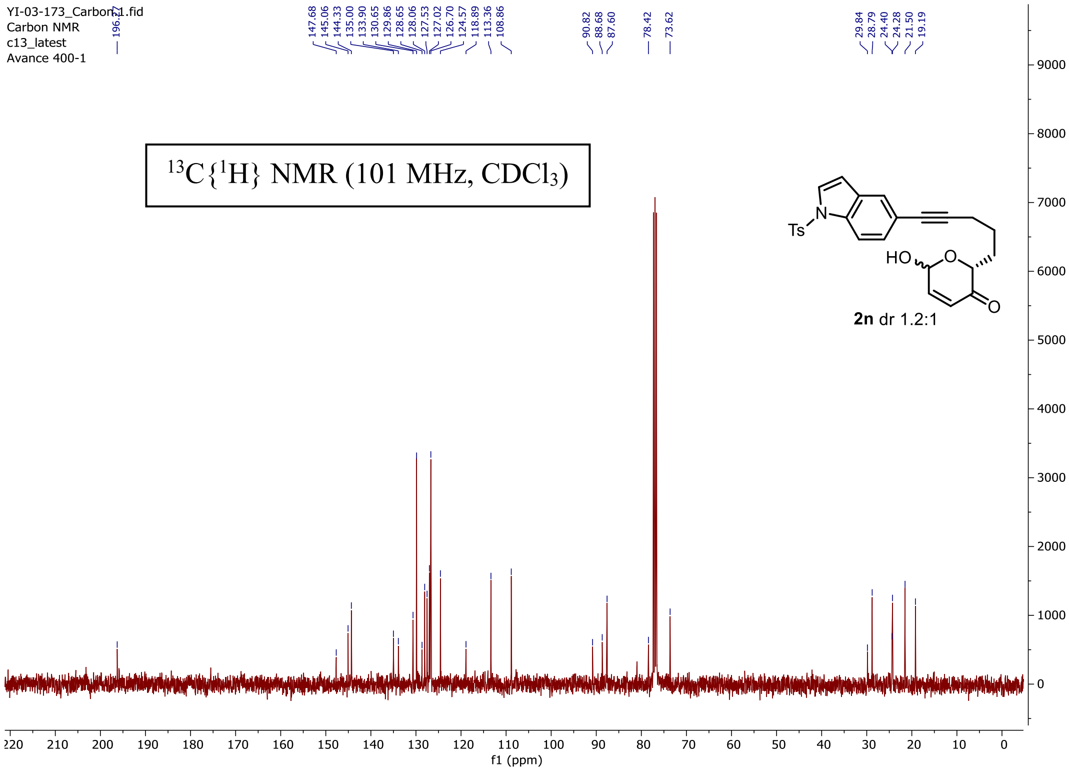
YI-03-173_Carbon-1.fid
Carbon NMR
c13_latest
Avance 400-1
^{13}C{^{1}H} NMR (101 MHz, CDCl$_3$)
Ts
N
HO
O
O
2n dr 1.2:1
f1 (ppm)

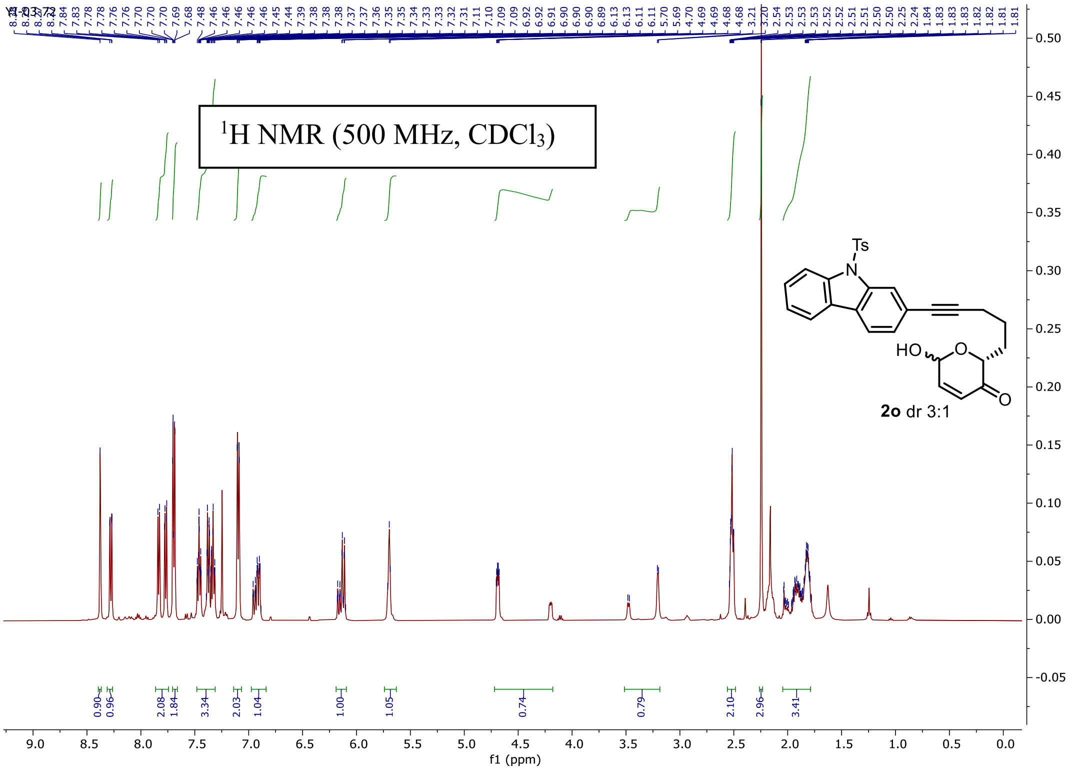
YI-03-72
1H NMR (500 MHz, CDCl3)
Ts
N
HO
O
O
2o dr 3:1
f1 (ppm)

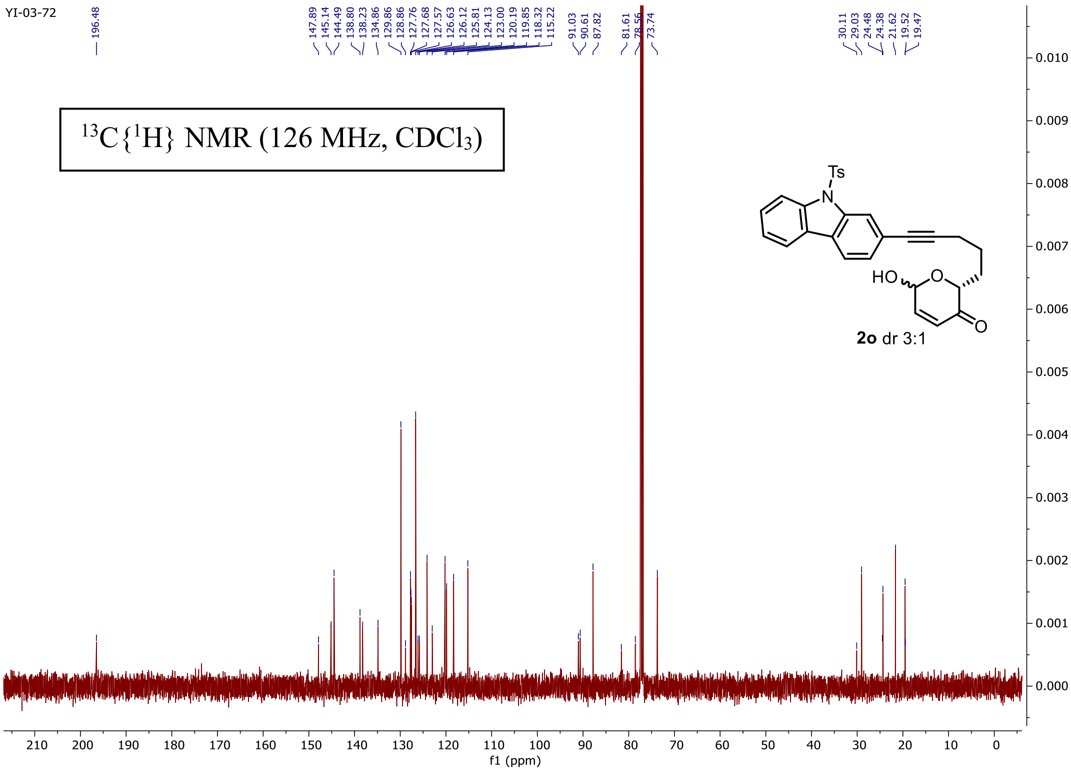
YI-03-72
196.48
147.89
145.14
144.49
138.80
138.23
134.86
129.86
128.86
127.76
127.68
127.57
126.63
126.12
125.81
124.13
123.00
120.19
119.85
118.32
115.22
91.03
90.61
87.82
81.61
78.56
73.74
30.11
29.03
24.48
24.38
21.62
19.52
19.47
13C{1H} NMR (126 MHz, CDCl3)
Ts
N
HO
O
O
2o dr 3:1
f1 (ppm)

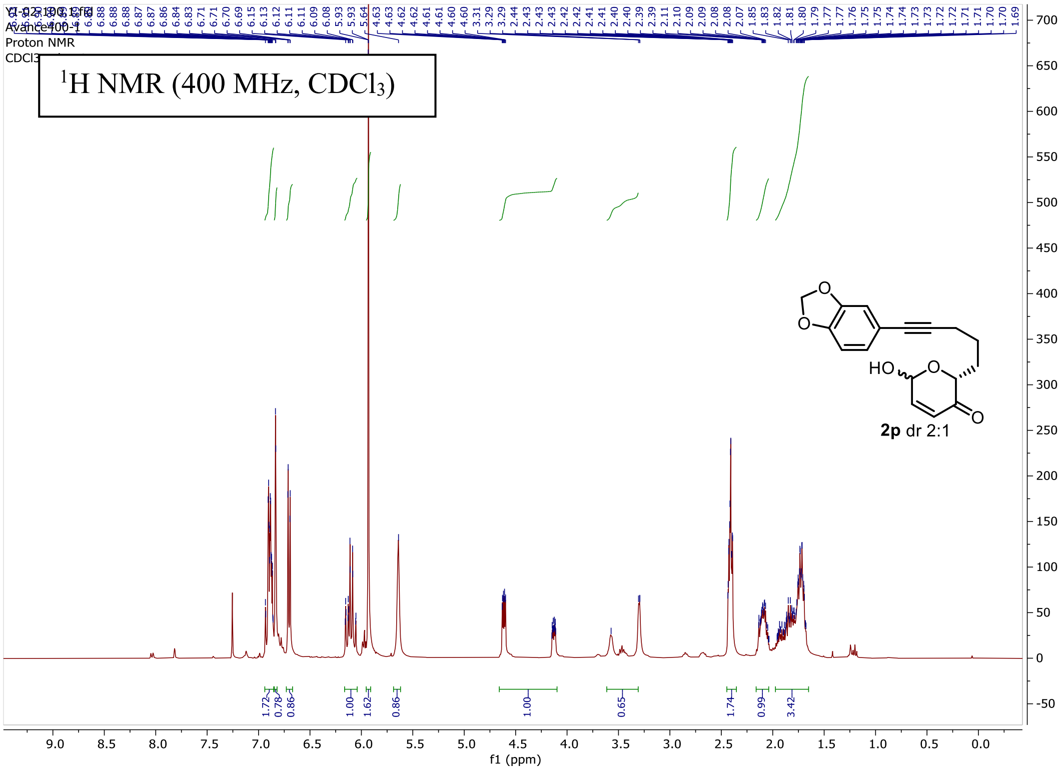

YI-02-100 Proton.fid
Avance 400-1
Proton NMR
CDCl3
^{1}H NMR (400 MHz, CDCl$_3$)
HO
O
2p dr 2:1
f1 (ppm)

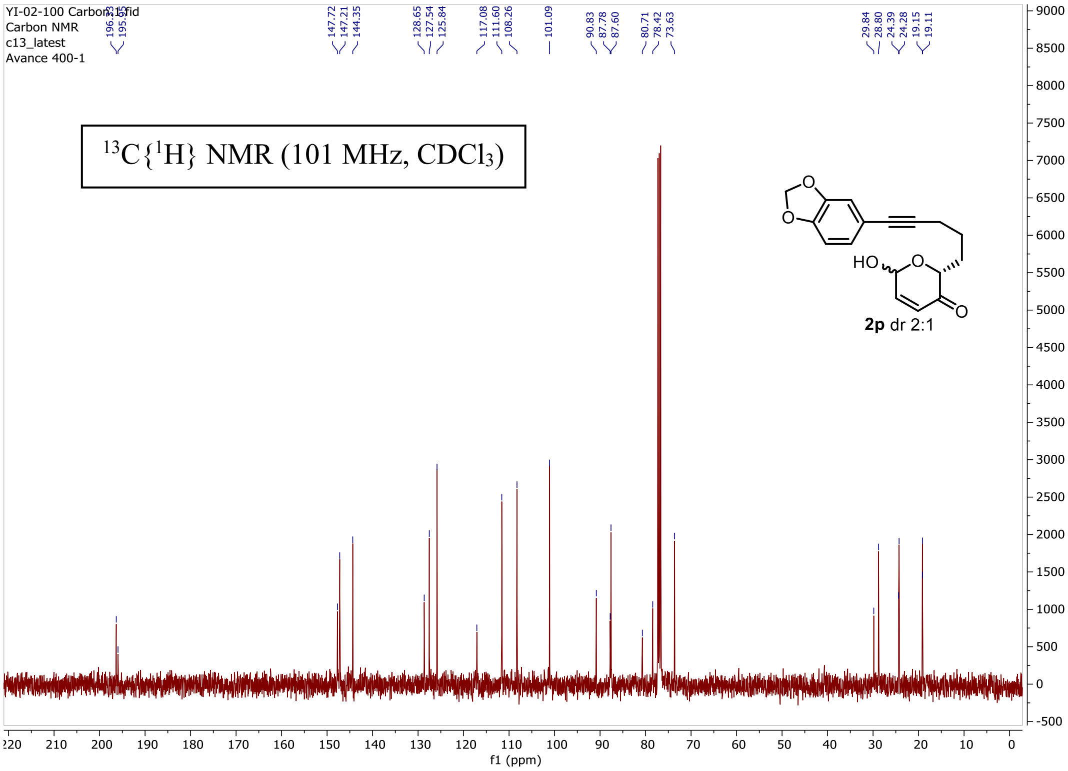

YI-02-100 Carbon.fid
Carbon NMR
c13_latest
Avance 400-1
^{13}C{^{1}H} NMR (101 MHz, CDCl$_3$)
196.15
195.94
147.72
147.21
144.35
128.65
127.54
125.84
117.08
111.60
108.26
101.09
90.83
87.78
87.60
80.71
78.42
73.63
29.84
28.80
24.39
24.28
19.15
19.11
HO
O
2p dr 2:1
f1 (ppm)

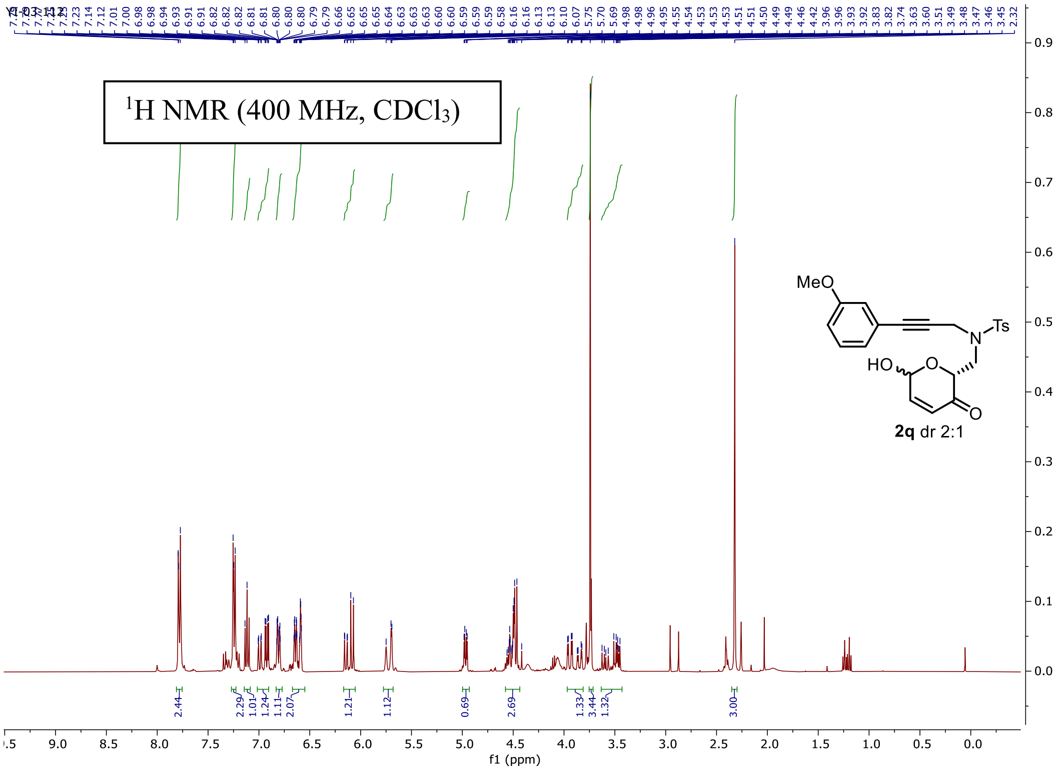

YI-03-112
1H NMR (400 MHz, CDCl3)
MeO
Ts
N
HO
O
O
2q dr 2:1
f1 (ppm)

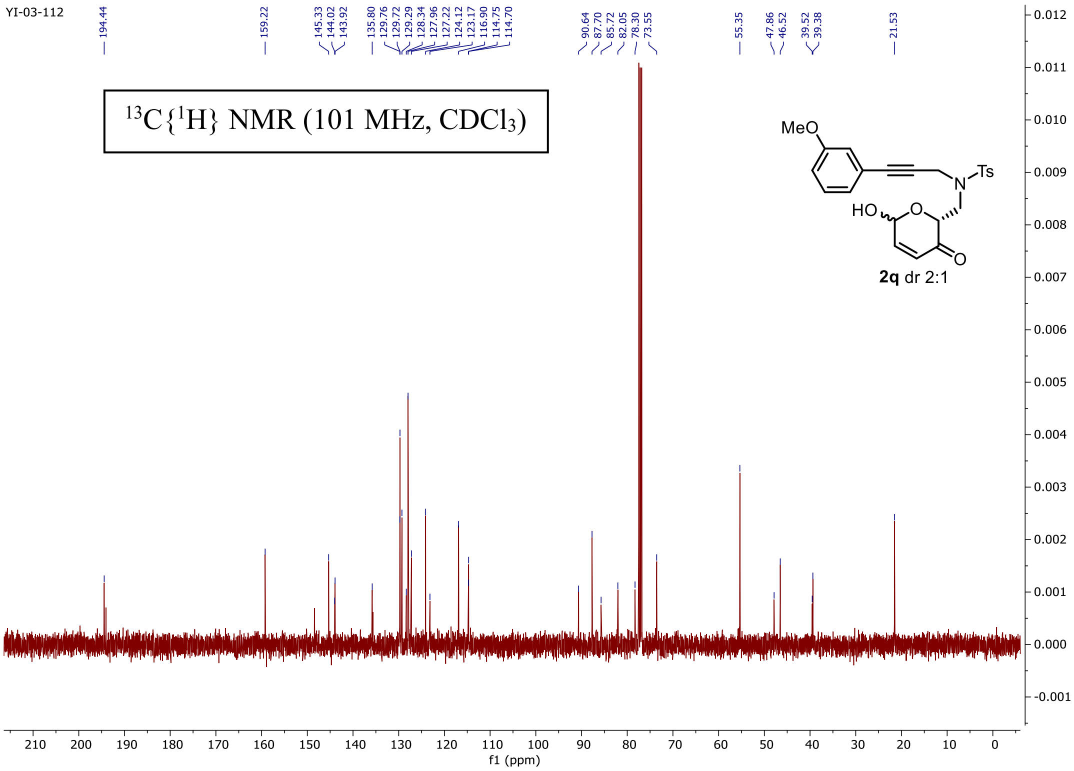

YI-03-112
13C{1H} NMR (101 MHz, CDCl3)
MeO
Ts
N
HO
O
O
2q dr 2:1
f1 (ppm)

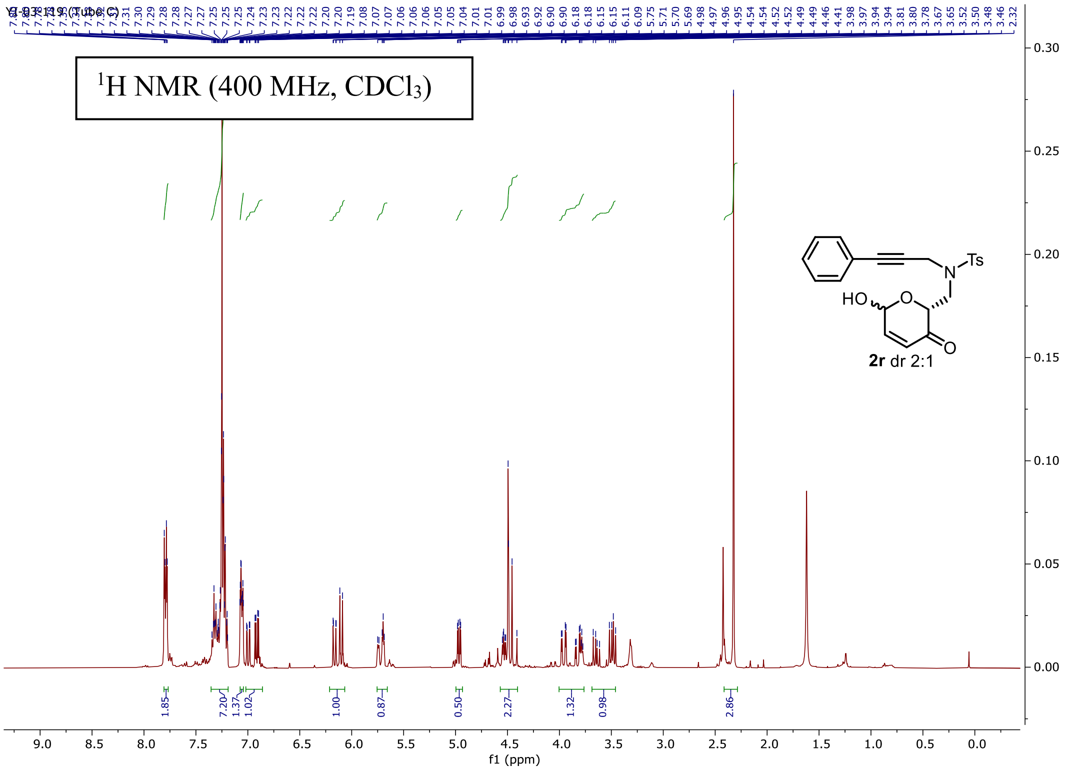

^{1}H NMR (400 MHz, CDCl$_3$)
HO
O
N
Ts
O
2r dr 2:1
f1 (ppm)

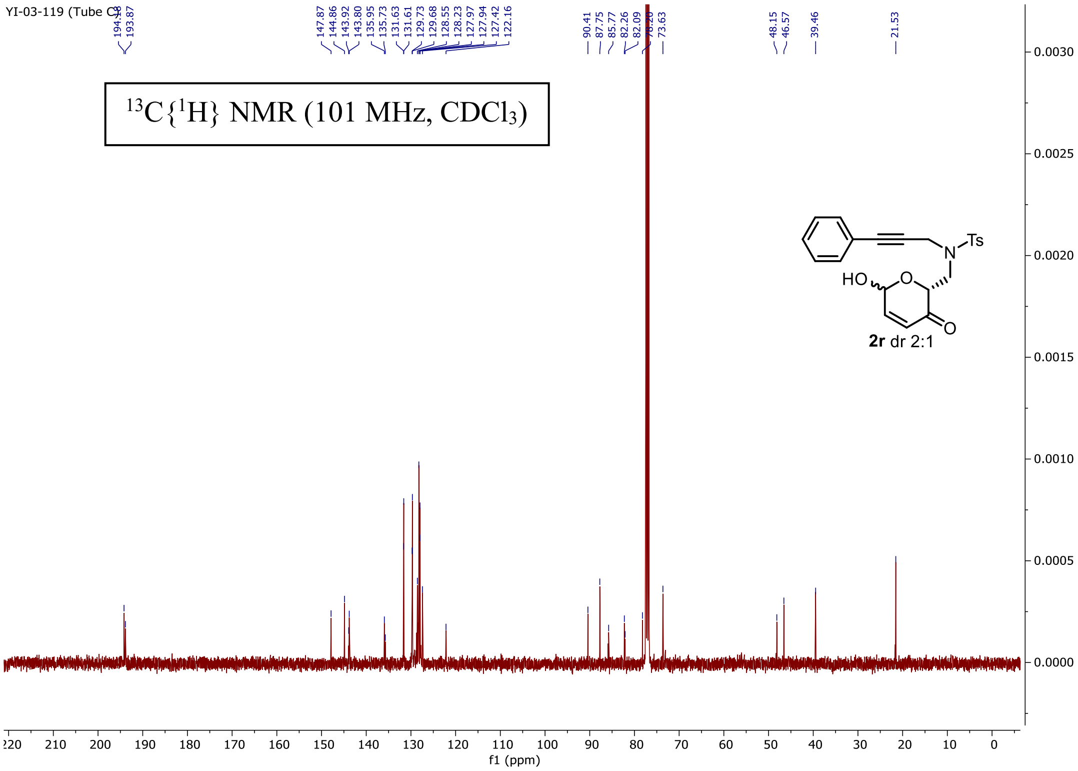

YI-03-119 (Tube C)
^{13}C{^{1}H} NMR (101 MHz, CDCl$_3$)
HO
O
N
Ts
O
2r dr 2:1
f1 (ppm)

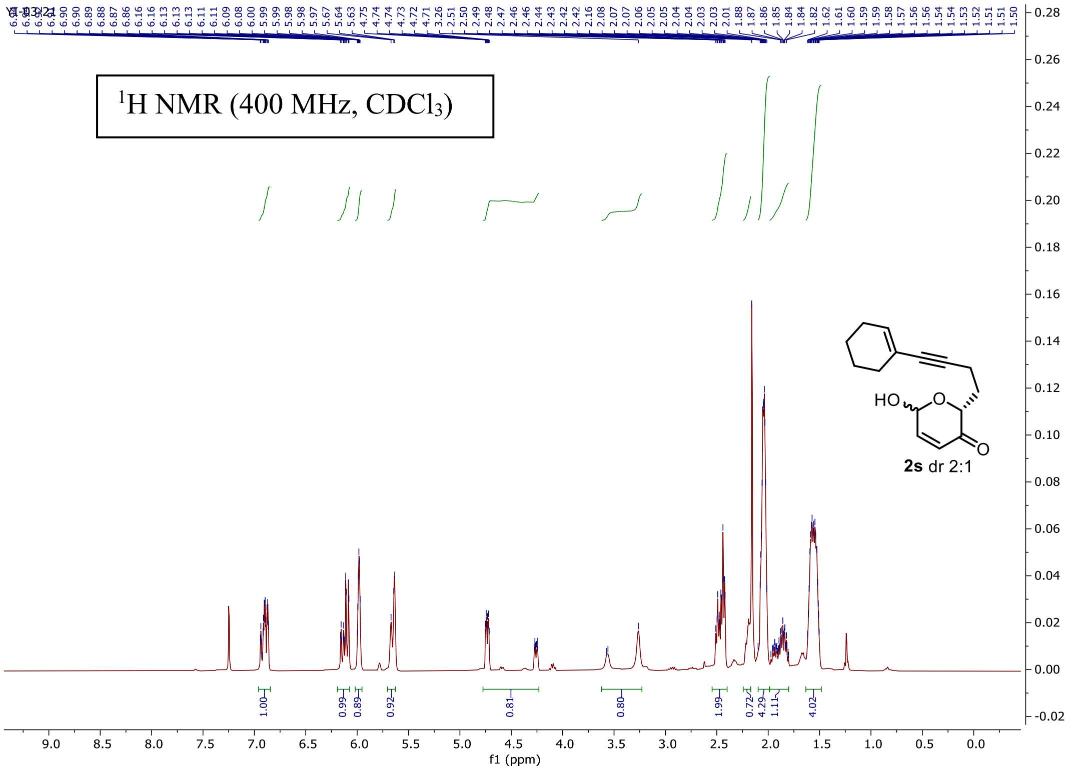

YI-03-21
1H NMR (400 MHz, CDCl3)
HO
O
O
2s dr 2:1
1.00
0.99
0.89
0.92
0.81
0.80
1.99
0.72
4.29
1.11
4.02
f1 (ppm)

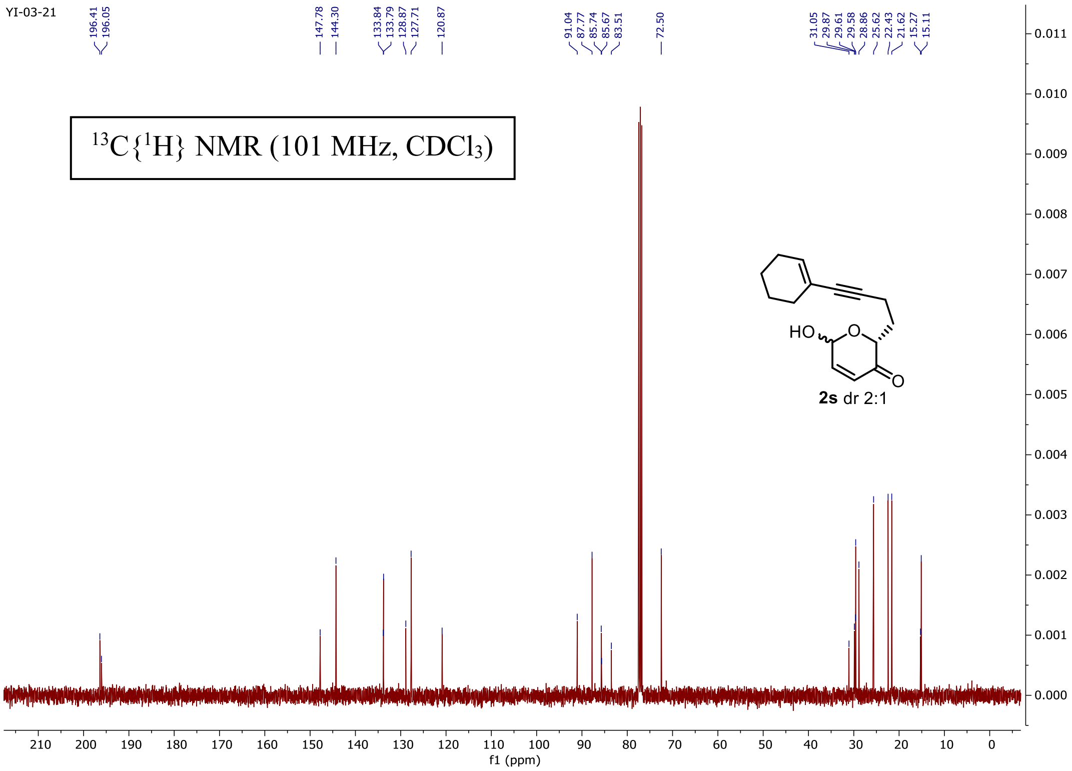

YI-03-21
196.41
196.05
147.78
144.30
133.84
133.79
128.87
127.71
120.87
91.04
87.77
85.74
85.67
83.51
72.50
31.05
29.87
29.61
29.58
28.86
25.62
22.43
21.62
15.27
15.11
13C{1H} NMR (101 MHz, CDCl3)
HO
O
O
2s dr 2:1
f1 (ppm)

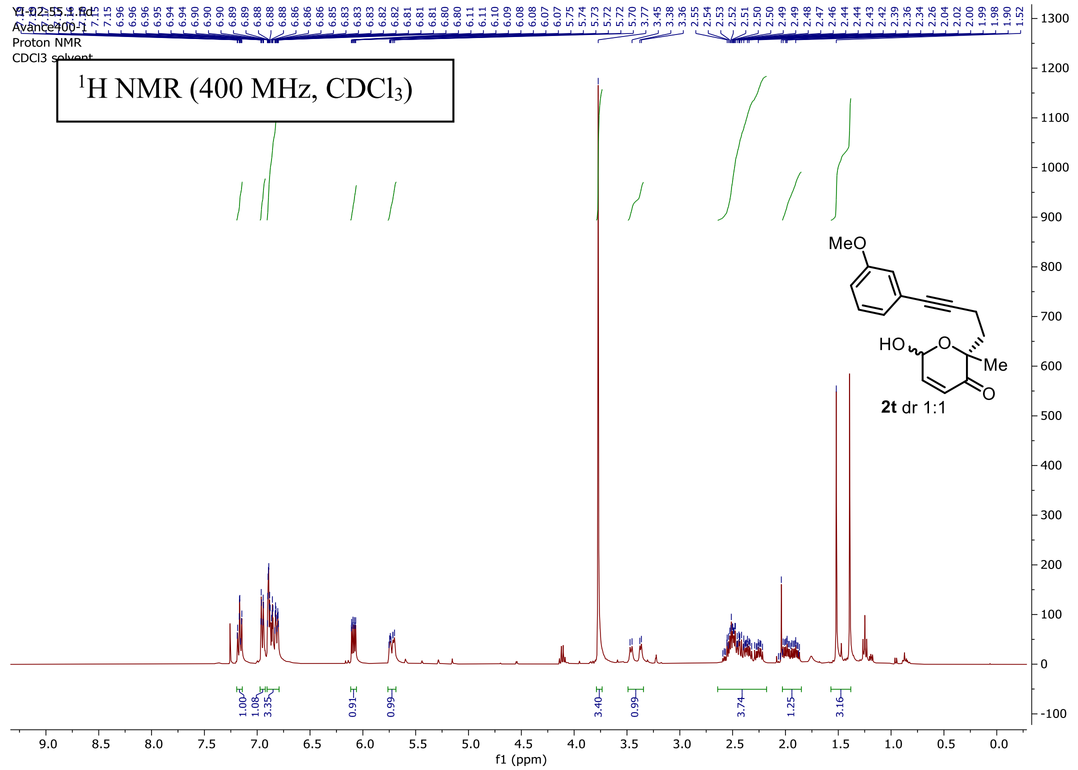

^{1}H NMR (400 MHz, CDCl$_3$)
MeO
HO
O
Me
O
2t dr 1:1
f1 (ppm)

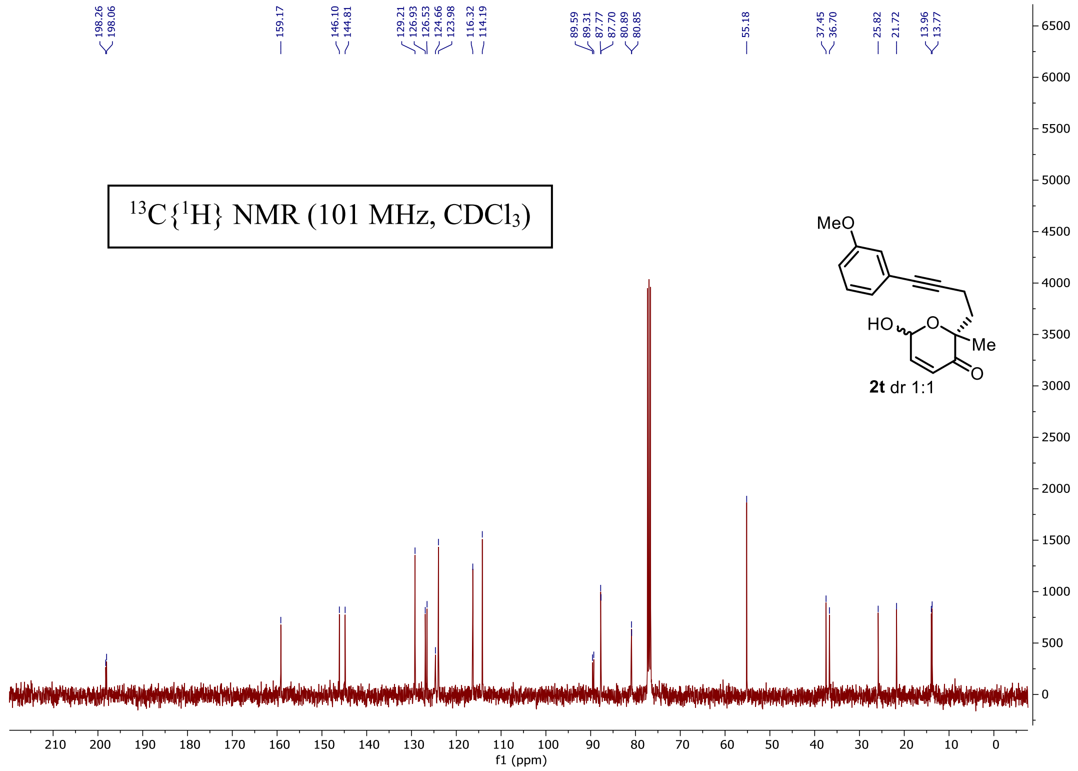

^{13}C{^{1}H} NMR (101 MHz, CDCl$_3$)
198.26
198.06
159.17
146.10
144.81
129.21
126.93
126.53
124.66
123.98
116.32
114.19
89.59
89.31
87.77
87.70
80.89
80.85
55.18
37.45
36.70
25.82
21.72
13.96
13.77
MeO
HO
O
Me
O
2t dr 1:1
f1 (ppm)

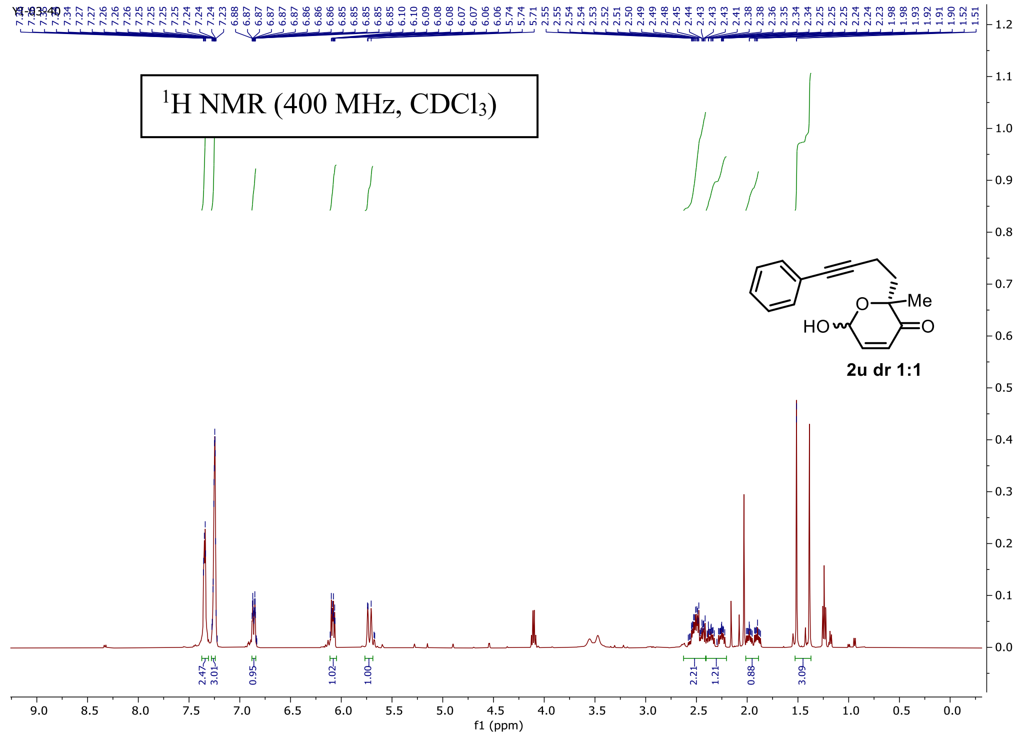
YI-03-40
1H NMR (400 MHz, CDCl3)
Me
HO
O
2u dr 1:1
f1 (ppm)

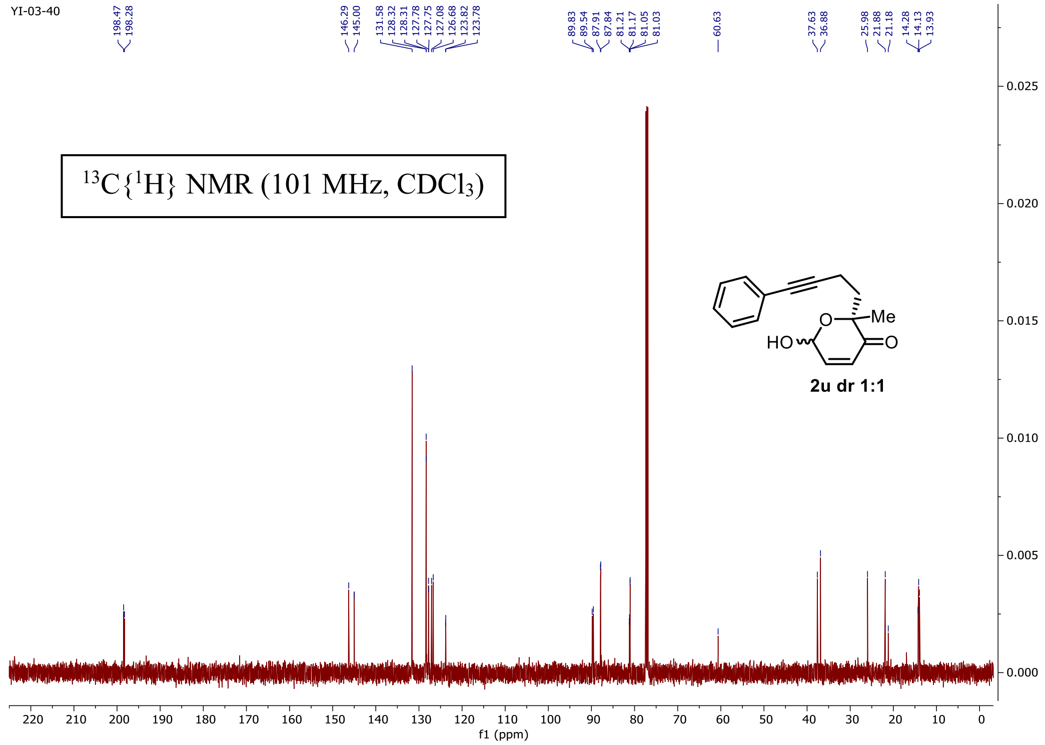
YI-03-40
13C{1H} NMR (101 MHz, CDCl3)
Me
HO
O
2u dr 1:1
f1 (ppm)

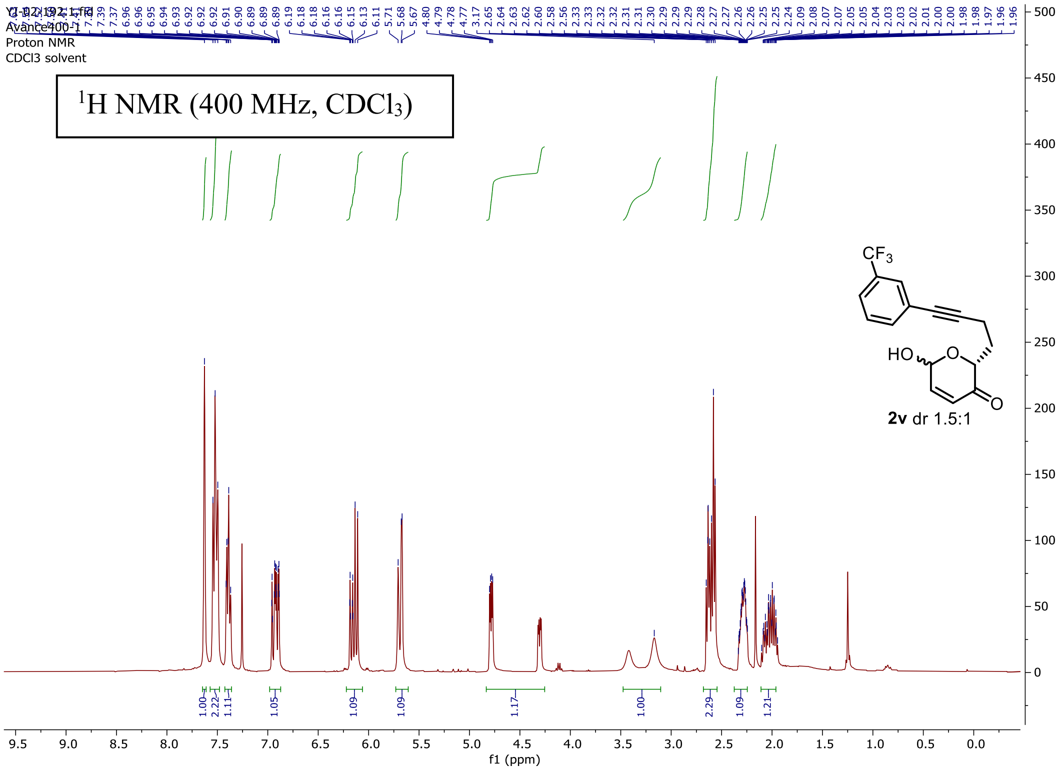

YI-02-192.1.fid
Avance 400-1
Proton NMR
CDCl3 solvent
^{1}H NMR (400 MHz, $CDCl_3$)
CF_3
HO
O
O
2v dr 1.5:1
f1 (ppm)

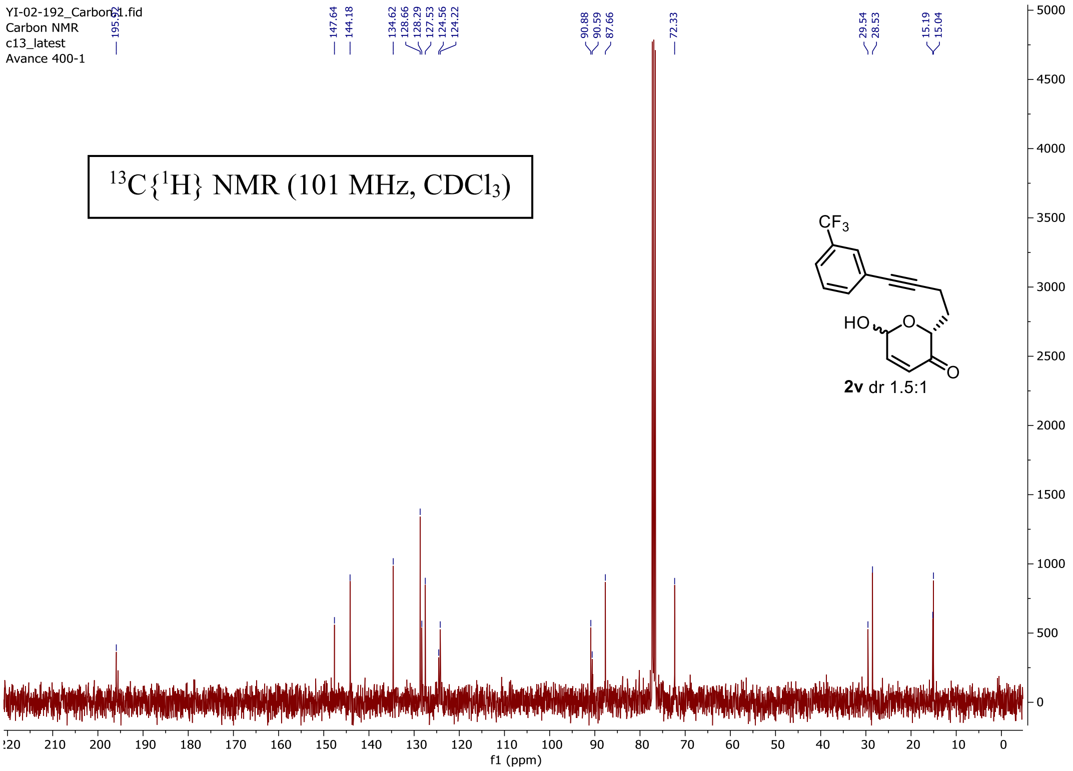

YI-02-192_Carbon.1.fid
Carbon NMR
c13_latest
Avance 400-1
195.52
147.64
144.18
134.62
128.66
128.29
127.53
124.56
124.22
90.88
90.59
87.66
72.33
29.54
28.53
15.19
15.04
^{13}C{^{1}H} NMR (101 MHz, $CDCl_3$)
CF_3
HO
O
O
2v dr 1.5:1
f1 (ppm)

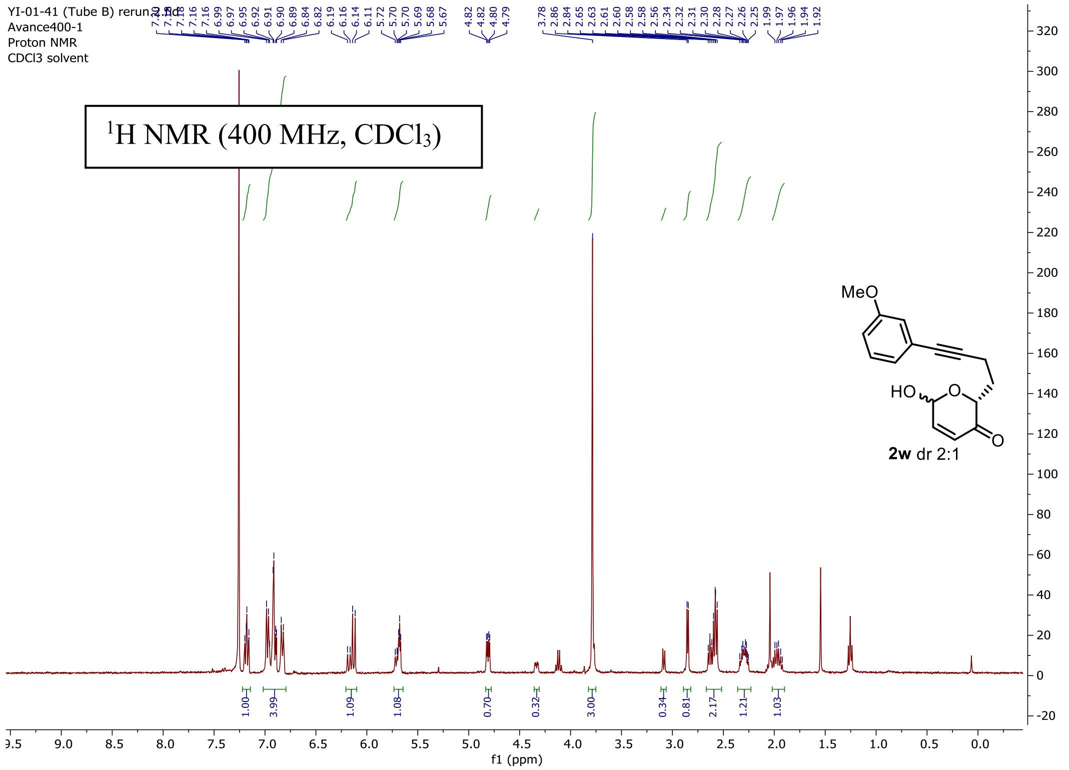
YI-01-41 (Tube B) rerun2.fid
Avance400-1
Proton NMR
CDCl3 solvent
^{1}H NMR (400 MHz, CDCl$_3$)
MeO
HO
O
O
2w dr 2:1
f1 (ppm)

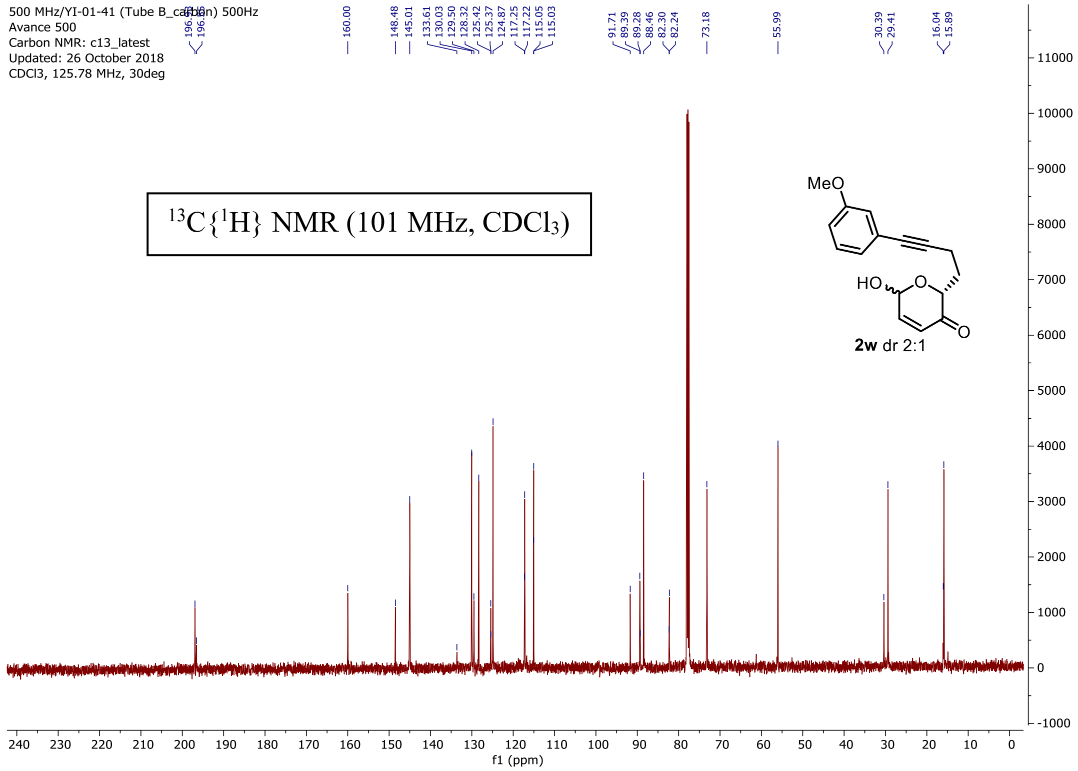
500 MHz/YI-01-41 (Tube B_carbon) 500Hz
Avance 500
Carbon NMR: c13_latest
Updated: 26 October 2018
CDCl3, 125.78 MHz, 30deg
^{13}C{^{1}H} NMR (101 MHz, CDCl$_3$)
MeO
HO
O
O
2w dr 2:1
f1 (ppm)

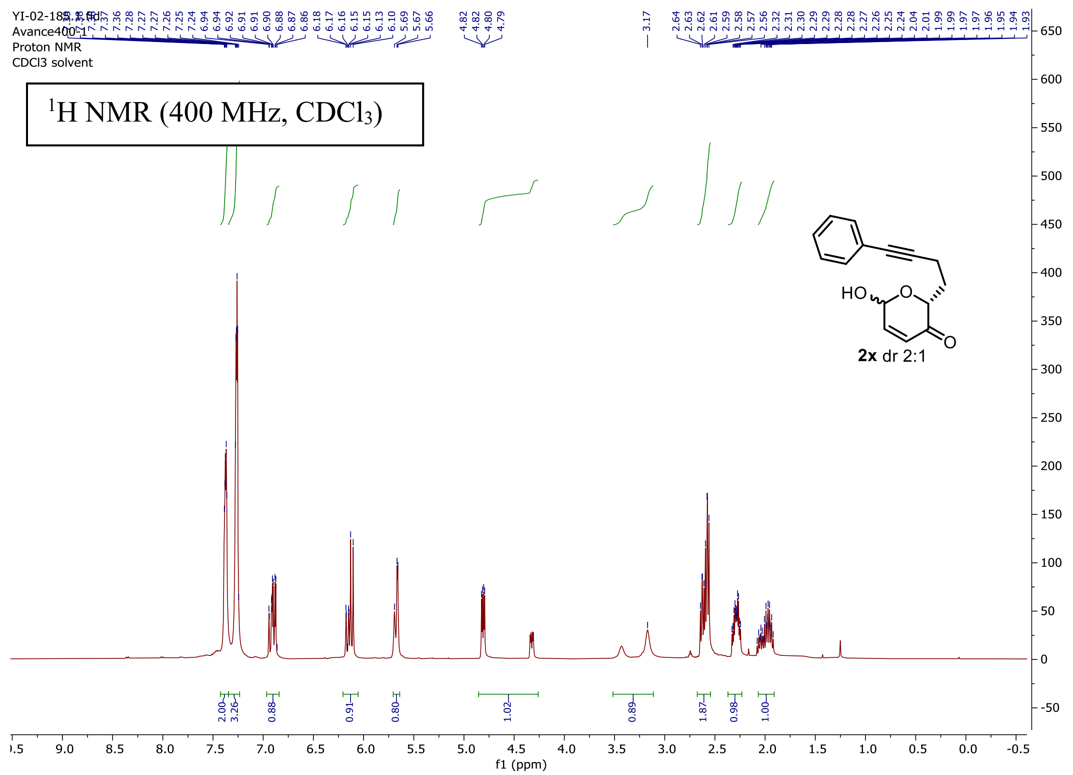

YI-02-185.1.fid
Avance400-1
Proton NMR
CDCl3 solvent
¹H NMR (400 MHz, CDCl₃)
HO
O
O
2x dr 2:1
f1 (ppm)

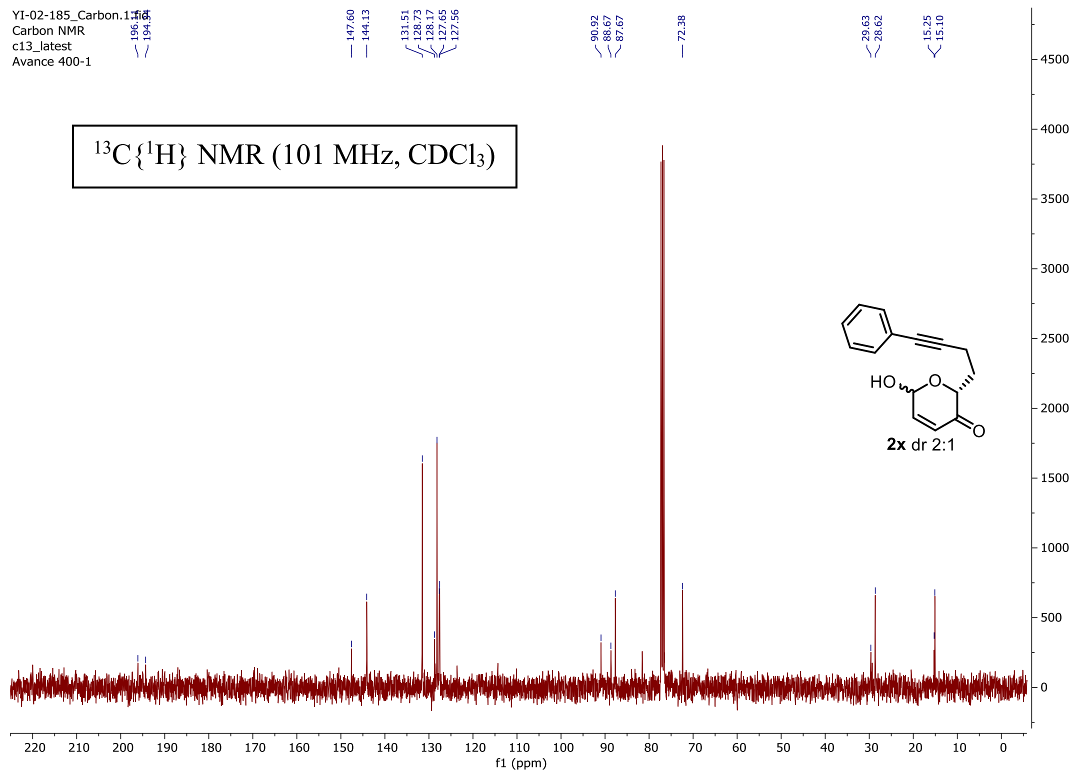

YI-02-185_Carbon.1.fid
Carbon NMR
c13_latest
Avance 400-1
196.14
194.94
147.60
144.13
131.51
128.73
128.17
127.65
127.56
90.92
88.67
87.67
72.38
29.63
28.62
15.25
15.10
¹³C{¹H} NMR (101 MHz, CDCl₃)
HO
O
O
2x dr 2:1
f1 (ppm)

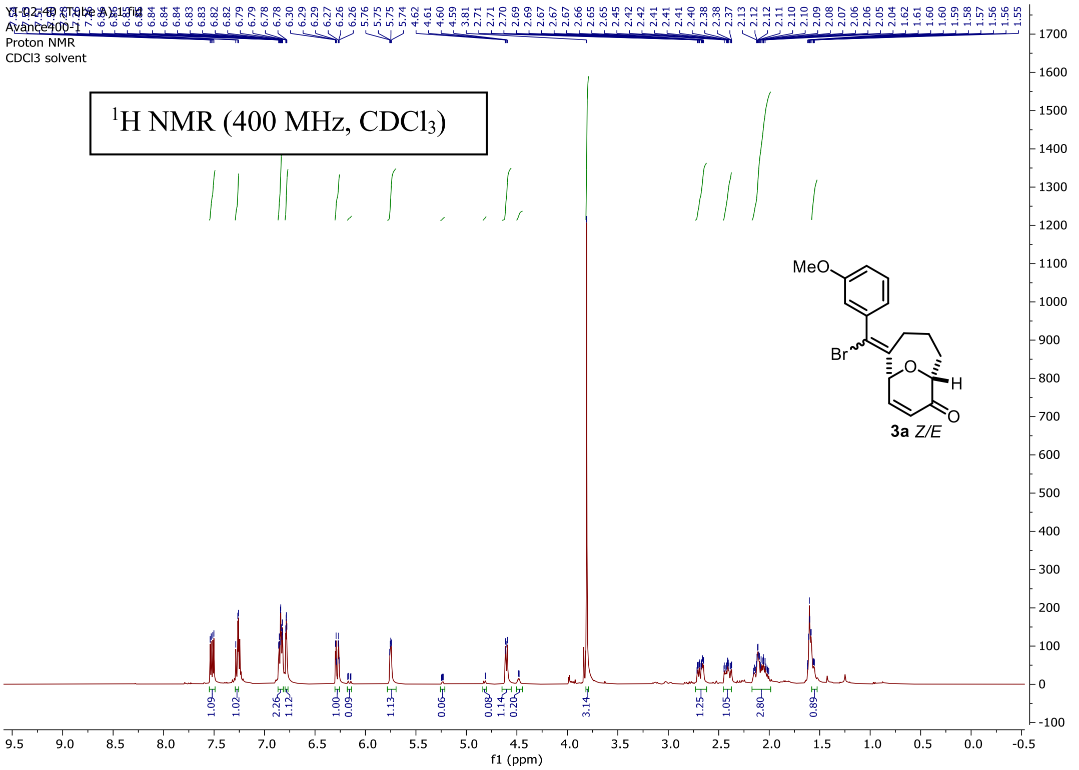
Avance400-1
Proton NMR
CDCl3 solvent
1H NMR (400 MHz, CDCl3)
MeO
Br
O
H
O
3a Z/E
f1 (ppm)

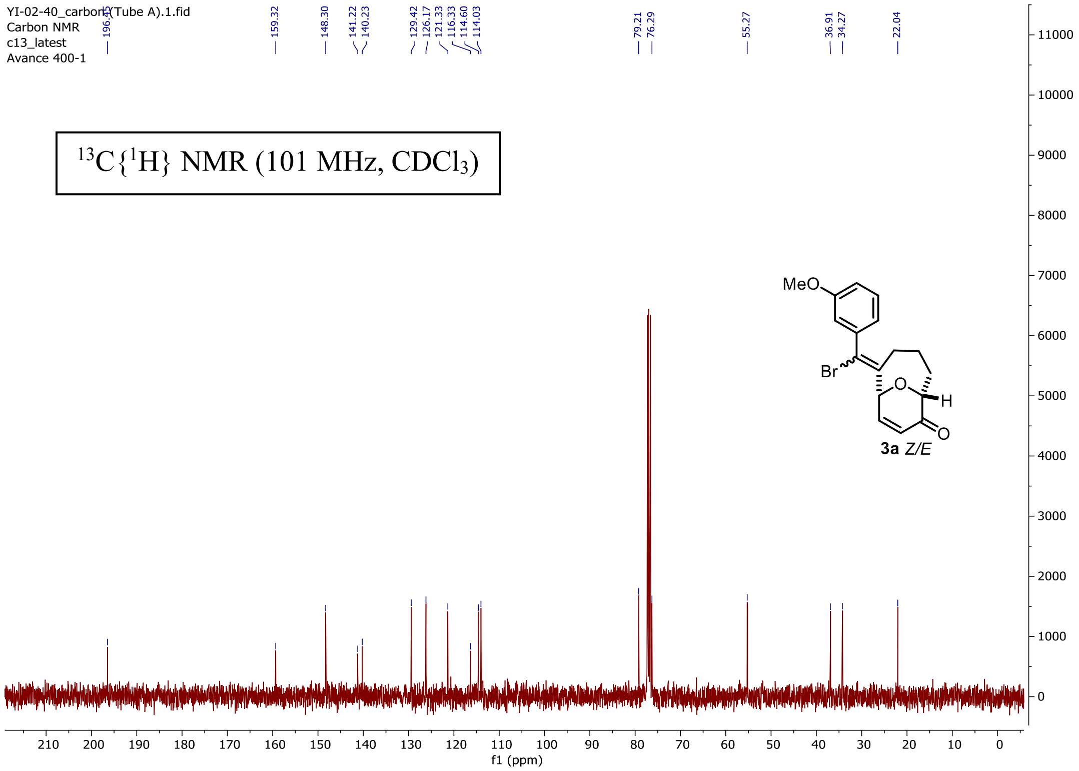
YI-02-40_carbon (Tube A).1.fid
Carbon NMR
c13_latest
Avance 400-1
159.32
148.30
141.22
140.23
129.42
126.17
121.33
116.33
114.60
114.03
79.21
76.29
55.27
36.91
34.27
22.04
13C{1H} NMR (101 MHz, CDCl3)
MeO
Br
O
H
O
3a Z/E
f1 (ppm)

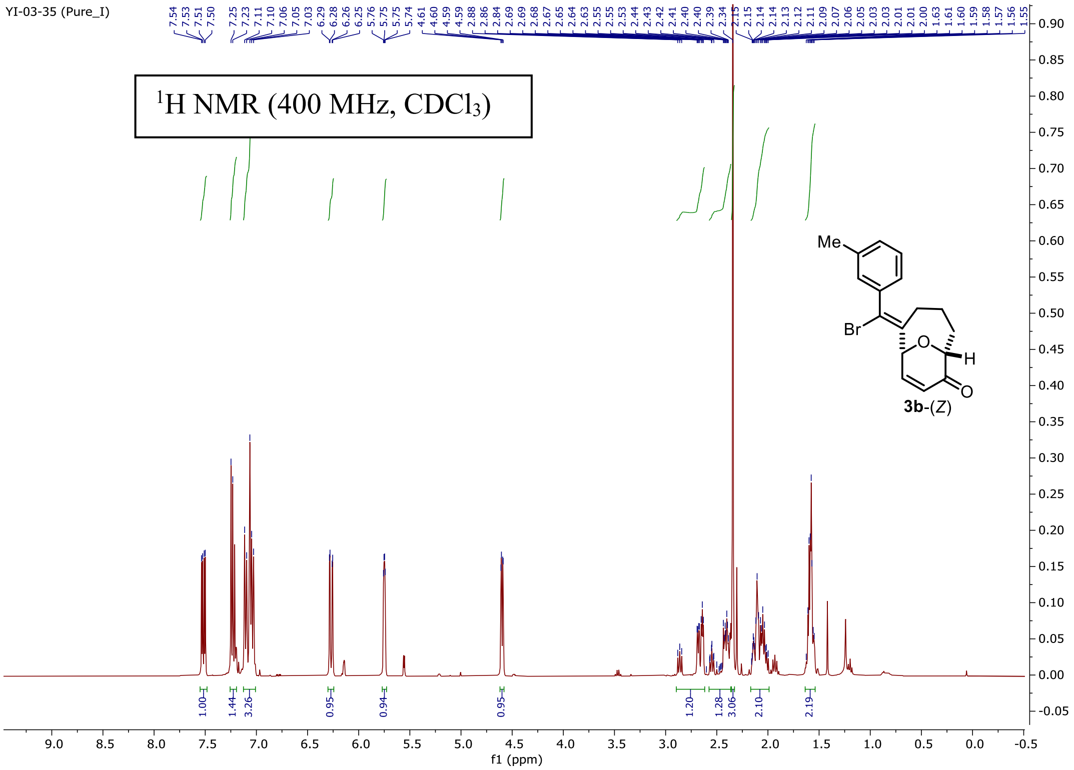
YI-03-35 (Pure_I)
1H NMR (400 MHz, CDCl3)
7.54 7.53 7.51 7.50 7.25 7.23 7.11 7.10 7.06 7.05 7.03 6.29 6.28 6.26 6.25 5.76 5.75 5.75 5.74 4.61 4.60 4.59 4.59 2.88 2.86 2.84 2.69 2.69 2.68 2.67 2.65 2.64 2.63 2.55 2.55 2.53 2.44 2.43 2.42 2.41 2.40 2.40 2.39 2.34 2.15 2.14 2.14 2.13 2.12 2.11 2.09 2.07 2.06 2.05 2.03 2.03 2.01 2.01 2.00 1.63 1.61 1.60 1.59 1.58 1.57 1.56 1.55
1.00 1.44 3.26 0.95 0.94 0.95 1.20 1.28 3.06 2.10 2.19
Me
Br
O
H
O
3b-(Z)
f1 (ppm)

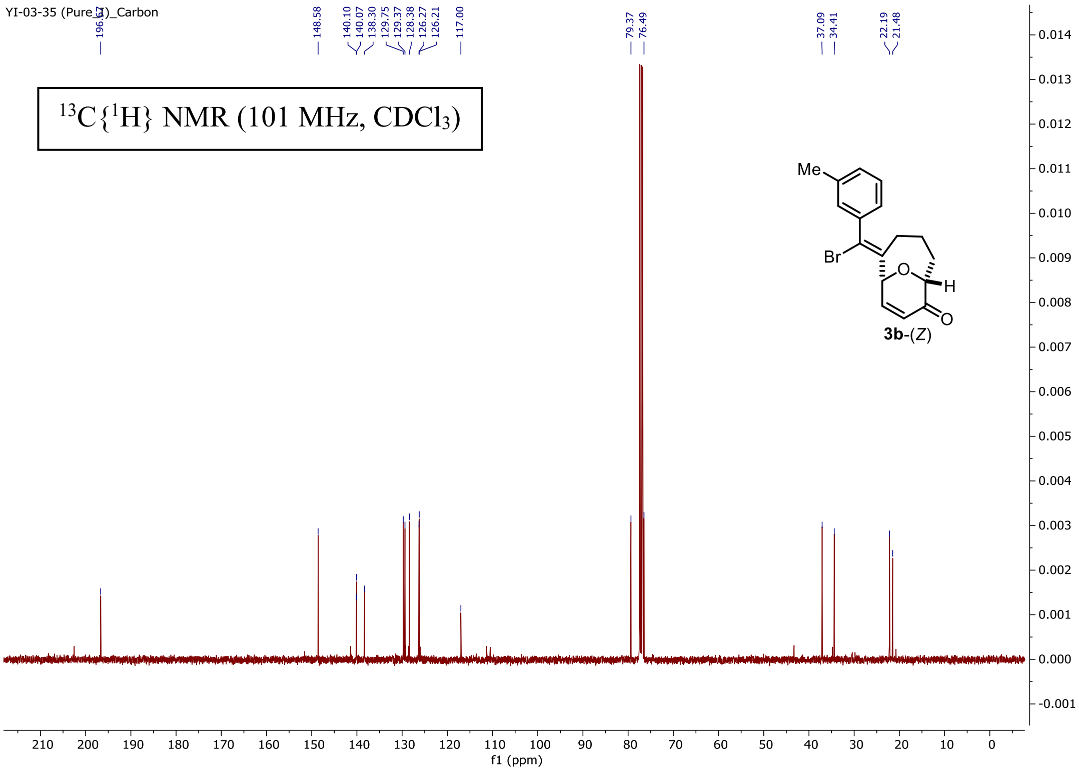
YI-03-35 (Pure_I)_Carbon
148.58 140.10 140.07 138.30 129.75 129.37 128.38 126.27 126.21 117.00 79.37 76.49 37.09 34.41 22.19 21.48
13C{1H} NMR (101 MHz, CDCl3)
Me
Br
O
H
O
3b-(Z)
f1 (ppm)

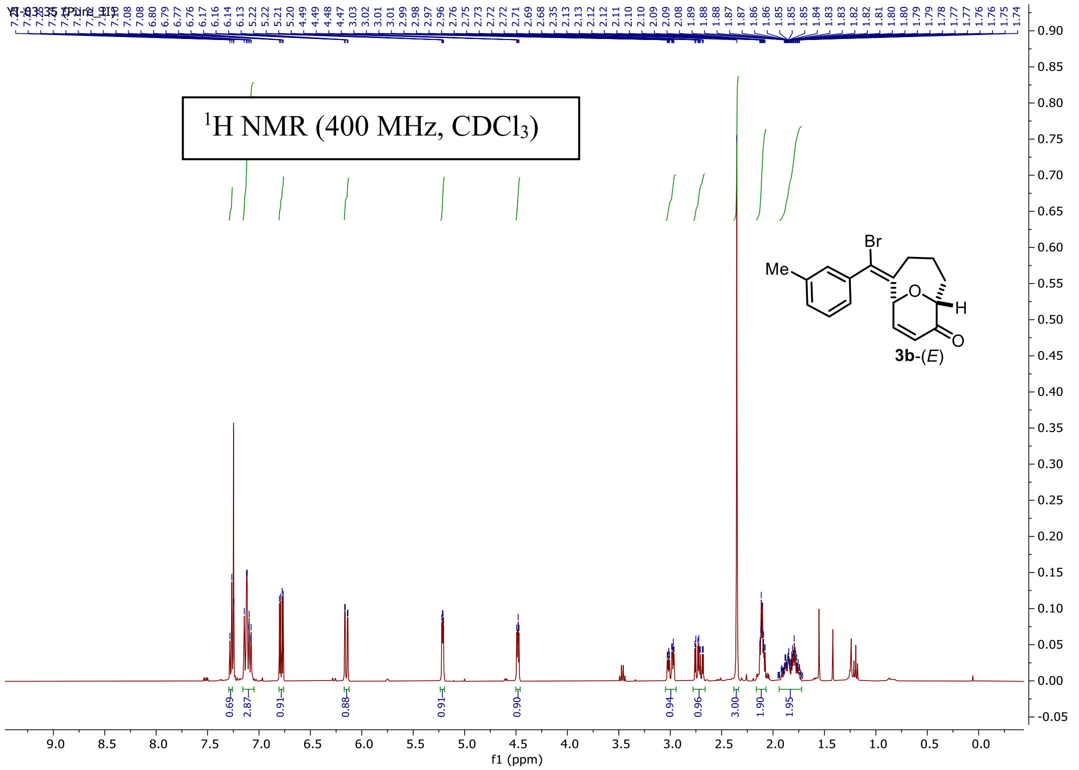

YI-03-35 (Pure_II)
^{1}H NMR (400 MHz, $CDCl_3$)
Br
Me
O
H
O
3b-(E)
f1 (ppm)

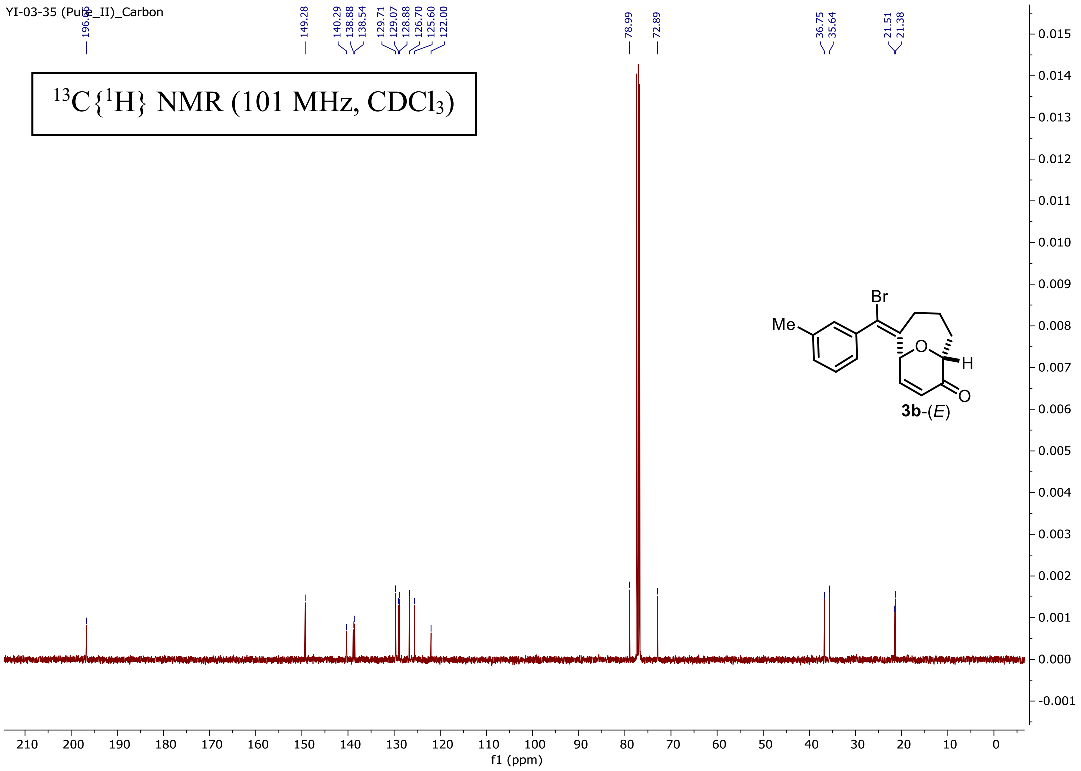

YI-03-35 (Pure_II)_Carbon
196.8
149.28
140.29
138.88
138.54
129.71
129.07
128.88
126.70
125.60
122.00
78.99
72.89
36.75
35.64
21.51
21.38
$^{13}C\{^1H\}$ NMR (101 MHz, $CDCl_3$)
Br
Me
O
H
O
3b-(E)
f1 (ppm)

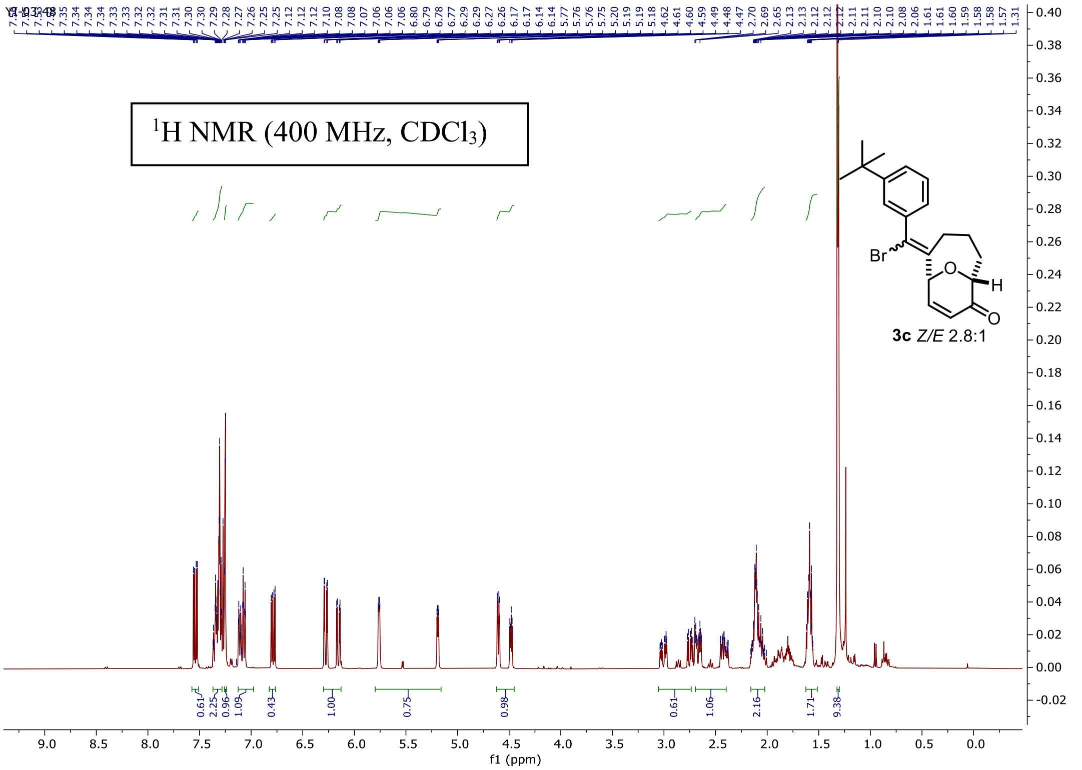
YI-03-48
¹H NMR (400 MHz, CDCl₃)
Br
O
H
O
3c Z/E 2.8:1
f1 (ppm)

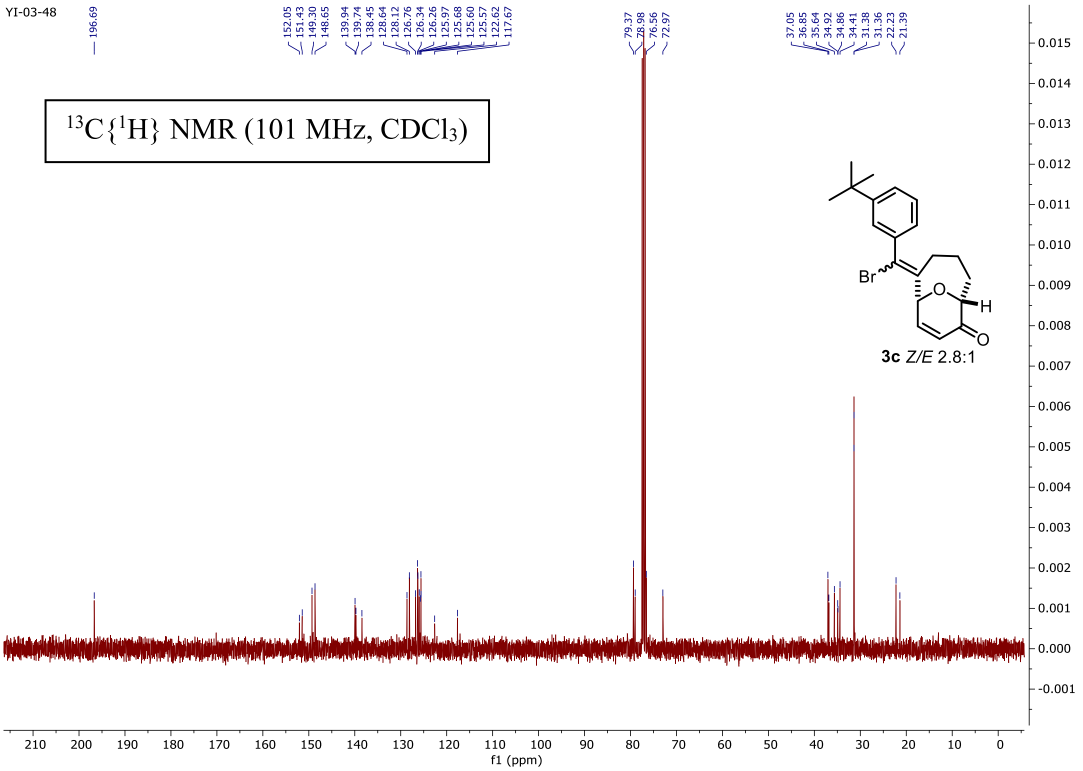
YI-03-48
¹³C{¹H} NMR (101 MHz, CDCl₃)
Br
O
H
O
3c Z/E 2.8:1
f1 (ppm)

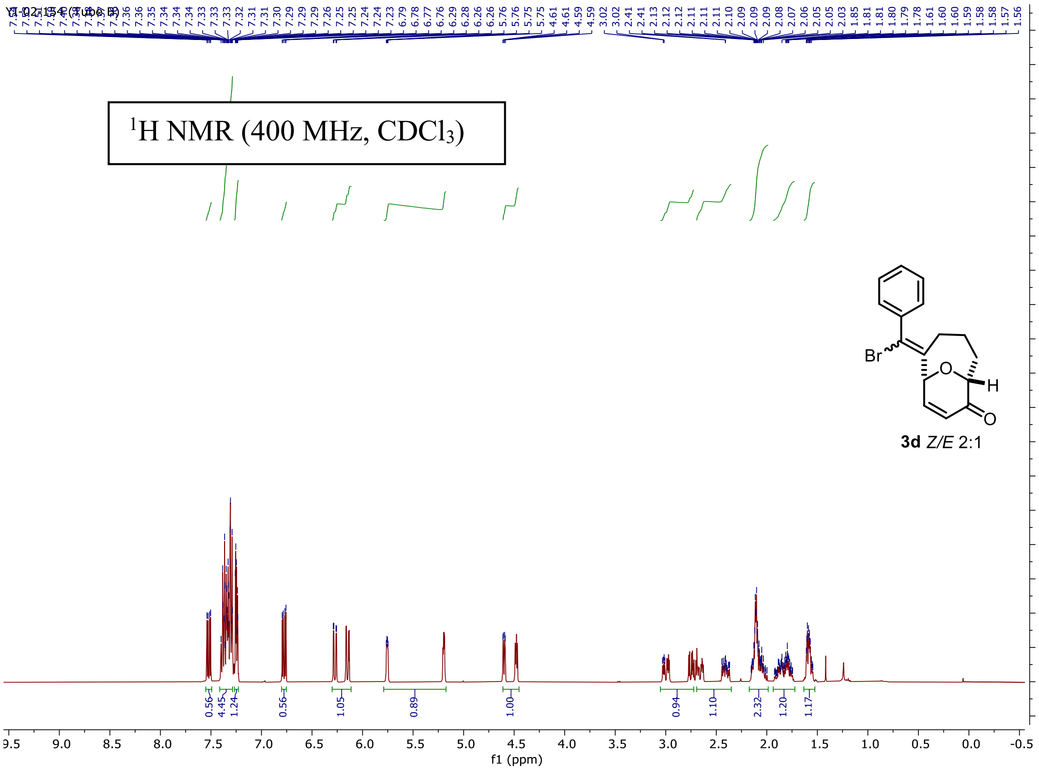
YI-02-154 (Tube B)
1H NMR (400 MHz, CDCl3)
Br
O
H
O
3d Z/E 2:1
f1 (ppm)

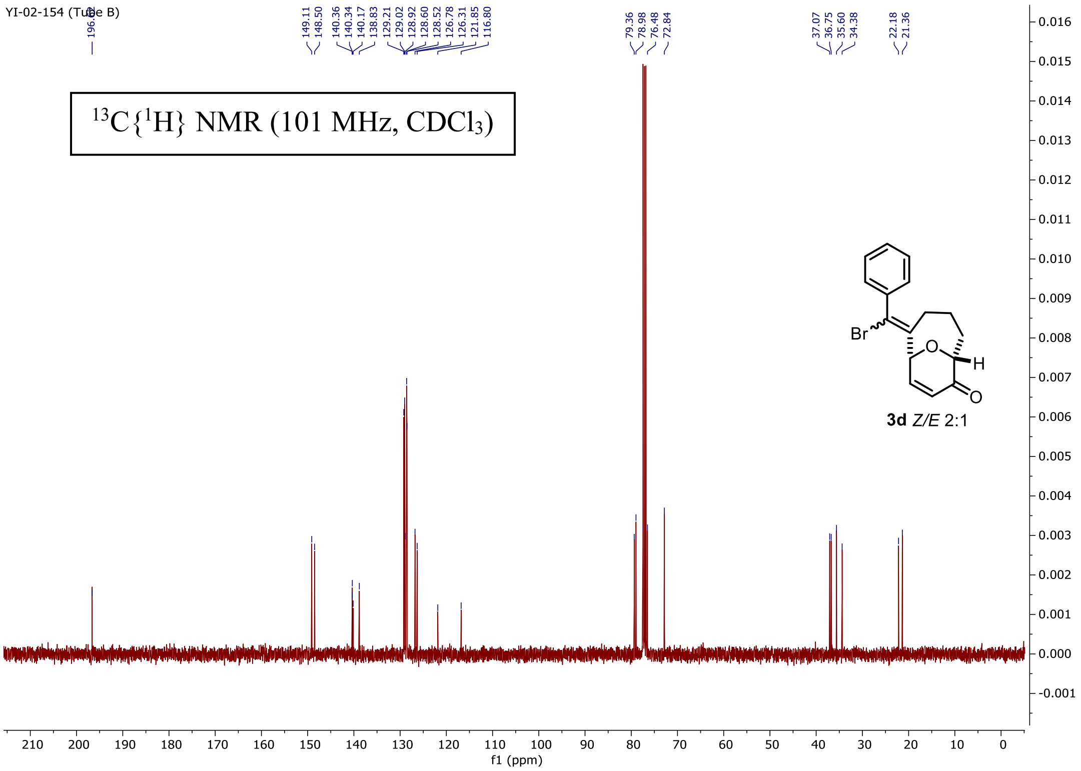
YI-02-154 (Tube B)
13C{1H} NMR (101 MHz, CDCl3)
Br
O
H
O
3d Z/E 2:1
f1 (ppm)

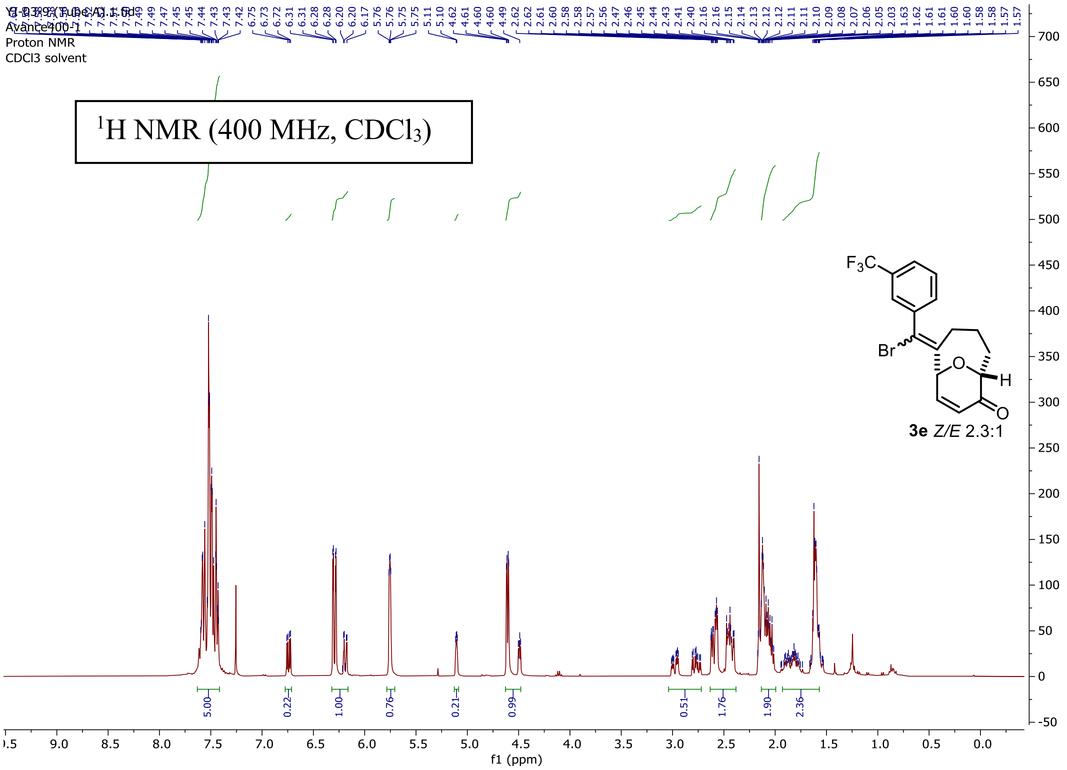
Avance 400-1
Proton NMR
CDCl3 solvent
^{1}H NMR (400 MHz, $CDCl_3$)
3e Z/E 2.3:1
f1 (ppm)

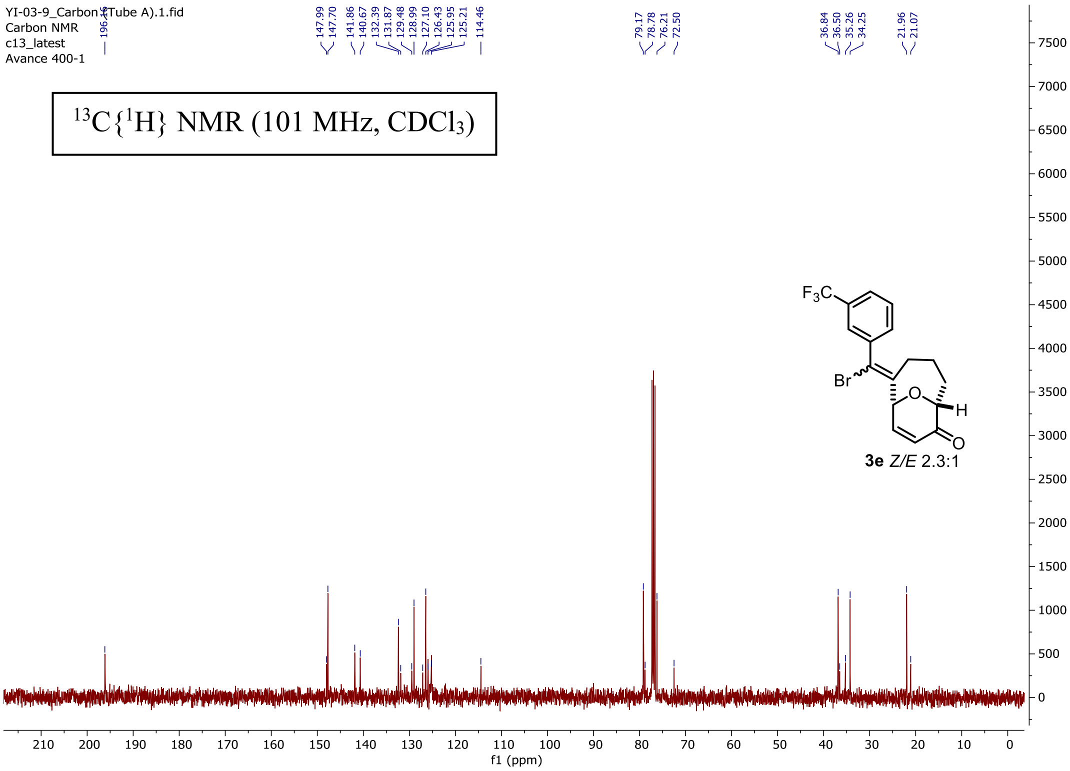
YI-03-9_Carbon (Tube A).1.fid
Carbon NMR
c13_latest
Avance 400-1
196.16
147.99
147.70
141.86
140.67
132.39
131.87
129.48
128.99
127.10
126.43
125.95
125.21
114.46
79.17
78.78
76.21
72.50
36.84
36.50
35.26
34.25
21.96
21.07
^{13}C{^{1}H} NMR (101 MHz, $CDCl_3$)
3e Z/E 2.3:1
f1 (ppm)

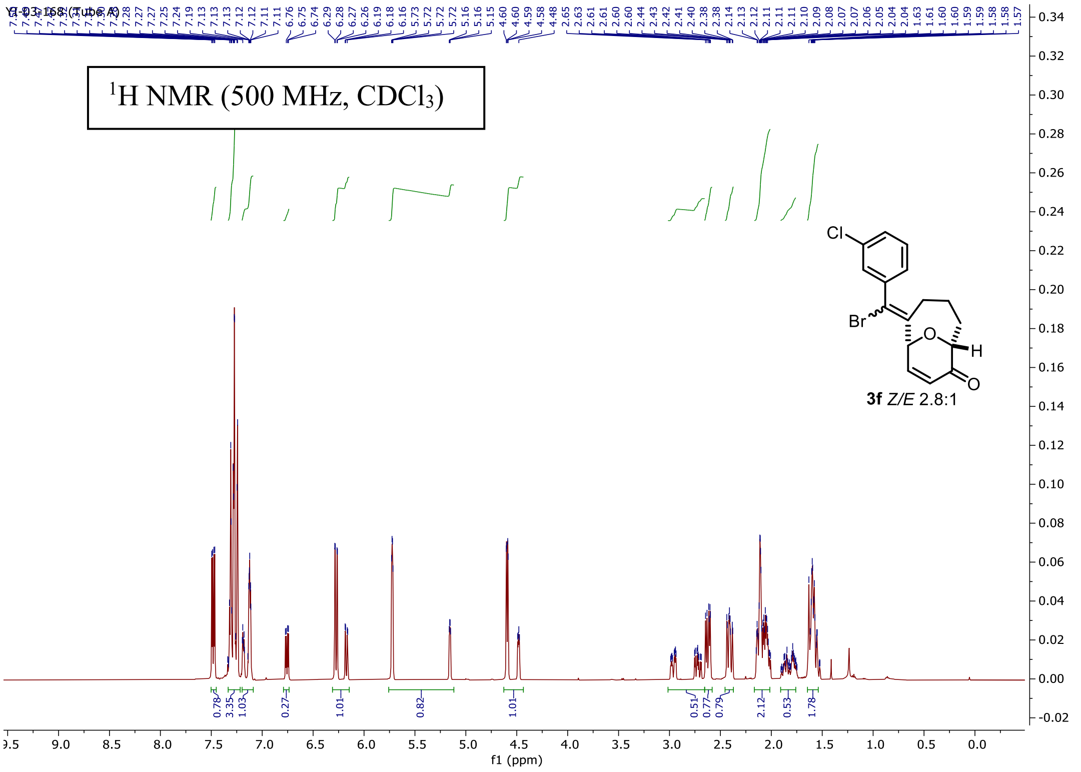
YI-03-168 (Tube A)
1H NMR (500 MHz, CDCl3)
Cl
Br
O
H
O
3f Z/E 2.8:1
f1 (ppm)

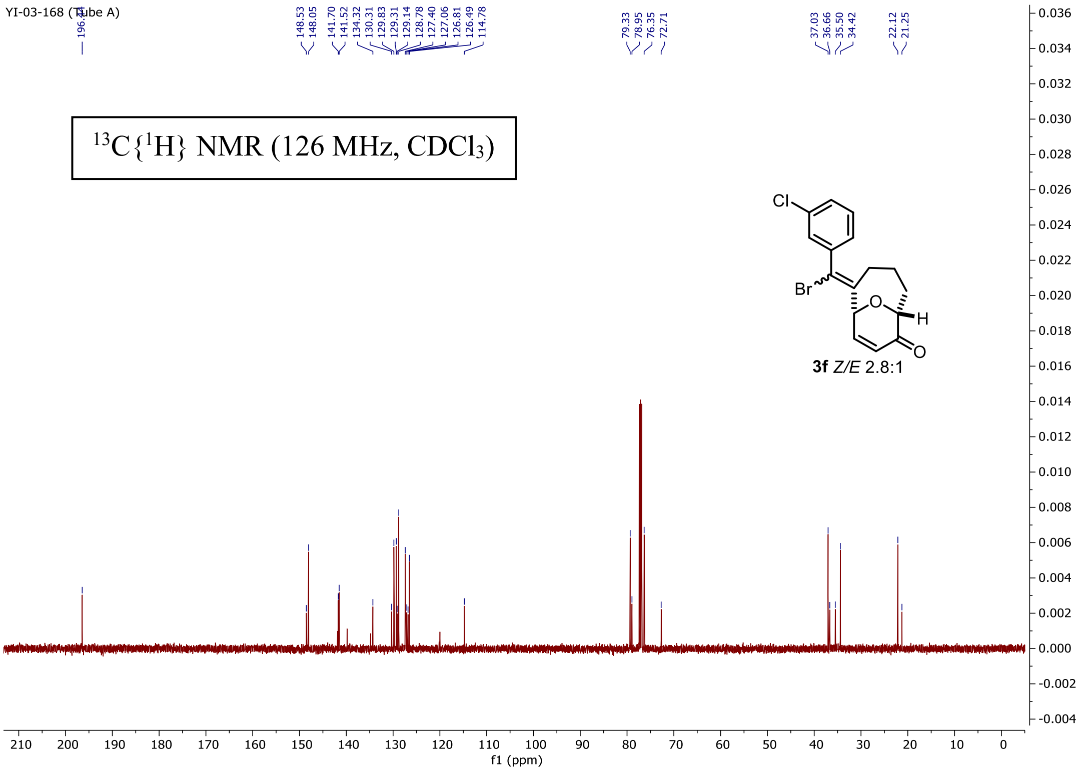
YI-03-168 (Tube A)
13C{1H} NMR (126 MHz, CDCl3)
Cl
Br
O
H
O
3f Z/E 2.8:1
f1 (ppm)

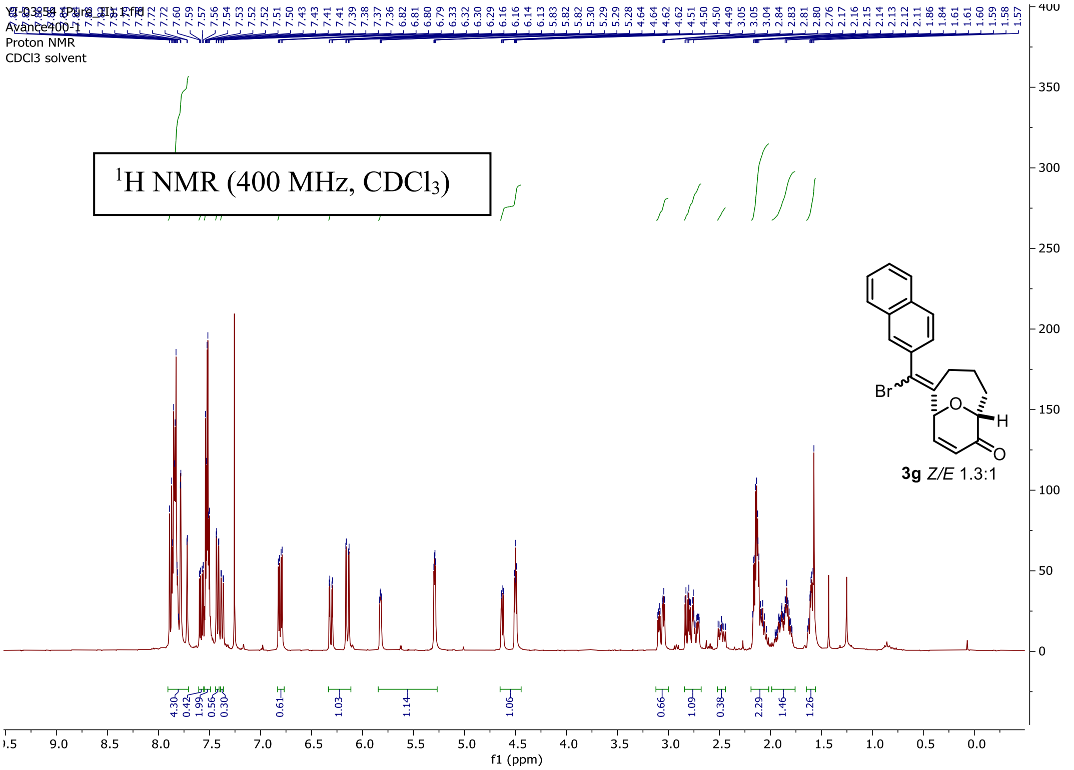
YI-03-54 (Pur8_II) 1.fid
Avance400-1
Proton NMR
CDCl3 solvent
1H NMR (400 MHz, CDCl3)
Br
O
H
O
3g Z/E 1.3:1
f1 (ppm)

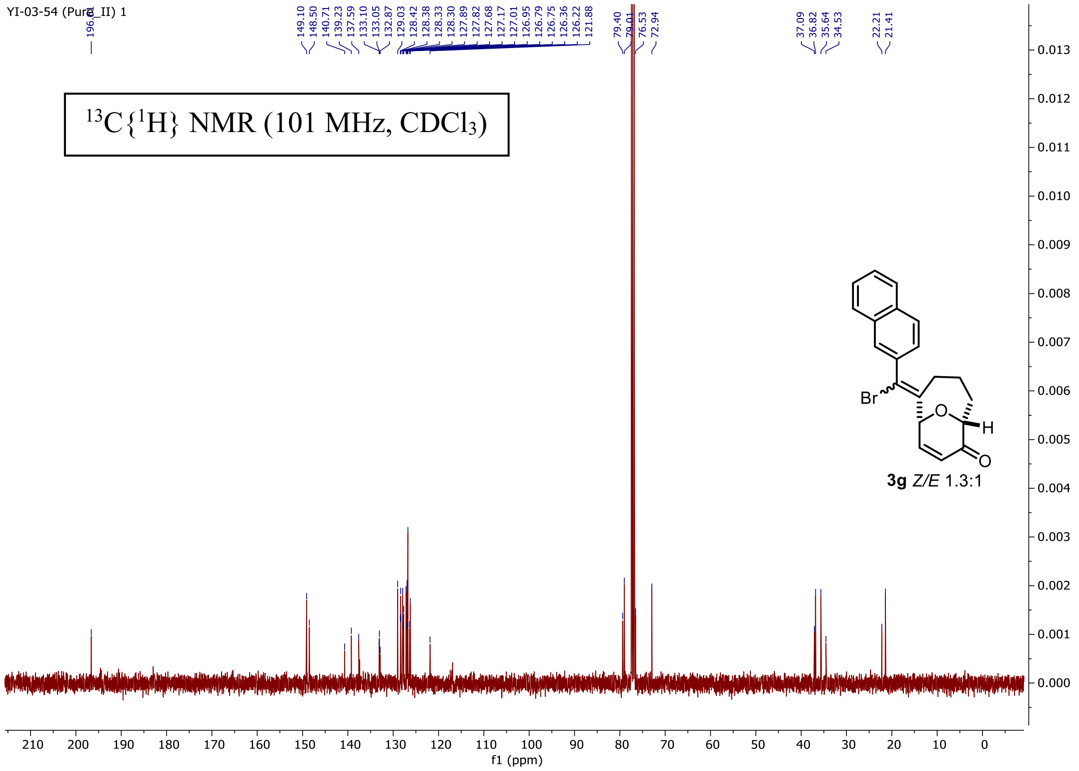
YI-03-54 (Pur8_II) 1
13C{1H} NMR (101 MHz, CDCl3)
Br
O
H
O
3g Z/E 1.3:1
f1 (ppm)

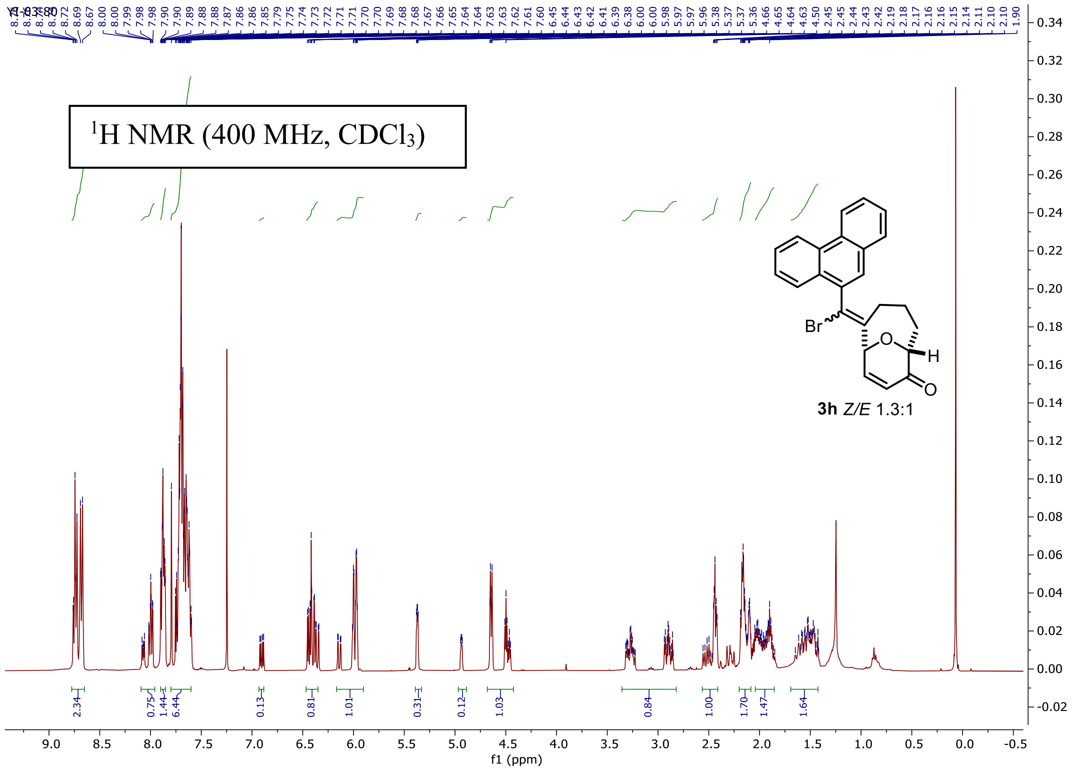
YI-03-80
1H NMR (400 MHz, CDCl3)
Br
O
H
O
3h Z/E 1.3:1
f1 (ppm)

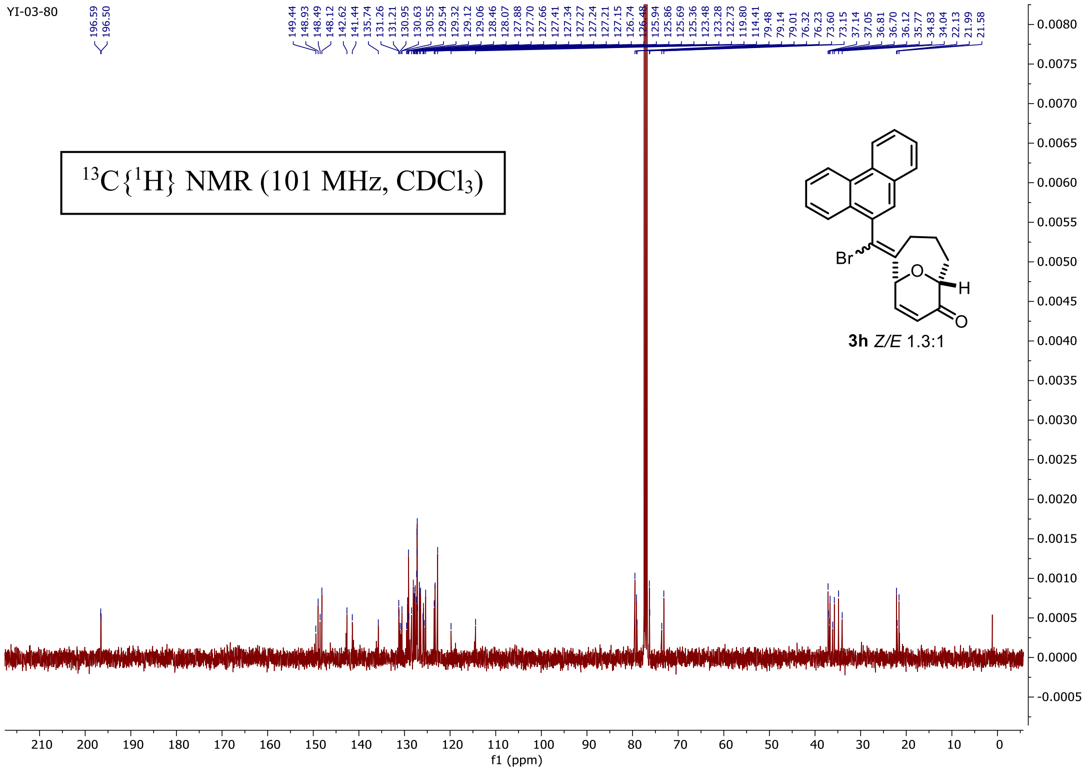
YI-03-80
13C{1H} NMR (101 MHz, CDCl3)
Br
O
H
O
3h Z/E 1.3:1
f1 (ppm)

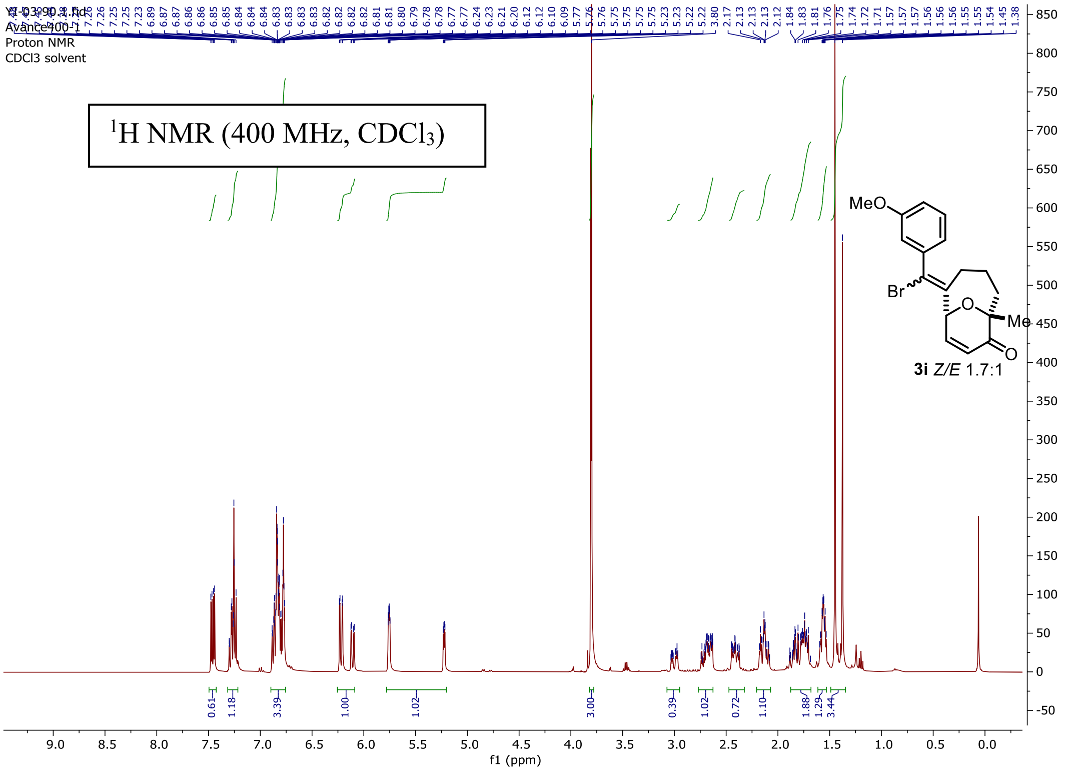
Proton NMR
CDCl3 solvent
1H NMR (400 MHz, CDCl3)
MeO
Br
O
Me
O
3i Z/E 1.7:1
f1 (ppm)

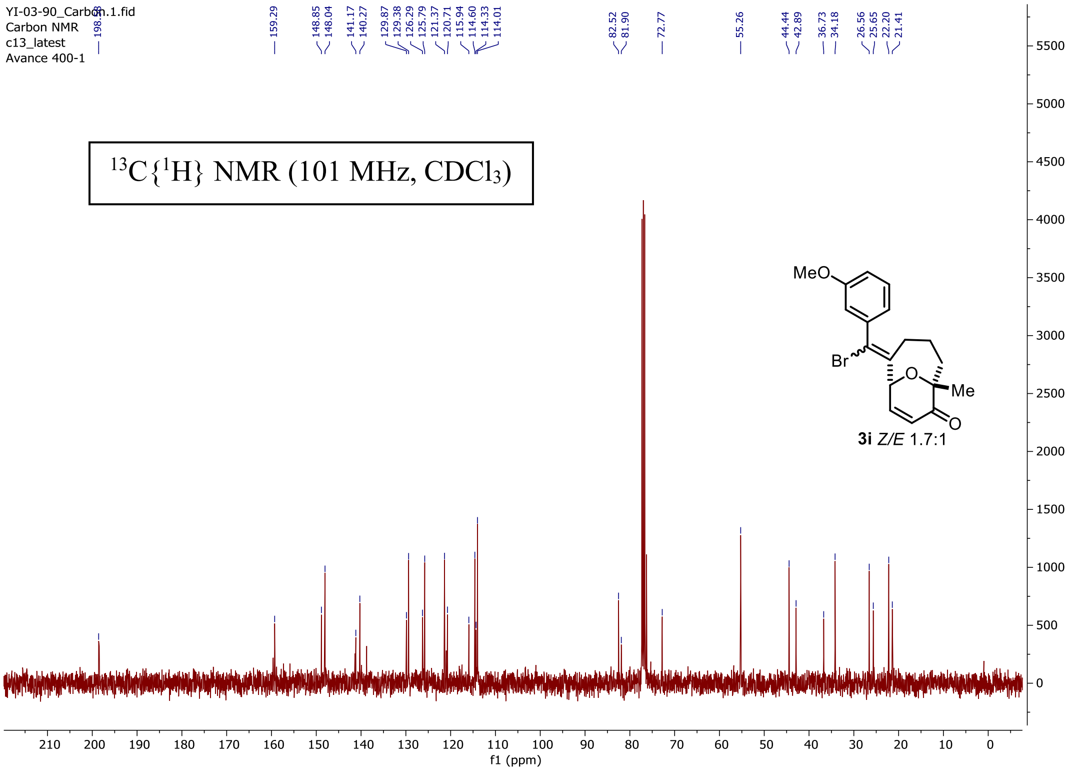
YI-03-90_Carbon.1.fid
Carbon NMR
c13_latest
Avance 400-1
13C{1H} NMR (101 MHz, CDCl3)
MeO
Br
O
Me
O
3i Z/E 1.7:1
f1 (ppm)

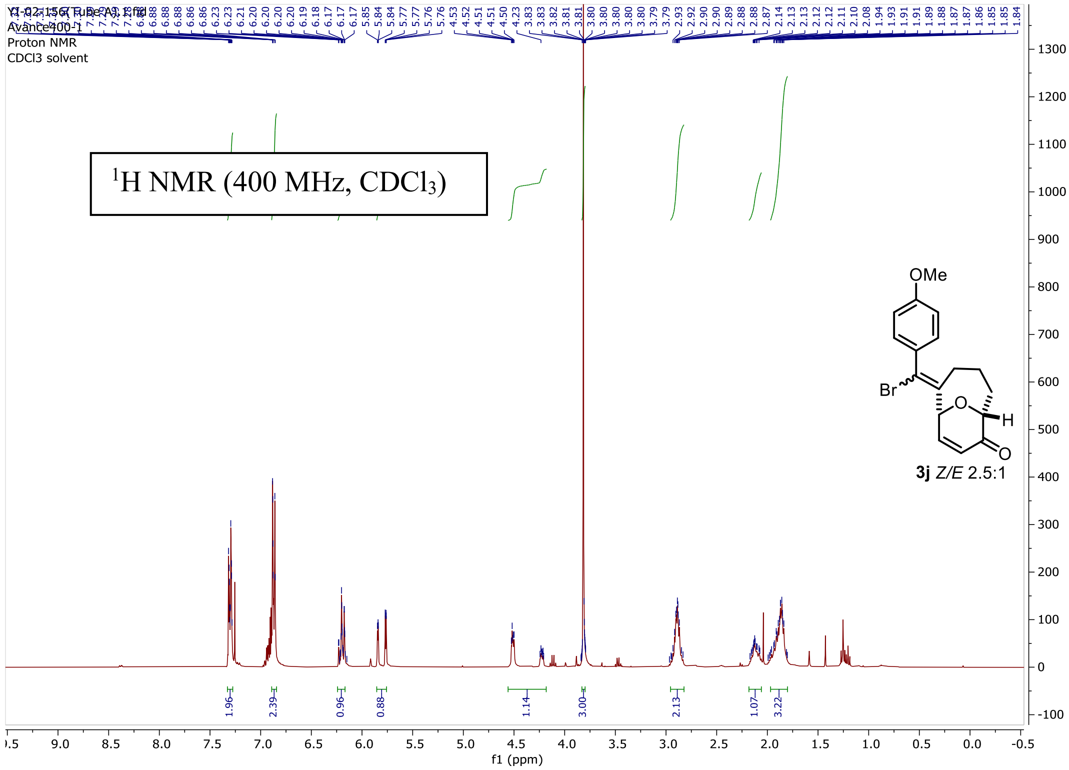

YI-02-156(Tube A)_1.fid
Avance 400-1
Proton NMR
CDCl3 solvent
^{1}H NMR (400 MHz, CDCl$_3$)
OMe
Br
O
H
O
3j Z/E 2.5:1
f1 (ppm)

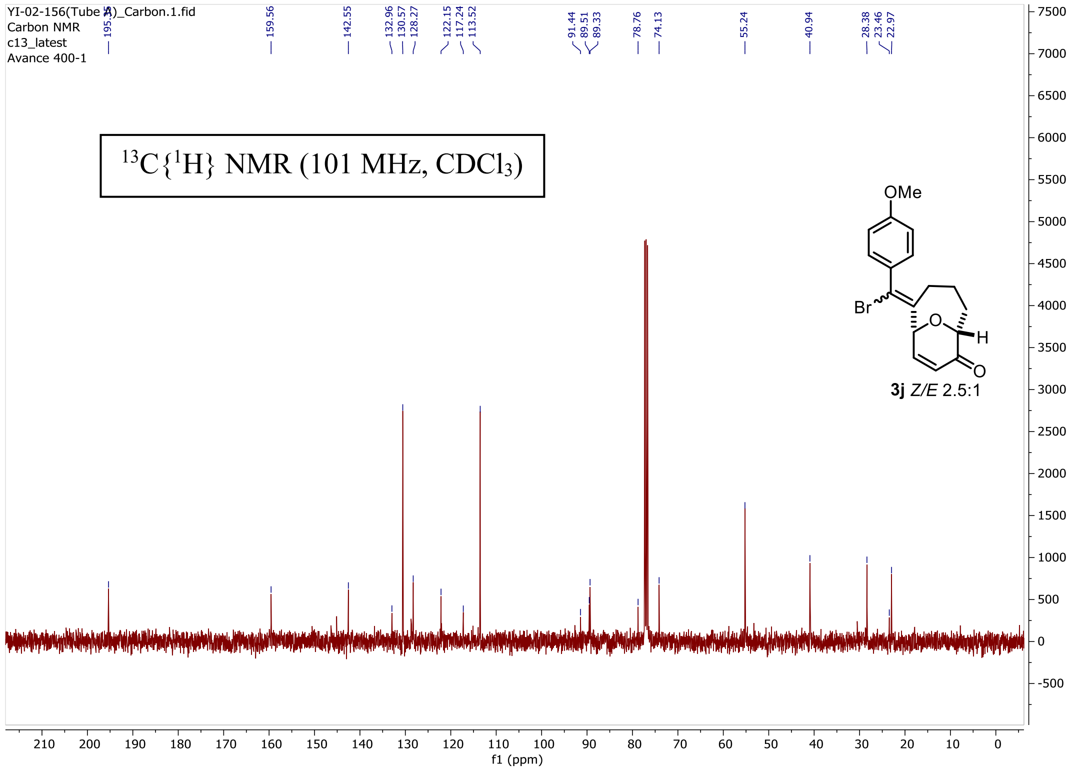

YI-02-156(Tube A)_Carbon.1.fid
Carbon NMR
c13_latest
Avance 400-1
195.5
159.56
142.55
132.96
130.57
128.27
122.15
117.24
113.52
91.44
89.51
89.33
78.76
74.13
55.24
40.94
28.38
23.46
22.97
^{13}C{^{1}H} NMR (101 MHz, CDCl$_3$)
OMe
Br
O
H
O
3j Z/E 2.5:1
f1 (ppm)

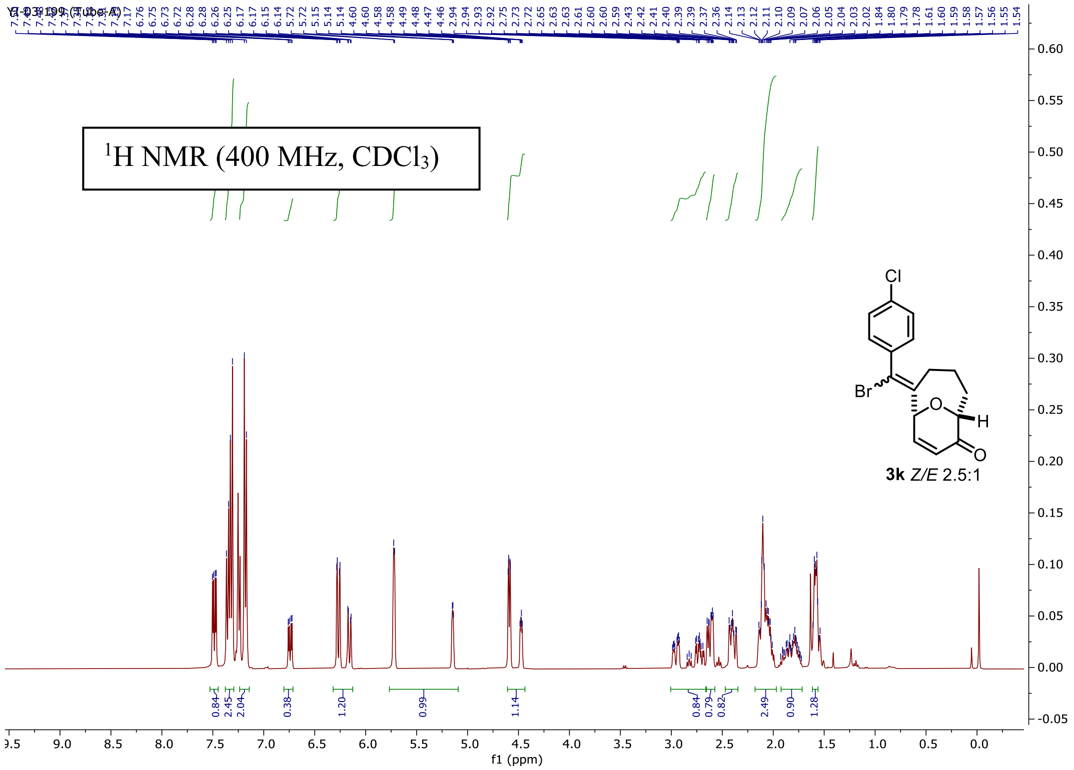
YI-03-199 (Tube-A)
^{1}H NMR (400 MHz, $CDCl_3$)
3k *Z/E* 2.5:1
f1 (ppm)

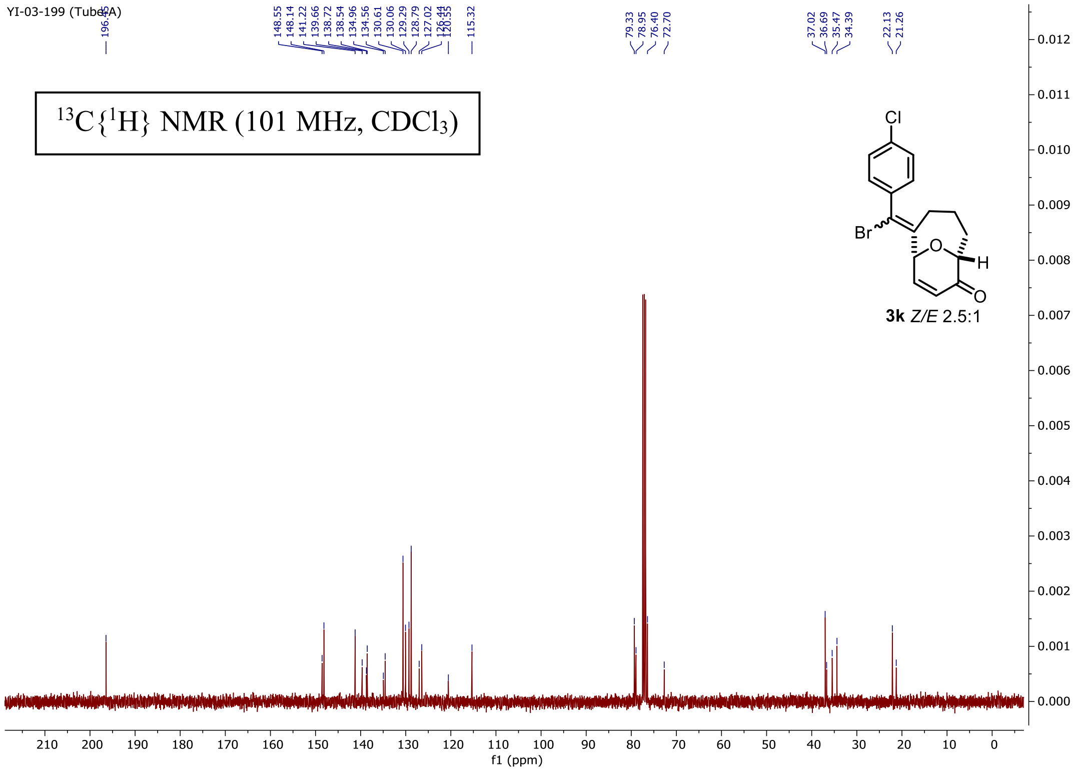
YI-03-199 (Tube-A)
$^{13}C\{^{1}H\}$ NMR (101 MHz, $CDCl_3$)
3k *Z/E* 2.5:1
f1 (ppm)

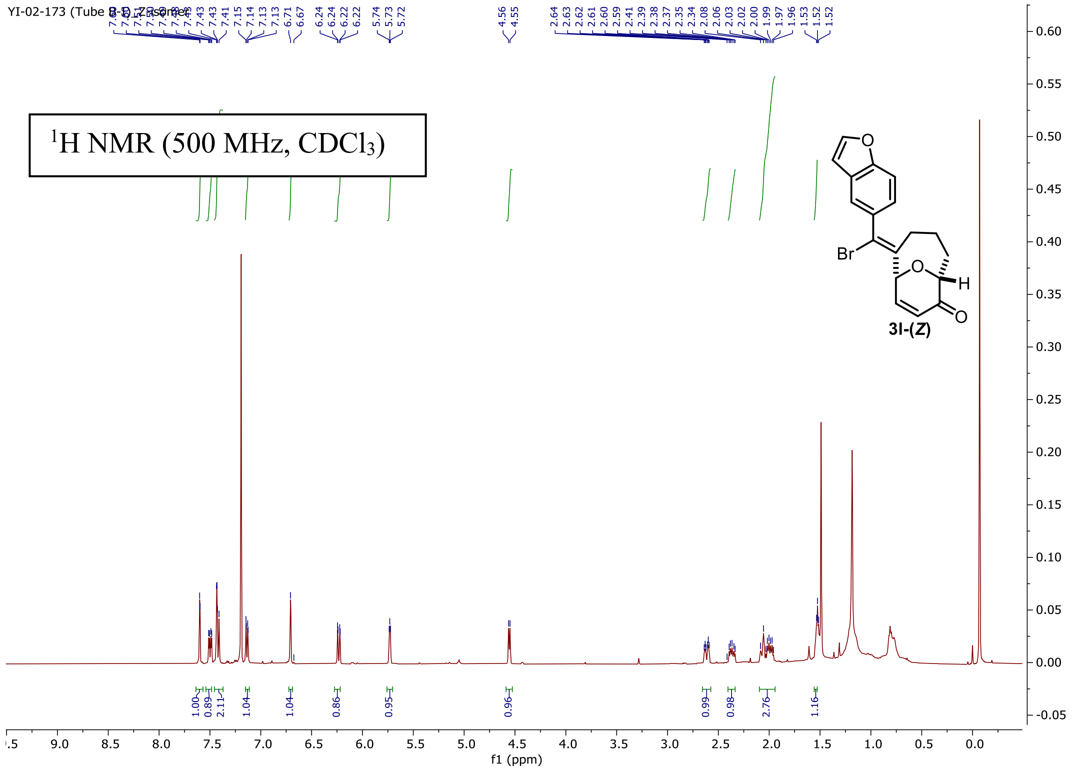
YI-02-173 (Tube B-I)_Z-isomer
^{1}H NMR (500 MHz, CDCl$_3$)
Br
3l-(Z)
f1 (ppm)

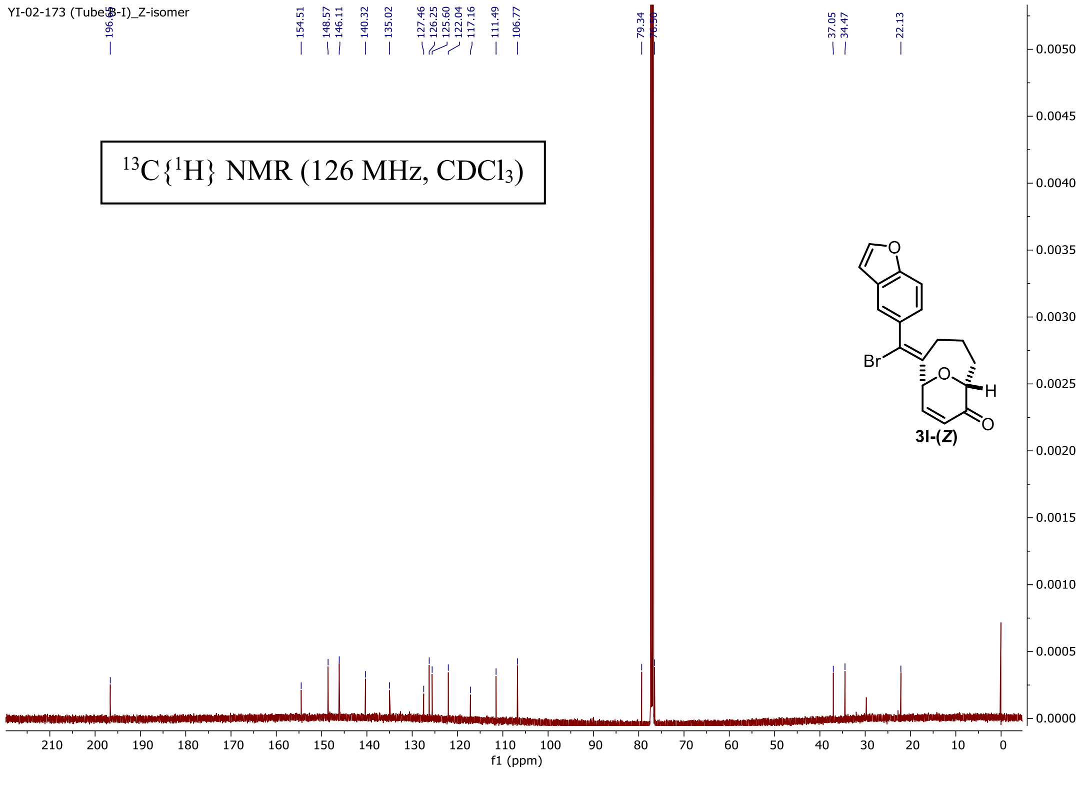
YI-02-173 (Tube B-I)_Z-isomer
154.51
148.57
146.11
140.32
135.02
127.46
126.25
125.60
122.04
117.16
111.49
106.77
79.34
37.05
34.47
22.13
^{13}C{^{1}H} NMR (126 MHz, CDCl$_3$)
Br
3l-(Z)
f1 (ppm)

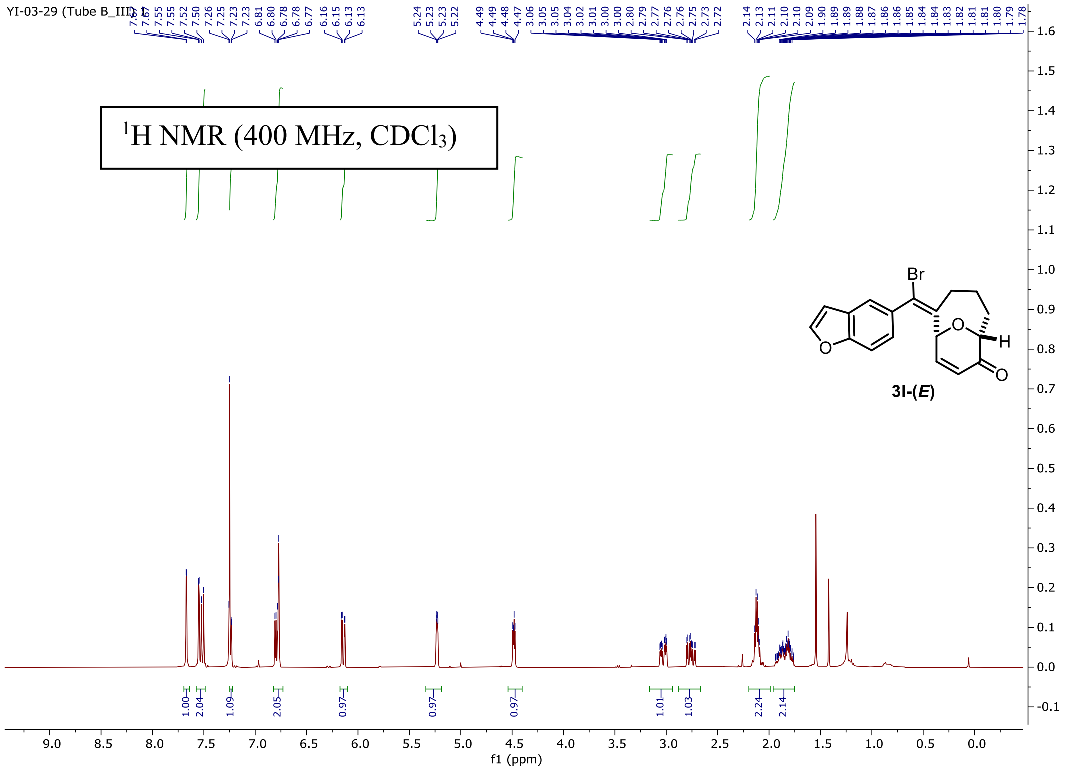

YI-03-29 (Tube B_III) 1
1H NMR (400 MHz, CDCl3)
Br
O
H
3l-(E)
f1 (ppm)

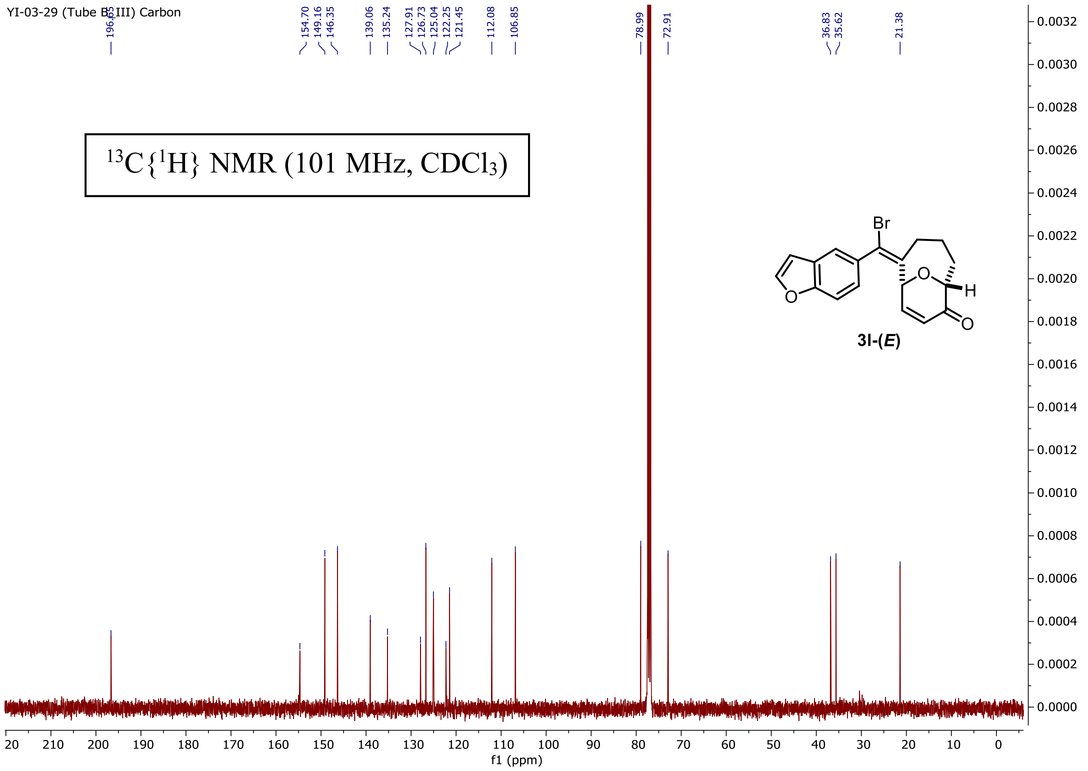

YI-03-29 (Tube B_III) Carbon
13C{1H} NMR (101 MHz, CDCl3)
Br
O
H
3l-(E)
f1 (ppm)

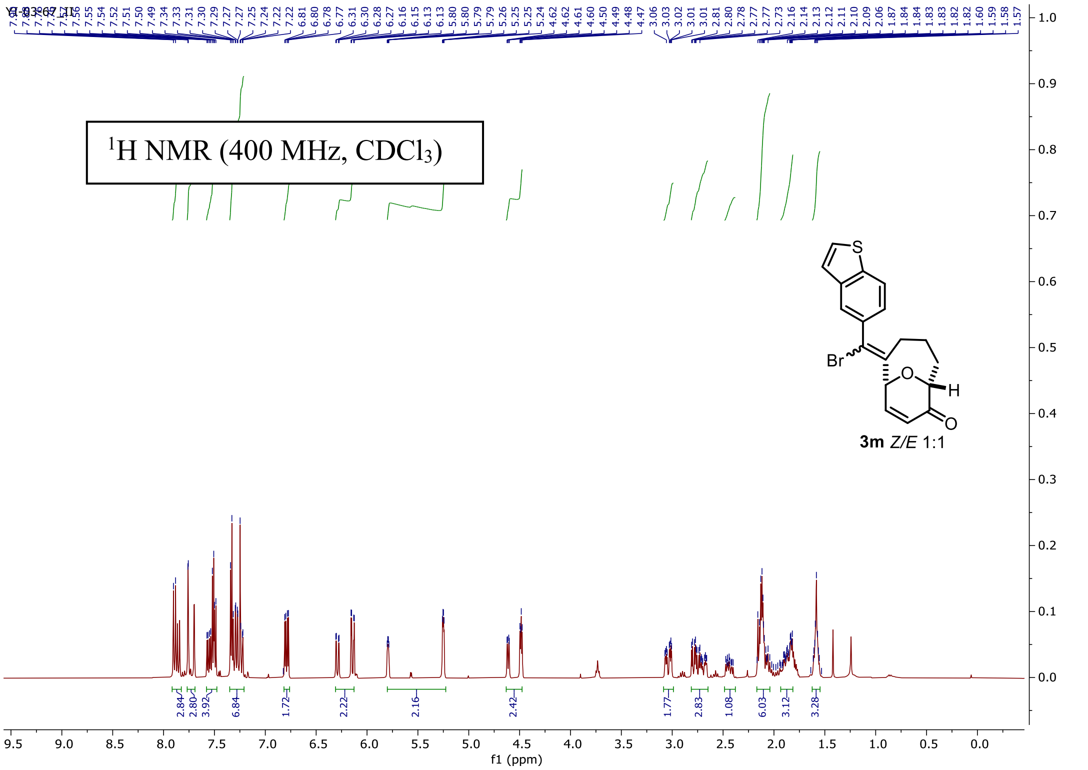

^{1}H NMR (400 MHz, CDCl$_3$)
Br
S
O
H
O
3m Z/E 1:1
f1 (ppm)

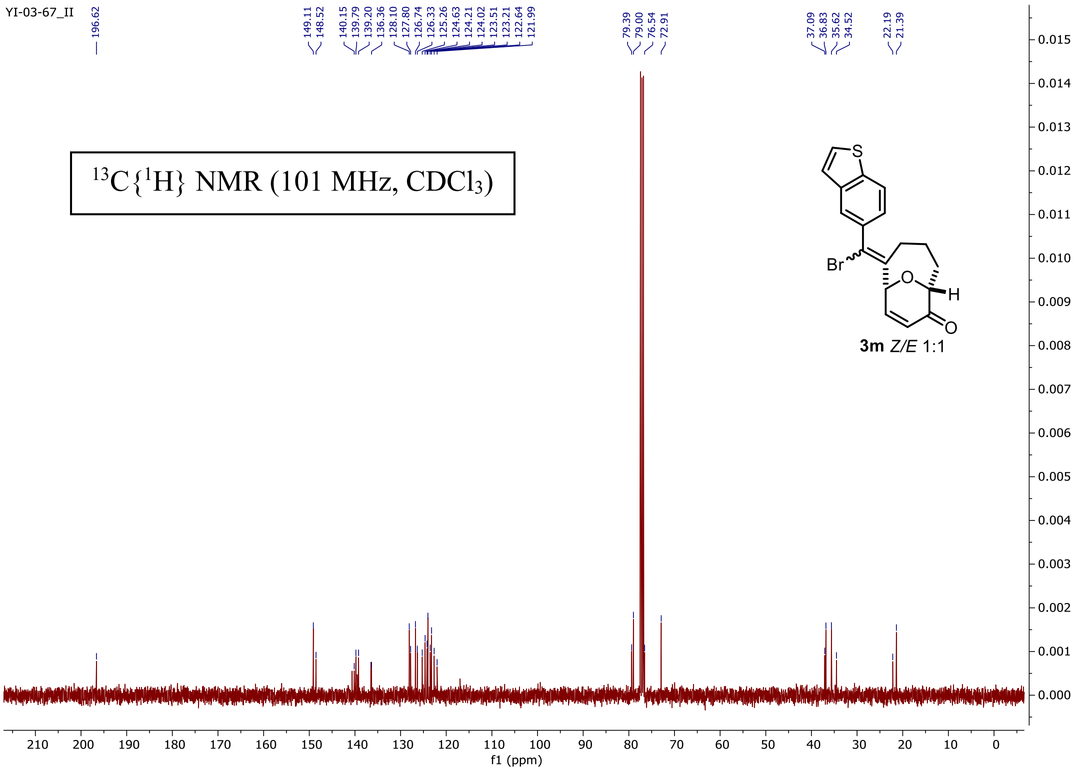

YI-03-67_II
196.62
149.11
148.52
140.15
139.79
139.20
136.36
128.10
127.80
126.74
126.33
125.26
124.63
124.21
124.02
123.51
123.21
122.64
121.99
79.39
79.00
76.54
72.91
37.09
36.83
35.62
34.52
22.19
21.39
^{13}C{^{1}H} NMR (101 MHz, CDCl$_3$)
Br
S
O
H
O
3m Z/E 1:1
f1 (ppm)

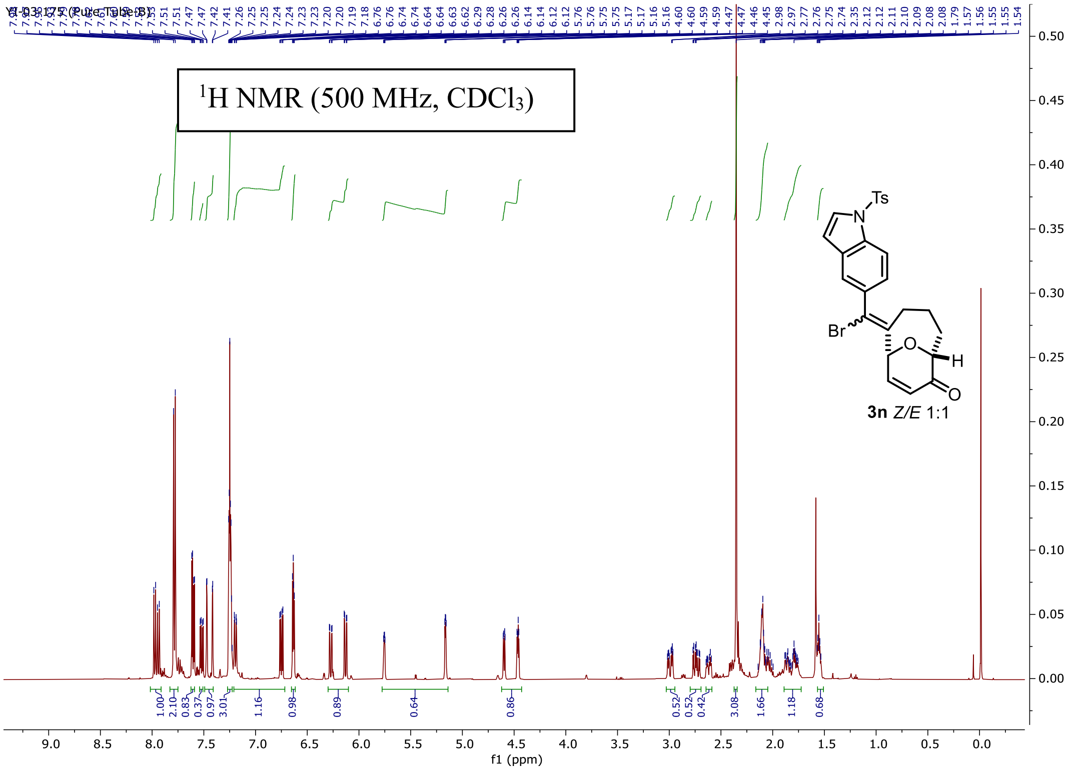

YI-03-175 (Pure Tube-B)
1H NMR (500 MHz, CDCl3)
Ts
N
Br
O
H
O
3n Z/E 1:1
f1 (ppm)

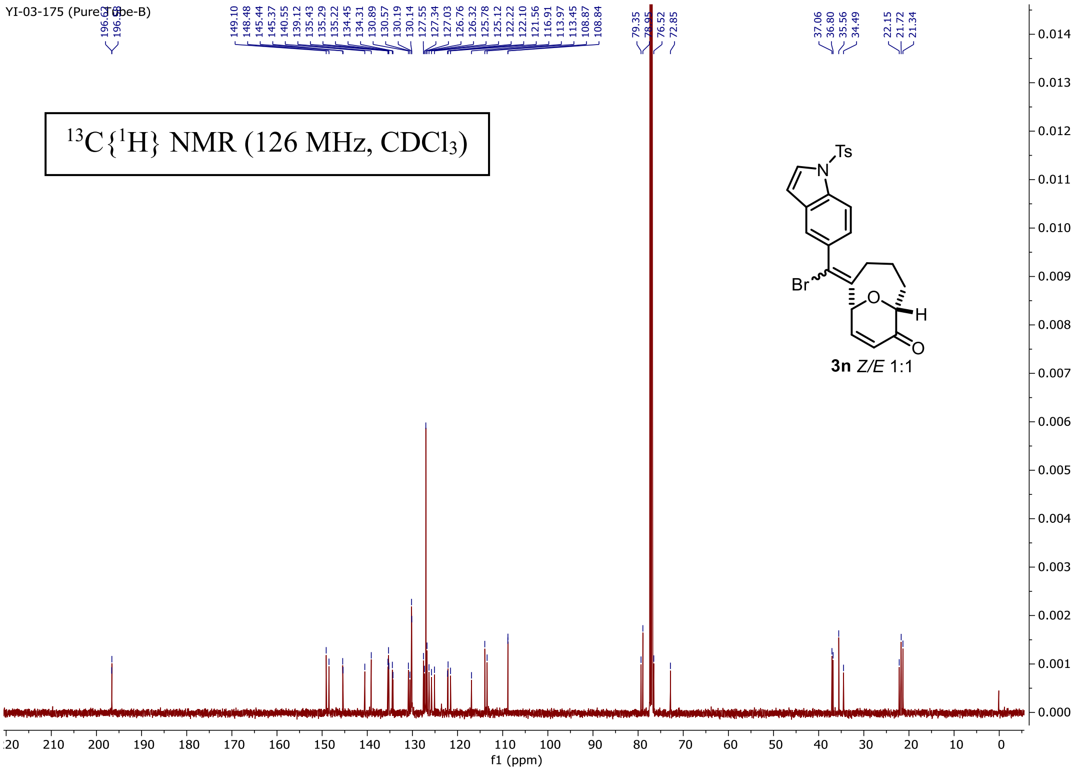

YI-03-175 (Pure Tube-B)
13C{1H} NMR (126 MHz, CDCl3)
Ts
N
Br
O
H
O
3n Z/E 1:1
f1 (ppm)

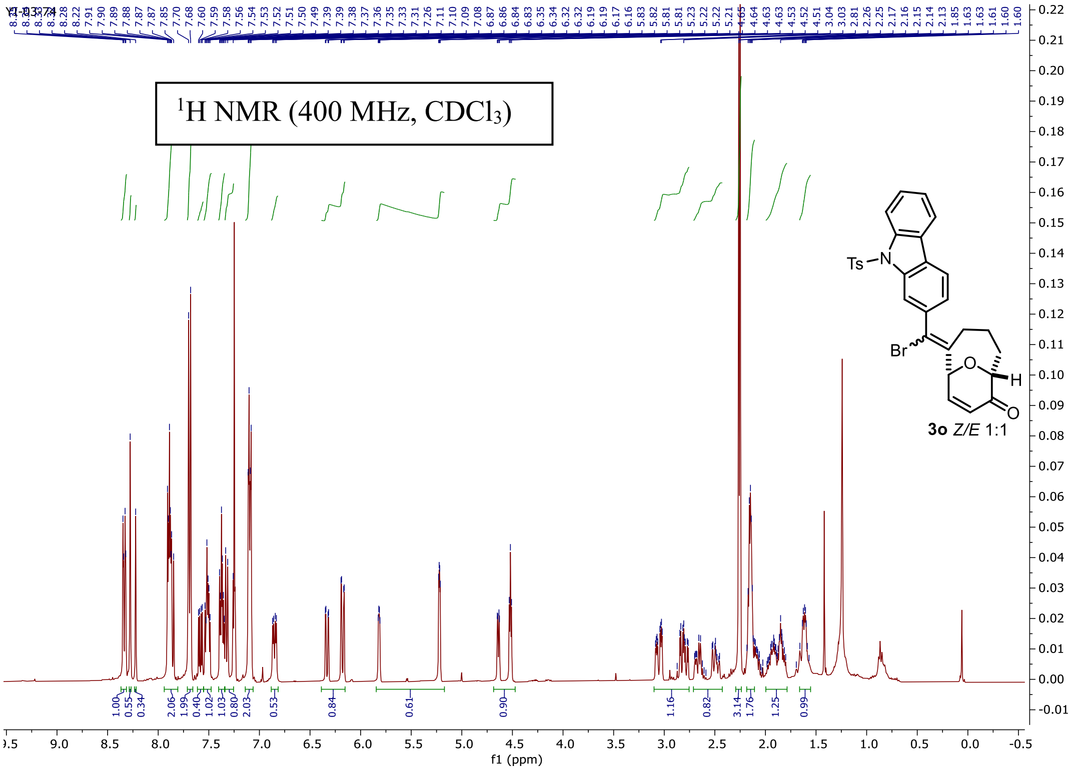

YI-03-74
^{1}H NMR (400 MHz, CDCl$_3$)
Ts
N
Br
O
H
O
3o Z/E 1:1
f1 (ppm)

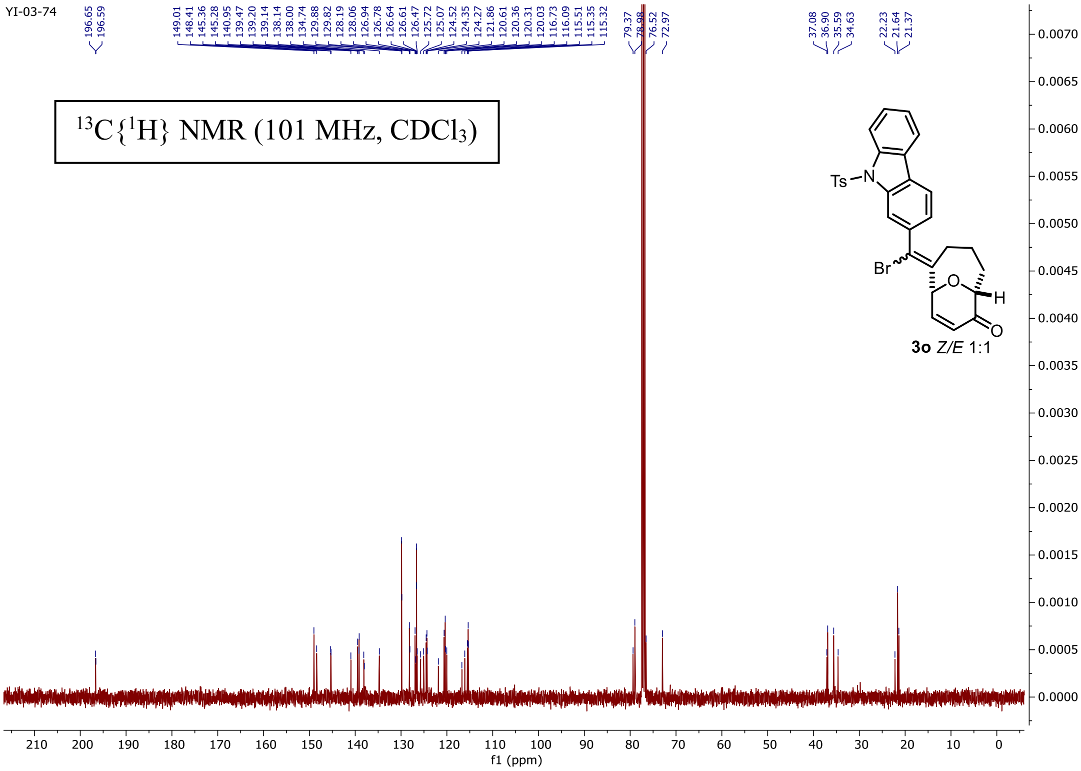

YI-03-74
^{13}C{^{1}H} NMR (101 MHz, CDCl$_3$)
Ts
N
Br
O
H
O
3o Z/E 1:1
f1 (ppm)

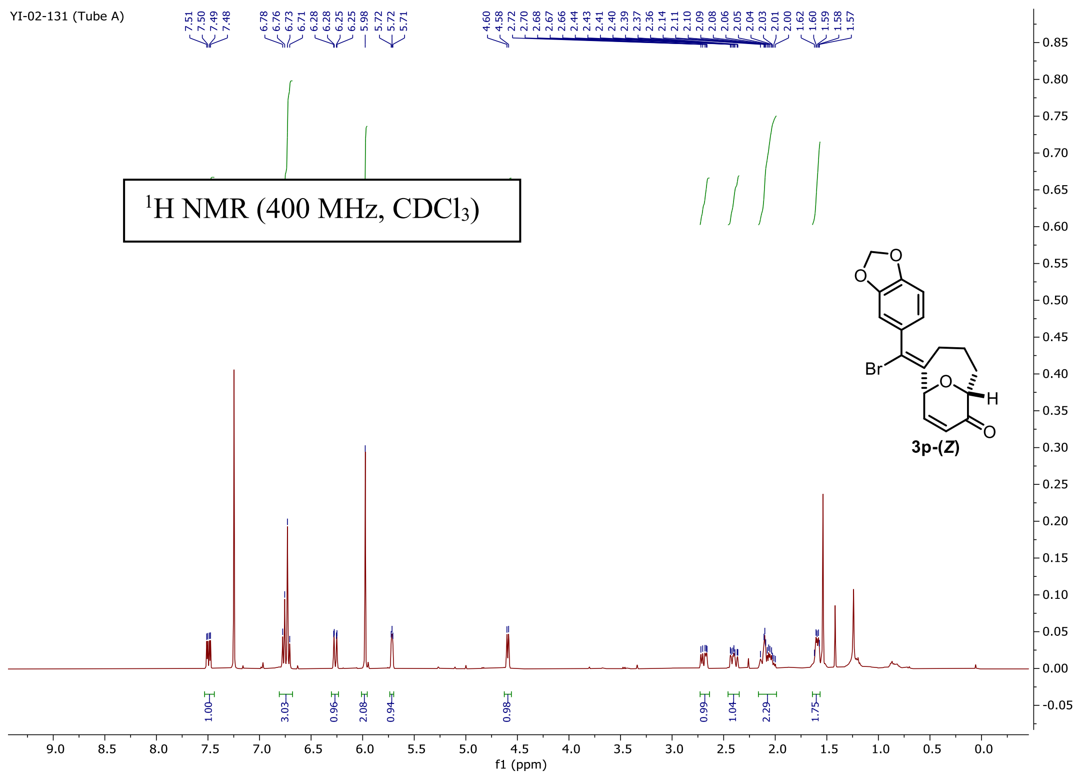
YI-02-131 (Tube A)
7.51 7.50 7.49 7.48 6.78 6.76 6.73 6.71 6.28 6.28 6.25 6.25 5.98 5.72 5.72 5.71 4.60 4.58 2.72 2.70 2.68 2.67 2.66 2.44 2.43 2.41 2.40 2.39 2.37 2.36 2.14 2.11 2.10 2.09 2.08 2.06 2.05 2.04 2.03 2.01 2.00 1.62 1.60 1.59 1.58 1.57
^{1}H NMR (400 MHz, $CDCl_3$)
Br
O
H
3p-(Z)
1.00 3.03 0.96 2.08 0.94 0.98 0.99 1.04 2.29 1.75
f1 (ppm)

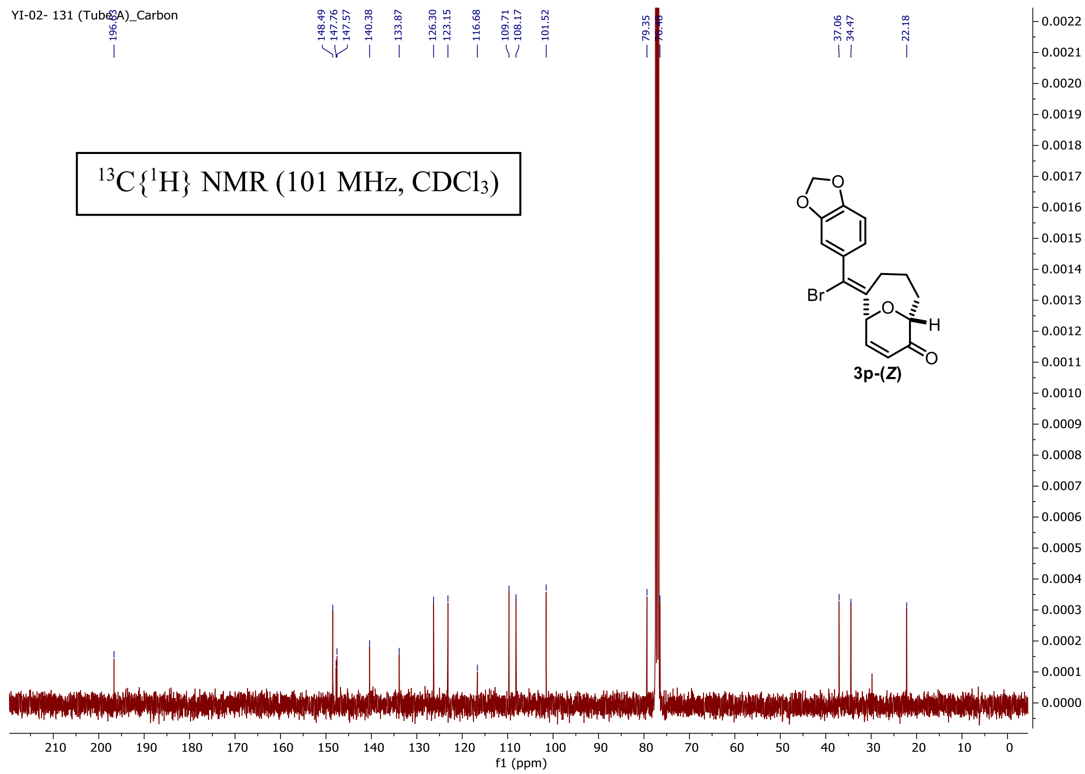
YI-02- 131 (Tube A)_Carbon
196.5 148.49 147.76 147.57 140.38 133.87 126.30 123.15 116.68 109.71 108.17 101.52 79.35 37.06 34.47 22.18
$^{13}C\{^{1}H\}$ NMR (101 MHz, $CDCl_3$)
Br
O
H
3p-(Z)
f1 (ppm)

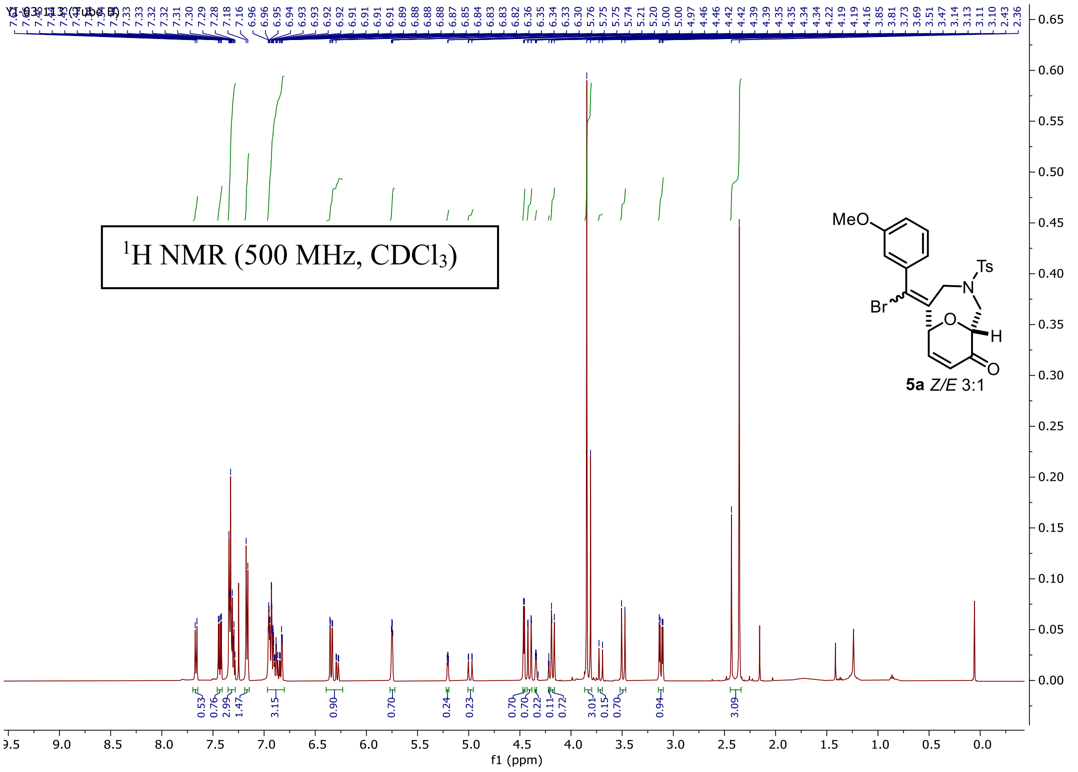
YI-03-113 (Tube B)
^{1}H NMR (500 MHz, CDCl$_3$)
MeO
Ts
N
Br
O
H
O
5a Z/E 3:1
f1 (ppm)

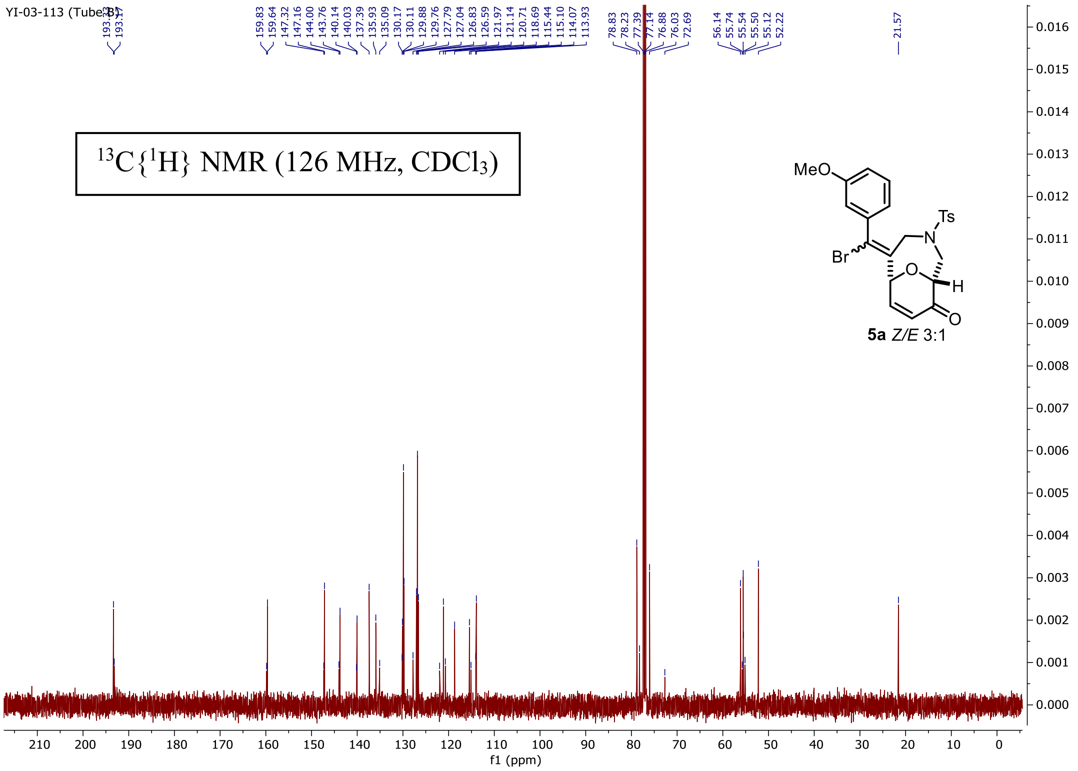
YI-03-113 (Tube B)
^{13}C{^{1}H} NMR (126 MHz, CDCl$_3$)
MeO
Ts
N
Br
O
H
O
5a Z/E 3:1
f1 (ppm)

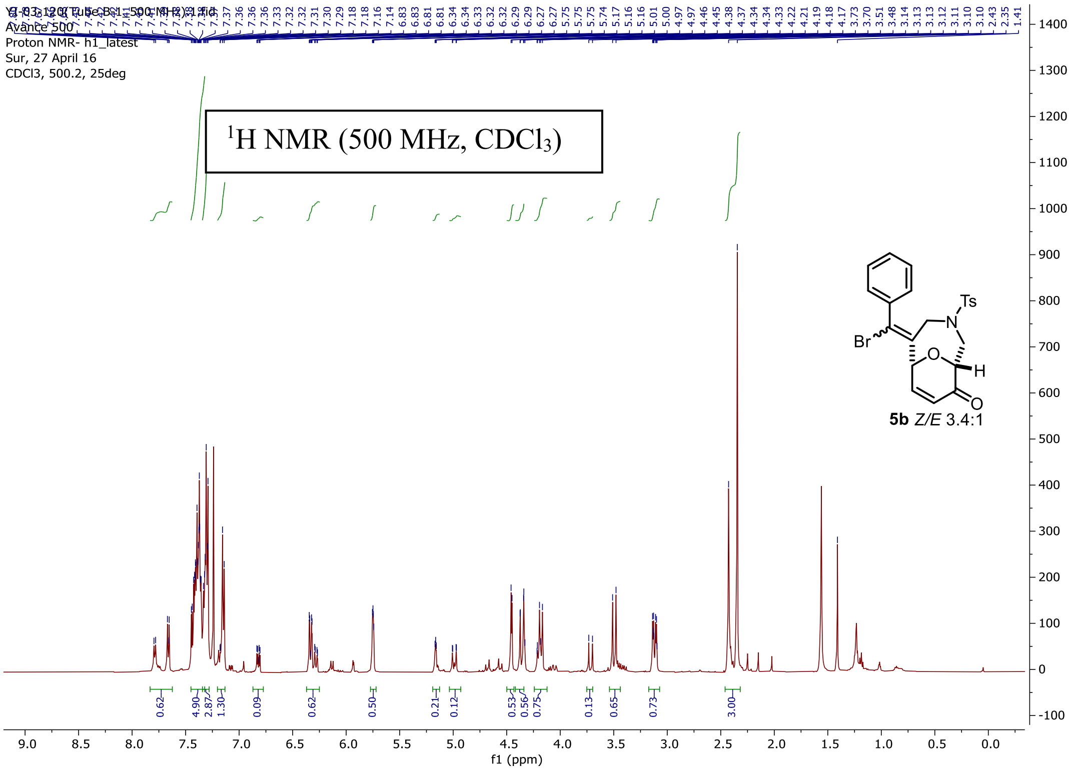

YI-03-120
Avance 500
Proton NMR- h1_latest
Sur, 27 April 16
CDCl3, 500.2, 25deg
^{1}H NMR (500 MHz, $CDCl_3$)
Br
N
Ts
O
H
O
5b Z/E 3.4:1
f1 (ppm)

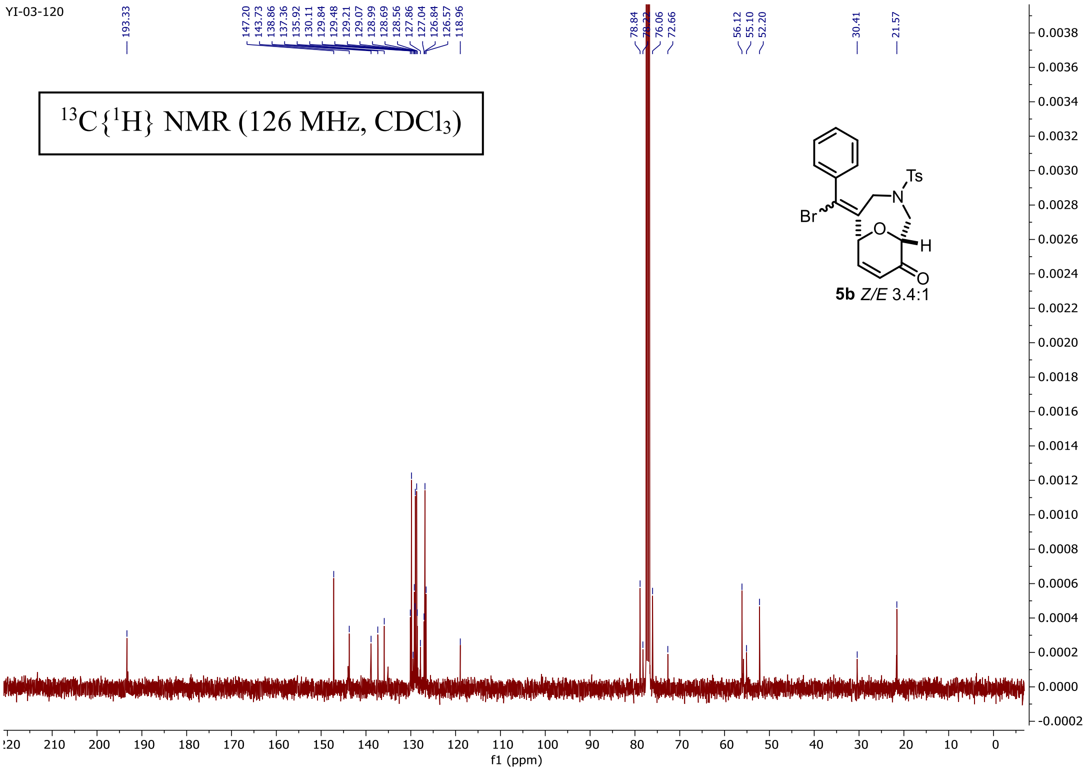

YI-03-120
193.33
147.20
143.73
138.86
137.36
135.92
130.11
129.84
129.48
129.21
129.07
128.99
128.69
128.56
127.86
127.04
126.84
126.57
118.96
78.84
76.06
72.66
56.12
55.10
52.20
30.41
21.57
^{13}C{^{1}H} NMR (126 MHz, $CDCl_3$)
Br
N
Ts
O
H
O
5b Z/E 3.4:1
f1 (ppm)

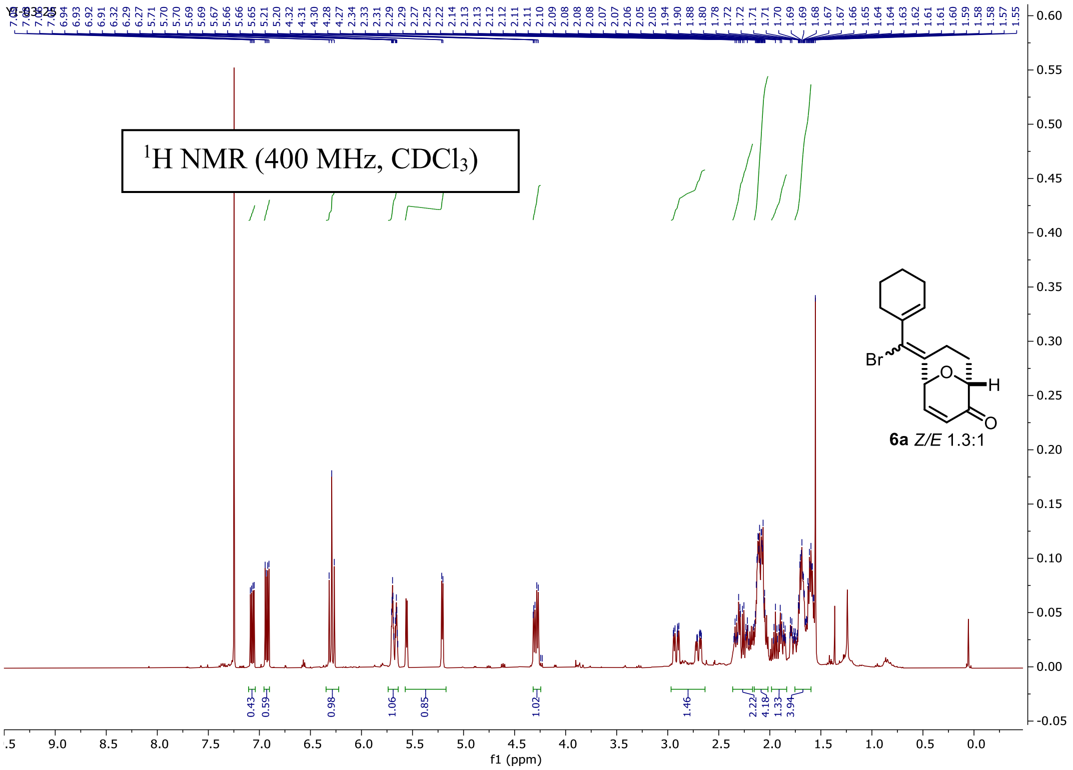
YI-03-25
1H NMR (400 MHz, CDCl3)
Br
O
H
O
6a Z/E 1.3:1
f1 (ppm)

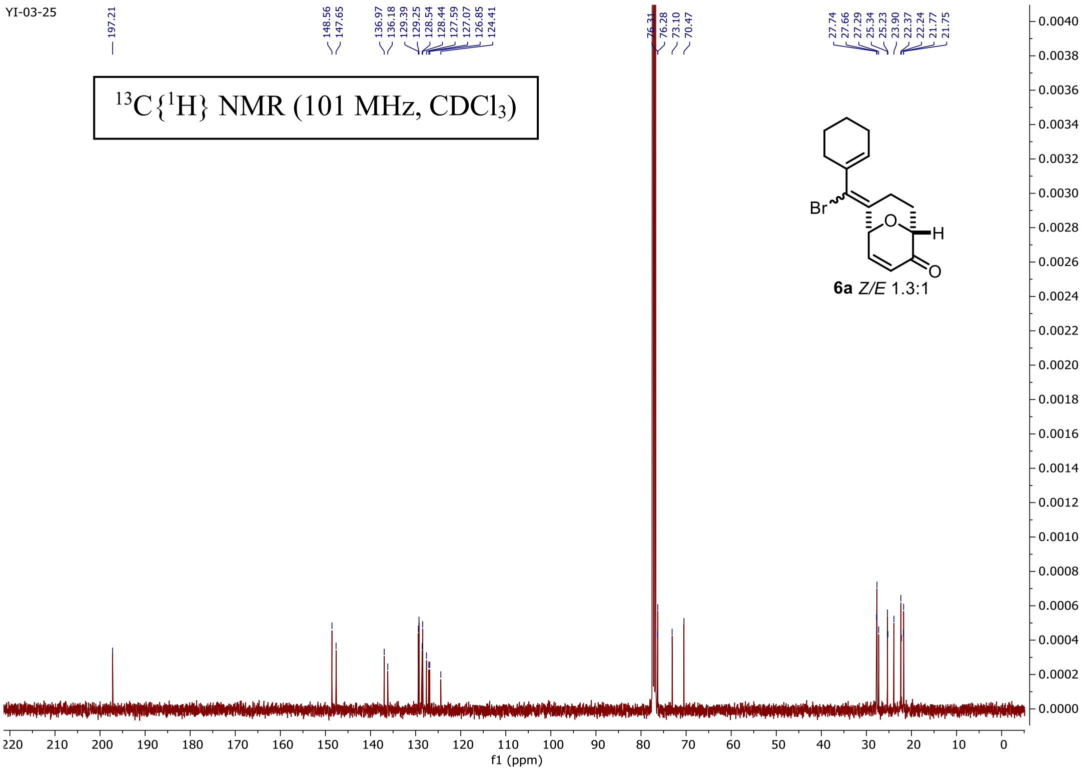
YI-03-25
13C{1H} NMR (101 MHz, CDCl3)
Br
O
H
O
6a Z/E 1.3:1
f1 (ppm)

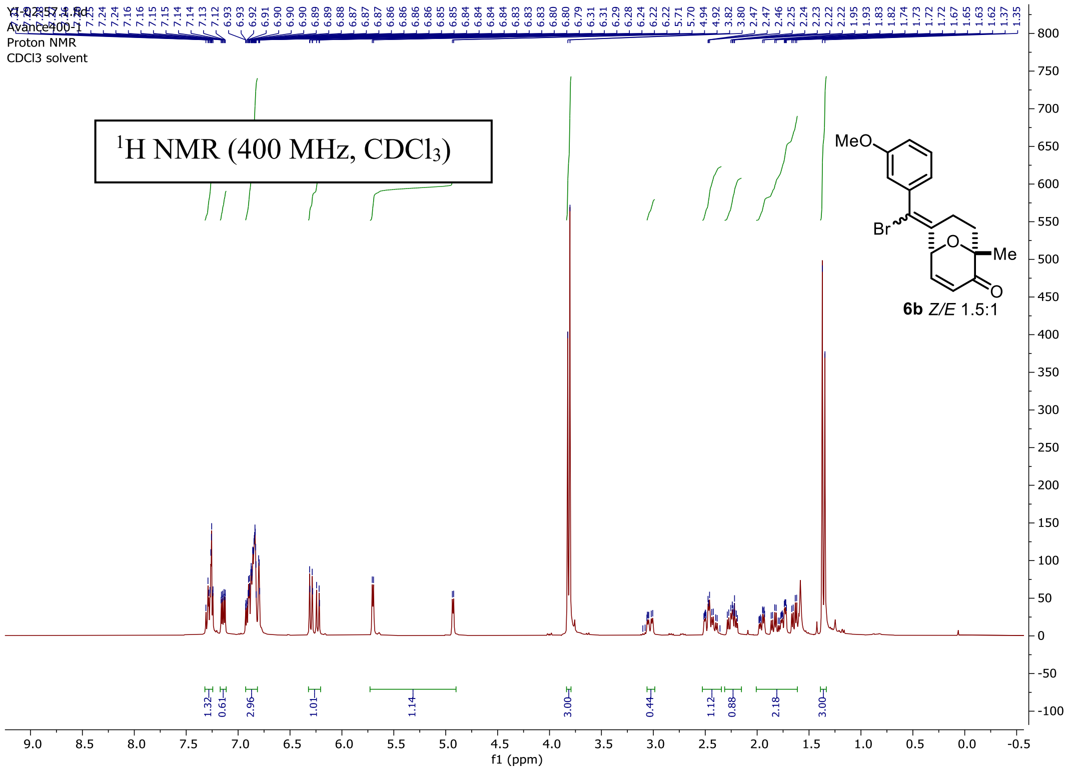

1H NMR (400 MHz, CDCl3)
MeO
Br
O
Me
O
6b Z/E 1.5:1
f1 (ppm)

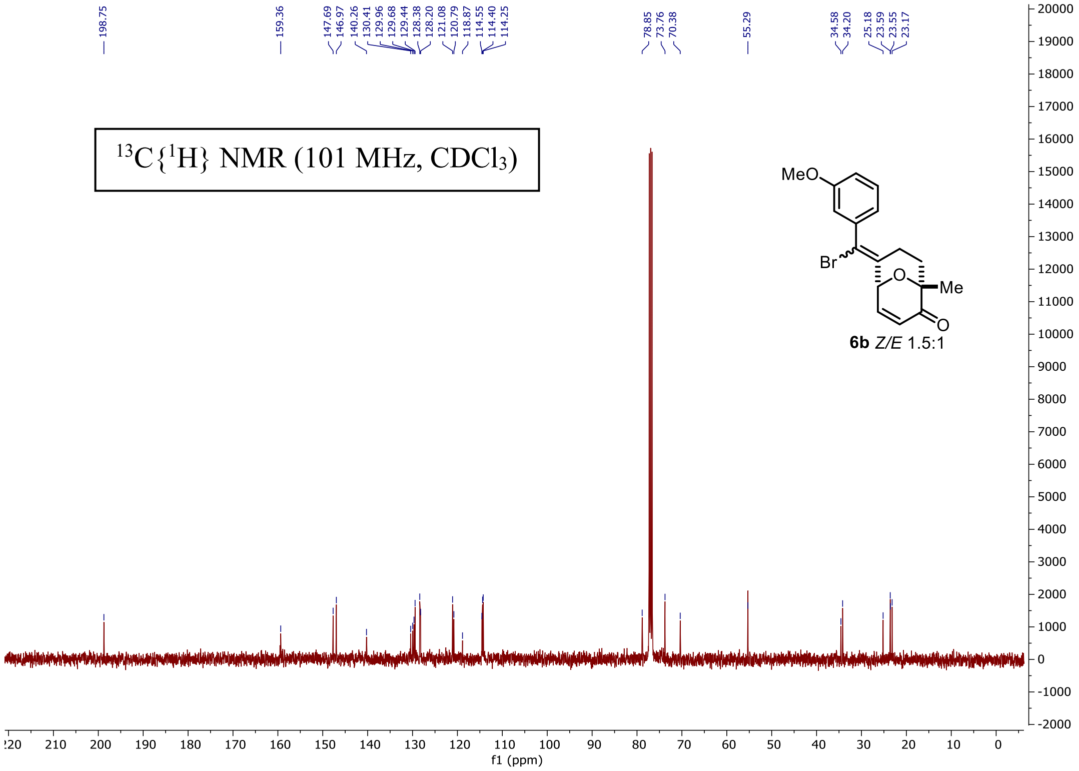

13C{1H} NMR (101 MHz, CDCl3)
MeO
Br
O
Me
O
6b Z/E 1.5:1
f1 (ppm)

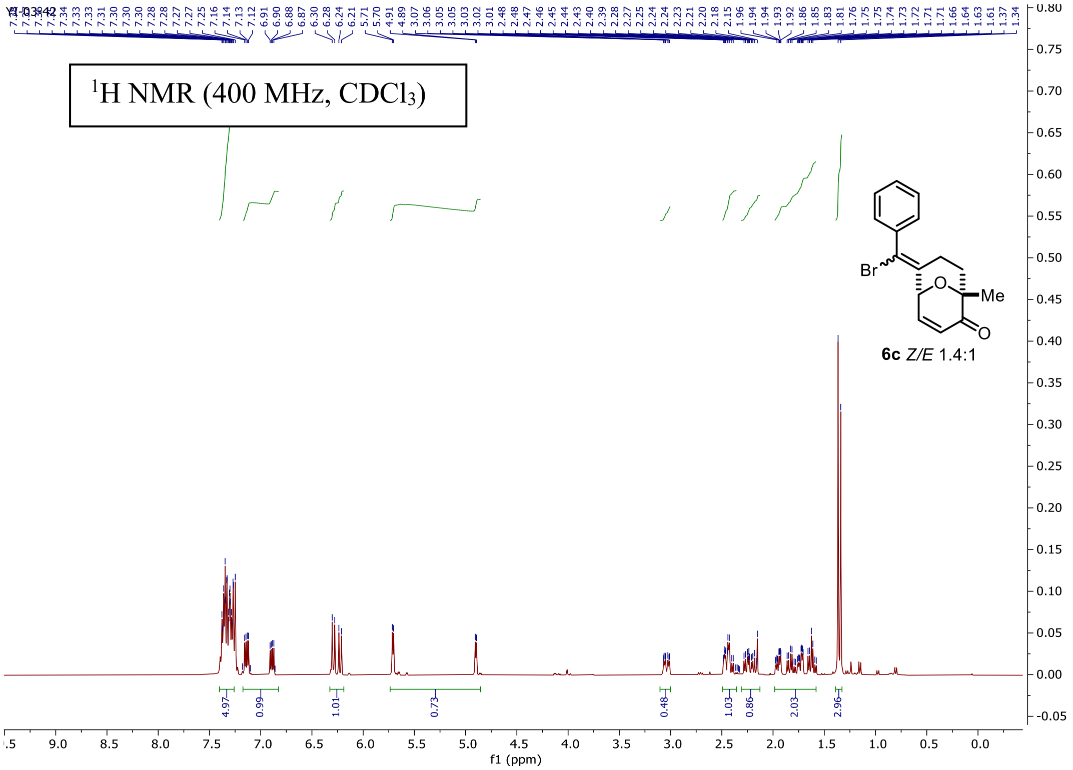
YI-03-42
1H NMR (400 MHz, CDCl3)
Br
O
Me
O
6c Z/E 1.4:1
f1 (ppm)

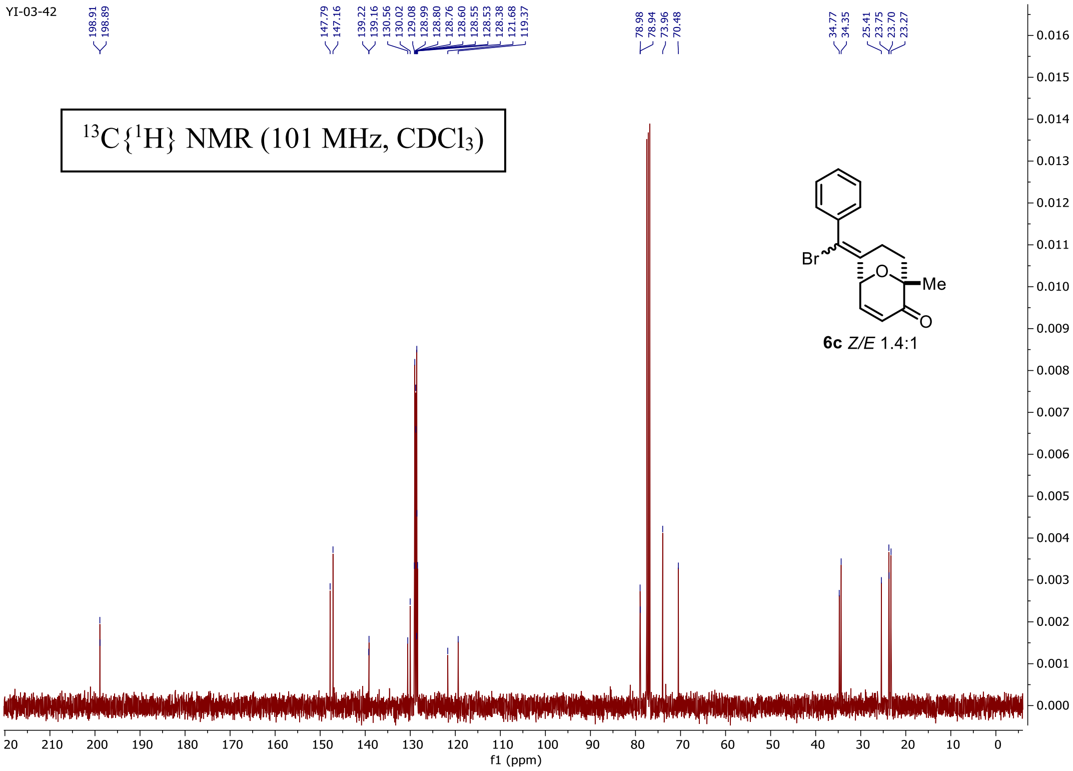
YI-03-42
13C{1H} NMR (101 MHz, CDCl3)
Br
O
Me
O
6c Z/E 1.4:1
f1 (ppm)

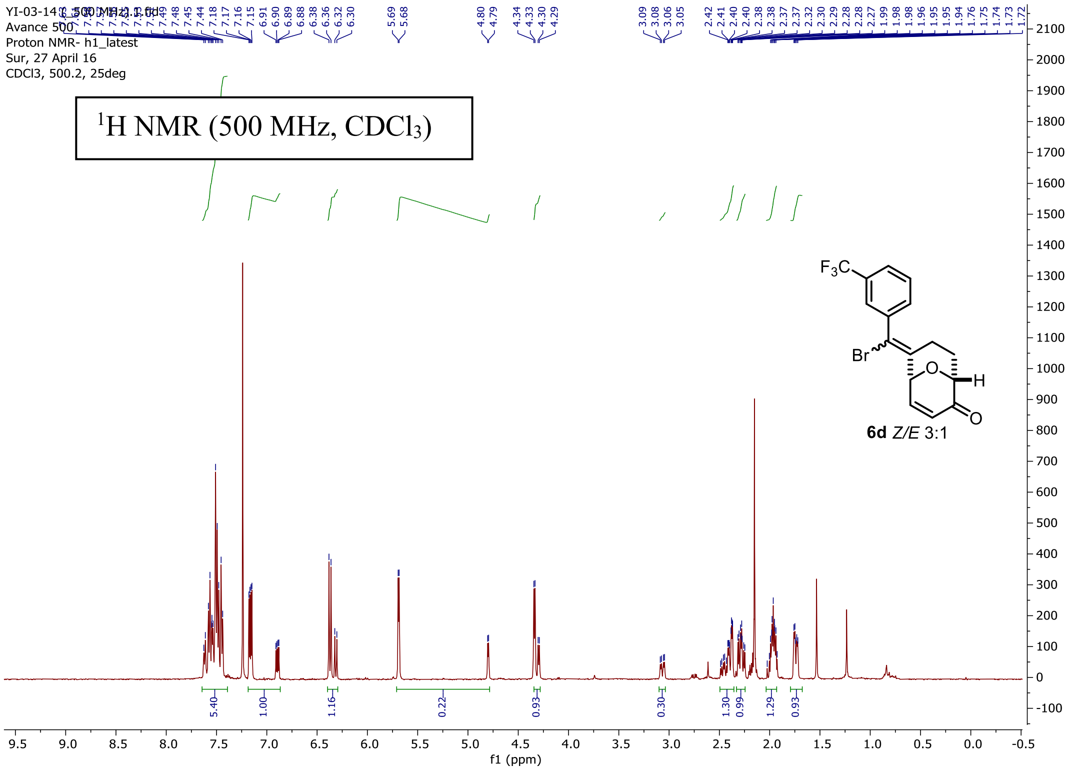
YI-03-147_500MHz-1.1.fid
Avance 500
Proton NMR- h1_latest
Sur, 27 April 16
CDCl3, 500.2, 25deg
¹H NMR (500 MHz, CDCl₃)
F3C
Br
O
H
O
6d Z/E 3:1
f1 (ppm)

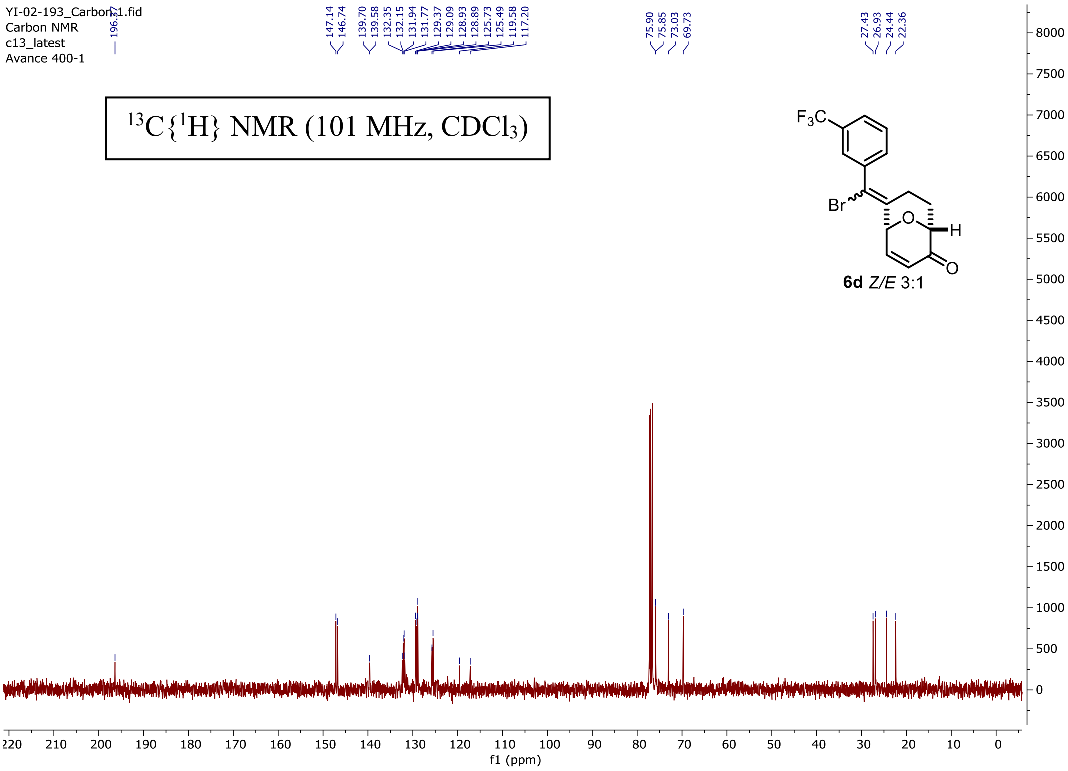
YI-02-193_Carbon-1.fid
Carbon NMR
c13_latest
Avance 400-1
196.77
147.14
146.74
139.70
139.58
132.35
132.15
131.94
131.77
129.37
129.09
128.93
128.89
125.73
125.49
119.58
117.20
75.90
75.85
73.03
69.73
27.43
26.93
24.44
22.36
¹³C{¹H} NMR (101 MHz, CDCl₃)
F3C
Br
O
H
O
6d Z/E 3:1
f1 (ppm)

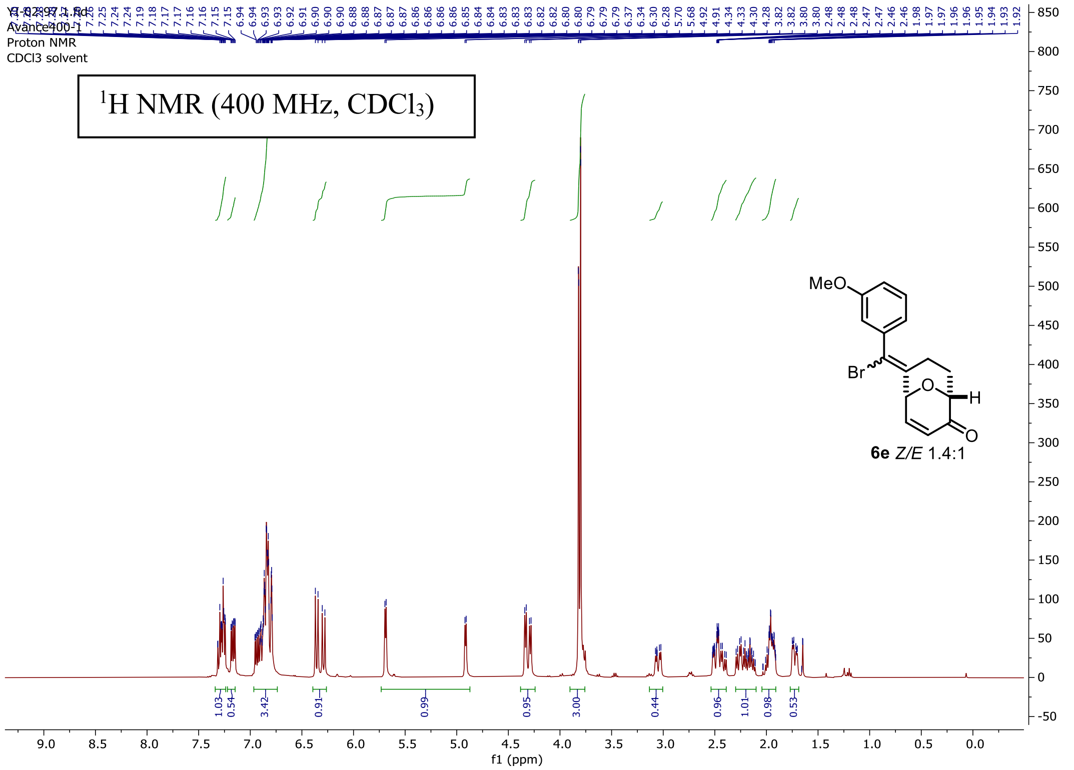
Proton NMR
CDCl3 solvent
1H NMR (400 MHz, CDCl3)
MeO
Br
O
H
O
6e Z/E 1.4:1
f1 (ppm)

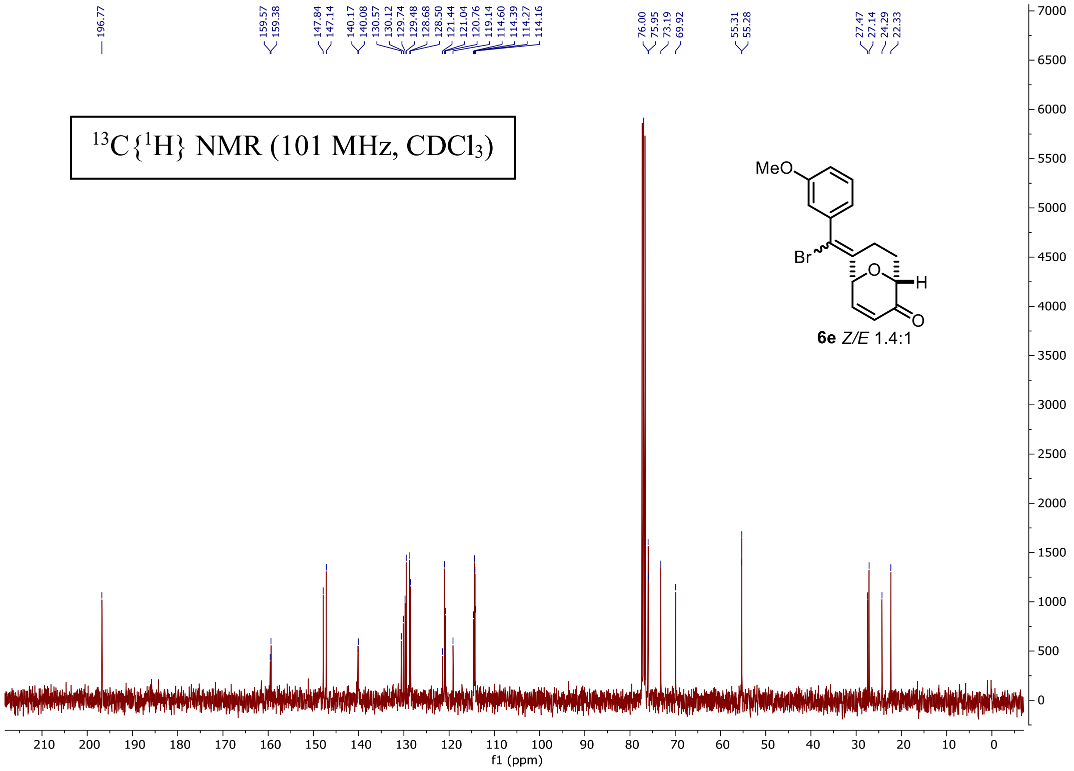
196.77
159.57
159.38
147.84
147.14
140.17
140.08
130.57
130.12
129.74
129.48
128.68
128.50
121.44
121.04
120.76
119.14
114.60
114.39
114.27
114.16
76.00
75.95
73.19
69.92
55.31
55.28
27.47
27.14
24.29
22.33
13C{1H} NMR (101 MHz, CDCl3)
MeO
Br
O
H
O
6e Z/E 1.4:1
f1 (ppm)

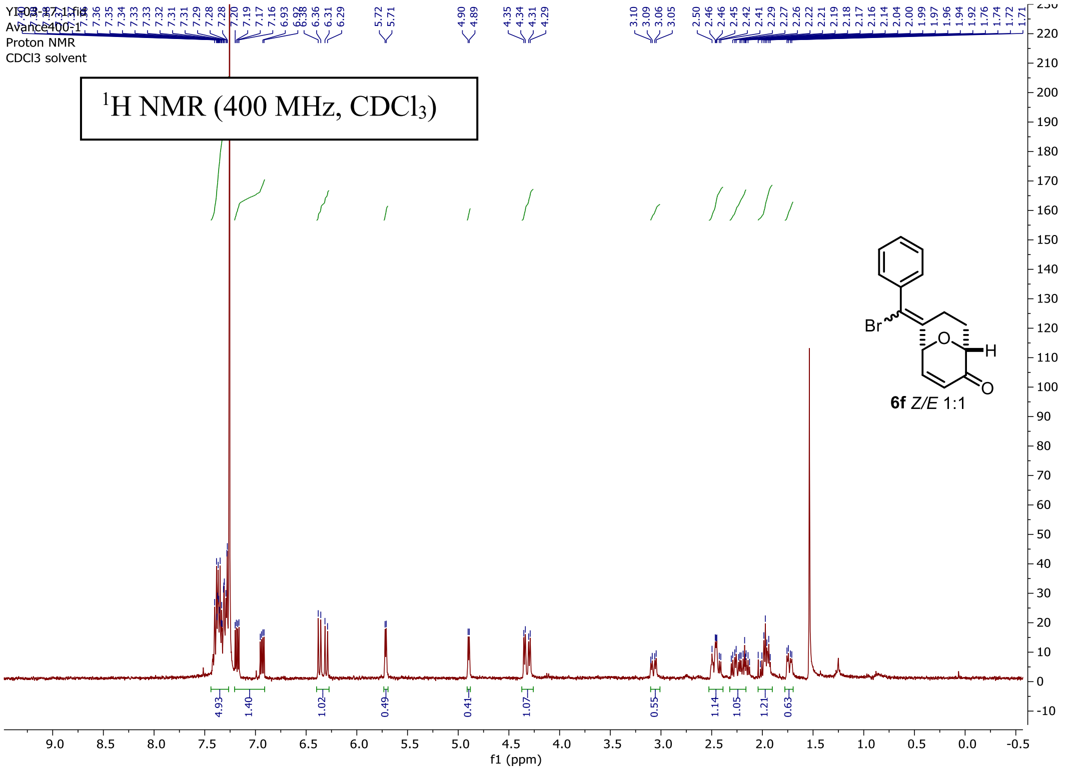

^{1}H NMR (400 MHz, $CDCl_3$)

6f *Z/E* 1:1

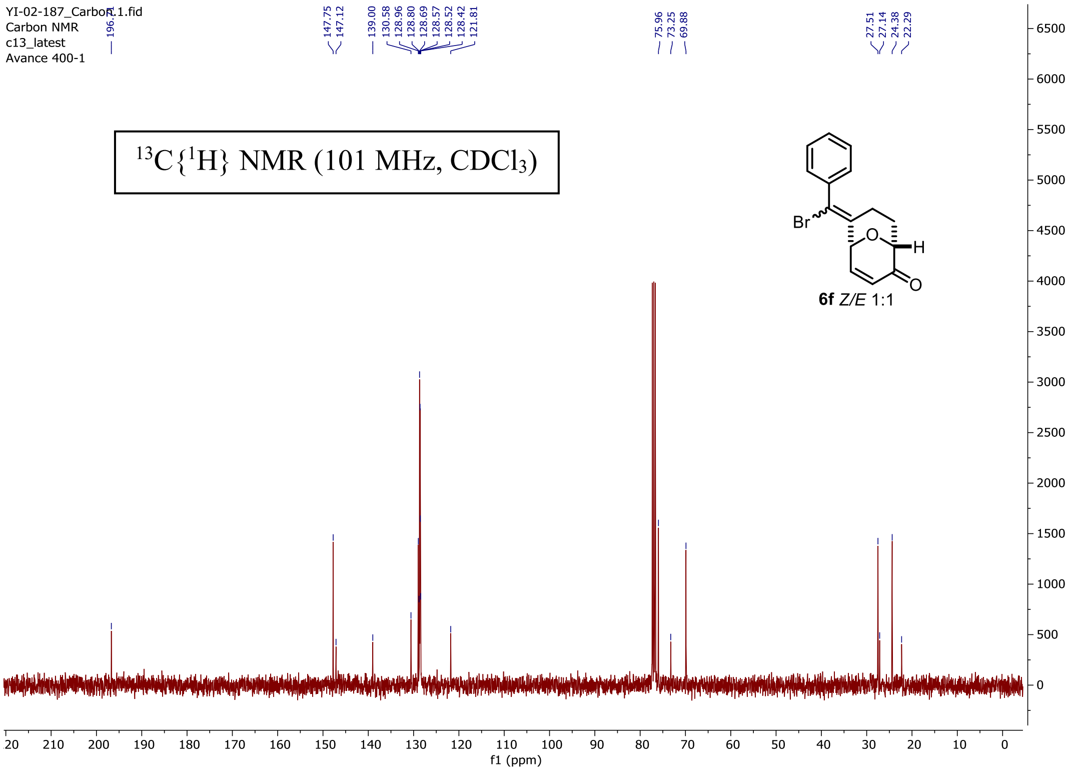

$^{13}C\{^{1}H\}$ NMR (101 MHz, $CDCl_3$)

6f *Z/E* 1:1

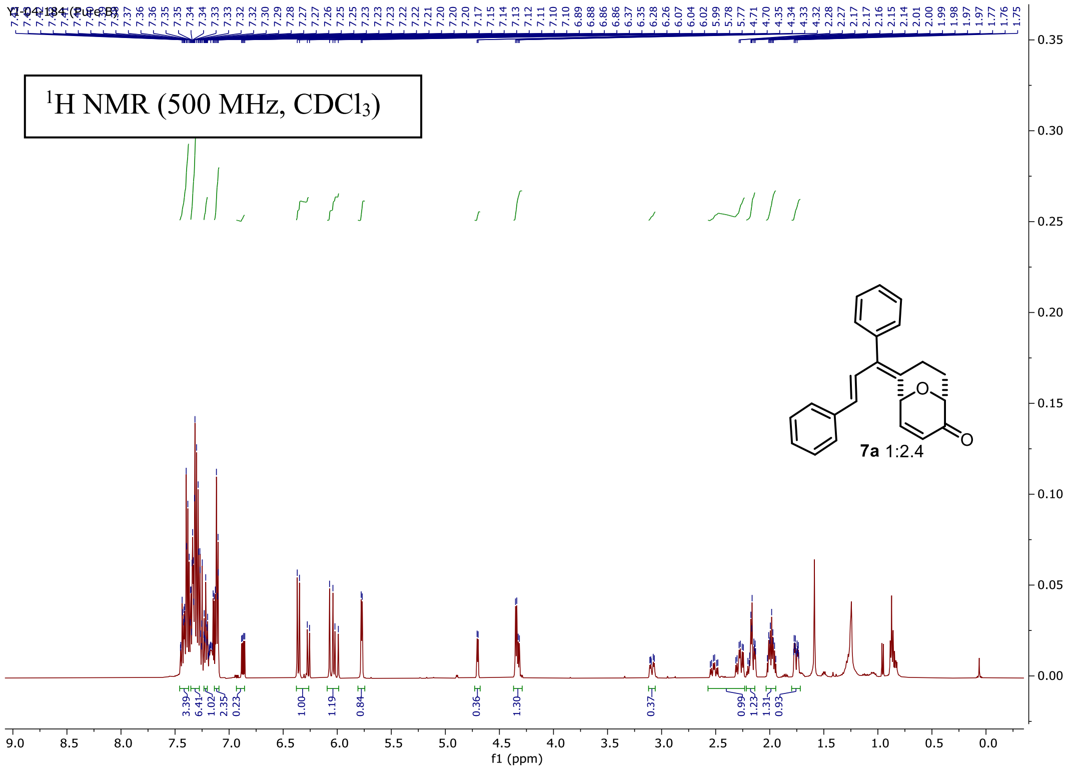

¹H NMR (500 MHz, CDCl₃)
7a 1:2.4
f1 (ppm)

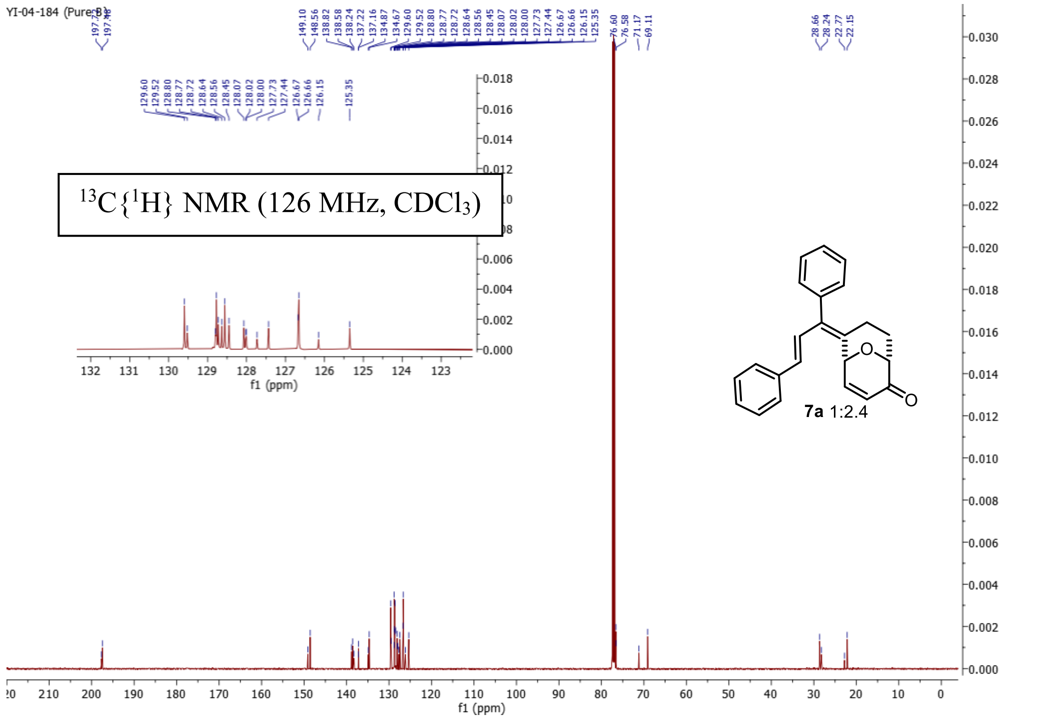

YI-04-184 (Pure B)
¹³C{¹H} NMR (126 MHz, CDCl₃)
7a 1:2.4
f1 (ppm)

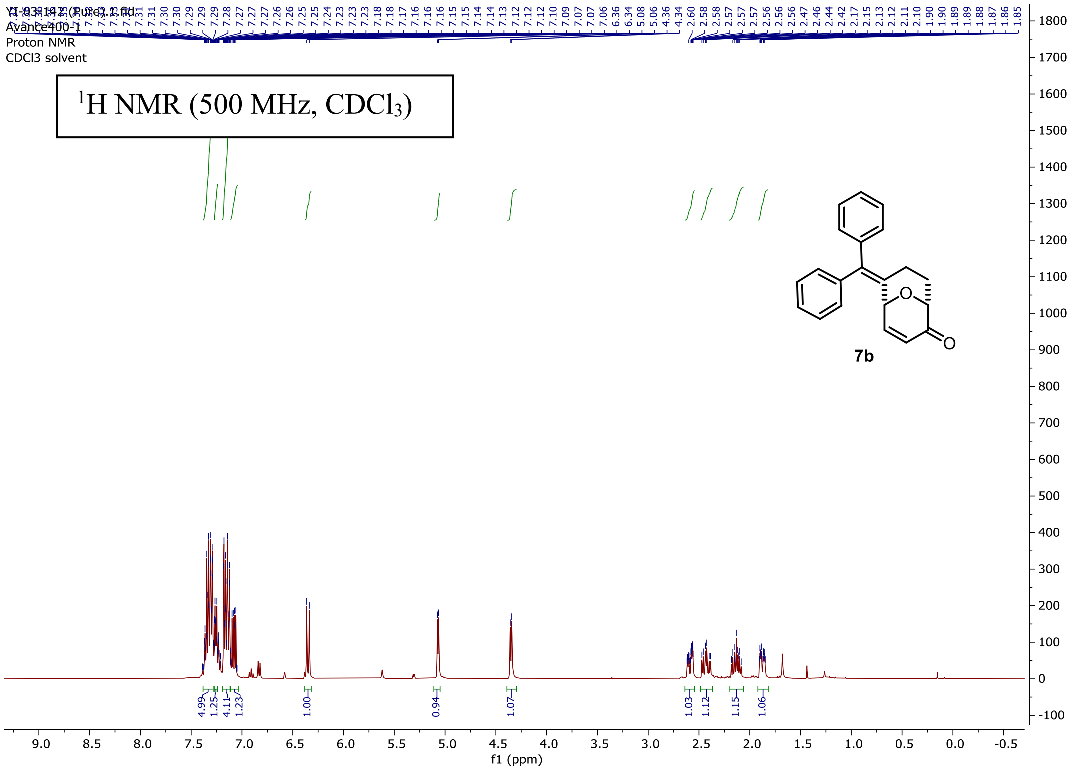
Avance400-1
Proton NMR
CDCl3 solvent
¹H NMR (500 MHz, CDCl₃)
7b
f1 (ppm)

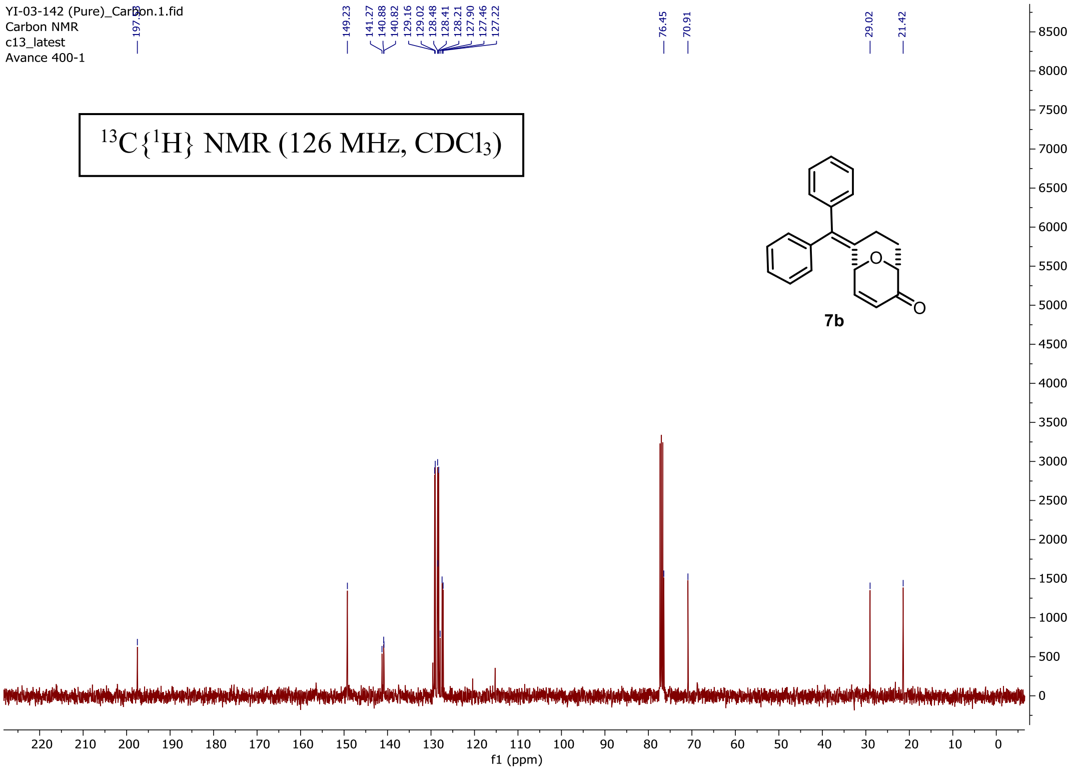
YI-03-142 (Pure)_Carbon.1.fid
Carbon NMR
c13_latest
Avance 400-1
149.23
141.27
140.88
140.82
129.16
129.02
128.48
128.41
128.21
127.90
127.46
127.22
76.45
70.91
29.02
21.42
¹³C{¹H} NMR (126 MHz, CDCl₃)
7b
f1 (ppm)

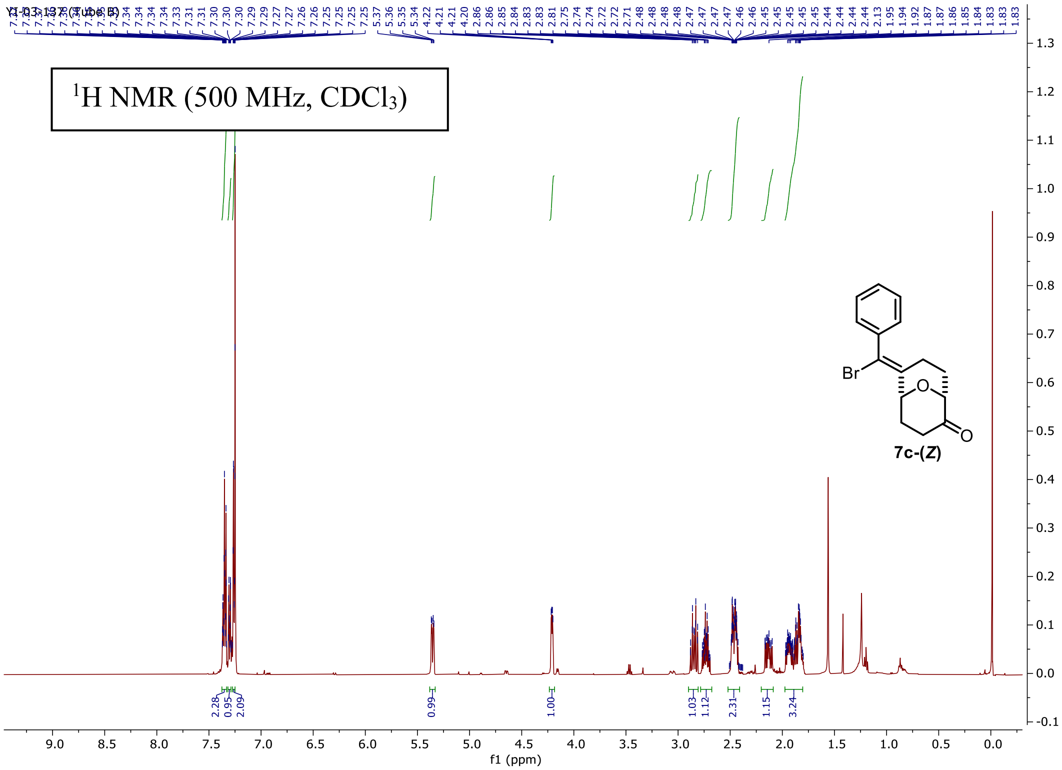
YI-03-137 (Tube B)
1H NMR (500 MHz, CDCl3)
Br
O
7c-(Z)
f1 (ppm)

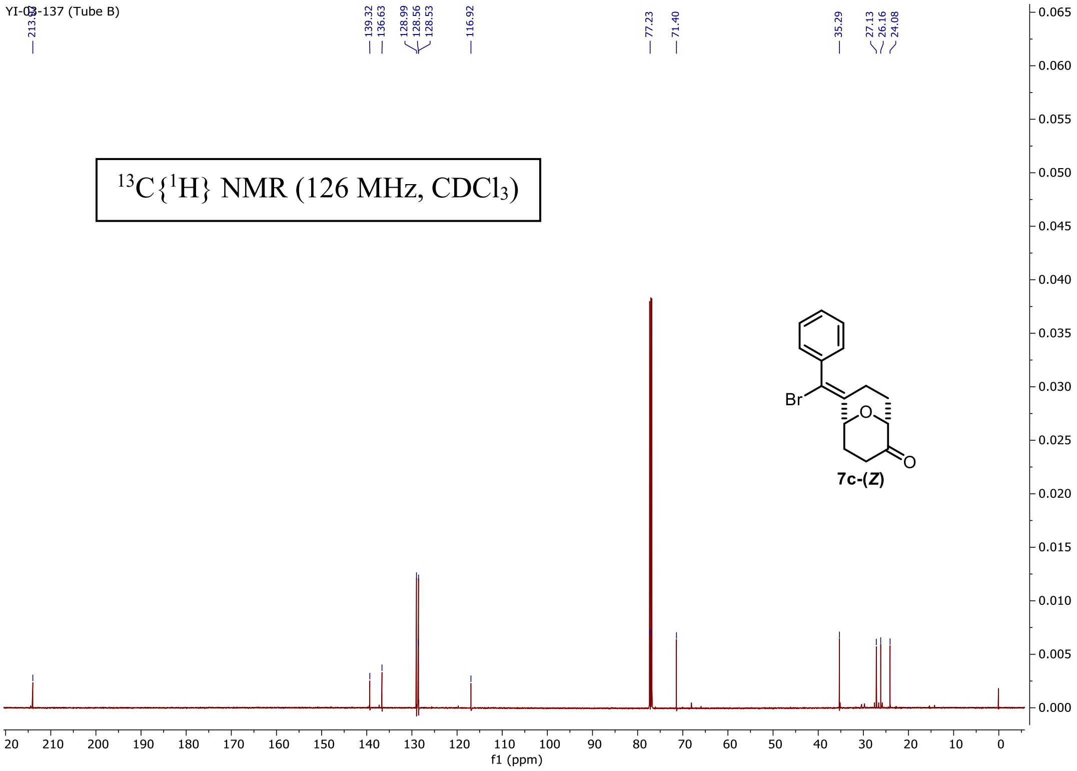
YI-03-137 (Tube B)
139.32
136.63
128.99
128.56
128.53
116.92
77.23
71.40
35.29
27.13
26.16
24.08
13C{1H} NMR (126 MHz, CDCl3)
Br
O
7c-(Z)
f1 (ppm)

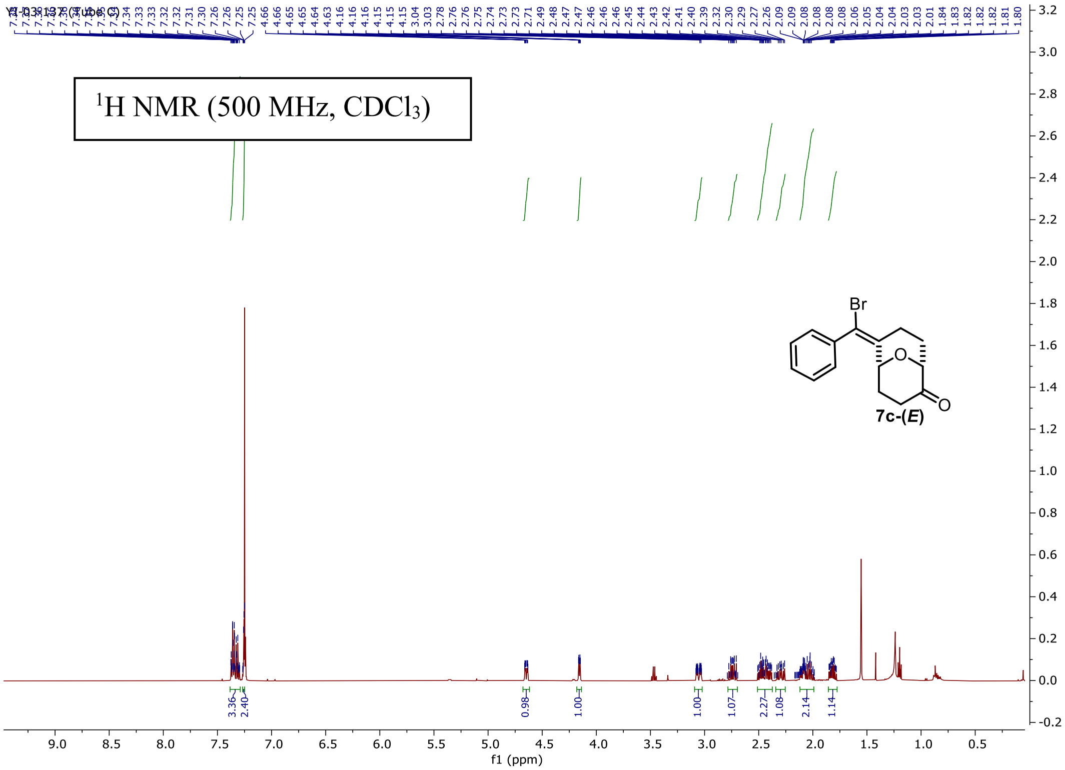
YI-03-137 (Tube C)
1H NMR (500 MHz, CDCl3)
Br
O
O
7c-(E)
f1 (ppm)

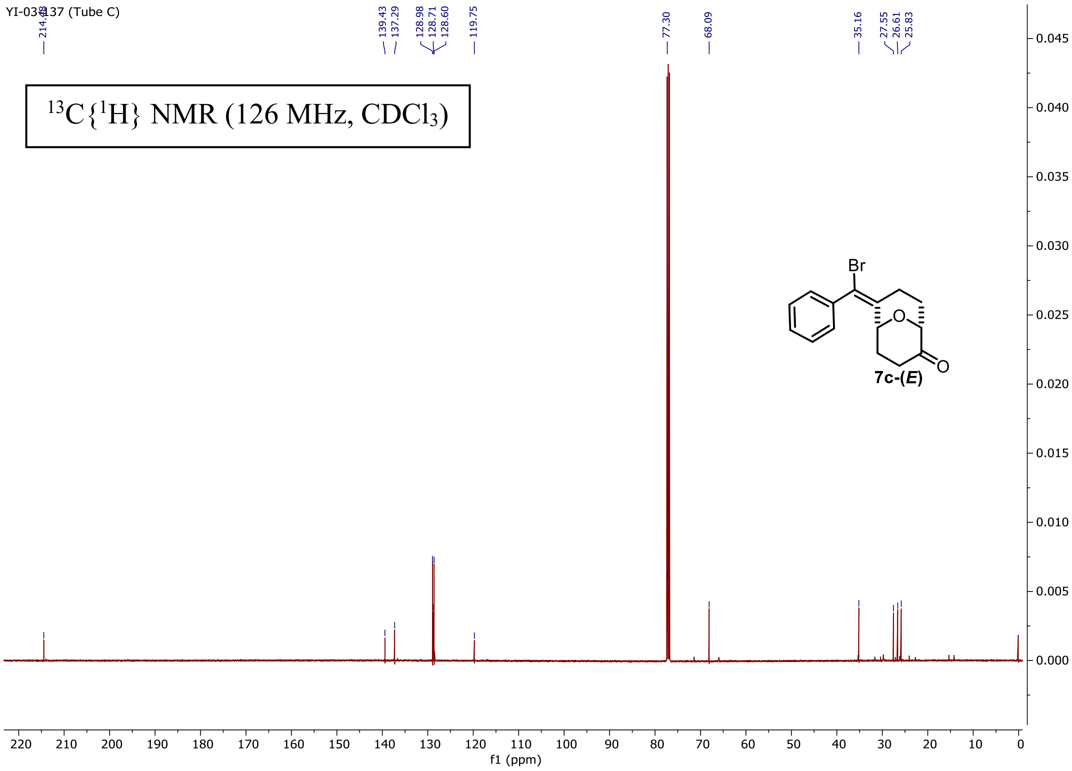
YI-03-137 (Tube C)
214.49
139.43
137.29
128.98
128.71
128.60
119.75
77.30
68.09
35.16
27.55
26.61
25.83
13C{1H} NMR (126 MHz, CDCl3)
Br
O
O
7c-(E)
f1 (ppm)

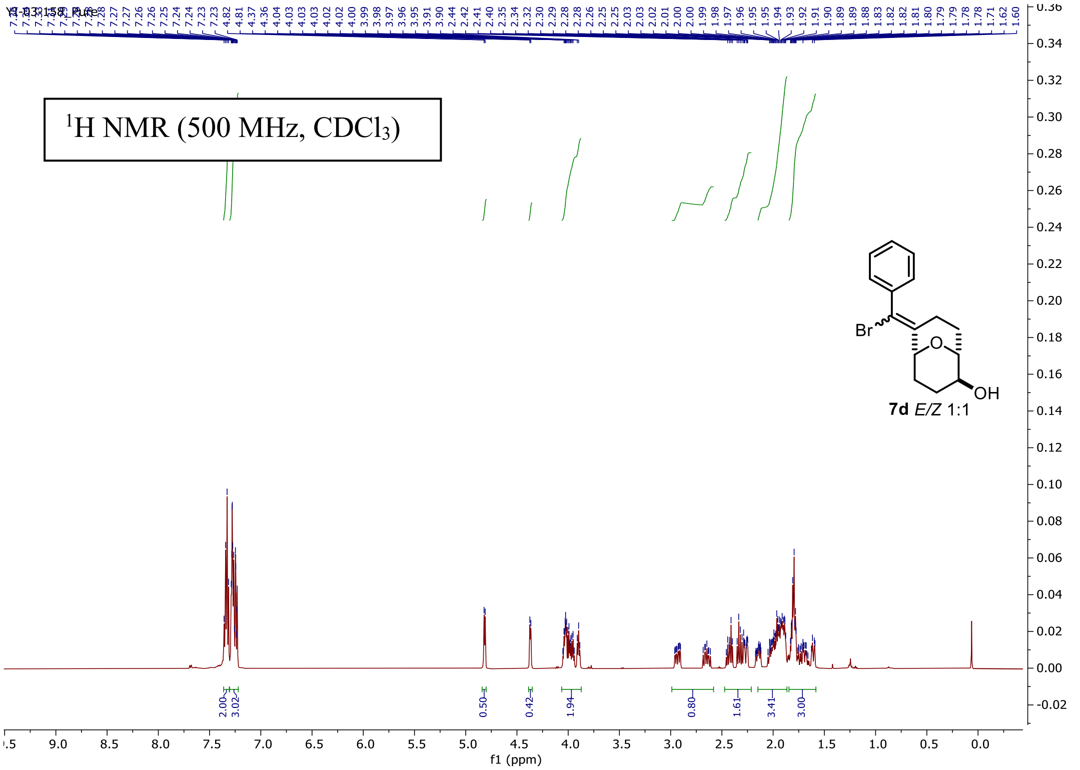
1H NMR (500 MHz, CDCl3)
Br
O
OH
7d E/Z 1:1
f1 (ppm)

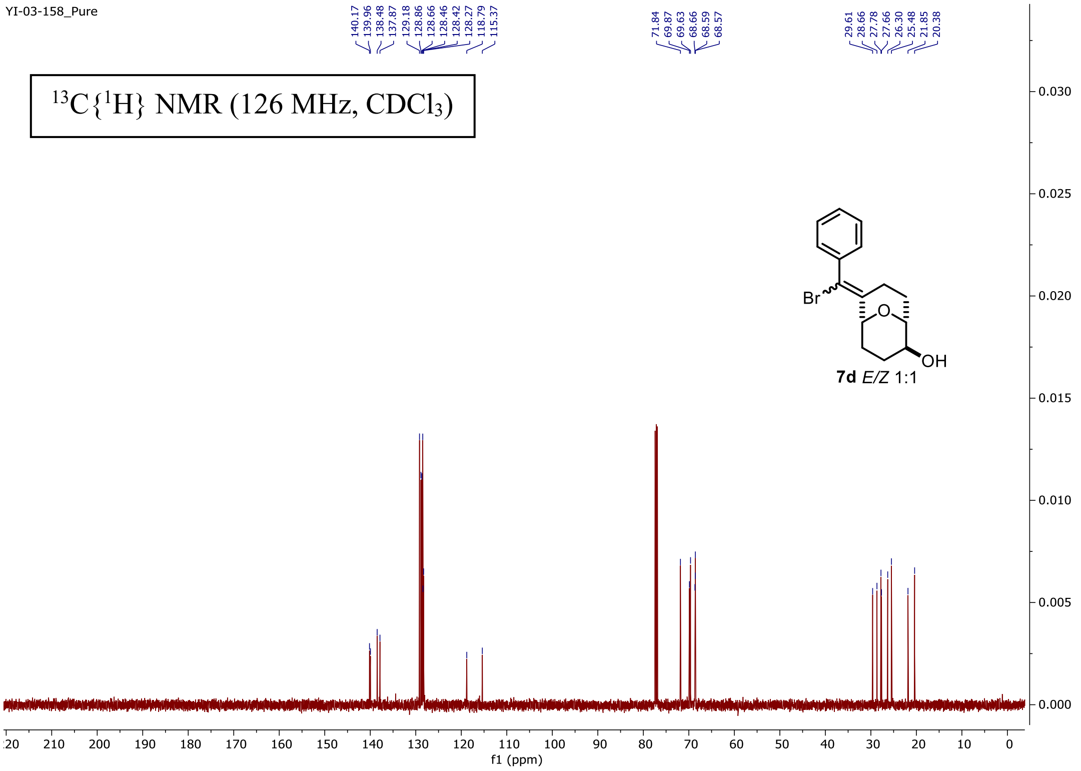
YI-03-158_Pure
140.17
139.96
138.48
137.87
129.18
128.86
128.66
128.46
128.42
128.27
118.79
115.37
71.84
69.87
69.63
68.66
68.59
68.57
29.61
28.66
27.78
27.66
26.30
25.48
21.85
20.38
13C{1H} NMR (126 MHz, CDCl3)
Br
O
OH
7d E/Z 1:1
f1 (ppm)

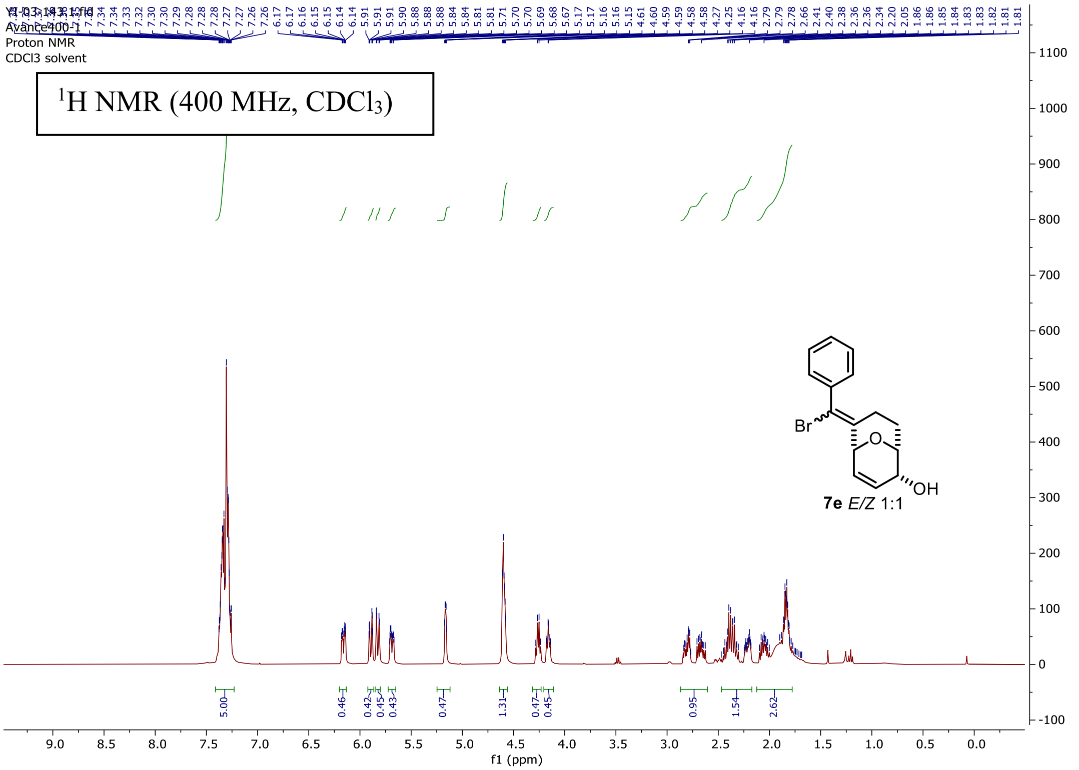
Avance400-1
Proton NMR
CDCl3 solvent
7.34 7.34 7.33 7.32 7.30 7.29 7.28 7.28 7.27 7.27 7.26 7.26 6.17 6.17 6.16 6.15 6.15 6.14 6.14 5.91 5.91 5.91 5.90 5.88 5.88 5.84 5.84 5.81 5.81 5.71 5.70 5.70 5.69 5.68 5.67 5.17 5.17 5.16 5.16 5.15 4.61 4.60 4.59 4.59 4.58 4.58 4.27 4.25 4.16 4.16 2.79 2.79 2.78 2.66 2.41 2.40 2.38 2.36 2.36 2.34 2.20 2.05 1.86 1.86 1.85 1.84 1.83 1.83 1.82 1.81 1.81
^{1}H NMR (400 MHz, CDCl$_3$)
Br
O
OH
7e E/Z 1:1
5.00 0.46 0.42 0.45 0.43 0.47 1.31 0.42 0.45 0.95 1.54 2.62
f1 (ppm)

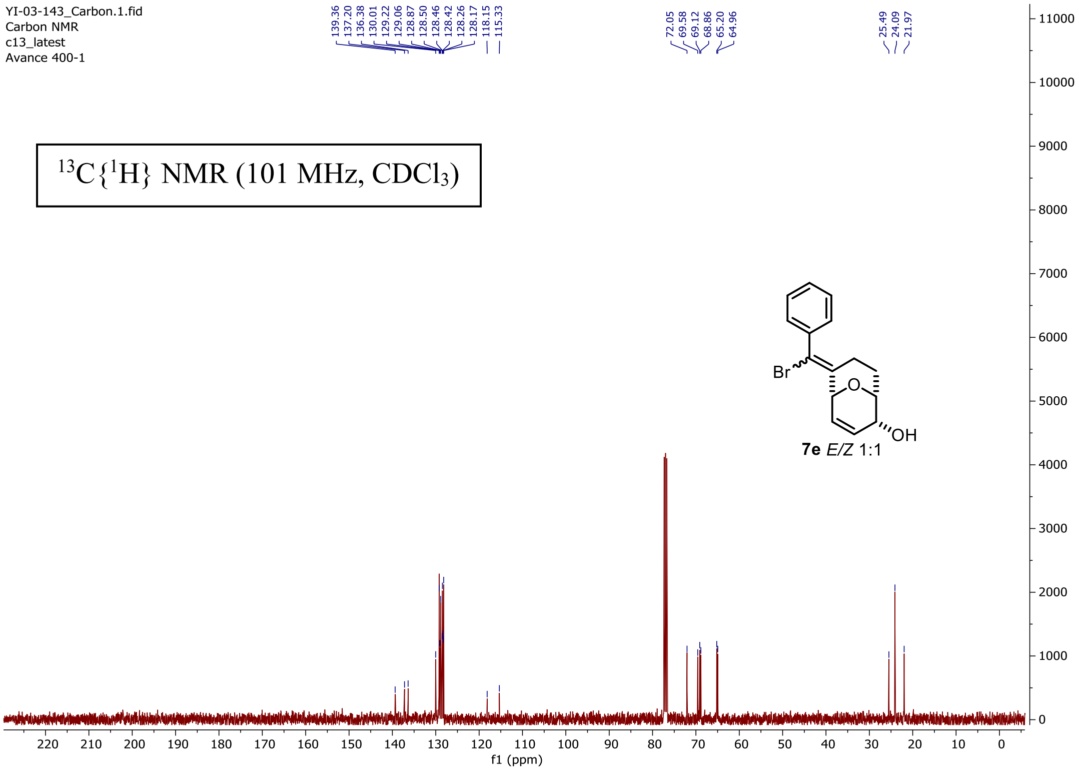
YI-03-143_Carbon.1.fid
Carbon NMR
c13_latest
Avance 400-1
139.36 137.20 136.38 130.01 129.22 129.06 128.87 128.50 128.46 128.42 128.26 128.17 118.15 115.33
72.05 69.58 69.12 68.86 65.20 64.96
25.49 24.09 21.97
^{13}C{^{1}H} NMR (101 MHz, CDCl$_3$)
Br
O
OH
7e E/Z 1:1
f1 (ppm)

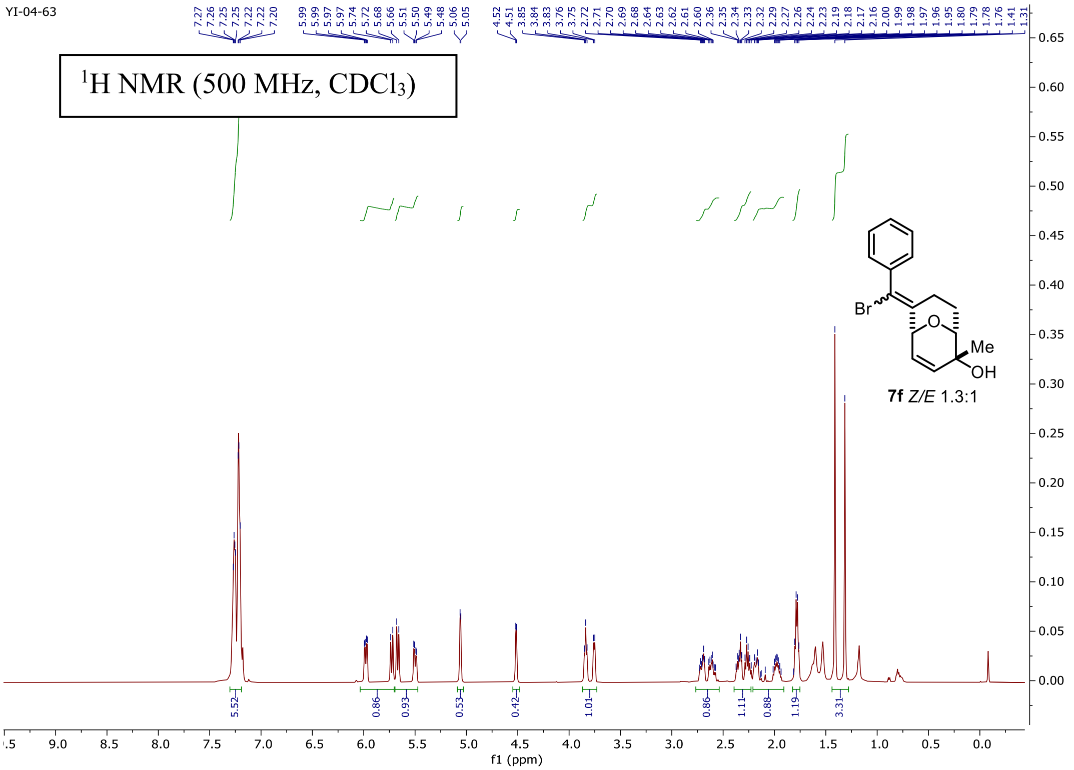
YI-04-63
¹H NMR (500 MHz, CDCl₃)
Br
O
Me
OH
7f Z/E 1.3:1
f1 (ppm)

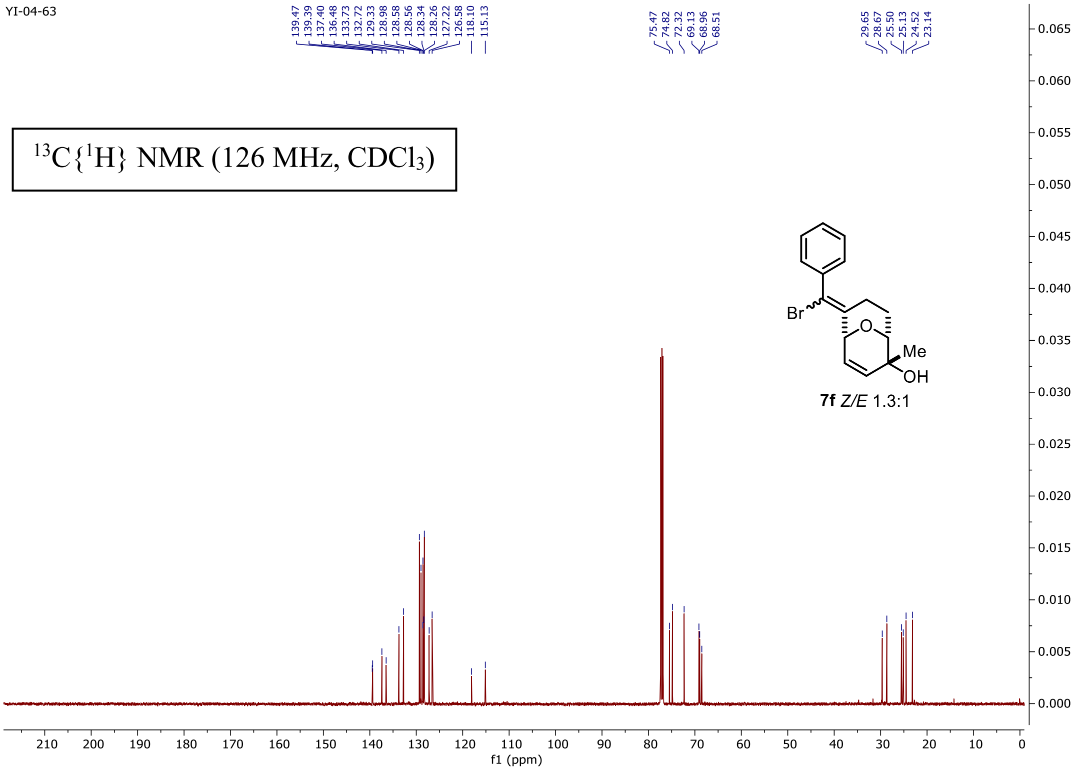
YI-04-63
¹³C{¹H} NMR (126 MHz, CDCl₃)
Br
O
Me
OH
7f Z/E 1.3:1
f1 (ppm)

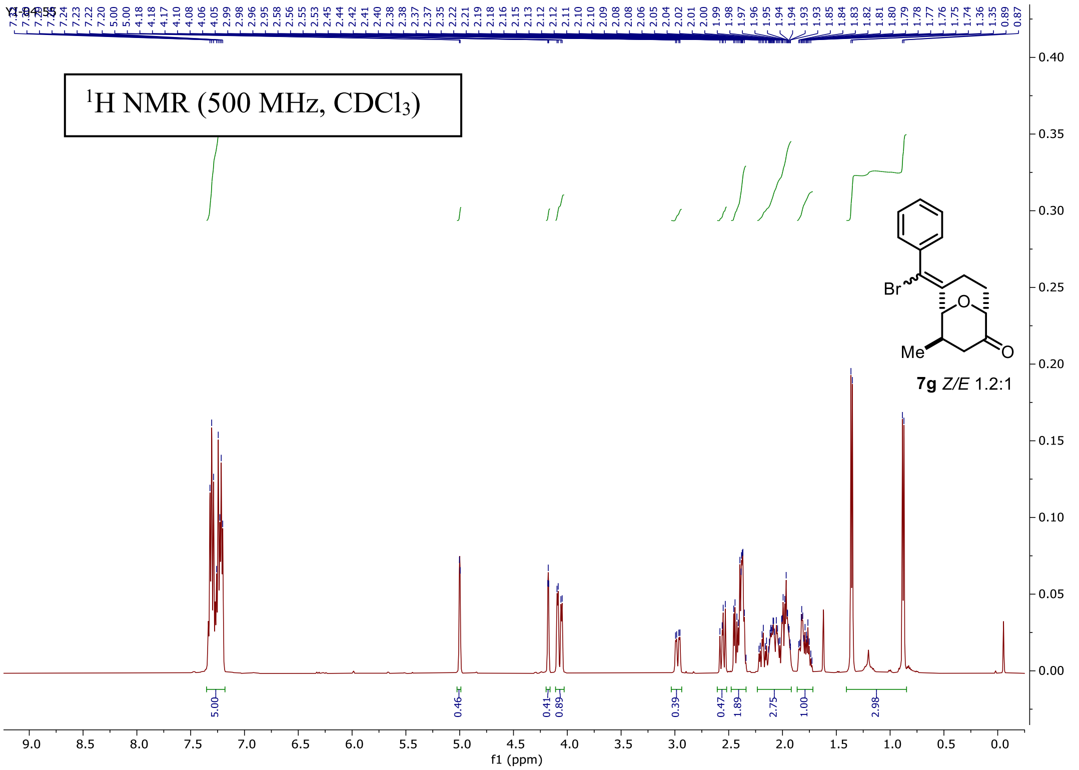

¹H NMR (500 MHz, CDCl3)
Br
O
Me
O
7g Z/E 1.2:1
f1 (ppm)

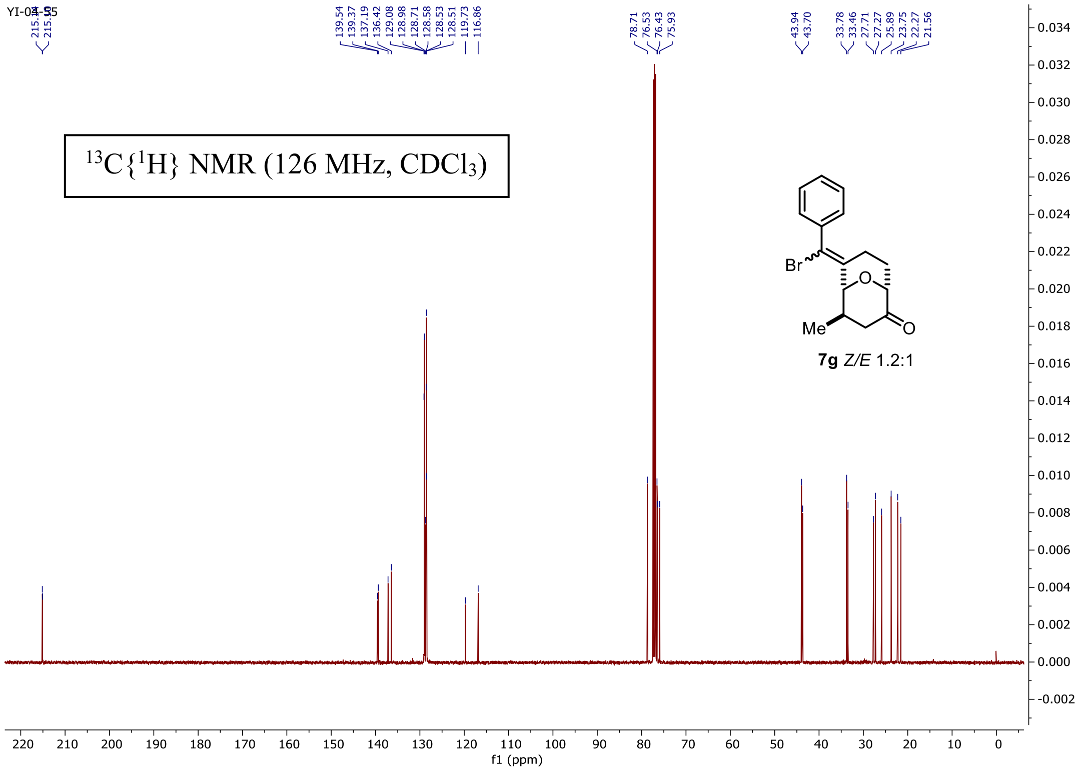

13C{1H} NMR (126 MHz, CDCl3)
Br
O
Me
O
7g Z/E 1.2:1
f1 (ppm)

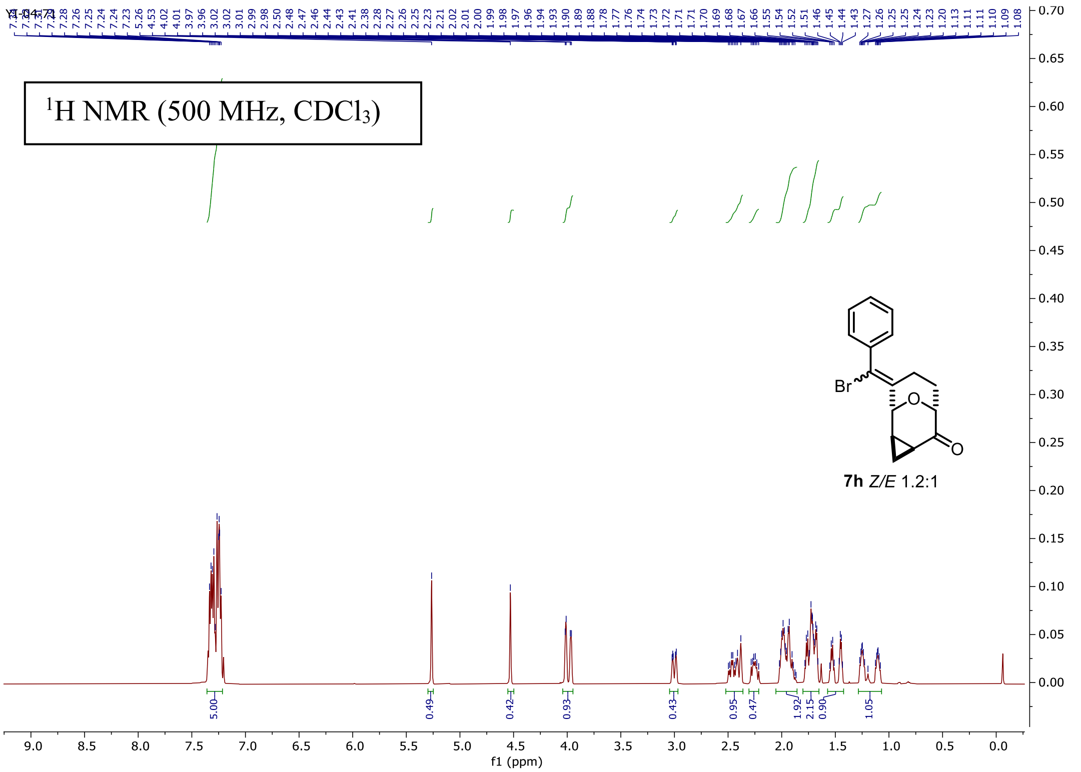
1H NMR (500 MHz, CDCl3)
Br
O
7h Z/E 1.2:1
f1 (ppm)

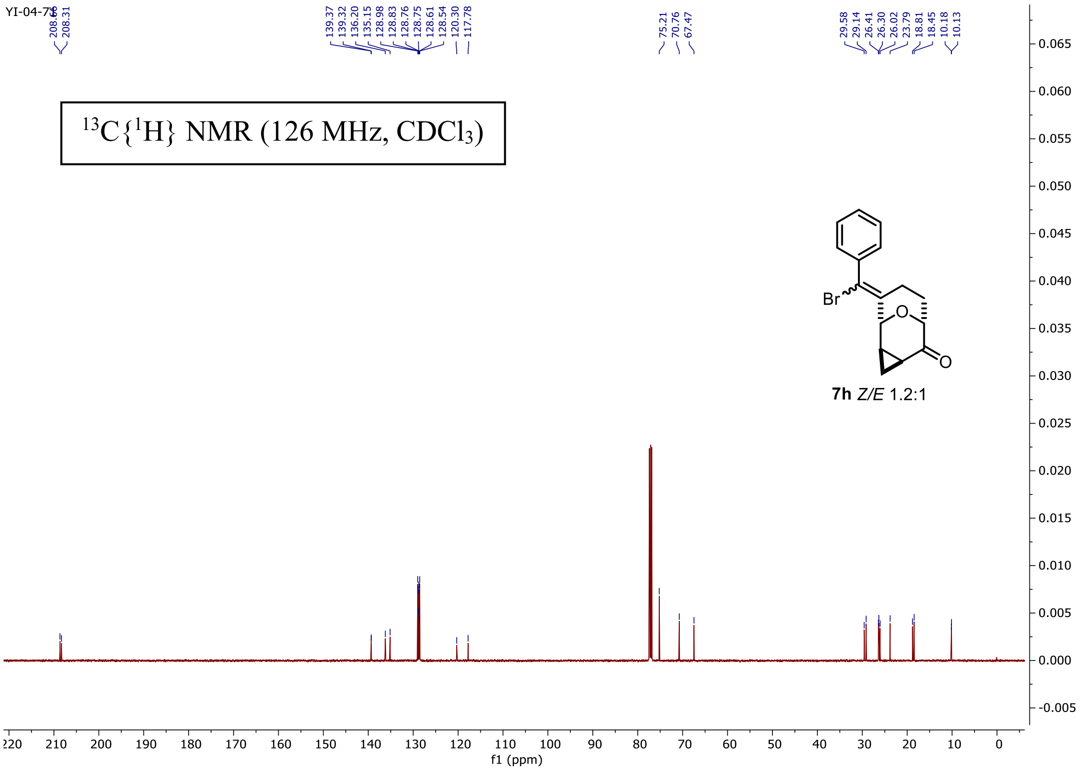
YI-04-71
208.31
139.37
139.32
136.20
135.15
128.98
128.83
128.76
128.75
128.61
128.54
120.30
117.78
75.21
70.76
67.47
29.58
29.14
26.41
26.30
26.02
23.79
18.81
18.45
10.18
10.13
13C{1H} NMR (126 MHz, CDCl3)
Br
O
7h Z/E 1.2:1
f1 (ppm)

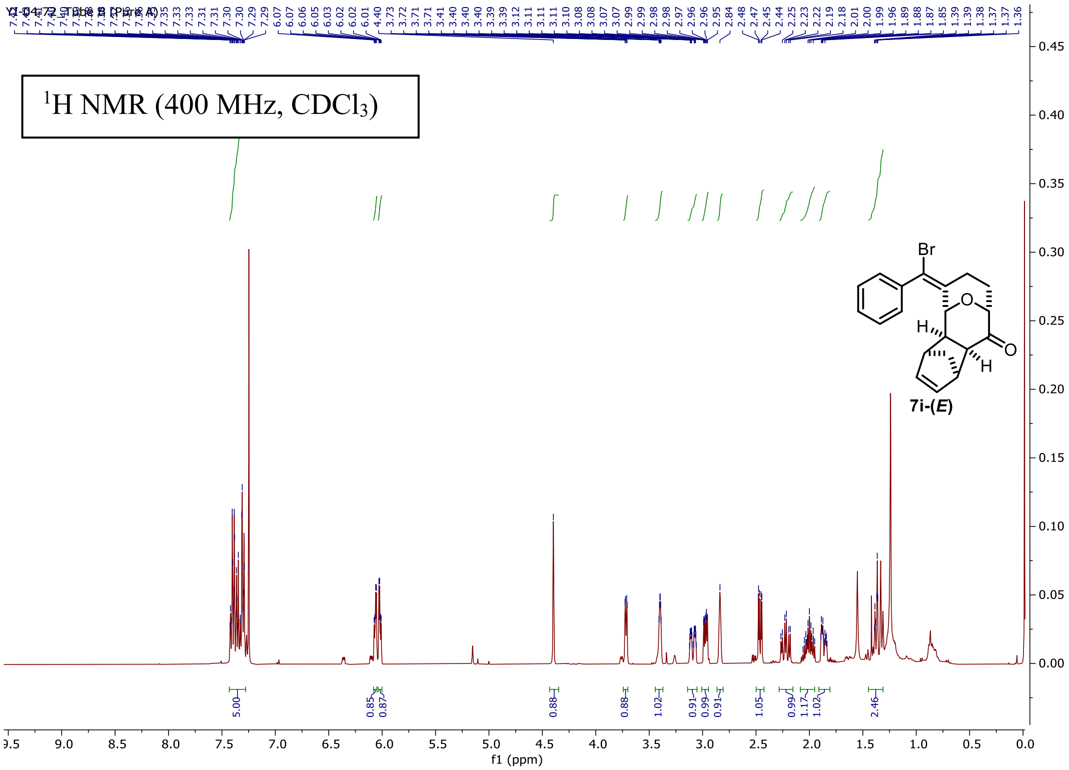

¹H NMR (400 MHz, CDCl₃)
Br
O
H
H
O
7i-(E)
f1 (ppm)

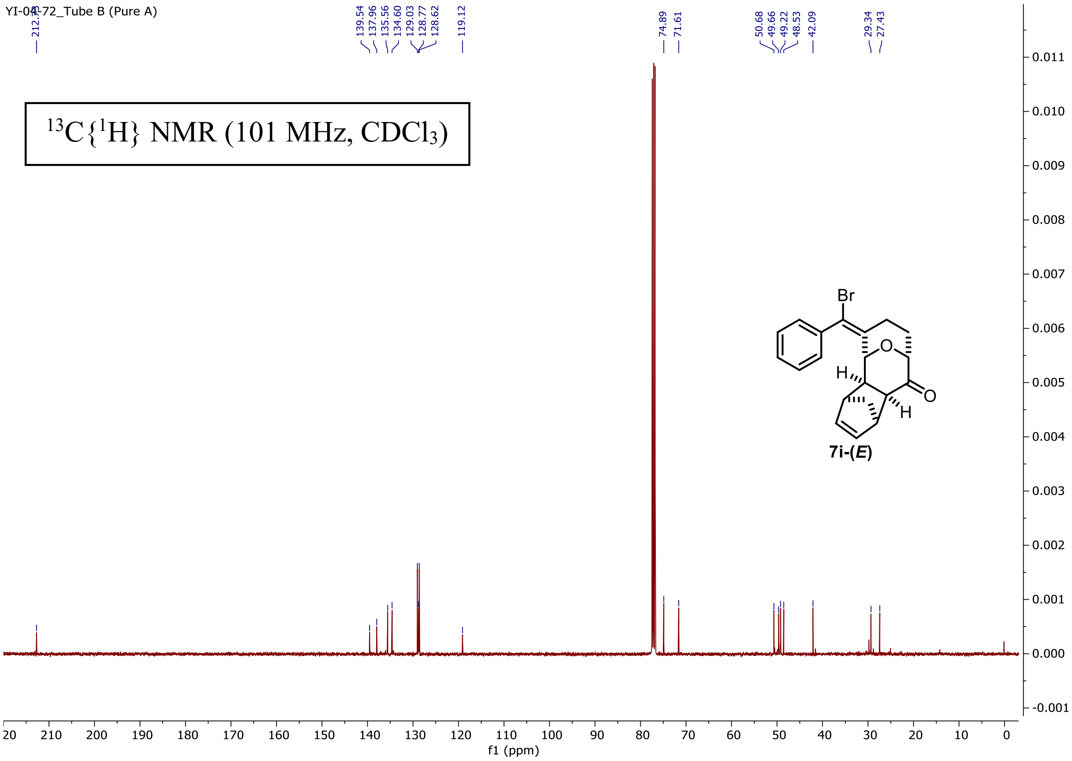

YI-04-72_Tube B (Pure A)
212.91
139.54
137.96
135.56
134.60
129.03
128.77
128.62
119.12
74.89
71.61
50.68
49.66
49.22
48.53
42.09
29.34
27.43
¹³C{¹H} NMR (101 MHz, CDCl₃)
Br
O
H
H
O
7i-(E)
f1 (ppm)

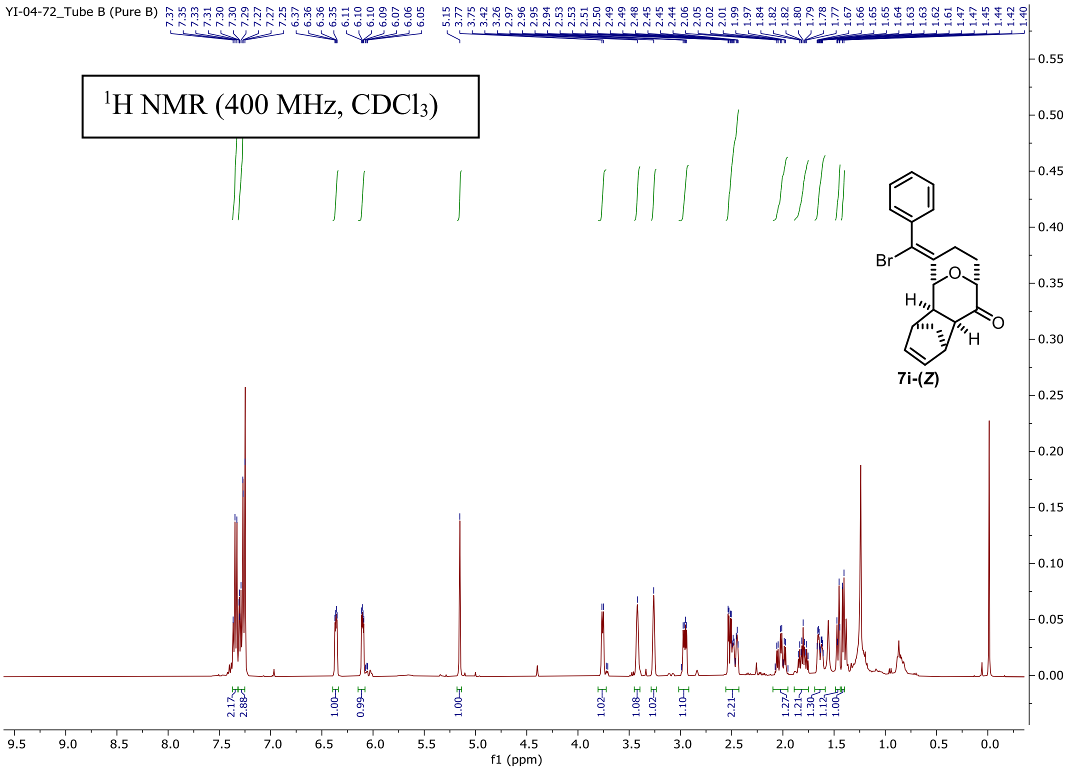
YI-04-72_Tube B (Pure B)
1H NMR (400 MHz, CDCl3)
7i-(Z)
f1 (ppm)

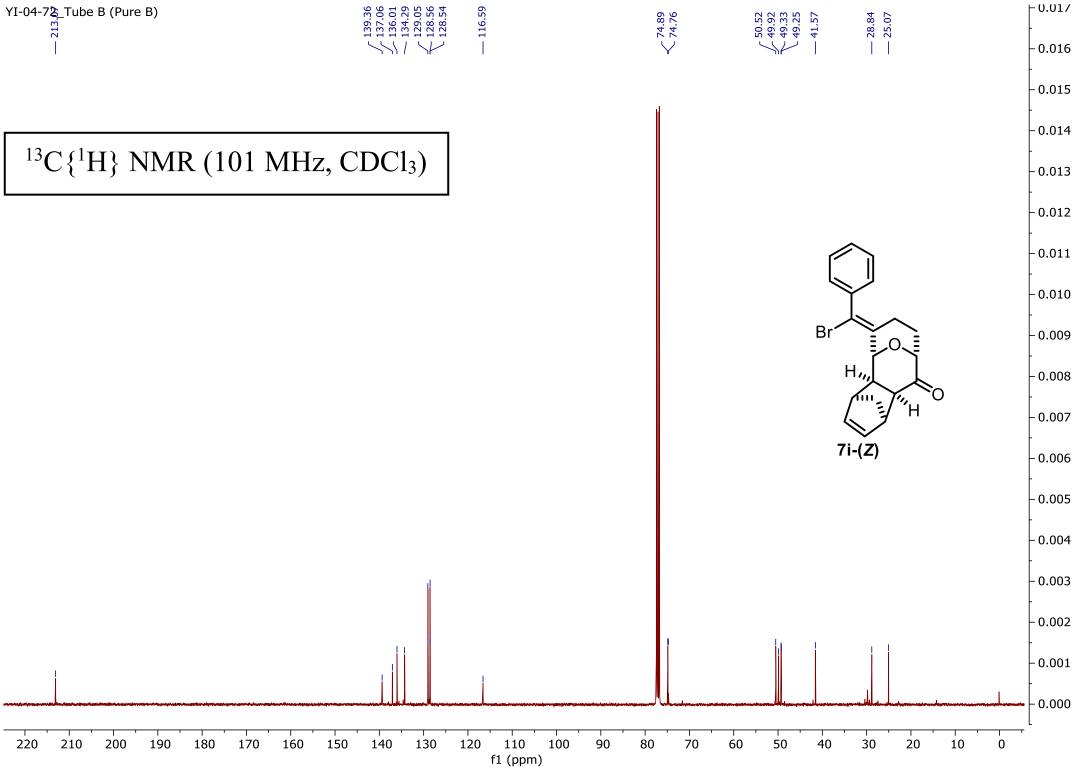
YI-04-72_Tube B (Pure B)
13C{1H} NMR (101 MHz, CDCl3)
7i-(Z)
f1 (ppm)

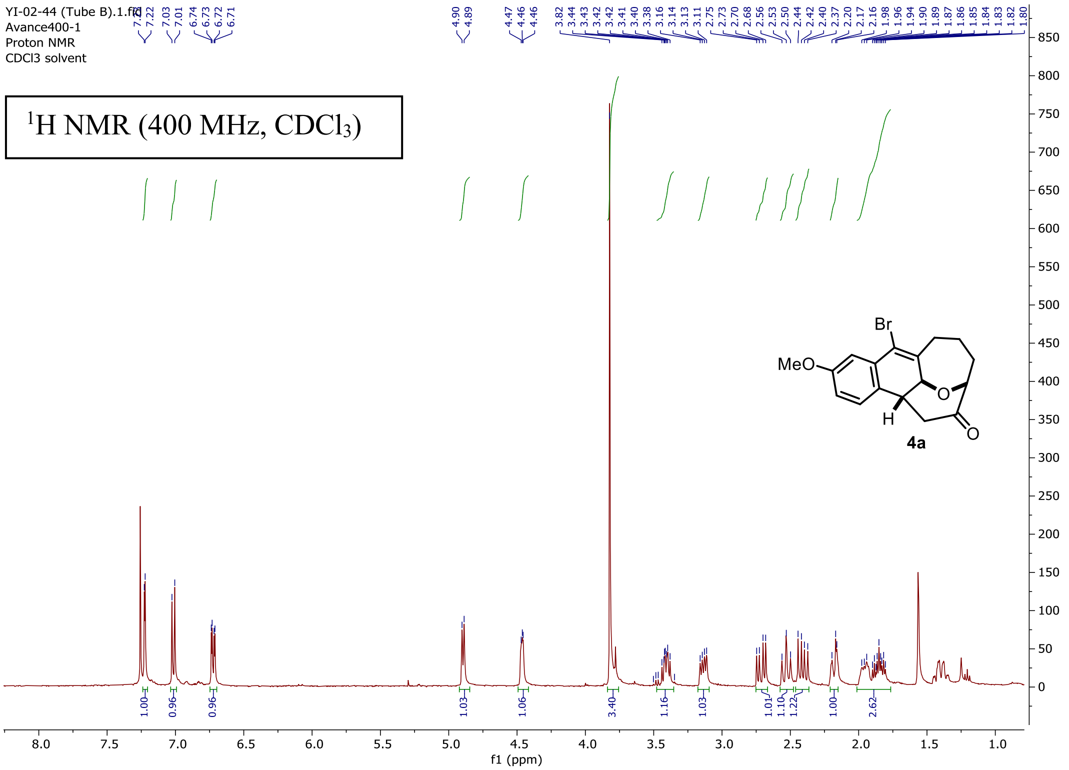
YI-02-44 (Tube B).1.fid
Avance400-1
Proton NMR
CDCl3 solvent
^{1}H NMR (400 MHz, CDCl$_3$)
MeO
Br
O
H
O
4a
f1 (ppm)

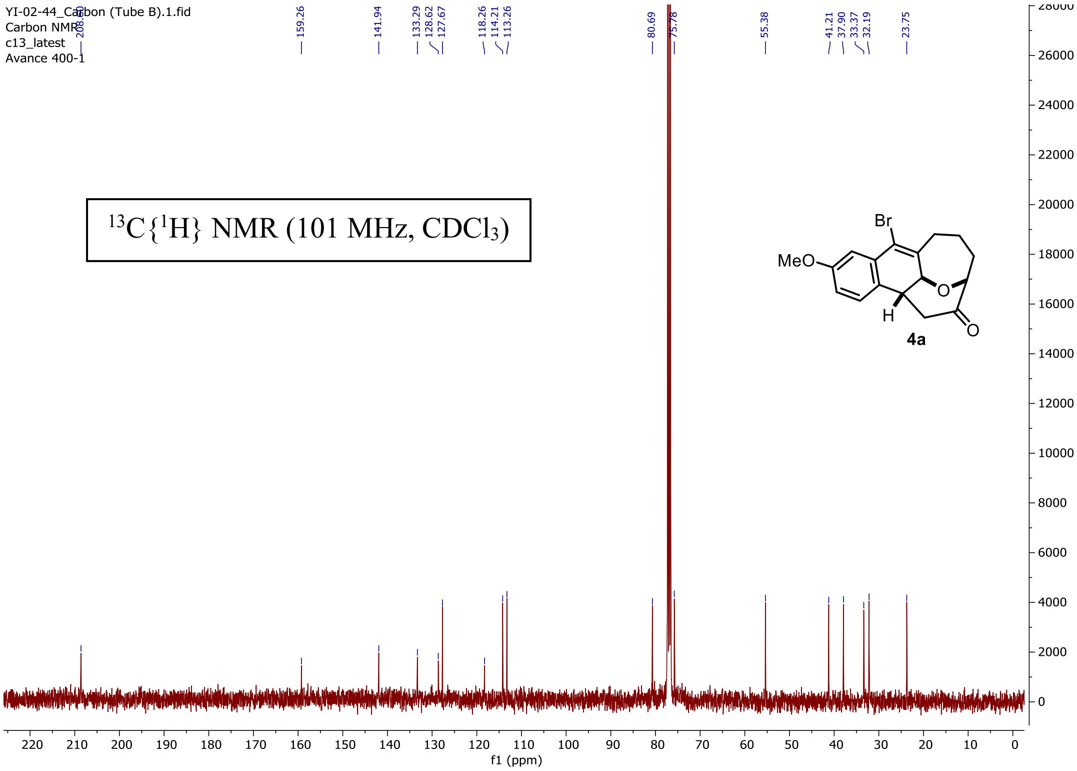
YI-02-44_Carbon (Tube B).1.fid
Carbon NMR
c13_latest
Avance 400-1
159.26
141.94
133.29
128.62
127.67
118.26
114.21
113.26
80.69
75.78
55.38
41.21
37.90
33.37
32.19
23.75
^{13}C{^{1}H} NMR (101 MHz, CDCl$_3$)
MeO
Br
O
H
O
4a
f1 (ppm)

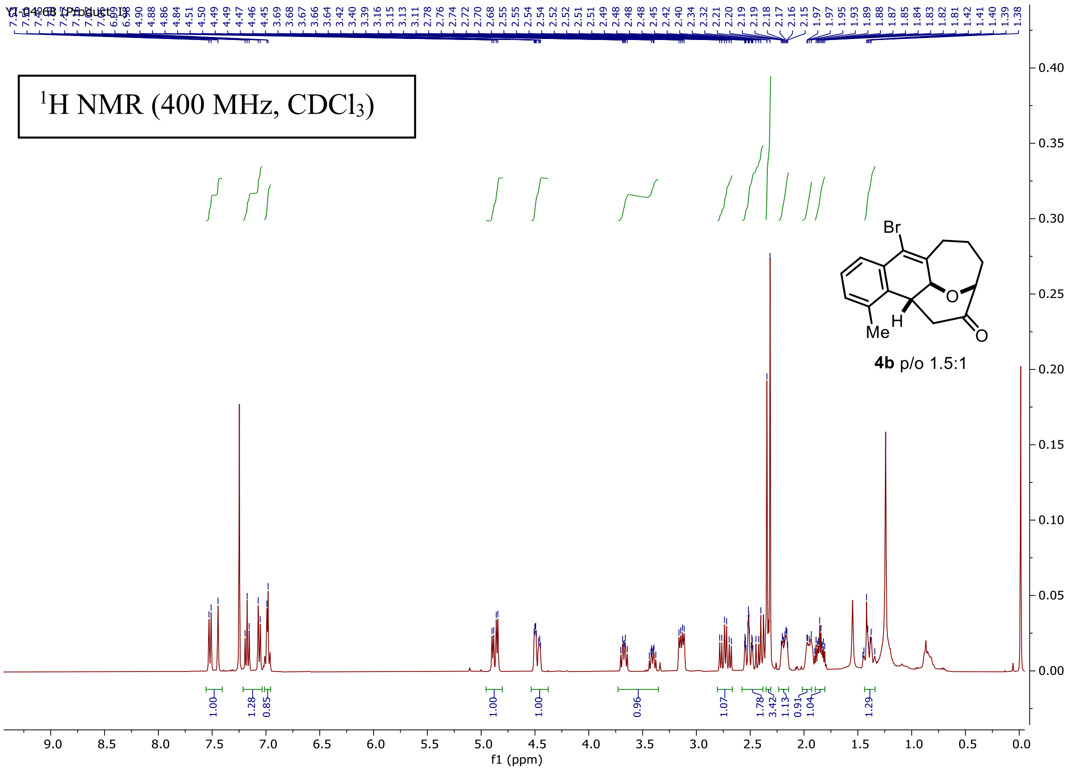
YI-04-68 (Product_I)
1H NMR (400 MHz, CDCl3)
Br
O
H
Me
O
4b p/o 1.5:1
f1 (ppm)

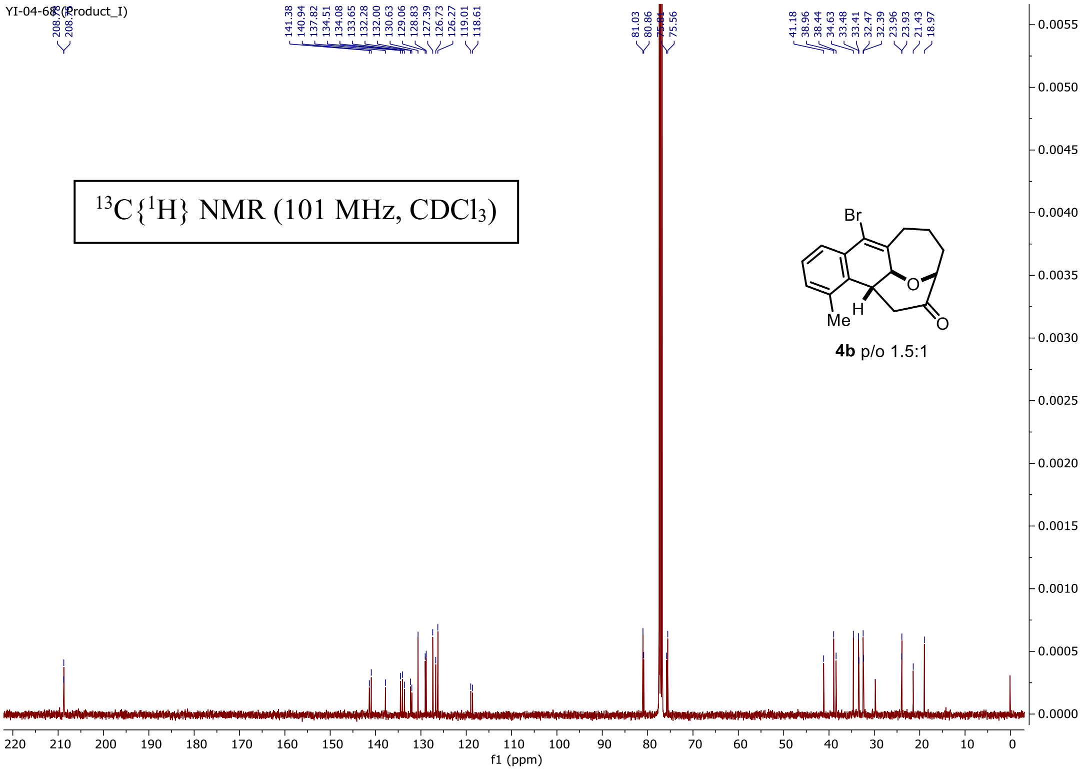
YI-04-68 (Product_I)
13C{1H} NMR (101 MHz, CDCl3)
Br
O
H
Me
O
4b p/o 1.5:1
f1 (ppm)

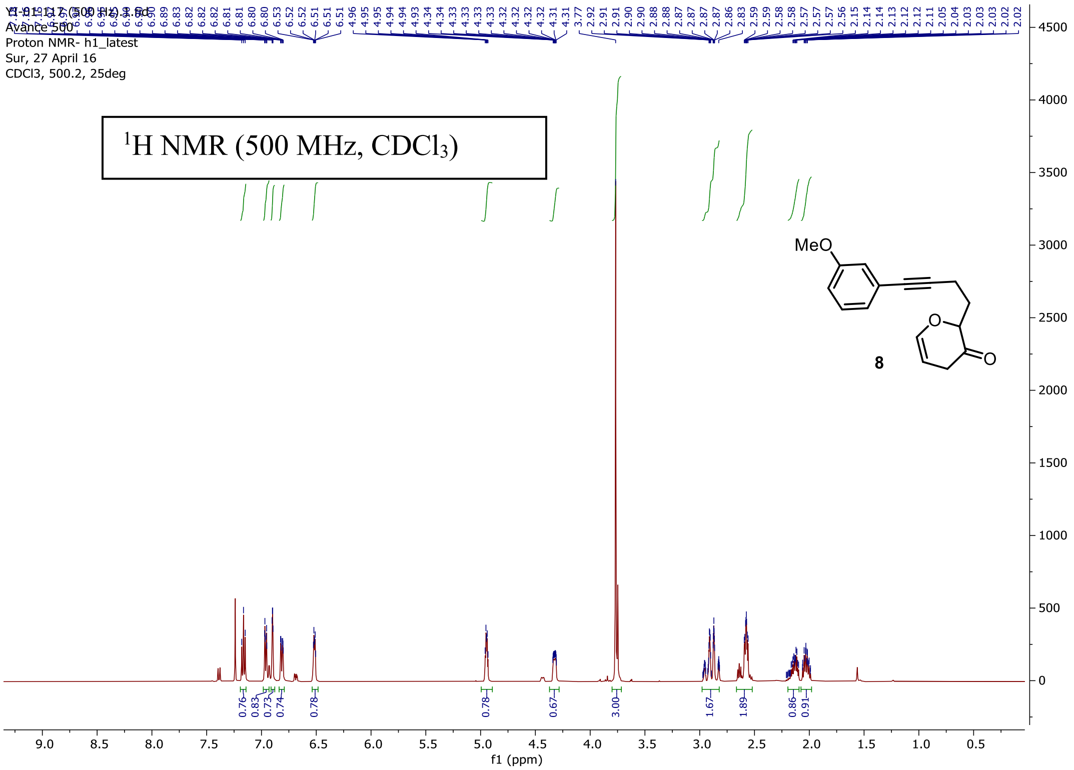
YI-01-117 (500 Hz).1.fid
Avance 500
Proton NMR- h1_latest
Sur, 27 April 16
CDCl3, 500.2, 25deg
^{1}H NMR (500 MHz, CDCl$_3$)
MeO
O
O
8
f1 (ppm)

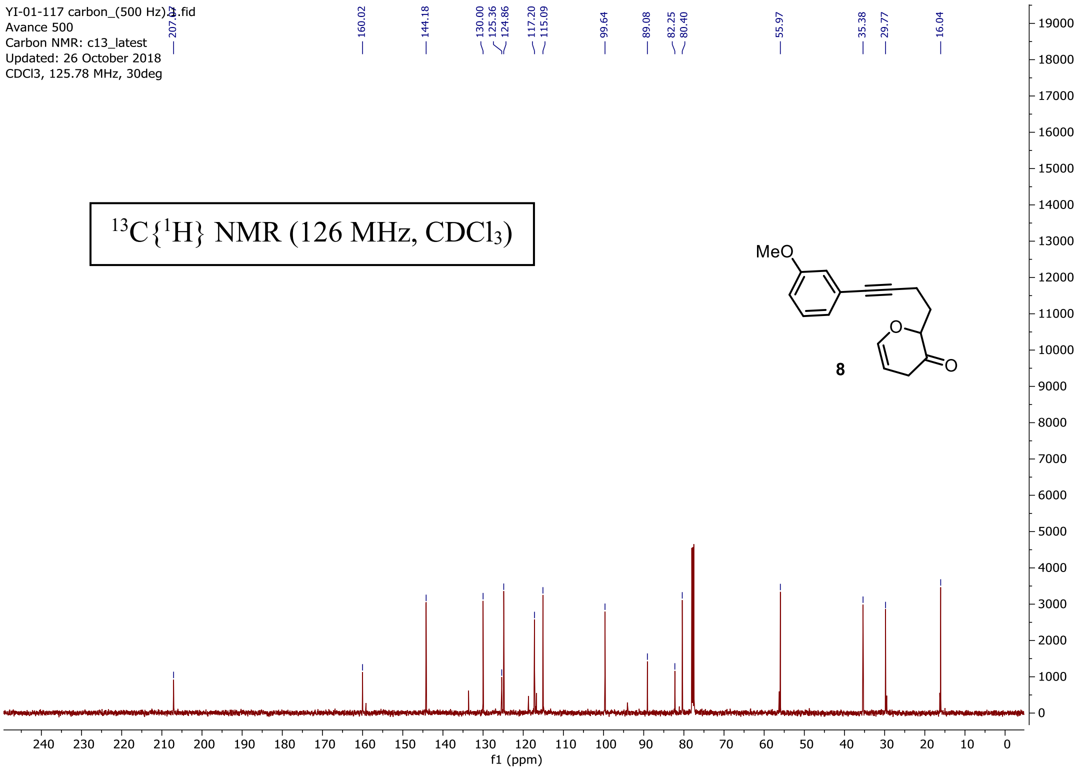
YI-01-117 carbon_(500 Hz).1.fid
Avance 500
Carbon NMR: c13_latest
Updated: 26 October 2018
CDCl3, 125.78 MHz, 30deg
207.3
160.02
144.18
130.00
125.36
124.86
117.20
115.09
99.64
89.08
82.25
80.40
55.97
35.38
29.77
16.04
^{13}C{^{1}H} NMR (126 MHz, CDCl$_3$)
MeO
O
O
8
f1 (ppm)

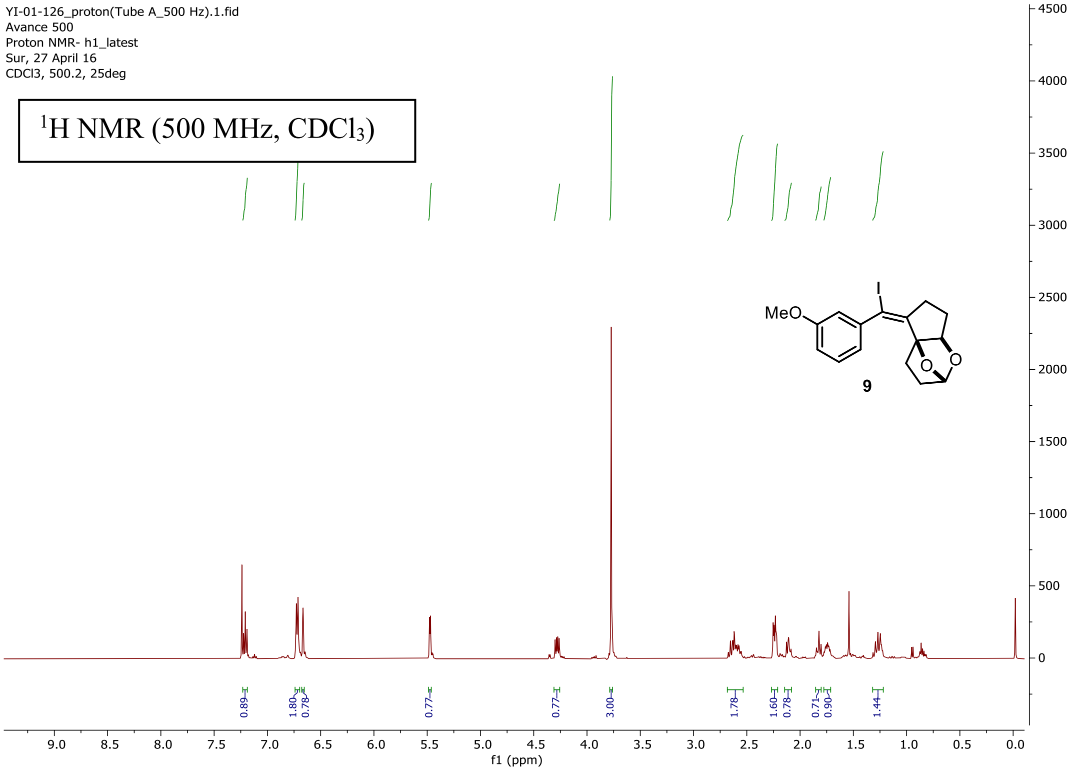
YI-01-126_proton(Tube A_500 Hz).1.fid
Avance 500
Proton NMR- h1_latest
Sur, 27 April 16
CDCl3, 500.2, 25deg
^{1}H NMR (500 MHz, $CDCl_3$)
MeO
I
O
O
9
0.89
1.80
0.78
0.77
0.77
3.00
1.78
1.60
0.78
0.71
0.90
1.44
f1 (ppm)

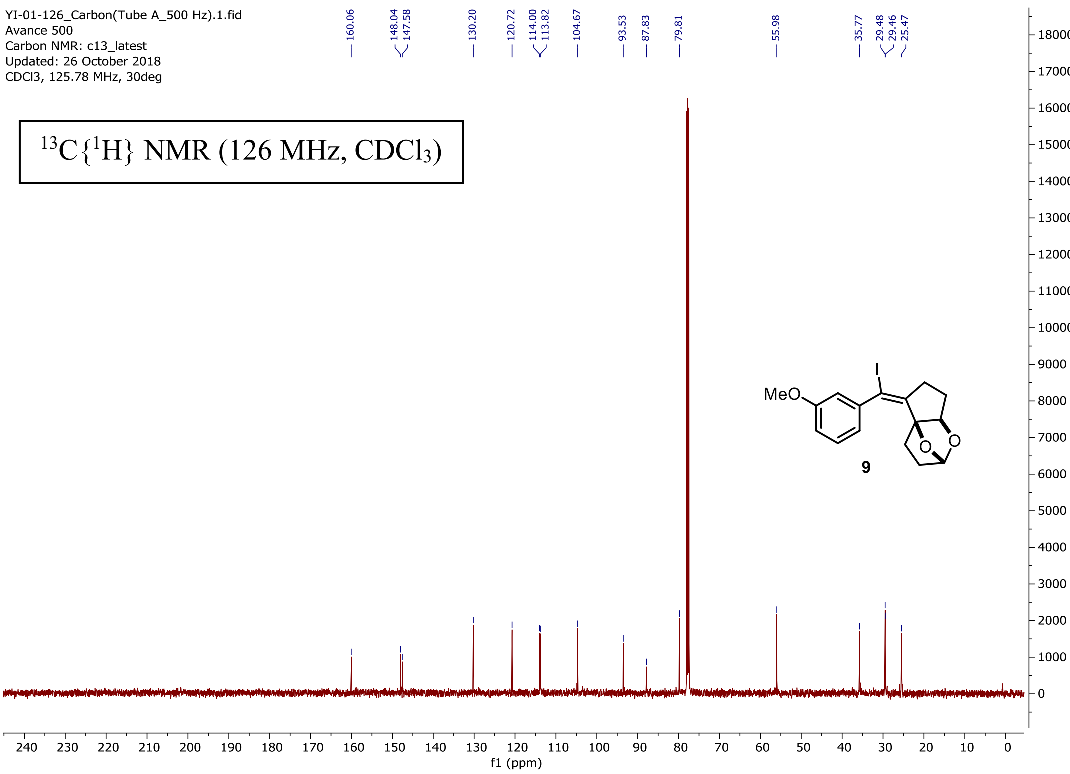
YI-01-126_Carbon(Tube A_500 Hz).1.fid
Avance 500
Carbon NMR: c13_latest
Updated: 26 October 2018
CDCl3, 125.78 MHz, 30deg
160.06
148.04
147.58
130.20
120.72
114.00
113.82
104.67
93.53
87.83
79.81
55.98
35.77
29.48
29.46
25.47
^{13}C{^{1}H} NMR (126 MHz, $CDCl_3$)
MeO
I
O
O
9
f1 (ppm)

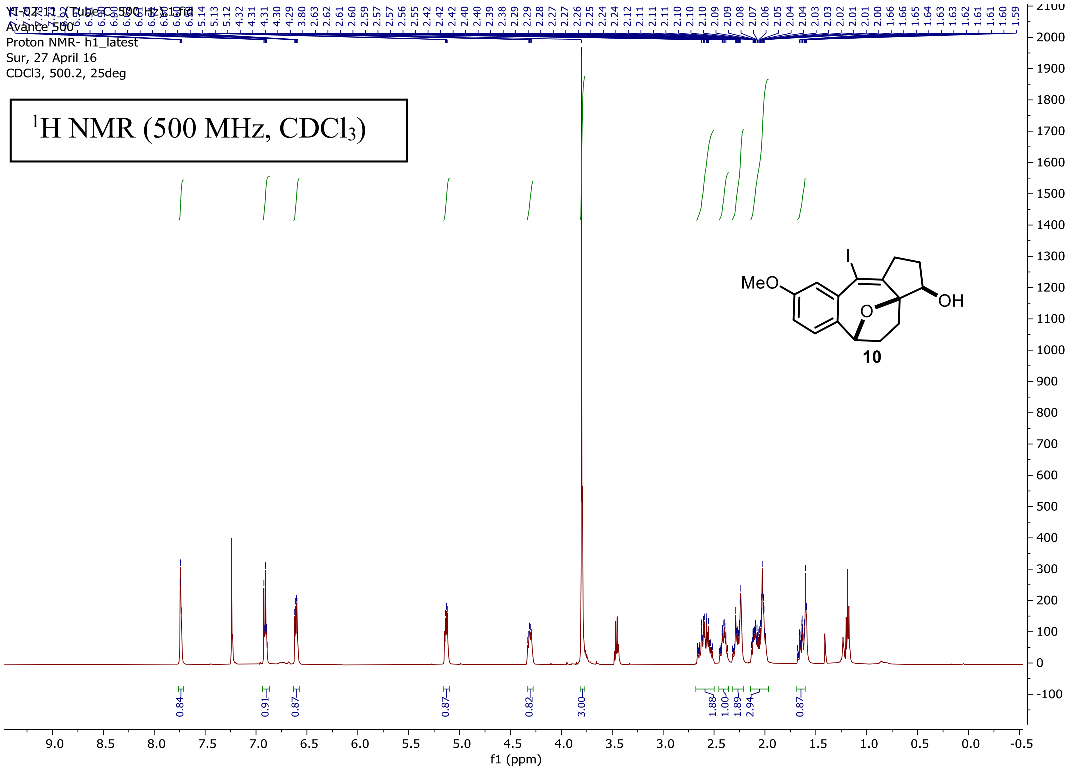
YI-02-11_(Tube C_500 Hz).1.fid
Avance 500
Proton NMR- h1_latest
Sur, 27 April 16
CDCl3, 500.2, 25deg
1H NMR (500 MHz, CDCl3)
MeO
I
OH
O
10
f1 (ppm)

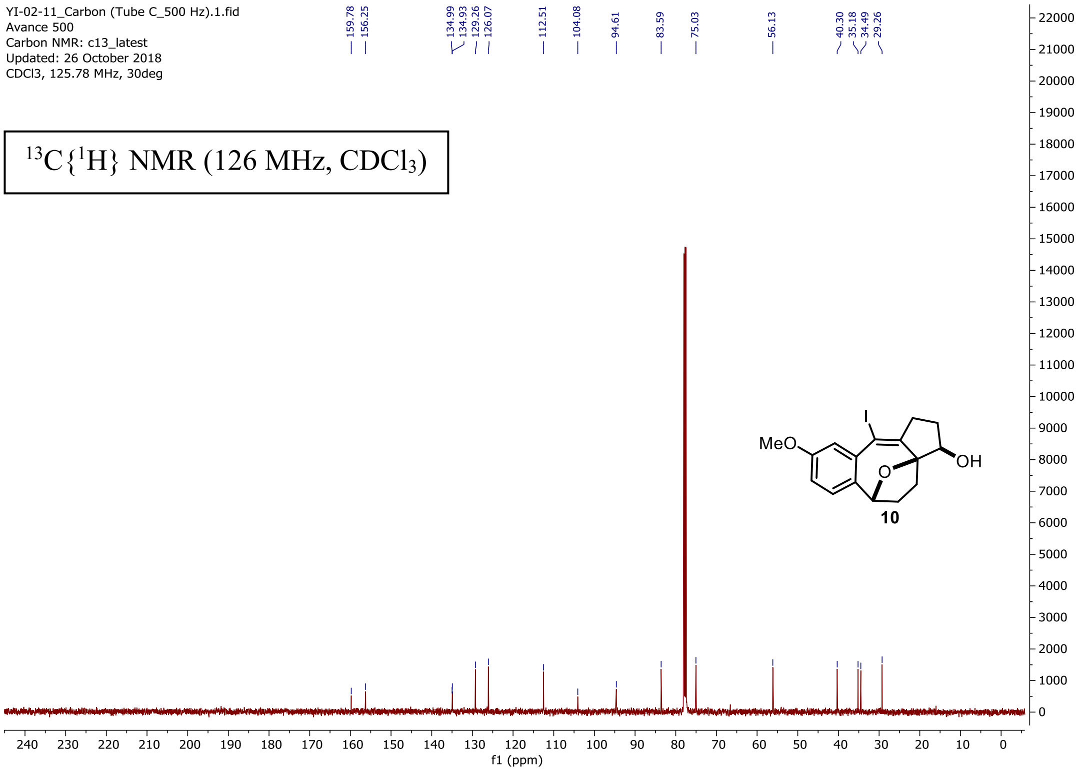
YI-02-11_Carbon (Tube C_500 Hz).1.fid
Avance 500
Carbon NMR: c13_latest
Updated: 26 October 2018
CDCl3, 125.78 MHz, 30deg
159.78
156.25
134.99
134.93
129.26
126.07
112.51
104.08
94.61
83.59
75.03
56.13
40.30
35.18
34.49
29.26
13C{1H} NMR (126 MHz, CDCl3)
MeO
I
OH
O
10
f1 (ppm)
